# δ C–H (hetero)arylation *via* Cu-catalyzed radical relay†
†Electronic supplementary information (ESI) available. See DOI: 10.1039/c8sc04366c


**DOI:** 10.1039/c8sc04366c

**Published:** 2018-11-09

**Authors:** Zuxiao Zhang, Leah M. Stateman, David A. Nagib

**Affiliations:** a The Ohio State University , Department of Chemistry and Biochemistry , Columbus , OH 43210 , USA . Email: nagib.1@osu.edu

## Abstract

A Cu-catalyzed strategy has been developed that harnesses a radical relay mechanism to intercept a distal C-centered radical for C–C bond formation. This approach enables selective δ C–H (hetero)arylation of sulfonamides *via* intramolecular hydrogen atom transfer (HAT) by an N-centered radical. The radical relay is both initiated and terminated by a Cu catalyst, which enables incorporation of arenes and heteroarenes by cross-coupling with boronic acids. The broad scope and utility of this catalytic method for δ C–H arylation is shown, along with mechanistic probes for selectivity of the HAT mechanism. A catalytic, asymmetric variant is also presented, as well as a method for accessing 1,1-diaryl-pyrrolidines *via* iterative δ C–H functionalizations.

## Abstract 2

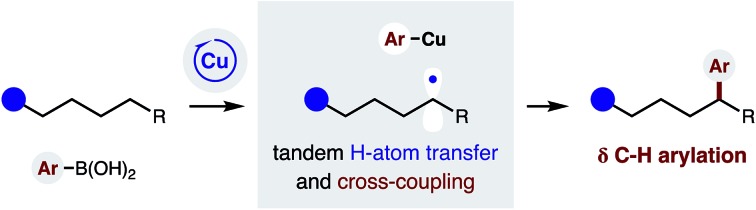
A radical relay strategy has been developed to enable selective δ C–H arylation. The approach employs a chiral copper catalyst, which serves the dual roles of generating an N-centered radical to promote intramolecular H-atom transfer, and then intercepting a distal C-centered radical for C–C bond formation with (hetero)aryl boronic acids.

## Introduction

**Fig. 1:**
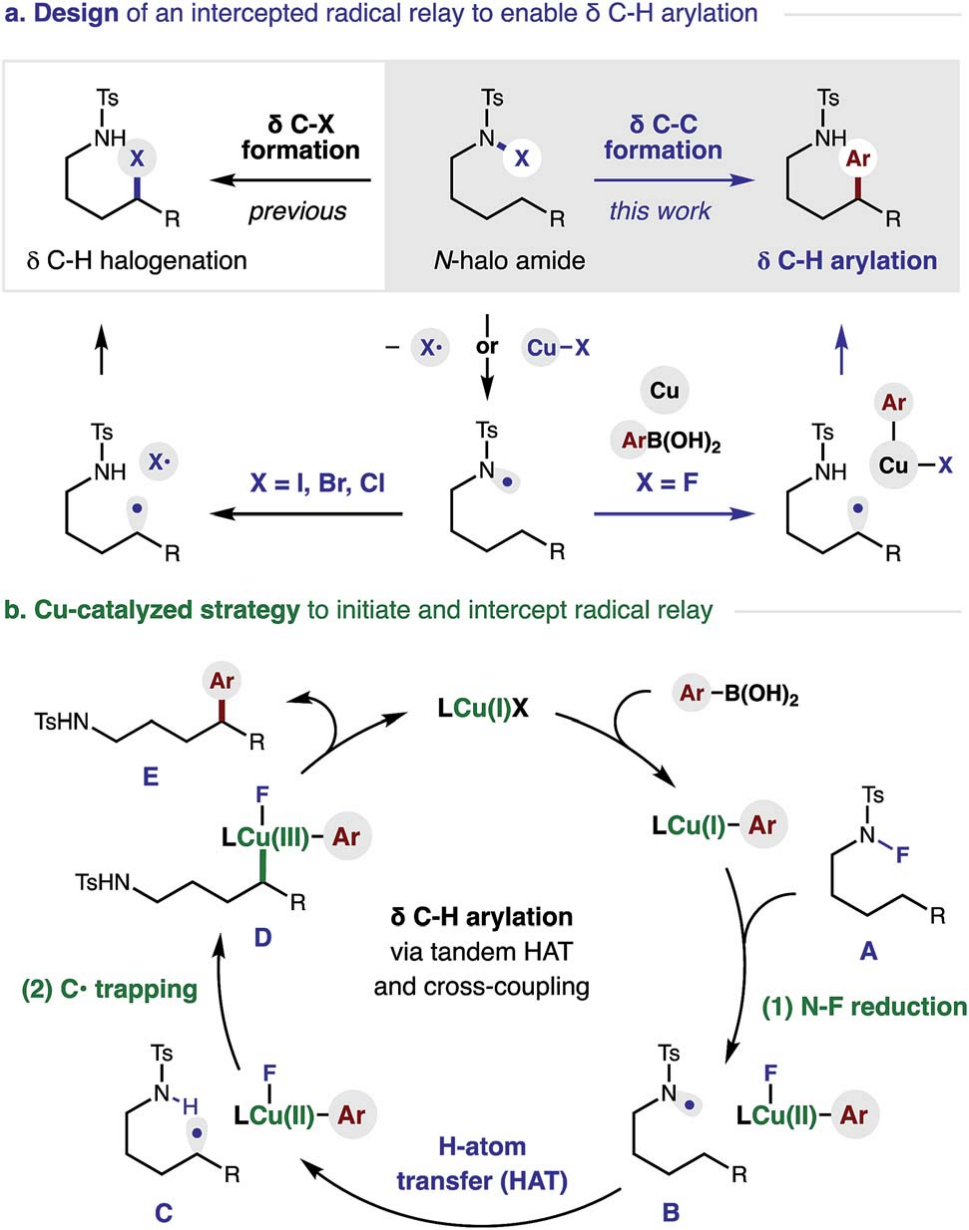
Cu-catalyzed radical relay enables δ C–H arylation.

The remote C–H functionalization of amines *via* intramolecular hydrogen atom transfer (HAT) has enabled a distinct approach to the synthesis of pyrrolidines for over a century.^1^,^2^ Yet, while this formal δ C–H amination has been interrupted to afford distal halogenation and oxygenation, it has rarely enabled δ C–C bond formation.^3^–^5^ A mechanistic explanation is that initiation of this radical rearrangement requires homolysis of an N-halo amide to generate the N-centered radical (Fig. 1a). Following selective 1,5-HAT, the translocated δ C˙ rapidly combines with the solvent-caged halide radical (X˙). Finally, intramolecular displacement of the resultant δ C–X bond is then spontaneous if X = I (or requires a strong base if X = Br, Cl). Notably, radical recombination to form C–X is rapid; and we have exclusively observed δ halogenation – even when this reaction is performed with a radical trap (*e.g.* acrylonitrile) as solvent. Given this challenge, the first examples of intercepting this N˙ to C˙ relay for C–C bond formation were only reported recently.^4^ Notably, these solutions (mostly entailing δ addition to acrylates) forgo the intermediacy of X˙ entirely; and instead, the N˙ is generated from an N–H or N–O bond.^4^,^5^

We proposed the interruption of this century-old X˙ rearrangement could also be facilitated by use of an N–F precursor. Since F˙ is highly unstable,^6^ N–F homolysis (and ensuing radical recombination with δ C˙) seemed unfavorable.^7^ In fact, a recent example of δ fluorination by HAT required an Fe-catalyzed protocol.^8^ Instead, we anticipated N–F reduction could be mediated by a Cu catalyst that would also enable Suzuki–Miyaura coupling of the distal organocopper with aryl boronic acids.^9^ A Cu-mediated pathway for C˙ formation and subsequent arylation also appeared viable – based on pioneering work by Liu and Stahl, who incorporated these elementary steps in their arylation of benzylic C–H bonds.^10^

In designing a strategy to enable the first δ C–H arylation *via* a radical relay mechanism,^11^–^14^ we proposed a Cu catalyst could serve the dual roles of radical initiation and aryl trapping of the distal C˙ (Fig. 1b). In our proposed mechanism, an *in situ* generated Cu(i) complex undergoes transmetallation with a (hetero)aryl boronic acid to afford a Cu(i)Ar species. We expected this more electron-rich Cu complex to be well-suited to initiate reduction of the N–F bond of amide **A***via* either a single-electron-transfer or atom-transfer mechanism. The resultant N-centered radical **B** would then undergo selective 1,5-HAT to afford δ C˙ amide **C**. In the second vital role of the Cu catalyst, an oxidized Cu(ii)Ar complex could combine with C˙ in the mechanism described by Kochi,^15^ as well as Liu.10*e* The highly oxidized organometallic **D** should then reductively eliminate Cu(i) and δ aryl amide **E**. This final step simultaneously affords turnover of the catalytic cycle and sp^3^–sp^2^ C–C coupling. Importantly, we were intrigued by the possibility that ligand tunability could enable control of both reactivity and stereoselectivity in this δ C–H arylation.

## Results and discussion

**Fig. 2:**
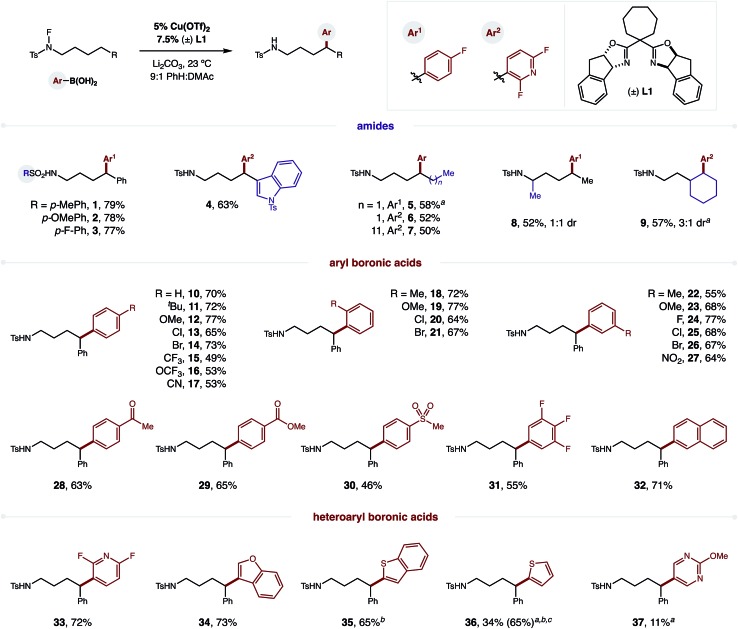
Scope of δ C–H (hetero)arylation by Cu-catalyzed radical relay and coupling with aryl boronic acids. Conditions: sulfonamide (0.2 mmol), arylboronic acid (2 equiv.), Li_2_CO_3_ (1 equiv.), 5% Cu(OTf)_2_, 7.5% (±) L1, PhH : DMAc (4 mL; 9 : 1), r.t. isolated yields. ^*a*^NMR yield. ^*b*^10% Cu(OTf)_2_, 15% (±) L1.^*c*^Based on recovered starting material.

To test our hypothesis, we combined *p*-F-phenylboronic acid, an N-fluoro-tosylamide, and 5% Cu(OTf)_2_ (Fig. 2). Upon optimization of ligand, base, and solvent mixtures (all crucial factors to enable maximum reaction efficiency, see ESI† for details), we were pleased to find the radical relay mechanism could indeed be interrupted to afford δ arylation (**1**, 79%). Control experiments reveal that both bisoxazoline ligand (±) **L1** and an aryl boronic acid are necessary for efficient amide consumption, suggesting the Cu complex requires both donating ligands to reduce the N–F.

We next turned our attention to investigating the scope and generality of this δ C–H arylation (Fig. 2). Interestingly, electronic variation of the sulfonamide does not greatly affect reaction efficiency (**1–3**). Heteroarenes, such as indole, can be incorporated on the amide (**4**; without 6-*exo*-trig cyclization), or within the aryl boronic acid (**Ar^2^**). In the latter case, we found the difluoropyridyl arene products to be particularly easy to isolate. Notably, we found that it is not necessary to abstract the H-atom from a benzylic position. In fact, secondary C–H bonds are readily arylated (**5–9**), in accordance with our previous work on δ C–H aminations.^16^ α-Branched amides are also viable (**8**), although they afford lower diastereoselectivity (1 : 1) than γ substitution (**9**, 3 : 1).

To showcase the synthetic utility of employing aryl boronic acids within this remote Cu-catalyzed cross-coupling, we embarked on an exhaustive investigation of this component. As shown in Fig. 2, this δ arylation is amenable to electronic perturbation of the ArB(OH)_2_ – with both donating and accepting groups tolerated (**10–31**) – ranging from –OMe to –NO_2_ and including orthogonal functional handles, such as bromides, ketones, esters, and sulfones. Similarly, both *ortho* and *meta* substitution, as well as polyaromatics (**32**), readily participate in this reaction. As further demonstration of the likely utility of this approach to the synthesis of medicinal agents, we employed a family of heteroaryl boronic acids, including those containing N, O, S, and F, and all are amenable to this δ C–H arylation (**33–37**).

**Fig. 3:**
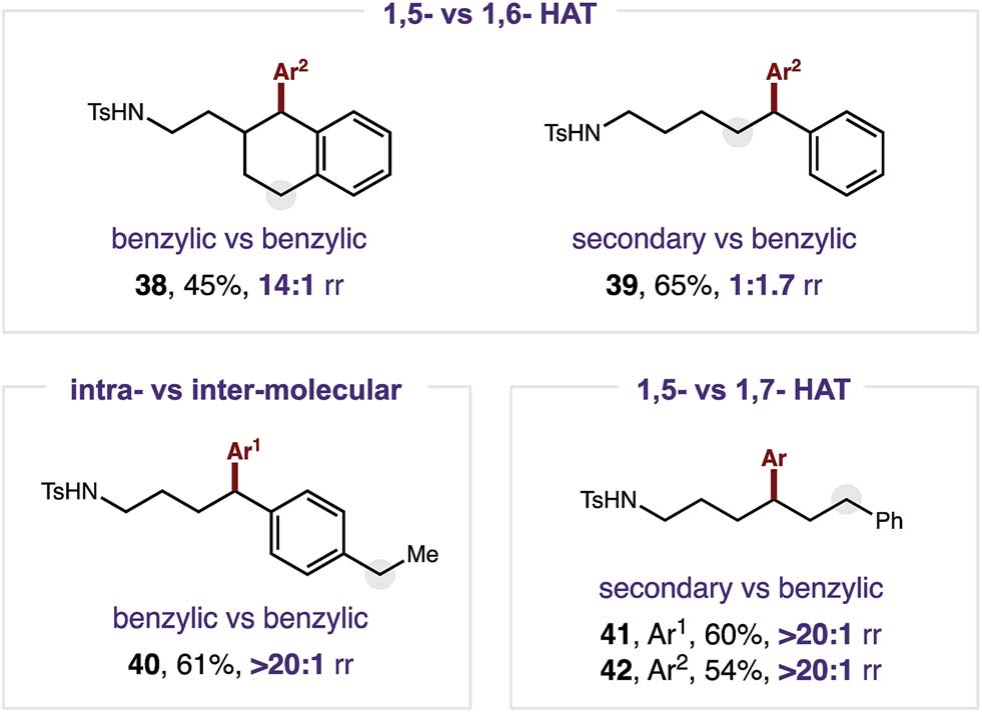
Regioselectivity probes of intramolecular HAT.

The robust δ regioselectivity observed in these reactions, including an arylation of a single 1-of-15 methylenes (**7**), led us to further investigate the limits of the selectivity in the key HAT step (Fig. 3). With this question in mind, we subjected an amide with two benzylic C–H bonds at δ and ε positions to these catalytic conditions. Selective δ arylation of this probe (**38**; 14 : 1) confirms 1,5-HAT is highly favored over 1,6-HAT in this reaction. However, this kinetic preference can be thermodynamically overridden if a weaker, benzylic ε C–H is pitted against a secondary δ C–H. In this case, 1,6-HAT is slightly favored (**39**; 1.7 : 1). Importantly, and as a complement to previous work on α C–H arylation,^10^–^12^ when a molecule containing a distal benzylic C–H bond is employed, no undirected arylation is observed (**40**; > 20 : 1 δ). Finally, in a three-way competition between δ/ε secondary C–H bonds and a ζ benzylic C–H bond, only the product of 1,5-HAT is observed, rather than either 1,6 or 1,7 HAT (**41–42**; > 20 : 1 δ).

**Fig. 4:**
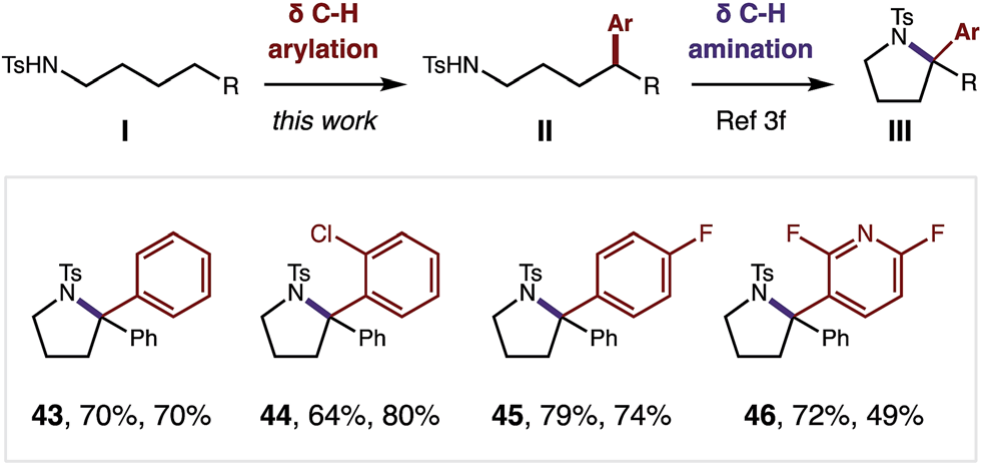
Pyrrolidine synthesis *via* iterative δ C–H arylation and δ C–H amination.

Although this δ C–H arylation was enabled by interrupting the classic Hofmann–Löffler–Freytag (HLF) reaction, we questioned if these complementary transformations could be sequentially combined (Fig. 4). For example, we have demonstrated that this Cu-catalyzed δ arylation (**I**) yields 4-aryl-butyl amides (**II**), and subsequent AcOI-mediated δ amination affords α diaryl pyrrolidines (**III**), bearing a tetra-substituted carbon (**43–46**). We expect this two-step, double C–H functionalization sequence will provide a synthetically enabling route to access medicinally relevant pyrrolidines,^17^ in a rapid, modular, and non-classical fashion.

Finally, given the strong dependence of reaction efficiency on ligand choice, as well as the ready availability of enantioenriched bisoxazoline ligands, we tested whether a catalytic, asymmetric version of this δ C–H arylation is possible (eqn (1)). To our delight, we found that employing enantiopure **L1**^18^ affords enantioenriched diaryl amide (**1**, 48% ee). Moreover, decreasing the temperature to –4 °C (and employing a co-solvent with a lower mp) allows access to this 1,1-diaryl-product with 65% ee – affording the first example of an enantioselective, radical C–H arylation.1
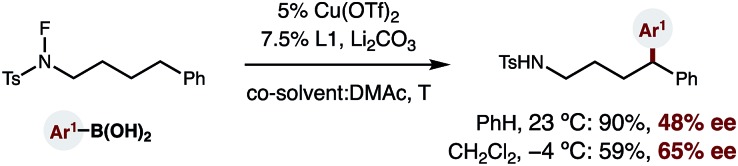



## Conclusions

In summary, we have interrupted the HLF reaction to afford a δ C–H arylation. The regioselectively translocated C˙ of this classic mechanism was intercepted and employed in C–C bond formation by using a Cu catalyst. The two key roles of this catalyst are: (i) to generate N˙ without also forming a δ halogenating X˙, and (ii) to trap the translocated C˙ as an organometallic intermediate. This organocopper species enables cross-coupling with a range of (hetero)aryl boronic acids at the δ position of sulfonamides in a regioselective (and stereoselective) manner. We expect this strategy will facilitate further development of methods to interrupt the classic HLF reaction with valuable C–C bond formation.

## Conflicts of interest

There are no conflicts to declare.

## Supplementary Material

Supplementary information
