# Supplementary material for: δ C–H (hetero)arylation via Cu-catalyzed radical relay
Source: Chem Sci. 2018 Nov 9;10(4):1207–11. doi: 10.1039/c8sc04366c (PMC6349054; doi:10.1039/c8sc04366c)
Supplement: Supplementary file 1 [file SC-010-C8SC04366C-s001.pdf]

Electronic Supplementary Material (ESI) for Chemical Science.
This journal is © The Royal Society of Chemistry 2018

Electronic Supplementary Information for:

δ C-H (Hetero)Arylation via Cu-Catalyzed Radical Relay

Zuxiao Zhang, Leah M. Stateman, David A. Nagib*

Department of Chemistry and Biochemistry, The Ohio State University,
151 W. Woodruff Ave., Columbus, OH, 43210, United States

Corresponding Author* Email: nagib.1@osu.edu

Table of Contents

I. General Information, S2
II. Optimization of conditions, S3
III. Experimental procedures and data, S8
IV. References, S32
V. NMR Spectra for new compounds, S33

I. General Information

All chemicals and reagents were purchased from Sigma-Aldrich, Alfa Aesar, Acros, TCI, or ChemImplex. Solvents were purified in the following manner. Acetonitrile and amine bases were distilled over calcium hydride. CH_2Cl_2, THF, Et_2O and DMF were degassed with N_2 and dried by passing through columns containing alumina, copper, or molecular sieves. Flash column chromatography, or preparative thin-layer chromatography, was performed with Silicycle F60 (230-400 mesh) silica gel. Thin layer chromatography (TLC) analyses were performed using EMD 60 F254 TLC plates and visualized by fluorescence quenching or $KMnO_4$ stain. All yields are averages of at least two experimental runs.

Nuclear magnetic resonance (NMR) spectra (^{1}H, ^{19}F, ^{13}C) were recorded using either a Bruker AVIII 400 or AVIII 600 MHz NMR spectrometer. ^{1}H and ^{13}C NMR chemical shifts are reported in parts per million and referenced to residual $CHCl_3$ signals in $CDCl_3$ (^{1}H: δ 7.26; ^{13}C: δ 77.16), or $CFCl_3$ (^{19}F: δ 0.0). ^{1}H NMR data are reported as follows: chemical shifts (δ ppm), multiplicity (s = singlet, d = doublet, t = triplet, q = quartet, m = multiplet, b = broad, ap = apparent), coupling constant (Hz), relative integral. Data for ^{13}C and ^{19}F NMR are reported in terms of chemical shift and multiplicity where appropriate. High-resolution Mass Spectrometry (HRMS) data were obtained using Bruker MicrOTOF (ESI). Infrared (IR) spectra were recorded using a Thermo Fisher Nicolet iS10 FT-IR and are reported in terms of frequency of absorption (cm^{-1}).

High Pressure Liquid chromatography (HPLC) was performed on a Hewlett-Packard 1100 Series chromatographs using a chiral column (25 cm) and guard column (5 cm) as noted for each compound. Optical rotations were measured on a Jasco P-1010 polarimeter with $[\alpha]_D$ values reported in degrees; concentration (c) is in g/100 mL.

II. Optimization of conditions

Table S1 Control experiments.

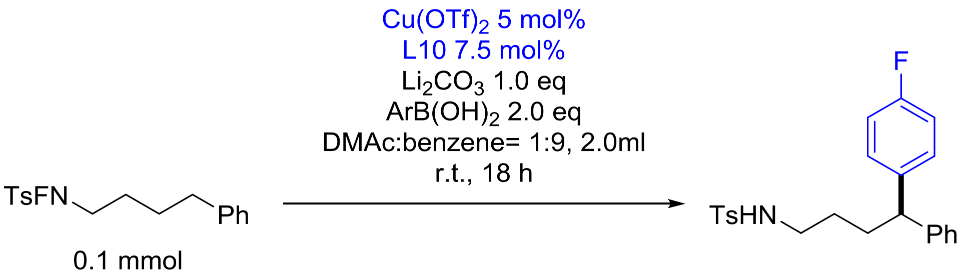

entry	conditions	result
1	standard	83% product
2	no Cu	SM only
3	no L	30% product, 80% SM
4	no base	56% product
5	no boronic acid	0% product, 100% SM
6	ArBpin instead of $ArB(OH)_2$	0% product, 100% SM

[a] Reactions were run with 0.1 mmol of 1a [b] all yields are NMR yield based on 1a using CH_2Br_2 as the internal standard.

Table S2 Optimization of copper catalysts.

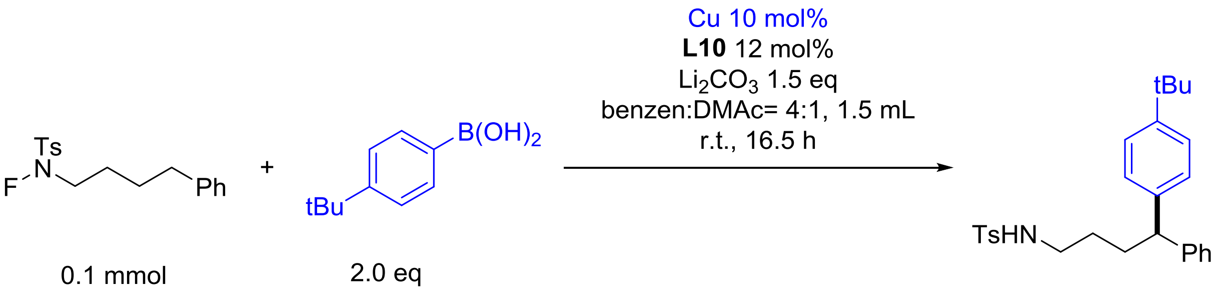

entry	catalyst	result
1	CuOAc	17% product
2	CuCl	31% product
3	CuOTf benzene	25% product
4	$Cu(CH_3CN)_4PF_6$	14% product, 31% SM
5	$Cu(OAc)_2$	5% product, 52% SM
**6**	**$Cu(OTf)_2$**	**62% product**

[a] Reactions were run with 0.1 mmol of 1a [b] all yields are NMR yield based on 1a using CH_2Br_2 as the internal standard.

Table S3 Optimization of ligands.

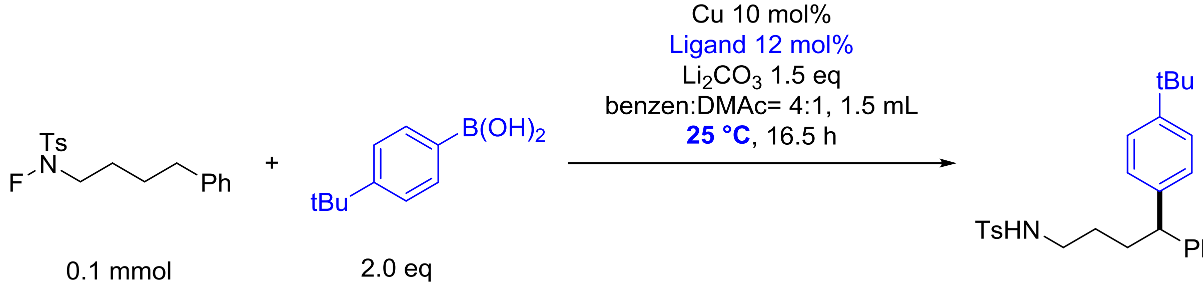

0.1 mmol   2.0 eq

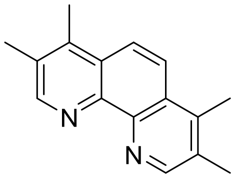

L10 r.t., 43%
35 °C, 50%
50 °C,42%

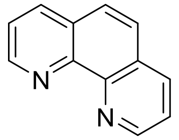

35 °C L2, 48%

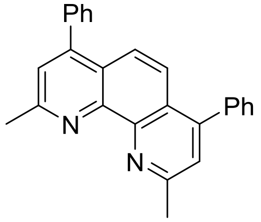

35 °C L3, 18%

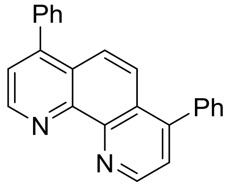

35 °C L4, 48%

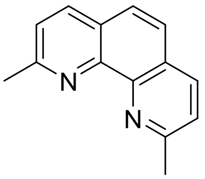

35 °C L5, 36%

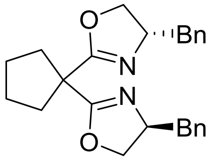

L6, 51.5%

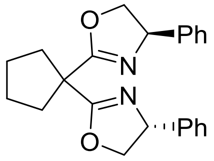

L7, 52%

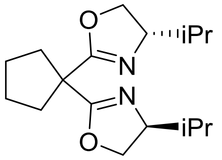

L8, 49%

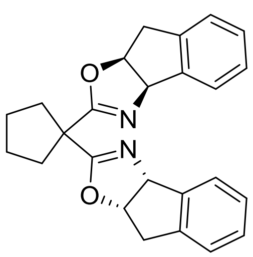

L9, 61%

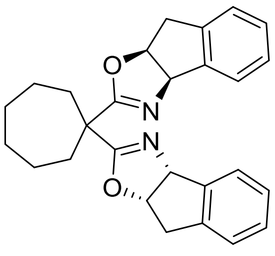

L1, 73%

[a] Reactions were run with 0.1 mmol of 1a [b] all yields are NMR yield based on 1a using CH_2Br_2 as the internal standard.

Table S4 Optimization of solvents.

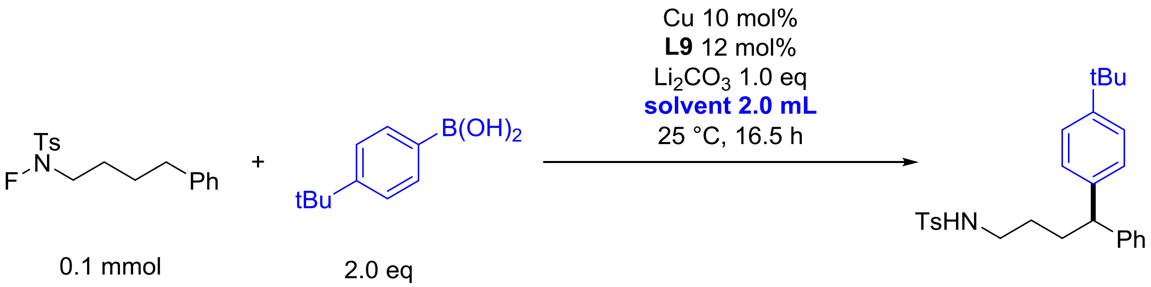

entry	conditions	result
1	DMF	42%
2	DMAc	46%
3	CH_3CN	0%
4	dioxane	32%
5	benzene	0%
6	DCM	0%
7	toluene	0%
8	THF	0%
9	DCM:DMAc = 4:1	63%
10	CH_3CN:DMAc = 4:1	16%
11	dioxane:DMAc = 4:1	55%
12	**toluene:DMAc = 4:1**	**65%**

[a] Reactions were run with 0.1 mmol of 1a [b] all yields are NMR yield based on 1a using CH_2Br_2 as the internal standard.

Table S5 Optimization of bases.

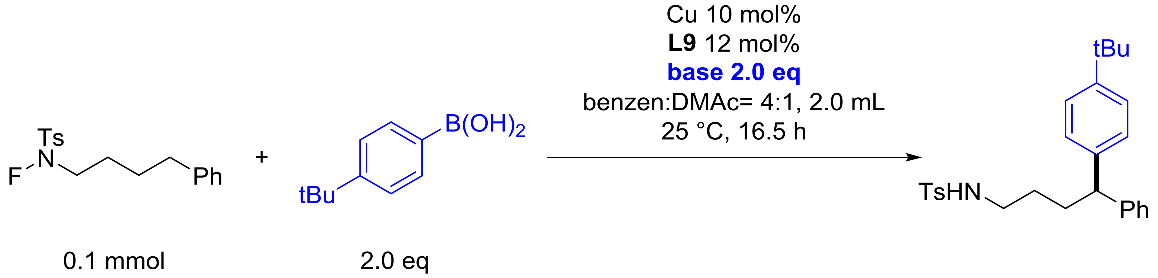

entry	conditions	result
1	no base	39%
2	0.5 eq, Li_2CO_3	52%
3	1.0 eq, Li_2CO_3	50%
4	1.5 eq, Li_2CO_3	51%
5	2.0 eq, Li_2CO_3	55%
6	2.0 eq, LiOtBu	30%
7	2.0 eq, NaOtBu	35%
8	2.0 eq, KOtBu	7%

[a] Reactions were run with 0.1 mmol of 1a [b] all yields are NMR yield based on 1a using CH_2Br_2 as the internal standard.

Table S6 Optimization of copper and ligand combination.

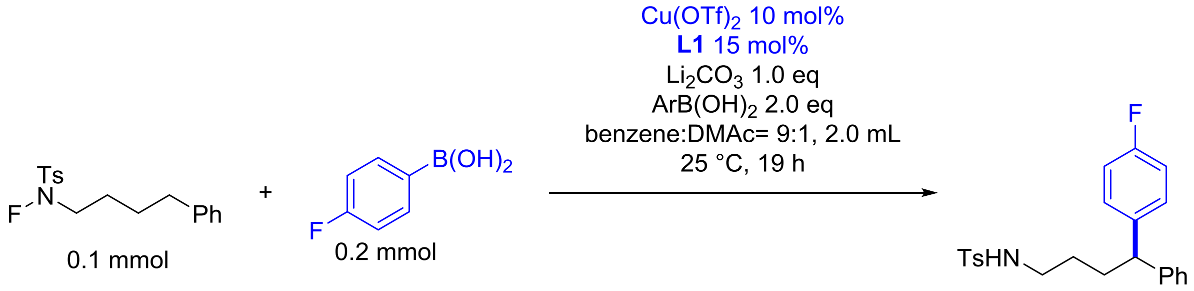

entry	conditions	result
1	standard	70%
2	Cu 5 %, L 5%	56%
3	Cu 5 %, L 6%	63%
4	Cu 5 %, L 7.5%	63%
5	Cu 5 %, L 10%	63%
6	Cu 5 %, L 10%, r.t.	70%
7	**Cu 5 %, L 7.5%, r.t.**	**83%**

[a] Reactions were run with 0.1 mmol of 1a [b] all yields are NMR yield based on 1a using CH_2Br_2 as the internal standard.

Table S7 Time study of enantioselectivity.

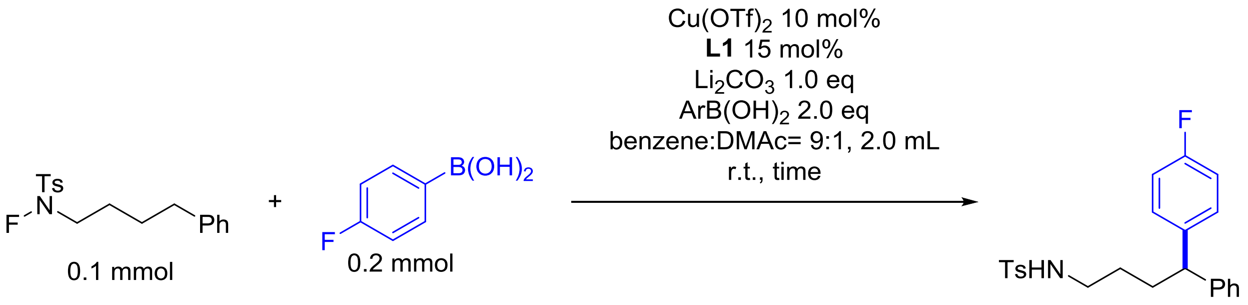

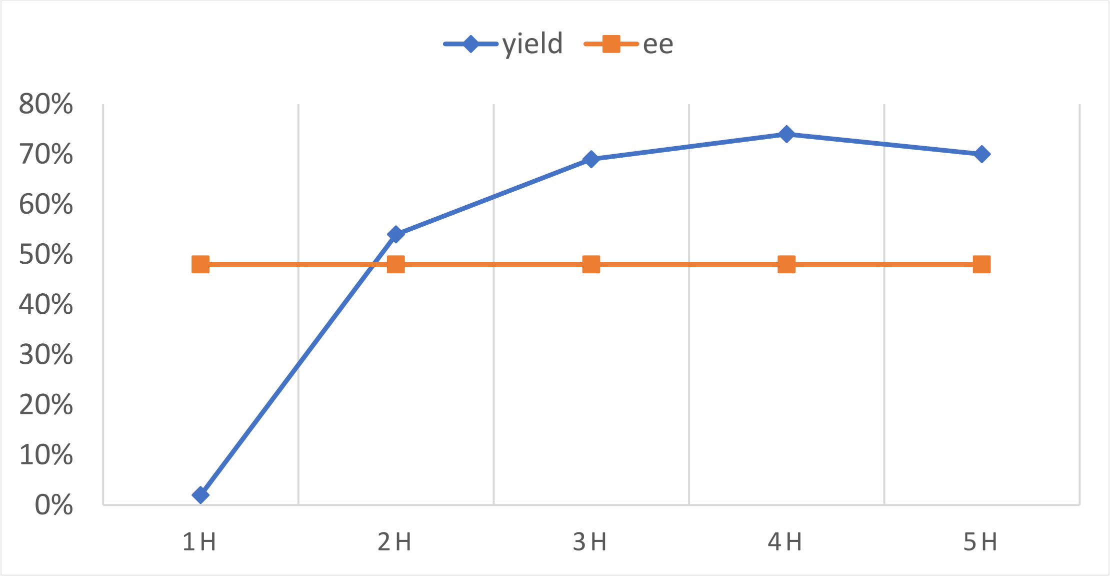

III. Experimental procedures and data

1. Synthesis of Starting Materials

General Procedure A − N-F sulfonamides

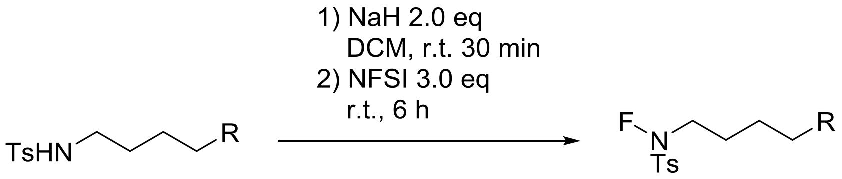

Synthesized according to a reported procedure[1] with slight modifications: In an oven dried round bottom flask, sulfonamide (10 mmol, 1.0 equiv) was dissolved in dry DCM (120 mL), followed by slow addition of NaH (20 mmol, 2.0 equiv). The mixture was allowed to stir for 30 min at room temperature under nitrogen. Then, NFSI (30 mmol, 3.0 equiv) was added portionwise to the mixture and the resulting slurry was stirred for another 6 hours. Upon completion, the reaction was quenched by the addition of water. The mixture was extracted with DCM (3 x 100 mL) and the organic layers were combined, washed with brine, and dried over anhydrous Na_2SO_4. The crude mixture was filtered through celite and concentrated. The resulting residue was purified by column chromatography on silica gel with a gradient eluent of petroleum ether and ethyl acetate.

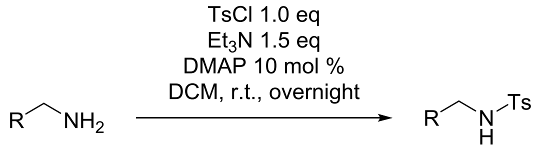

Synthesized according to a reported procedure.[2] To a clean, dry round bottom flask was added a magnetic stir bar and primary amine (1 equiv) under nitrogen at RT. The substrate was dissolved in DCM [0.2 M], followed by addition of freshly distilled triethylamine (1.5 equiv), 4-(Dimethylamino)pyridine (10 mol %) and *p*-toluenesulfonyl chloride (1 equiv) were subsequently added. The reaction was allowed to stir at room temperature overnight. H_2O was added to the reaction and the aqueous layer was extracted DCM (3 x 100 mL). The combined organic layers were washed with brine, dried over Na_2SO_4, and concentrated *in vacuo*. The crude material was purified by silica gel chromatography.

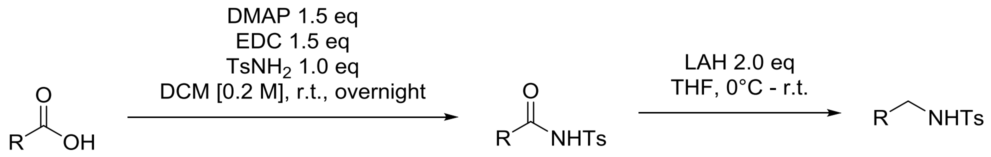

To a clean, dry round bottom flask was added a magnetic stir bar, the starting carboxylic acid (1.0 equiv), 4-(Dimethylamino)pyridine (1.5 equiv) and tosylamide (1.0 equiv) under nitrogen at room temperature. The mixture was dissolved in DCM, followed by addition of EDC (1.5 equiv). The reaction was allowed to stir at room temperature overnight. Upon completion, 4N HCl was added, the organic phase was collected, and the aqueous layer was extracted three times with DCM. The combined organic phase was washed with brine, dried over Na_2SO_4, and concentrated *in vacuo*. The crude mixture was then taken onto the reduction step.

To a dry round bottom flask, was added a magnetic stir bar, the starting amide (1.0 equiv), and lithium aluminum hydride (2.0 equiv) under nitrogen. Reaction was cooled to 0 °C and slowly dissolved in THF. The reaction was monitored by TLC and upon consumption of starting material, the mixture was cooled to 0 °C and quenched carefully by addition of a 1 M solution of sodium hydroxide. The reaction was allowed to warm to room temperature and stirred for 20 minutes. The mixture was filtered through celite and the resulting clear solution was dried over Na_2SO_4 and concentrated *in vacuo*. Final substrates were purified by silica gel chromatography.

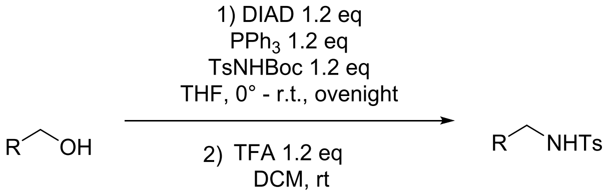

To a dry round bottom flask, was added a magnetic stir bar, TsNHBoc (1.2 equiv), and triphenylphosphine (1.2 equiv). The solids were dissolved in THF under nitrogen and alcohol (1.0 equiv) was added to the solution. The reaction was cooled to 0 °C, followed by slow addition of DIAD (1.2 equiv) and the mixture was allowed to gradually warm to room temperature and stir overnight. Water was added to the crude reaction and then extracted 3x with Et_2O. The combined organic layers were washed with brine, dried over Na_2SO_4, and concentrated *in vacuo*. Purification by silica gel chromatography provided the N-boc protected amine. To a solution of N-boc protected amine in DCM, was added TFA at room temperature. The reaction was stirred overnight. The reaction was diluted with DCM and slowly quenched with a saturated solution of $NaHCO_3$.

The combined organic layers were washed with brine, dried over Na_2SO_4, and concentrated *in vacuo*. Column chromatography on silica gel provided the pure product.

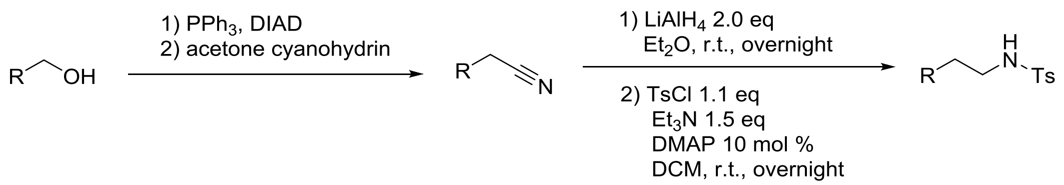

A 100 mL flask was charged with alcohol and dry toluene. DIAD (2 equiv), triphenylphosphine (2 equiv), and acetone cyanohydrin (4 equiv) were added to the solution. The reaction was stirred for 12 h at room temperature and then quenched with H_2O. The aqueous layer was extracted with EtOAc (3 × 30 mL) and the combined organic layers were dried over Na_2SO_4, and concentrated *in vacuo* to furnish a colorless oil. The resulting residue was purified by silica gel chromatography to provide the target nitrile.

To a dry round bottom flask, was added a magnetic stir bar and the starting nitrile (1.0 equiv). Et_2O was added, the solution was cooled to 0 °C, and then lithium aluminum hydride (2.0 equiv) was added portionwise. After stirring overnight, the mixture was cooled to 0 °C and quenched carefully by addition of a 1 M solution of sodium hydroxide. The reaction was allowed to warm to room temperature and stirred for 20 minutes. The mixture was filtered through celite, the resulting clear solution was dried over Na_2SO_4, and concentrated *in vacuo*. Final substrates were purified by silica gel chromatography.

2. Synthesis of Products

General Procedure B − Racemic remote C-H arylation

In an oven dried vial with a septa cap, racemic **L1** (0.015 mmol, 7.5 mol %) and $Cu(OTf)_2$ (0.010 mmol, 5 mol %) were dissolved in a premade solvent mixture (benzene: dimethylacetamide = 9:1, 2.0 mL) under a nitrogen atmosphere, and the mixture was stirred for 30 minutes at room temperature. In a separate oven dried vial, substrate **1a** (0.2 mmol, 1.0 equiv) and arylboronic acid (0.4 mmol, 2.0 equiv) were added, then brought into the glove box where Li_2CO_3 (0.2 mmol, 1.0 equiv) was added. The vial was sealed and removed from the glove box. To this vial, was added

the copper solution via syringe under a nitrogen atmosphere, and then sealed with parafilm. The reaction mixture was stirred at room temperature for 14-30 hours. Then, the reaction was diluted with ethyl acetate (20 mL), then sequentially washed with water (15 mL) and brine (15 mL). The organic layer was dried with Na_2SO_4 for 2h and concentrated *in vacuo*. The residue was purified by column chromatography on silica gel with the indicated solvent combination to afford the desired product.

Note: The Li_2CO_3 was ground and dried by high vacuum overnight, then stored in the glove box.

General Procedure C − Enantioselective remote C-H arylation

In an oven dried vial with a septa cap, enantiopure **L1** (0.015 mmol, 7.5 mol %) and $Cu(OTf)_2$ (0.010 mmol, 5 mol %) were dissolved in a premade solvent mixture (DCM: dimethylacetamide = 9:1, 2.0 mL) under a nitrogen atmosphere, and the mixture was stirred for 30 minutes at room temperature. In a separate oven dried vial, substrate **1a** (0.2 mmol, 1.0 equiv) and arylboronic acid (0.4 mmol, 2.0 equiv) were added, then brought into the glove box where Li_2CO_3 (0.2 mmol, 1.0 eq) was added. The vial was sealed and removed from the glove box. To this vial, was added the copper solution via syringe under a nitrogen atmosphere, and then sealed with parafilm. The reaction mixture was stirred at -4 ℃ for 24 h. The desired product was purified by column chromatography on silica gel with a gradient eluent of hexane, dichloromethane and ether.

N-F sulfonamides:

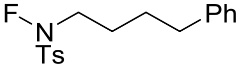

N-fluoro-4-methyl-N-(4-phenylbutyl)benzenesulfonamide (S1)

Prepared following general procedure A the reaction mixture was purified by column chromatography using 7% EtOAc in hexane to provide the title compound in 60% yield as a white solid. ^{1}H NMR (600 MHz, $CDCl_3$) δ 7.82 (d, *J* = 8.3 Hz, 2H), 7.40 (d, *J* = 8.0 Hz, 2H), 7.28 (t, *J* = 7.5 Hz, 2H), 7.19 (d, *J* = 7.4 Hz, 1H), 7.18 – 7.15 (m, 2H), 3.23 (dt, *J* = 40.8, 6.2 Hz, 2H), 2.64 (t, *J* = 7.0 Hz, 2H), 2.48 (s, 3H), 1.79 – 1.72 (m, 4H). ^{19}F NMR (565 MHz, $CDCl_3$) δ -49.67 (t, *J* = 40.7 Hz). ^{13}C NMR (151 MHz, $CDCl_3$) δ 146.34 (s), 141.84 (s), 130.06 (s), 130.05 (s), 129.03 (s), 128.48 (s), 126.01 (s), 53.64 (d, *J* = 12.4 Hz), 35.41 (s), 28.41 (s), 25.99 (s), 21.88 (s). IR (cm^-): 3335, 3259, 3062, 3026, 2861, 2829, 2359, 2342, 1597, 1527, 1496, 1452, 1378, 1299, 1157, 1129, 1096, 1066, 903. HRMS (ESI) calcd. For $[M+Na]^+$ 344.1091, found: 344.1091.
R_f = 0.6 (15% EtOAc in hexane).

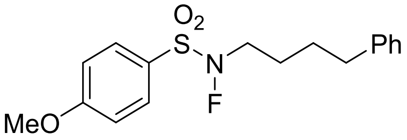

N-fluoro-4-methoxy-N-(4-phenylbutyl)benzenesulfonamide (S2)

Prepared following general procedure A the reaction mixture was purified by column chromatography using 10% EtOAc in hexane to provide the title compound in 60% yield as a white solid. ^{1}H NMR (600 MHz, $CDCl_3$) δ 7.88 – 7.82 (m, 2H), 7.30 – 7.24 (m, 2H), 7.20 – 7.17 (m, 1H), 7.18 – 7.14 (m, 2H), 7.07 – 7.04 (m, 2H), 3.91 (s, 3H), 3.23 (dt, *J* = 40.9, 6.3 Hz, 2H), 2.64 (t, *J* = 7.0 Hz, 2H), 1.75 (dt, *J* = 7.0, 3.4 Hz, 4H). ^{19}F NMR (565 MHz, $CDCl_3$) δ -49.43 (t, *J* = 40.9 Hz). ^{13}C NMR (151 MHz, $CDCl_3$) δ 164.89 (s), 141.88 (s), 132.35 (s), 128.51 (s), 128.50 (s), 126.03 (s), 123.27 (s), 114.68 (s), 55.93 (s), 53.63 (d, *J* = 12.3 Hz), 35.45 (s), 28.46 (s), 26.02 (s). IR (cm^-): 3265, 2360, 2392, 1636, 1592, 1495, 1331, 1292, 1236, 1167, 1153, 1123, 1093.
HRMS (ESI) calcd. For $[M+Na]^+$ 360.1040, found:360.1036 .
R_f = 0.5 (15% EtOAc in hexane).

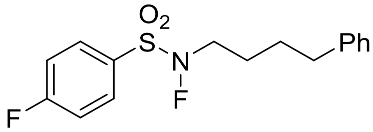

N,4-difluoro-N-(4-phenylbutyl)benzenesulfonamide (S3)

Prepared following general procedure A the reaction mixture was purified by column chromatography using 7% EtOAc in hexane to provide the title compound in 60% yield as a white solid. ^{1}H NMR (600 MHz, $CDCl_3$) δ 7.99 – 7.94 (m, 2H), 7.32 – 7.25 (m, 4H), 7.21 – 7.17 (m, 1H), 7.16 (dd, *J* = 7.8, 0.9 Hz, 2H), 3.26 (dt, *J* = 40.5, 6.5 Hz, 2H), 2.65 (t, *J* = 7.0 Hz, 2H), 1.80 – 1.72 (m, 4H). ^{19}F NMR (565 MHz, $CDCl_3$) δ -49.21 – -49.45 (m), -101.01 (dq, *J* = 8.6, 5.2 Hz). ^{13}C NMR (151 MHz, $CDCl_3$) δ 166.74 (d, *J* = 258.6 Hz), 141.78 (s), 132.98 (d, *J* = 9.8 Hz), 128.54 (s), 128.51 (s), 128.33 (d, *J* = 3.1 Hz), 126.09 (s), 116.90 (d, *J* = 22.9 Hz), 53.45 (d, *J* = 12.4 Hz), 35.43 (s), 28.41 (s), 25.97 (s). IR (cm^-): 3258, 3025, 2945, 2839, 2359, 2342, 1595, 1577, 1497, 1457, 1370, 1335, 1303, 1260, 1188, 1160, 1125, 1093, 1025.
HRMS (ESI) calcd. For $[M+Na]^+$ 348.0840, found:348.0831 .
R_f = 0.6 (15% EtOAc in hexane).

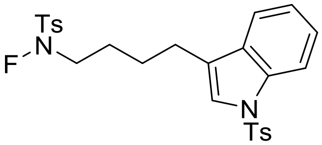

N-fluoro-4-methyl-N-(4-(1-tosyl-1H-indol-3-yl)butyl)benzenesulfonamide (S4)

Prepared following general procedure A using substrate (400 mg, 0.81 mmol). After 6 hours, the reaction mixture was purified by column chromatography using 30% hexanes in DCM then recrystallized with DCM-hexanes to give the title compound **(**18.2 mg, 0.035 mmol 4.3%). ^{1}H NMR (600 MHz, $CDCl_3$) δ 7.97 (d, *J* = 8.3 Hz, 1H), 7.82 (d, *J* = 8.3 Hz, 2H), 7.72 (d, *J* = 8.4 Hz, 2H), 7.44 (d, *J* = 7.9 Hz, 1H), 7.41 (d, *J* = 8.1 Hz, 2H), 7.32 – 7.27 (m, 2H), 7.24 – 7.17 (m, 3H), 3.26 (t, *J* = 40.5, 6.6 Hz, 2H), 2.68 (t, *J* = 7.0 Hz, 2H), 2.48 (s, 3H), 2.32 (s, 3H), 1.83 – 1.71 (m, 4H). ^{19}F NMR (565 MHz, $CDCl_3$) δ -49.65 (t, *J* = 40.5 Hz). ^{13}C NMR (151 MHz, $CDCl_3$) δ 146.45 (s), 144.89 (s), 135.56 (s), 135.42 (s), 131.03 (s), 130.12 (s), 130.11 (s), 129.96 (s), 128.98 (s), 126.87 (s), 124.82 (s), 123.17 (s), 122.94 (s), 122.69 (s), 119.53 (s), 113.99 (s), 53.52 (d, *J* = 12.3 Hz), 26.07 (s), 26.00 (s), 24.48 (s), 21.94 (s), 21.68 (s). IR (flim) cm^{-1} 3046, 2987, 2945, 1373, 1036.
HRMS (ESI-TOF) *m/z* calcd. for $[M+Na]^+$ 537.1294, found 537.1289.
R_f = 0.5 (30% hexanes in DCM)

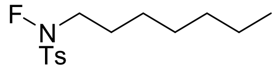

N-fluoro-N-heptyl-4-methylbenzenesulfonamide (S5)

Prepared following general procedure A the reaction mixture was purified by column chromatography using 7% EtOAc in hexane to provide the title compound in 80% yield as a light yellow solid. ^{1}H NMR (600 MHz, $CDCl_3$) δ 7.82 (d, *J* = 8.3 Hz, 2H), 7.40 (dd, *J* = 8.5, 0.5 Hz, 2H), 3.20 (dt, *J* = 40.7, 7.0 Hz, 2H), 2.48 (s, 3H), 1.70 (dt, *J* = 14.9, 7.5 Hz, 2H), 1.43 – 1.34 (m, 2H), 1.34 – 1.21 (m, 6H), 0.87 (t, *J* = 7.0 Hz, 3H). ^{19}F NMR (565 MHz, $CDCl_3$) δ -49.90 (t, *J* = 40.7 Hz). ^{13}C NMR (151 MHz, $CDCl_3$) δ 146.28 (s), 130.11 (s), 130.05 (s), 129.19 (s), 53.86 (d, *J* = 12.4 Hz), 31.75 (s), 28.91 (s), 26.69 (s), 26.45 (s), 22.66 (s), 21.92 (s), 14.16 (s). IR (cm^{-}): 2927, 2857, 2360, 2342, 1596, 1457, 1373, 1188, 1171, 1089.
HRMS (ESI) calcd. For $[M+Na]^+$ 310.1247, found: 310.1239.
R_f = 0.6 (15% EtOAc in hexane).

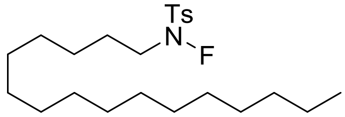

N-fluoro-N-hexadecyl-4-methylbenzenesulfonamide (S7)

Prepared following general procedure A the reaction mixture was purified by column chromatography using 5% EtOAc in hexane to provide the title compound in 65% yield as a white powder. ^{1}H NMR (400 MHz, $CDCl_3$) δ 7.83 (d, *J* = 8.3 Hz, 2H), 7.40 (d, *J* = 8.0 Hz, 2H), 3.20 (dt, *J* = 40.7, 7.0 Hz, 2H), 2.48 (s, 3H), 1.70 (dt, *J* = 14.8, 7.5 Hz, 2H), 1.44 – 1.34 (m, 2H), 1.33 – 1.19 (m, 23H), 0.88 (t, *J* = 6.9 Hz, 3H). ^{19}F NMR (377 MHz, $CDCl_3$) δ -49.88 (t, *J* = 40.7 Hz). ^{13}C NMR (101 MHz, $CDCl_3$) δ 146.27 (s), 130.09 (s), 130.04 (s), 129.15 (s), 53.87 (d, *J* = 12.4 Hz), 32.06 (s), 29.82 (s), 29.79 (s), 29.75 (s), 29.65 (s), 29.55 (s), 29.49 (s), 29.25 (s), 26.72 (s), 26.45 (s), 22.82 (s), 21.89 (s), 14.24 (s).
Rf = 0.7 (hexane:EA= 10:1)

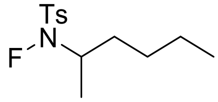

N-fluoro-N-(hexan-2-yl)-4-methylbenzenesulfonamide (S8)

Prepared following general procedure A the reaction mixture was purified by column chromatography using 7% EtOAc in hexane to provide the title compound in 60% yield as a light-yellow oil. ^{1}H NMR (600 MHz, $CDCl_3$) δ 7.84 (d, *J* = 8.3 Hz, 2H), 7.37 (d, *J* = 8.2 Hz, 2H), 4.01 (ddd, *J* = 37.3, 13.4, 6.7 Hz, 1H), 2.46 (s, 3H), 1.75 (tdd, *J* = 9.7, 8.0, 6.4 Hz, 1H), 1.57 – 1.49 (m, 1H), 1.43 – 1.27 (m, 4H), 1.23 (dd, *J* = 6.5, 1.2 Hz, 3H), 0.92 – 0.87 (m, 3H). ^{19}F NMR (565 MHz, $CDCl_3$) δ -80.34 (d, *J* = 37.3 Hz). ^{13}C NMR (151 MHz, $CDCl_3$) δ 145.79 (s), 132.94 (s), 129.96 (s), 129.36 (s), 59.27 (d, *J* = 13.1 Hz), 34.10 (d, *J* = 1.7 Hz), 28.29 (s), 22.48 (s), 21.87 (s), 15.96 (d, *J* = 11.3 Hz), 14.07 (s). IR (cm^{-}): 2956, 2931, 2862, 2361, 1399, 1596, 1457, 1359, 1187, 1169, 1089, 992.

HRMS (ESI) calcd. For $[M+Na]^+$ 296.1091, found:296.1084.
R_f = 0.6 (15% EtOAc in hexane).

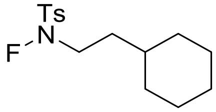

N-(2-cyclohexylethyl)-N-fluoro-4-methylbenzenesulfonamide (S9)

Prepared following general procedure A using substrate (593 mg. 2.1 mmol). After 6 hours, the reaction mixture was purified by column chromatography using 5% ethyl acetate in hexanes to give the title compound as a colorless solid (251 mg, 0.83 mmol 40%). ^1H NMR (600 MHz, $CDCl_3$) δ 7.83 (d, *J* = 8.3 Hz, 2H), 7.40 (d, *J* = 8.0 Hz, 2H), 3.24 (dt, *J* = 40.4, 7.2 2H), 2.48 (s, 3H), 1.74 – 1.65 (m, 4H), 1.60 (dd, *J* = 14.4, 6.9 Hz, 2H), 1.43 – 1.36 (m, 1H), 1.29 – 1.08 (m, 4H), 0.98 – 0.83 (m, 3H).^{19}F NMR (565 MHz, $CDCl_3$) δ -50.35 (t, *J* = 41.0 Hz). ^{13}C NMR (151 MHz, $CDCl_3$) δ 146.28 (s), 130.12 (s), 130.07 (s), 129.18 (s), 51.80 (d, *J* = 12.4 Hz), 35.11 (s), 33.64 (s), 33.13 (s), 26.56 (s), 26.26 (s), 21.93 (s). IR (flim) cm^{-1} 2936, 2920, 2875, 1599, 1355, 1174.
HRMS (ESI-TOF) *m/z* calcd for $[M+Na]^+$ 322.1253, found 322.1244.
R_f = 0.3 (5% EtOAc in hexanes)

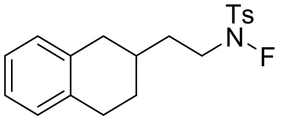

N-fluoro-4-methyl-N-(2-(1,2,3,4-tetrahydronaphthalen-2-yl)ethyl)benzenesulfonamide (S38)

Prepared following general procedure A the reaction mixture was purified by column chromatography using 7% EtOAc in hexane to provide the title compound in 55% yield as a light yellow oil. ^1H NMR (600 MHz, $CDCl_3$) δ 7.85 (d, *J* = 8.3 Hz, 2H), 7.42 (d, *J* = 8.1 Hz, 2H), 7.11 – 7.03 (m, 4H), 3.37 (dt, *J* = 40.3, 7.1 Hz, 2H), 2.87 (dd, *J* = 16.2, 5.0 Hz, 1H), 2.83 – 2.77 (m, 2H), 2.49 (s, 3H), 2.45 (dd, *J* = 16.2, 10.4 Hz, 1H), 1.98 – 1.88 (m, 2H), 1.86 – 1.72 (m, 2H), 1.49 – 1.38 (m, 1H). ^{19}F NMR (565 MHz, $CDCl_3$) δ -50.02 (t, *J* = 40.3 Hz). ^{13}C NMR (151 MHz, $CDCl_3$) δ 146.26 (s), 136.44 (s), 135.84 (s), 129.97 (s), 129.12 (s), 129.07 (s), 128.86 (s), 125.71 (s), 125.61 (s), 51.48 (d, *J* = 12.4 Hz), 35.72 (s), 32.58 (s), 31.65 (s), 29.13 (s), 28.82 (s), 21.80 (s). IR (cm^-): 3060, 3016, 2921, 2843, 1595, 1494, 1452, 1435, 1373, 1188, 1171, 1088. HRMS (ESI) calcd. For $[M+Na]^+$ 370.1247, found: 370.1238 .
R_f = 0.6 (15% EtOAc in hexane).

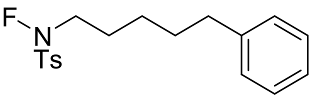

N-fluoro-4-methyl-N-(5-phenylpentyl)benzenesulfonamide (S39)

Prepared following general procedure A the reaction mixture was purified by column chromatography using 7% EtOAc in hexane to provide the title compound in 65% yield as a white solid. ^1H NMR (600 MHz, $CDCl_3$) δ 7.82 (d, *J* = 8.2 Hz, 2H), 7.40 (d, *J* = 8.0 Hz, 2H), 7.30 – 7.25 (m, 2H), 7.17 (dd, *J* = 16.0, 7.2 Hz, 3H), 3.20 (dt, *J* = 40.6, 7.0 Hz, 2H), 2.61 (dd, *J* = 14.9, 7.3 Hz, 2H), 2.48 (s, 3H), 1.74 (dt, *J* = 14.7, 7.3 Hz, 2H), 1.65 (dt, *J* = 15.5, 7.7 Hz, 2H), 1.44 (dt, *J* = 15.3, 7.7 Hz, 2H). ^{19}F NMR (565 MHz, $CDCl_3$) δ -49.79 (t, *J* = 40.6 Hz). ^{13}C NMR (151 MHz, $CDCl_3$) δ 146.32 (s), 142.38 (s), 130.10 (s), 130.07 (s), 129.13 (s), 128.51 (s), 128.45 (s), 125.89 (s), 53.73 (d, *J* = 12.4 Hz), 35.82 (s), 31.04 (s), 26.35 (s), 21.92 (s). IR (cm^-): 3061, 3025, 2929, 2858, 1595, 1494, 1452, 1399, 1372, 1306, 1295, 1188, 1171, 1089, 1018.
HRMS (ESI) calcd. For $[M+Na]^+$ 358.1247, found: 358.1243.
R_f = 0.6 (15% EtOAc in hexane).

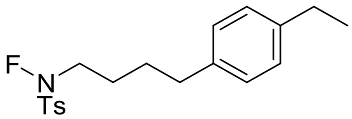

N-(4-(4-ethylphenyl)butyl)-N-fluoro-4-methylbenzenesulfonamide (S40)

Prepared following general procedure A the reaction mixture was purified by column chromatography using 7% EtOAc in hexane to provide the title compound in 40% yield as a colorless oil. ^{1}H NMR (600 MHz, $CDCl_3$) δ 7.81 (d, J = 8.3 Hz, 2H), 7.40 (d, J = 8.0 Hz, 2H), 7.11 (d, J = 8.1 Hz, 2H), 7.07 (d, J = 8.1 Hz, 2H), 3.22 (dt, J = 40.7, 6.5 Hz, 2H), 2.65 – 2.57 (m, 4H), 2.48 (s, 3H), 1.79 – 1.68 (m, 4H), 1.22 (t, J = 7.6 Hz, 3H). ^{19}F NMR (565 MHz, $CDCl_3$) δ -49.68 (t, J = 40.7 Hz). ^{13}C NMR (151 MHz, $CDCl_3$) δ 146.32 (s), 141.93 (s), 139.03 (s), 130.11 (s), 130.06 (s), 129.15 (s), 128.44 (s), 127.99 (s), 53.65 (d, J = 12.3 Hz), 35.01 (s), 28.57 (s), 28.53 (s), 26.04 (s), 21.92 (s), 15.77 (s). IR (cm^{-}): 2962, 2359, 2393, 1374, 1188, 1174, 1090, 819.
HRMS (ESI) calcd. For $[M+Na]^+$ 372.1404, found: 372.1392.
R_f = 0.6 (15% EtOAc in hexane).

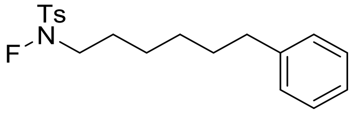

N-fluoro-4-methyl-N-(6-phenylhexyl)benzenesulfonamide (S41)

Prepared following general procedure A using substrate (565 mg, 3.5). After 6 hours, the reaction mixture was purified by column chromatography using 10% ethyl acetate in hexanes to give the title compound as a colorless solid (320 mg, 52%) ^{1}H NMR (600 MHz, $CDCl_3$) δ 7.82 (d, J = 8.3 Hz, 2H), 7.40 (d, J = 8.0 Hz, 2H), 7.30 – 7.26 (m, 2H), 7.20 – 7.13 (m, 3H), 3.20 (dt, J = 40.6, 7.0 Hz, 2H), 2.62 – 2.58 (m, 2H), 2.48 (s, 3H), 1.76 – 1.66 (m, 2H), 1.66 – 1.57 (m, 2H), 1.47 – 1.40 (m, 2H), 1.40 – 1.32 (m, 2H).^{19}F NMR (565 MHz, $CDCl_3$) δ -49.87 (t, J = 40.6 Hz). ^{13}C NMR (151 MHz, $CDCl_3$) δ 146.30 (s), 142.69 (s), 130.11 (s), 130.06 (s), 129.15 (s), 128.52 (s), 128.41 (s), 125.80 (s), 53.79 (d, J = 12.4 Hz), 35.91 (s), 31.34 (s), 28.86 (s), 26.58 (s), 26.37 (s), 21.92 (s). IR (flim) cm^{-1}3056, 3032, 2940, 2859, 1602, 1380, 1181.
HRMS (ESI-TOF) *m/z* calc'd for $[M+Na]^+$ 372.1409 found 372.1391.
R_f = 0.2 (5% EtOAc in hexanes)

Arylation product:

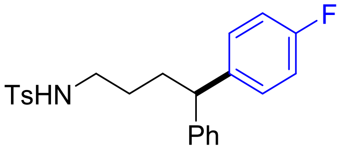

N-(4-(4-fluorophenyl)-4-phenylbutyl)-4-methylbenzenesulfonamide (1)

Prepared following general procedure B using substrate (64.2 mg, 0.2 mmol). After 16 hours, the reaction mixture was purified by column chromatography using 5% hexane in DCM to provide the title compound (66.3 mg, 79% yield) as a colorless oil. ^{1}H NMR (400 MHz, $CDCl_3$) δ 7.73 – 7.67 (m, 2H), 7.30 – 7.21 (m, 4H), 7.21 – 7.06 (m, 5H), 6.97 – 6.88 (m, 2H), 4.46 (t, *J* = 6.2 Hz, 1H), 3.79 (t, *J* = 7.9 Hz, 1H), 2.94 (q, *J* = 6.8 Hz, 2H), 2.42 (s, 3H), 2.02 – 1.92 (m, 2H), 1.45 – 1.35 (m, 2H). ^{19}F NMR (377 MHz, $CDCl_3$) δ -116.97 (tt, *J* = 8.7, 5.4 Hz). ^{13}C NMR (151 MHz, $CDCl_3$) δ 161.47 (d, *J* = 244.4 Hz), 144.36 (s), 143.52 (s), 140.34 (d, *J* = 3.2 Hz), 137.07 (s), 129.83 (s), 129.22 (d, *J* = 7.9 Hz), 128.70 (s), 127.76 (s), 127.19 (s), 126.52 (s), 115.36 (d, *J* = 21.2 Hz), 50.11 (s), 43.19 (s), 32.71 (s), 28.16 (s), 21.64 (s). IR (cm^{-}): 3276, 3027, 2926, 2866, 2360, 2341, 1599, 1507, 1493, 1451, 1416, 1321, 1304, 1220, 1154, 1093, 964.
HRMS (ESI) calcd. For $[M+Na]^+$ 420.1404, found: 420.1411.
R_f = 0.45 (DCM).
Prepared following general procedure C using substrate (64.2 mg, 0.2 mmol). After 24 hours, the reaction mixture was monitored by NMR and HPLC to get the yield and *ee* value.
HPLC (ADH, 0.46*25 cm, 5μm, hexane / isopropanol = 90/10, flow 0.7 mL/min, detection at 220 nm) retention time = 34.18min (minor) and 37.59min (major).

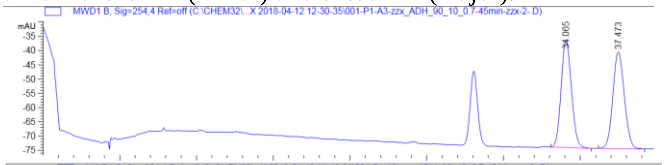

Signal 1: MWD1 B, Sig=254,4 Ref=off

Peak #	RetTime [min]	Type	Width [min]	Area [mAU*s]	Height [mAU]	Area %
1	34.065	BB	0.7219	1760.72192	37.63029	50.0600
2	37.473	BB	0.8005	1756.50122	33.90126	49.9400

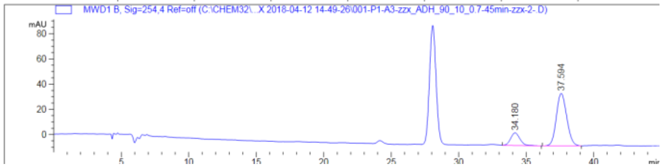

Signal 1: MWD1 B, Sig=254,4 Ref=off

Peak #	RetTime [min]	Type	Width [min]	Area [mAU*s]	Height [mAU]	Area %
1	34.180	BB	0.7046	470.97778	9.94300	17.5429
2	37.594	BB	0.8272	2213.74634	41.57830	82.4571

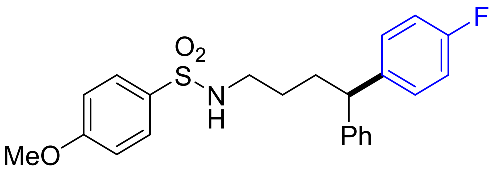

N-(4-(4-fluorophenyl)-4-phenylbutyl)-4-methoxybenzenesulfonamide (2)

Prepared following general procedure B using substrate (67.4 mg, 0.2 mmol). After 16 hours, the reaction mixture was purified by column chromatography using 5% hexane in DCM to provide the title compound (64.4 mg, 78% yield) as a colorless oil. ^{1}H NMR (600 MHz, $CDCl_3$) δ 7.78 – 7.73 (m, 2H), 7.28 – 7.23 (m, 3H), 7.19 – 7.15 (m, 1H), 7.15 – 7.08 (m, 4H), 6.97 – 6.91 (m, 4H), 4.48 – 4.37 (m, 1H), 3.86 (s, 3H), 3.79 (t, *J* = 7.9 Hz, 1H), 2.94 (q, *J* = 6.8 Hz, 2H), 2.02 – 1.91 (m, 2H), 1.46 – 1.34 (m, 2H). ^{19}F NMR (565 MHz, $CDCl_3$) δ -116.97 (dq, *J* = 8.5, 5.3 Hz). ^{13}C NMR (151 MHz, $CDCl_3$) δ 163.00 (s), 161.47 (d, *J* = 244.5 Hz), 144.36 (s), 140.33 (d, *J* = 3.2 Hz), 131.65 (s), 129.30 (s), 129.22 (d, *J* = 7.8 Hz), 128.71 (s), 127.76 (s), 126.53 (s), 115.37 (d, *J* = 21.2 Hz), 114.37 (s), 55.73 (s), 50.12 (s), 43.17 (s), 32.74 (s), 28.16 (s). R_f = 0.35 (DCM). IR (cm^{-}): 3280, 2942, 2360, 2341, 1596, 1578, 1507, 1497, 1322, 1300, 1259, 1220, 1152, 1095, 1025.

HRMS (ESI) calcd. For $[M+Na]^+$ 436.1353, found: 436.1345.
R_f = 0.40 (DCM).

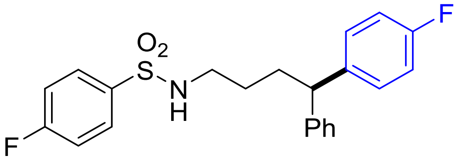

4-fluoro-N-(4-(4-fluorophenyl)-4-phenylbutyl)benzenesulfonamide (3)

Prepared following general procedure B using substrate (65.0 mg, 0.2 mmol). After 16 hours, the reaction mixture was purified by column chromatography using 5% hexane in DCM to provide the title compound (64.5 mg, 77% yield) as a colorless oil. ^{1}H NMR (600 MHz, $CDCl_3$) δ 7.85 – 7.80 (m, 2H), 7.29 – 7.23 (m, 2H), 7.20 – 7.09 (m, 7H), 6.97 – 6.91 (m, 2H), 4.47 (t, *J* = 6.1 Hz, 1H), 3.79 (t, *J* = 7.9 Hz, 1H), 2.96 (dd, *J* = 13.4, 6.9 Hz, 2H), 2.03 – 1.91 (m, 2H), 1.44 – 1.34 (m, 2H). ^{19}F NMR (565 MHz, $CDCl_3$) δ -105.19 – -105.26 (m), -116.81 – -116.90 (m). ^{13}C NMR (151 MHz, $CDCl_3$) δ 165.18 (d, *J* = 254.8 Hz), 161.52 (d, *J* = 244.6 Hz), 144.26 (s), 140.26 (d, *J* = 3.2 Hz), 136.19 (d, *J* = 3.2 Hz), 129.85 (d, *J* = 9.3 Hz), 129.20 (d, *J* = 7.8 Hz), 128.75 (s), 127.74 (s), 126.61 (s), 116.47 (d, *J* = 22.6 Hz), 115.42 (d, *J* = 21.2 Hz), 50.13 (s), 43.25 (s), 32.73 (s), 28.23 (s). IR (cm^-): 3275, 2937, 2363, 1592, 1507, 1493, 1326, 1292,1223, 1165, 1153, 1093, 838.
HRMS (ESI) calcd. For $[M+Na]^+$ 424.1153, found: 424.1150.
R_f = 0.45 (DCM).

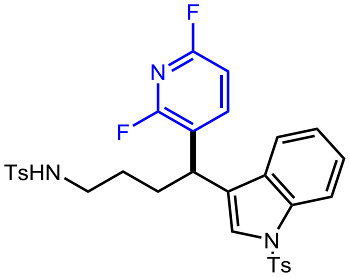

N-(4-(2,6-difluoropyridin-3-yl)-4-(1-tosyl-1H-indol-3-yl)butyl)-N-fluoro-4-methylbenzenesulfonamide (4)

Prepared following general procedure A using substrate (400 mg, 0.81 mmol) After 30 hours, the reaction mixture was purified by column chromatography using 50-70% dichloromethane in hexanes to 3% diethyl ether in hexanes to give the title compound **(**11.5 mg, 0.018 mmol 63%). ^{1}H NMR (600 MHz, $CDCl_3$) δ 7.95 (d, *J* = 8.4 Hz, 1H), 7.76 (d, *J* = 8.4 Hz, 4H), 7.72 (d, *J* = 8.2 Hz, 2H), 7.53 (m, 1H), 7.49 (s, 1H), 7.30 – 7.27 (m, 3H), 7.24 (d, *J* = 8.2 Hz, 2H), 7.19 (d, *J* = 7.8 Hz, 1H), 7.13 (t, *J* = 7.5 Hz, 1H), 6.66 (dd, *J* = 8.1, 2.7 Hz, 1H), 4.52 (t, *J* = 6.3 Hz, 1H), 4.25 (t, *J* = 7.6 Hz, 1H), 3.03 – 2.94 (m, 2H), 2.40 (s, 3H), 2.35 (s, 3H), 2.22 – 2.09 (m, 1H), 2.03 – 1.90 (m, 2H), 1.49 – 1.37 (m, 2H).^{19}F NMR (565 MHz, $CDCl_3$) δ -70.65 – -70.72 (m), -72.71 (t, *J* = 10.3 Hz). ^{13}C NMR (151 MHz, $CDCl_3$) δ 160.15 (dd, *J* = 246.1, 13.8 Hz), 158.85 (dd, *J* = 245.0, 14.1 Hz), 145.32 (s), 143.80 (s), 136.92 (s), 135.55 (s), 135.12 (s), 130.13 (s), 129.93 (s), 129.73 (s), 127.19 (s), 126.96 (s), 125.32 (s), 123.67 (s), 123.57 (s), 123.31 (s), 121.44 (dd, *J* = 26.9, 5.9 Hz), 119.49 (s), 114.05 (s), 106.77 (dd, *J* = 34.6, 5.6 Hz), 42.97 (s), 34.06 (s), 31.31 (s), 27.83 (s), 21.73 (s), 21.64 (s). IR (flim) cm^{-1} 3281, 2926, 2856, 2254, 1605, 1465, 1157.
HRMS (ESI-TOF) *m/z* calc'd For $[M+Na]^+$ 632.1465, found 632.1445.
R_f = 0.2 (DCM).

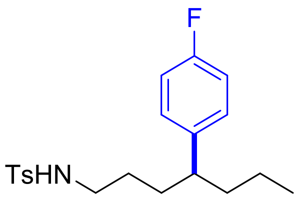

N-(4-(4-fluorophenyl)heptyl)-4-methylbenzenesulfonamide (5)

Prepared following general procedure B using substrate (57.4 mg, 0.2 mmol). After 16 hours, the reaction mixture was purified by column chromatography using 5% hexane in DCM, purified by prepare plate in 10% hexane in DCM to provide the title compound as a colorless oil. ^{1}H NMR (600 MHz, $CDCl_3$) δ 7.69 (t, *J* = 5.1 Hz, 2H), 7.29 – 7.26 (m, 2H), 7.02 – 6.97 (m, 2H), 6.96 – 6.91 (m, 2H), 4.28 (t, *J* = 6.1 Hz, 1H), 2.89 – 2.82 (m, 2H), 2.42 (s, 3H), 2.42 – 2.37 (m, 1H), 1.55 – 1.38 (m, 3H), 1.36 – 1.16 (m, 3H), 1.15 – 1.02 (m, 2H), 0.81 (t, *J* = 7.3 Hz, 3H). ^{19}F NMR (565 MHz, $CDCl_3$) δ -117.31 – -117.38 (m). ^{13}C NMR (151 MHz, $CDCl_3$) δ 161.40 (d, *J* = 243.5 Hz), 143.50 (s), 140.91 (d, *J* = 3.1 Hz), 137.13 (s), 129.80 (s), 128.90 (d, *J* = 7.7 Hz), 127.20 (s), 115.22 (d, *J* = 21.0 Hz), 44.74 (s), 43.34 (s), 39.36 (s), 33.87 (s), 27.78 (s), 21.64 (s), 20.66 (s), 14.15 (s). IR (cm^-): 3275, 2925, 2869, 2159, 1599, 1522, 1321, 1220, 1156, 1093, 832.
HRMS (ESI) calcd. For $[M+Na]^+$ 386.1560, found: 386.1552.
R_f = 0.55 (DCM).

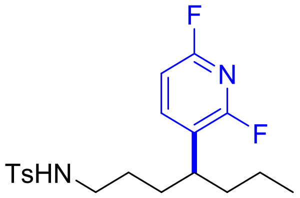

N-(4-(2,6-difluoropyridin-3-yl)heptyl)-4-methylbenzenesulfonamide (6)

Prepared following general procedure B using substrate (57.4 mg, 0.2 mmol). After 16 hours, the reaction mixture was purified by column chromatography using 2% Et_2O in DCM to provide the title compound (39.7 mg, 52% yield) as a colorless oil. ^{1}H NMR (600 MHz, $CDCl_3$) δ 7.71 – 7.68 (m, 2H), 7.61 (dd, *J* = 17.1, 8.0 Hz, 1H), 7.28 (d, *J* = 7.9 Hz, 2H), 6.75 (dd, *J* = 8.1, 2.9 Hz, 1H), 4.71 (t, *J* = 6.2 Hz, 1H), 2.88 (dd, *J* = 13.3, 6.8 Hz, 2H), 2.79 – 2.72 (m, 1H), 2.42 (s, 3H), 1.63 (ddd, *J* = 13.7, 8.2, 4.1 Hz, 1H), 1.57 – 1.44 (m, 3H), 1.39 – 1.29 (m, 1H), 1.27 – 1.17 (m, 1H), 1.17 – 1.03 (m, 2H), 0.82 (t, *J* = 7.3 Hz, 3H). ^{19}F NMR (565 MHz, $CDCl_3$) δ -72.12 – -72.27 (m, 1F), -72.95 (t, *J* = 10.1 Hz, 1F). ^{13}C NMR (151 MHz, $CDCl_3$) δ 159.69 (dd, *J* = 244.6, 14.2 Hz), 159.35 (dd, *J* = 245.0, 13.5 Hz),143.62 (s), 143.54 – 143.40 (m), 136.99 (s), 129.83 (s), 127.14 (s), 123.02 (dd, *J* = 27.3, 5.8 Hz), 106.31 (dd, *J* = 34.3, 5.6 Hz), 43.11 (s), 37.56 (s), 37.39 (d, *J* = 1.9 Hz), 32.28 (s), 27.58 (s), 21.61 (s), 20.49 (s), 13.99 (s). IR (cm-): 3283, 2958, 2931, 2872, 2369, 2345, 1609, 1467, 1409, 1324, 1304, 1158, 1093, 992, 814.
HRMS (ESI) calcd. For $[M+Na]^+$ 405.1419, found: 405.1418.
R_f = 0.25 (DCM).

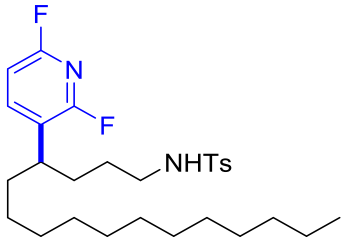

N-(4-(2,6-difluoropyridin-3-yl)hexadecyl)-4-methylbenzenesulfonamide (7)

Prepared following general procedure B using substrate (79.0 mg, 0.2 mmol). After 16 hours, the reaction mixture was purified by column chromatography using 2% Et_2O in DCM to provide the title compound (50.8 mg, 50% yield) as a colorless oil. ^{1}H NMR (600 MHz, $CDCl_3$) δ 7.70 (d, *J* = 8.3 Hz, 2H), 7.60 (dd, *J* = 17.0, 8.0 Hz, 1H), 7.27 (d, *J* = 7.9 Hz, 2H), 6.74 (dd, *J* = 8.1, 2.9 Hz, 1H), 4.79 (t, *J* = 6.2 Hz, 1H), 2.87 (dd, *J* = 13.3, 6.8 Hz, 2H), 2.76 – 2.70 (m,

1H), 2.42 (s, 3H), 1.67 – 1.59 (m, 1H), 1.58 – 1.52 (m, 1H), 1.48 (ddt, *J* = 23.5, 9.5, 4.8 Hz, 2H), 1.34 (ddd, *J* = 12.4, 11.7, 6.2 Hz, 1H), 1.31 – 1.07 (m, 20H), 1.02 (dt, *J* = 29.7, 14.9 Hz, 1H), 0.86 (t, *J* = 7.1 Hz, 3H). ^{19}F NMR (565 MHz, $CDCl_3$) δ -72.16 – -72.31 (m, 1F), -72.94 (t, *J* = 10.0 Hz, 1F). ^{13}C NMR (151 MHz, $CDCl_3$) δ 159.67 (dd, *J* = 244.6, 14.2 Hz), 159.33 (dd, *J* = 245.0, 13.6 Hz), 143.58 (s), 143.46 (t, *J* = 6.7 Hz), 136.99 (s), 129.82 (s), 127.13 (s), 123.06 (dd, *J* = 27.3, 5.7 Hz), 106.29 (dd, *J* = 34.3, 5.6 Hz), 43.11 (s), 37.69 (s), 35.40 (s), 32.32 (s), 32.01 (s), 29.74 (s), 29.73 (s), 29.71 (s), 29.66 (s), 29.59 (s), 29.56 (s), 29.44 (s), 27.56 (s), 27.38 (s), 22.79 (s), 21.60 (s), 14.22 (s).
HRMS (ESI) calcd. For $[M+Na]^+$ 531.2827, found 531.2823.
R_f = 0.30 (DCM).

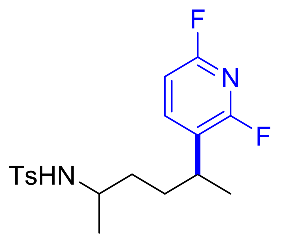

N-5-(2,6-difluoropyridin-3-yl)hexan-2-yl)-4-methylbenzenesulfonamide (*dr* =1:1) (8)
Prepared following general procedure B using substrate (54.6 mg, 0.2 mmol). After 16 hours, the reaction mixture was purified by column chromatography using 2% Et_2O in DCM to provide the title compound (38.3 mg, 52% yield, 1:1 d.r.) as a colorless oil. ^{1}H NMR (600 MHz, $CDCl_3$) δ 7.76 – 7.68 (m, 2H), 7.63 (dd, *J* = 16.6, 8.4 Hz, 1H), 7.26 (dd, *J* = 8.7, 7.4 Hz, 2H), 6.74 (dd, *J* = 8.1, 2.9 Hz, 1H), 4.77 – 4.60 (m, 1H), 3.35 – 3.18 (m, 1H), 2.90 – 2.77 (m, 1H), 2.40 (d, *J* = 4.7 Hz, 3H), 1.61 – 1.50 (m, 1H), 1.50 – 1.39 (m, 1H), 1.38 – 1.27 (m, 1H), 1.27 – 1.16 (m, 1H), 1.14 (dt, *J* = 13.2, 6.6 Hz, 3H), 0.98 – 0.92 (m, 3H). ^{19}F NMR (565 MHz, $CDCl_3$) δ -72.46 – -72.68 (m, 2F), -72.89 (t, *J* = 10.5 Hz, 1F), -73.21 (t, *J* = 10.5 Hz, 1F). Isomer A: ^{13}C NMR (151 MHz, $CDCl_3$) δ δ 159.64 (dd, *J* = 247.2, 16.5 Hz), 159.63 (dd, *J* = 245.8, 15.1 Hz), 143.45 (s), 143.00 – 142.74 (m), 138.31 (s), 129.76 (s), 127.08 (s), 124.80 (dd, *J* = 20.3, 6.0 Hz), 106.27 (dd, *J* = 11.3, 5.6 Hz), 50.11 (s), 35.40 (s), 32.57 (s), 32.19 (s), 31.79 (s), 21.79 (d, *J* = 20.3 Hz), 21.58 (s). Isomer B: ^{13}C NMR (151 MHz, $CDCl_3$) δ 158.92 (dd, *J* = 245.3, 3.9 Hz), 158.83 (dd, *J* = 245.3, 3.5 Hz),143.44 (s), 142.87 – 142.74 (m), 138.27 (s), 129.74 (s), 127.06 (s), 124.62 (dd, *J* = 20.6, 6.1 Hz), 106.04 (dd, *J* = 11.3, 5.6 Hz), 49.93 (s), 35.31 (s), 32.57 (s), 32.17 (s), 31.78 (s), 21.58 (s), 20.68 (d, *J* = 26.2 Hz). IR (cm^-): 3275, 2968, 2932, 2360, 2341, 1609, 1590, 1467, 1409, 1381, 1319, 1302, 1157, 1091, 991, 899.
HRMS (ESI) calcd. For $[M+Na]^+$ 391.1262, found:391.1257.
R_f = 0.25 (DCM).

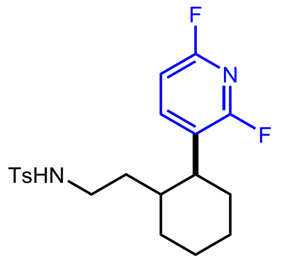

N-(2-(2,6-difluoropyridin-3-yl)cyclohexyl)ethyl)-4-methylbenzenesulfonamide (9)
Prepared following general procedure B using substrate (59.9 mg, 0.2 mmol**)**. After 26 hours, the reaction mixture was concentrated and a crude NMR was taken with DCE (1 equiv) as standard indicating 57% (3:1 d.r.) of the target product. The reaction mixture was then purified by column chromatography using 30% hexanes in DCM to 2% Et2O in DCM to give the title compound as an inseparable mixture of diastereomers as a clear oil (26.4 mg, 0.067 mmol 33%, 3:1 d.r.) Major Diastereomer A: ^{1}H NMR (600 MHz, $CDCl_3$) δ 7.78 – 7.52 (m, 3H), 7.32 – 7.26 (m, 2H), 6.83 – 6.74 (m, 1H), 4.43 – 4.32 (m, 1H), 2.92 –2.72 (m, 2H), 2.50 – 2.44 (m, 1H), 2.43 (s, 3H), 1.9 – 0.92 (m, 10H)**.** Minor Diastereomer B: ^{1}H NMR (600 MHz, $CDCl_3$) δ 7.78 – 7.52 (m, 3H), 7.32 – 7.26 (m, 2H), 6.74 – 6.72 (m, 1H), 4.43 – 4.32 (m, 1H), 3.04 (dt, *J* = 12.4, 3.4, 1H), 2.93 – 2.71 (m, 1H), 2.59 –2.50 (m, 1H), 2.43 (s, 3H), 1.9 – 0.92 (m, 10H)**.** ^{13}C NMR (151 MHz, $CDCl_3$) δ 159.76 (dd, *J* = 244.6, 14.3 Hz), 159.61 (dd, *J* = 244.8, 14.3 Hz), 158.99 (dd, *J* = 244.6, 13.7 Hz), 158.82 (dd, *J* = 246.4, 13.5 Hz), 143.63 (s), 143.61 (s), 136.86 (s), 129.83 (s), 129.82 (s), 127.11 (s), 123.60 (dd, *J* = 27.5, 5.7 Hz), 123.12 (dd, *J* = 26.6, 5.7 Hz), 106.49 (dd, *J* = 34.2, 5.5 Hz), 105.68 (dd, *J* = 33.8, 5.6 Hz), 60.53

(s), 41.97 (s), 40.60 (s), 39.21 (s), 38.65 (s), 34.80 (s), 34.27 (s), 33.92 (s), 31.72 (s), 29.82 (s), 29.08 (s), 26.43 (s), 26.22 (s), 25.99 (s), 25.18 (s), 21.62 (s), 20.15 (s). ^{19}F NMR (565 MHz, $CDCl_3$) δ -71.95 (t), -71.99 – -72.04 (m), -72.51 – -72.56 (m). IR (flim) cm^{-1} 3274, 2927, 2856, 1606, 1466, 1156.
HRMS (ESI-TOF) *m/z* calc'd for $[M+Na]^+$ 417.1424, found 417.1408.
R_f = 0.4 (DCM)

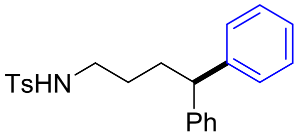

N-(4,4-diphenylbutyl)-4-methylbenzenesulfonamide (10)

Prepared following general procedure B using substrate (64.2 mg, 0.2 mmol). After 16 hours, the reaction mixture was purified by column chromatography using 5% hexane in DCM to provide the title compound (53.1 mg, 70% yield) as a colorless oil. ^{1}H NMR (600 MHz, $CDCl_3$) δ 7.71 (d, *J* = 8.3 Hz, 2H), 7.30 – 7.22 (m, 6H), 7.19 – 7.14 (m, 6H), 4.34 (t, *J* = 6.2 Hz, 1H), 3.80 (t, *J* = 7.9 Hz, 1H), 2.95 (dd, *J* = 13.3, 6.9 Hz, 2H), 2.42 (s, 3H), 2.00 (ddd, *J* = 10.3, 7.9, 5.3 Hz, 2H), 1.47 – 1.35 (m, 2H). ^{13}C NMR (151 MHz, $CDCl_3$) δ 144.57 (s), 143.49 (s), 137.09 (s), 129.83 (s), 128.63 (s), 127.86 (s), 127.21 (s), 126.40 (s), 50.92 (s), 43.27 (s), 32.62 (s), 28.23 (s), 21.66 (s). IR (cm^-): 3276, 3059, 3025, 2925, 2864, 2360, 2341, 1597, 1507, 1450, 1420, 1322, 1304, 1157, 1093, 963, 907.
HRMS (ESI) calcd. For $[M+Na]^+$ 402.1498, found: 402.1483.
R_f = 0.45 (DCM).

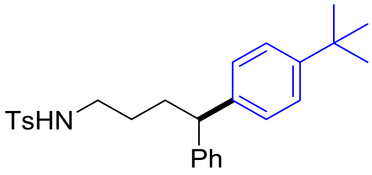

N-(4-(4-(tert-butyl)phenyl)-4-phenylbutyl)-4-methylbenzenesulfonamide (11)

Prepared following general procedure B using substrate (64.2 mg, 0.2 mmol). After 16 hours, the reaction mixture was purified by column chromatography using 5% hexane in DCM to provide the title compound (62.6 mg, 72% yield) as a colorless oil. ^{1}H NMR (600 MHz, $CDCl_3$) δ 7.73 – 7.69 (m, 2H), 7.29 – 7.21 (m, 6H), 7.16 (d, *J* = 8.0 Hz, 3H), 7.10 – 7.06 (m, 2H), 4.43 (t, *J* = 6.2 Hz, 1H), 3.75 (t, *J* = 7.8 Hz, 1H), 2.93 (q, *J* = 6.9 Hz, 2H), 2.41 (s, 3H), 2.01 – 1.95 (m, 2H), 1.44 – 1.35 (m, 2H), 1.27 (s, 9H). ^{13}C NMR (101 MHz, $CDCl_3$) δ 149.06 (s), 144.77 (s), 143.44 (s), 141.50 (s), 137.09 (s), 129.81 (s), 128.57 (s), 127.88 (s), 127.37 (s), 127.21 (s), 126.30 (s), 125.46 (s), 50.48 (s), 43.28 (s), 34.45 (s), 32.72 (s), 31.48 (s), 28.21 (s), 21.65 (s). IR (cm^-): 3279, 3025, 2959, 2866, 2360, 2341, 1598, 1508, 1493, 1451, 1411, 1323, 1305, 1157, 1093, 1018, 963, 908.
HRMS (ESI) calcd. For $[M+Na]^+$ 458.2124, found: 458.2116.
R_f = 0.45 (DCM).

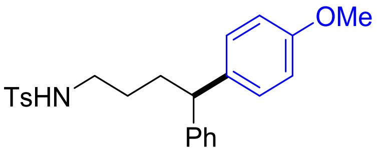

N-(4-(4-methoxyphenyl)-4-phenylbutyl)-4-methylbenzenesulfonamide (12)

Prepared following general procedure B using substrate (64.2 mg, 0.2 mmol). After 16 hours, the reaction mixture was purified by column chromatography using 1% Et_2O in DCM to provide the title compound (63.0 mg, 77% yield) as a colorless oil. ^{1}H NMR (600 MHz, $CDCl_3$) δ 7.73 – 7.69 (m, 2H), 7.30 – 7.21 (m, 4H), 7.17 – 7.12 (m, 3H), 7.09 – 7.05 (m, 2H), 6.81 – 6.77 (m, 2H), 4.46 (t, *J* = 6.2 Hz, 1H), 3.76 (s, 3H), 3.74 (t, *J* = 7.9 Hz, 1H), 2.94 (q, *J* = 6.9 Hz, 2H), 2.43 (s, 3H), 2.00 – 1.92 (m, 2H), 1.44 – 1.36 (m, 2H). ^{13}C NMR (101 MHz, $CDCl_3$) δ 158.09 (s), 144.99 (s), 143.45 (s), 137.07 (s), 136.73 (s), 129.81 (s), 128.75 (s), 128.58 (s), 127.74 (s), 127.19 (s), 126.27 (s), 113.99 (s), 55.34 (s), 50.03 (s), 43.25 (s), 32.76 (s), 28.19 (s), 21.63 (s). IR (cm^-): 3278, 3024, 2938, 2866, 2835, 2360, 2343, 1608, 1598, 1510, 1499, 1451, 1420, 1323, 1303, 1245, 1177, 1156, 1093, 1032, 964, 907.

HRMS (ESI) calcd. For $[M+Na]^+$ 432.1604, found: 432.1590.
R_f = 0.35 (DCM).

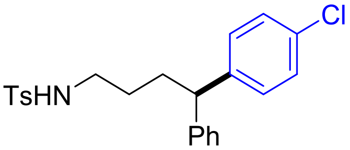

N-(4-(4-chlorophenyl)-4-phenylbutyl)-4-methylbenzenesulfonamide (13)

Prepared following general procedure B using substrate (64.2 mg, 0.2 mmol). After 16 hours, the reaction mixture was purified by column chromatography using 5% hexane in DCM to provide the title compound (53.7 mg, 65% yield) as a colorless oil. ^{1}H NMR (400 MHz, $CDCl_3$) δ 7.82 – 7.62 (m, 2H), 7.31 – 7.04 (m, 11H), 4.43 (t, *J* = 6.2 Hz, 1H), 3.78 (t, *J* = 7.9 Hz, 1H), 2.94 (q, *J* = 6.8 Hz, 2H), 2.42 (s, 3H), 2.03 – 1.92 (m, 2H), 1.45 – 1.34 (m, 2H). ^{13}C NMR (101 MHz, $CDCl_3$) δ 144.02 (s), 143.55 (s), 143.13 (s), 137.06 (s), 132.12 (s), 129.84 (s), 129.20 (s), 128.74 (s), 128.74 (s), 127.77 (s), 127.19 (s), 126.62 (s), 50.25 (s), 43.18 (s), 32.49 (s), 28.15 (s), 21.66 (s). IR (cm^-): 3279, 3026, 2927, 2867, 1598, 1489, 1451, 1409, 1322, 1157, 1091, 1013, 964, 905, 814.
HRMS (ESI) calcd. For $[M+Na]^+$ 436.1108, found: 436.1097.
R_f = 0.45 (DCM).

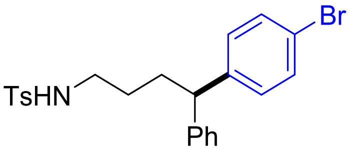

N-(4-(4-bromophenyl)-4-phenylbutyl)-4-methylbenzenesulfonamide (14)

Prepared following general procedure B using substrate (64.2 mg, 0.2 mmol). After 16 hours, the reaction mixture was purified by column chromatography using 5% hexane in DCM to provide the title compound (66.7 mg, 73% yield) as a colorless oil. ^{1}H NMR (600 MHz, $CDCl_3$) δ 7.71 (d, *J* = 8.3 Hz, 2H), 7.38 – 7.33 (m, 2H), 7.26 (dd, *J* = 13.0, 7.6 Hz, 4H), 7.17 (t, *J* = 7.4 Hz, 1H), 7.12 (d, *J* = 7.2 Hz, 2H), 7.03 (d, *J* = 8.4 Hz, 2H), 4.64 (t, *J* = 6.2 Hz, 1H), 3.76 (t, *J* = 7.9 Hz, 1H), 2.94 (q, *J* = 6.7 Hz, 2H), 2.42 (s, 3H), 1.98 (ddd, *J* = 10.2, 7.4, 1.9 Hz, 2H), 1.43 – 1.36 (m, 2H). ^{13}C NMR (151 MHz, $CDCl_3$) δ 143.94 (s), 143.67 (s), 143.52 (s), 137.03 (s), 131.65 (s), 129.83 (s), 129.61 (s), 128.72 (s), 127.76 (s), 127.17 (s), 126.60 (s), 120.14 (s), 50.29 (s), 43.14 (s), 32.39 (s), 28.07 (s), 21.65 (s). IR (cm^-): 3278, 3059, 3025, 2940, 2866, 2360, 2343, 1598, 1485, 1450, 1404, 1320, 1304, 1289, 1159, 1092, 1071, 1009, 962, 902, 812.
HRMS (ESI) calcd. For $[M+Na]^+$ 480.0603, found: 480.0612.
R_f = 0.45 (DCM).

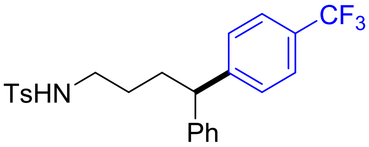

4-methyl-N-(4-phenyl-4-(4-(trifluoromethyl)phenyl)butyl)benzenesulfonamide (15)

Prepared following general procedure B using substrate (64.2 mg, 0.2 mmol). After 36 hours, the reaction mixture was purified by column chromatography using 5% hexane in DCM to provide the title compound (43.8 mg, 49% yield) as a colorless oil. ^{1}H NMR (600 MHz, $CDCl_3$) δ 7.72 – 7.69 (m, 2H), 7.50 (d, *J* = 8.1 Hz, 2H), 7.30 – 7.24 (m, 6H), 7.21 – 7.17 (m, 1H), 7.15 (dd, *J* = 8.1, 1.0 Hz, 2H), 4.40 (t, *J* = 6.2 Hz, 1H), 3.87 (t, *J* = 7.9 Hz, 1H), 2.96 (q, *J* = 6.7 Hz, 2H), 2.41 (s, 3H), 2.08 – 1.95 (m, 2H), 1.47 – 1.36 (m, 2H). ^{19}F NMR (565 MHz, $CDCl_3$) δ -62.40 (s). ^{13}C NMR (151 MHz, $CDCl_3$) δ 148.73 (s), 148.72 (s), 143.60 (s), 143.47 (s), 137.07 (s), 129.86 (s), 128.86 (s), 128.19 (s), 127.84 (s), 127.20 (s), 126.83 (s), 126.03 (q, *J* = 272.2 Hz), 125.59 (q, *J* = 3.7 Hz), 50.74 (s), 43.16 (s), 32.36 (s), 28.15 (s), 21.64 (s). IR (cm^-): 3276, 3027, 2926, 1617, 1598, 1499, 1451, 1417, 1322, 1155, 1114, 1093, 1066, 1017, 955.
HRMS (ESI) calcd. For $[M+Na]^+$ 470.1372, found: 470.1357.
R_f = 0.45 (DCM).

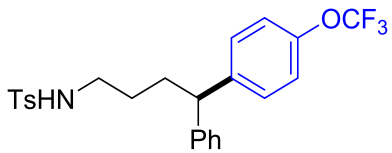

4-methyl-N-(4-phenyl-4-(4-(trifluoromethoxy)phenyl)butyl)benzenesulfonamide (16)

Prepared following general procedure B using substrate (64.2 mg, 0.2 mmol). After 16 hours, the reaction mixture was purified by column chromatography using 5% hexane in DCM to provide the title compound (49.1 mg, 53% yield) as a colorless oil. ^{1}H NMR (600 MHz, $CDCl_3$) δ 7.73 – 7.69 (m, 2H), 7.30 – 7.24 (m, 4H), 7.21 – 7.16 (m, 3H), 7.14 (dd, J = 8.1, 1.0 Hz, 2H), 7.09 (d, J = 8.0 Hz, 2H), 4.54 (t, J = 6.2 Hz, 1H), 3.82 (t, J = 7.9 Hz, 1H), 2.95 (q, J = 6.8 Hz, 2H), 2.41 (s, 3H), 2.00 (ddd, J = 11.6, 7.3, 3.3 Hz, 2H), 1.41 (tt, J = 14.2, 7.0 Hz, 2H). ^{19}F NMR (565 MHz, $CDCl_3$) δ -57.86 (s). ^{13}C NMR (151 MHz, $CDCl_3$) δ 147.69 (s), 143.89 (s), 143.56 (s), 143.40 (s), 137.05 (s), 129.84 (s), 129.08 (s), 128.78 (s), 127.82 (s), 127.19 (s), 126.68 (s), 121.07 (s), 120.60 (q, J = 256.8 Hz), 50.25 (s), 43.16 (s), 32.60 (s), 28.13 (s), 21.62 (s). IR (cm$^-$): 3277, 3028, 2943, 2868, 2360, 2341, 2256, 1598, 1506, 1499, 1452, 1419, 1322, 1309, 1220, 1153, 1093, 1018, 963, 907, 813.
HRMS (ESI) calcd. For $[M+Na]^+$ 486.1321, found: 486.1309
Rf = 0.45 (DCM)

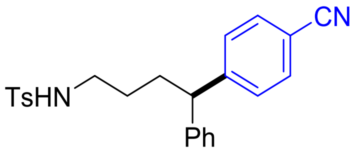

N-(4-(4-cyanophenyl)-4-phenylbutyl)-4-methylbenzenesulfonamide (17)

Prepared following general procedure B using substrate (64.2 mg, 0.2 mmol). After 16 hours, the reaction mixture was purified by column chromatography using 2% Et2O in DCM to provide the title compound (42.8 mg, 53% yield) as a colorless oil. ^{1}H NMR (600 MHz, $CDCl_3$) δ 7.73 – 7.69 (m, 2H), 7.54 – 7.51 (m, 2H), 7.28 (ddd, J = 8.8, 6.0, 3.4 Hz, 6H), 7.22 – 7.18 (m, 1H), 7.15 – 7.11 (m, 2H), 4.53 (t, J = 6.3 Hz, 1H), 3.87 (t, J = 7.9 Hz, 1H), 2.95 (ddd, J = 13.2, 6.7, 1.8 Hz, 2H), 2.42 (s, 3H), 2.09 – 1.97 (m, 2H), 1.46 – 1.35 (m, 2H). ^{13}C NMR (151 MHz, $CDCl_3$) δ 150.26 (s), 143.62 (s), 142.87 (s), 137.01 (s), 132.48 (s), 129.86 (s), 128.95 (s), 128.66 (s), 127.85 (s), 127.18 (s), 127.01 (s), 118.99 (s), 110.30 (s), 50.94 (s), 43.06 (s), 32.15 (s), 28.07 (s), 21.66 (s). IR (cm$^-$): 3276, 3060, 3027, 2942, 2867, 2360, 2341, 2224, 1598, 1492, 1457, 1414, 1322, 1304, 1289, 1154, 1092, 962, 903, 813.
HRMS (ESI) calcd. For $[M+Na]^+$ 427.1451, found: 427.1456.
Rf= 0.15 (DCM)

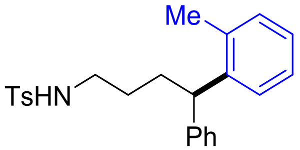

4-methyl-N-(4-phenyl-4-(o-tolyl)butyl)benzenesulfonamide (18)

Prepared following general procedure B using substrate (64.2 mg, 0.2 mmol). After 16 hours, the reaction mixture was purified by column chromatography using 5% hexane in DCM to provide the title compound (56.6 mg, 72% yield) as a colorless oil. ^{1}H NMR (600 MHz, $CDCl_3$) δ 7.72 – 7.68 (m, 2H), 7.29 – 7.26 (m, 2H), 7.25 – 7.21 (m, 3H), 7.20 – 7.12 (m, 2H), 7.12 – 7.08 (m, 4H), 4.23 (t, J = 6.2 Hz, 1H), 4.00 (t, J = 7.7 Hz, 1H), 2.95 (ddd, J = 13.3, 6.9, 2.8 Hz, 2H), 2.42 (s, 3H), 2.21 (s, 3H), 1.97 (dd, J = 15.6, 7.8 Hz, 2H), 1.50 – 1.38 (m, 2H). ^{13}C NMR (151 MHz, $CDCl_3$) δ 144.19 (s), 143.52 (s), 142.19 (s), 137.10 (s), 136.42 (s), 130.75 (s), 129.84 (s), 128.52 (s), 128.19 (s), 127.23 (s), 126.52 (s), 126.33 (s), 126.25 (s), 126.23 (s), 46.47 (s), 43.37 (s), 33.16 (s), 28.26 (s), 21.66 (s), 20.00 (s). IR (cm$^-$): 3276, 2925, 2863, 2360, 2341, 1491, 1457, 1324, 1305, 1158, 1094, 814.
HRMS (ESI) calcd. For $[M+Na]^+$ 416.1655, found: 416.1642.
R_f = 0.45 (DCM).

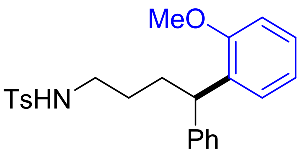

N-(4-(2-methoxyphenyl)-4-phenylbutyl)-4-methylbenzenesulfonamide (19)

Prepared following general procedure B using substrate (64.2 mg, 0.2 mmol). After 16 hours, the reaction mixture was purified by column chromatography using 5% hexane in DCM to provide the title compound (63.0 mg, 77% yield) as a colorless oil. ^{1}H NMR (600 MHz, $CDCl_3$) δ 7.72 – 7.68 (m, 2H), 7.29 – 7.21 (m, 4H), 7.19 – 7.13 (m, 4H), 7.07 (dd, J = 7.6, 1.6 Hz, 1H), 6.87 (td, J = 7.5, 1.0 Hz, 1H), 6.83 (dd, J = 8.2, 0.9 Hz, 1H), 4.38 (t, J = 6.1 Hz, 1H), 4.29 (t, J = 7.9 Hz, 1H), 3.79 (s, 3H), 3.02 – 2.87 (m, 2H), 2.42 (s, 3H), 1.95 (dd, J = 15.3, 7.8 Hz, 2H), 1.42 (dq, J = 13.6, 6.8 Hz, 2H). ^{13}C NMR (151 MHz, $CDCl_3$) δ 156.95 (s), 144.17 (s), 143.42 (s), 137.15 (s), 133.15 (s), 129.81 (s), 128.37 (s), 128.25 (s), 127.73 (s), 127.34 (s), 127.22 (s), 126.15 (s), 120.78 (s), 110.84 (s), 55.61 (s), 43.15 (s), 42.53 (s), 31.76 (s), 27.81 (s), 21.65 (s). IR (cm$^-$): 3280, 3026, 2933, 2865, 2360, 2341, 1597, 1490, 1458, 1437, 1324, 1241, 1158, 1099, 1028, 814.
HRMS (ESI) calcd. For $[M+Na]^+$ 432.1604, found: 432.1591.
R_f = 0.45 (DCM).

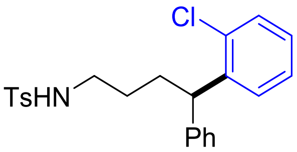

N-(4-(2-chlorophenyl)-4-phenylbutyl)-4-methylbenzenesulfonamide (20)

Prepared following general procedure B using substrate (64.2 mg, 0.2 mmol). After 16 hours, the reaction mixture was purified by column chromatography using 5% hexane in DCM to provide the title compound (52.9 mg, 64% yield) as a colorless oil. ^{1}H NMR (600 MHz, $CDCl_3$) δ 7.72 – 7.69 (m, 2H), 7.31 (dd, J = 7.9, 1.0 Hz, 1H), 7.29 – 7.22 (m, 4H), 7.22 – 7.16 (m, 5H), 7.11 (ddd, J = 8.0, 6.8, 2.2 Hz, 1H), 4.40 – 4.34 (m, 2H), 3.02 – 2.92 (m, 2H), 2.42 (s, 3H), 2.06 – 1.91 (m, 2H), 1.49 – 1.39 (m, 2H). ^{13}C NMR (151 MHz, $CDCl_3$) δ 143.51 (s), 143.06 (s), 141.97 (s), 137.09 (s), 134.22 (s), 129.84 (s), 129.83 (s), 128.59 (s), 128.49 (s), 128.20 (s), 127.59 (s), 127.22 (s), 127.17 (s), 126.58 (s), 46.23 (s), 43.24 (s), 32.36 (s), 27.98 (s), 21.66 (s). IR (cm$^-$): 2360, 2341, 1323, 1251, 1158, 1093, 1035, 906, 813.
HRMS (ESI) calcd. For $[M+Na]^+$ 436.1108, found: 436.1102.
R_f = 0.45 (DCM).

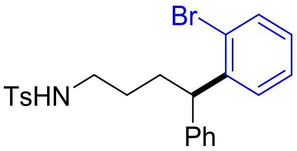

N-(4-(2-bromophenyl)-4-phenylbutyl)-4-methylbenzenesulfonamide (21)

Prepared following general procedure B using substrate (64.2 mg, 0.2 mmol). After 16 hours, the reaction mixture was purified by column chromatography using 5% hexane in DCM to provide the title compound (61.4 mg, 67% yield) as a colorless oil. ^{1}H NMR (600 MHz, $CDCl_3$) δ 7.73 – 7.69 (m, 2H), 7.51 (dd, J = 8.0, 1.2 Hz, 1H), 7.28 – 7.16 (m, 9H), 7.03 (ddd, J = 8.0, 7.2, 1.8 Hz, 1H), 4.46 (t, J = 6.2 Hz, 1H), 4.37 (t, J = 7.8 Hz, 1H), 2.97 (ddd, J = 13.2, 6.8, 2.0 Hz, 2H), 2.41 (s, 3H), 2.05 – 1.90 (m, 2H), 1.52 – 1.38 (m, 2H). ^{13}C NMR (151 MHz, $CDCl_3$) δ 143.62 (s), 143.49 (s), 143.04 (s), 137.07 (s), 133.15 (s), 129.83 (s), 128.75 (s), 128.57 (s), 128.20 (s), 127.90 (s), 127.82 (s), 127.21 (s), 126.58 (s), 125.25 (s), 48.80 (s), 43.23 (s), 32.59 (s), 27.91 (s), 21.66 (s). IR (cm$^-$): 3278, 3059, 3026, 2943, 2866, 2360, 2342, 1598, 1494, 1466, 1450, 1437, 1321, 1304, 1298, 1155, 1092, 1019, 962, 906, 813.
HRMS (ESI) calcd. For $[M+Na]^+$ 480.0603, found: 480.0600.
R_f = 0.45 (DCM).

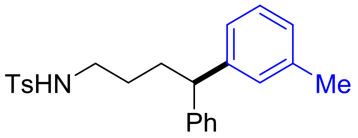

4-methyl-N-(4-phenyl-4-(m-tolyl)butyl)benzenesulfonamide (22)

Prepared following general procedure B using substrate (64.2 mg, 0.2 mmol). After 16 hours, the reaction mixture was purified by column chromatography using 5% hexane in DCM to provide the title compound (43.2 mg, 55% yield) as a colorless oil.^{1}H NMR (600 MHz, $CDCl_3$) δ 7.71 (d, *J* = 8.3 Hz, 2H), 7.31 – 7.22 (m, 4H), 7.18 – 7.12 (m, 4H), 7.01 – 6.94 (m, 3H), 4.32 (t, *J* = 6.1 Hz, 1H), 3.75 (t, *J* = 7.8 Hz, 1H), 2.95 (q, *J* = 6.8 Hz, 2H), 2.42 (s, 3H), 2.29 (s, 3H), 1.99 (ddd, *J* = 10.0, 7.9, 5.6 Hz, 2H), 1.45 – 1.35 (m, 2H). ^{13}C NMR (151 MHz, $CDCl_3$) δ 144.70 (s), 144.50 (s), 143.48 (s), 138.17 (s), 137.09 (s), 129.82 (s), 128.68 (s), 128.61 (s), 128.50 (s), 127.84 (s), 127.22 (s), 127.19 (s), 126.35 (s), 124.80 (s), 50.91 (s), 43.30 (s), 32.64 (s), 28.25 (s), 21.65 (s), 21.63 (s). IR (cm^{-}): 3275, 2923, 1598, 1493, 1450, 1420, 1321, 1304, 1154, 1092, 1018, 964, 813.
HRMS (ESI) calcd. For $[M+Na]^+$ 416.1655, found: 416.1651.
R_f = 0.45 (DCM).

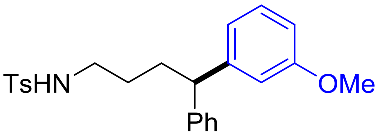

N-(4-(3-methoxyphenyl)-4-phenylbutyl)-4-methylbenzenesulfonamide (23)

Prepared following general procedure B using substrate (64.2 mg, 0.2 mmol). After 16 hours, the reaction mixture was purified by column chromatography using 5% hexane in DCM to provide the title compound (55.6 mg, 68% yield) as a colorless oil. ^{1}H NMR (600 MHz, $CDCl_3$) δ 7.73 – 7.67 (m, 2H), 7.27 (dd, *J* = 8.5, 0.5 Hz, 2H), 7.25 – 7.23 (m, 2H), 7.20 – 7.13 (m, 4H), 6.79 – 6.74 (m, 1H), 6.73 – 6.68 (m, 2H), 4.38 (t, *J* = 6.2 Hz, 1H), 3.78 – 3.74 (m, 4H), 2.94 (q, *J* = 6.9 Hz, 2H), 2.42 (s, 3H), 1.98 (ddd, *J* = 10.0, 7.9, 5.6 Hz, 2H), 1.44 – 1.38 (m, 2H). ^{13}C NMR (151 MHz, $CDCl_3$) δ 159.80 (s), 146.23 (s), 144.42 (s), 143.49 (s), 137.07 (s), 129.83 (s), 129.57 (s), 128.62 (s), 127.80 (s), 127.20 (s), 126.43 (s), 120.28 (s), 114.09 (s), 111.25 (s), 55.26 (s), 50.92 (s), 43.26 (s), 32.55 (s), 28.20 (s), 21.64 (s). IR (cm^{-}): 3279, 2926, 2360, 2341, 2256, 1596, 1582, 1488, 1450, 1435, 1320, 1289, 1259, 1154, 1092, 1044, 907.
HRMS (ESI) calcd. For $[M+Na]^+$ 432.1604, found: 432.1599.
R_f = 0.35 (DCM).

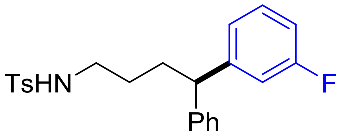

N-(4-(3-fluorophenyl)-4-phenylbutyl)-4-methylbenzenesulfonamide (24)

Prepared following general procedure B using substrate (64.2 mg, 0.2 mmol). After 16 hours, the reaction mixture was purified by column chromatography using 5% hexane in DCM to provide the title compound (61.1 mg, 77% yield) as a colorless oil. ^{1}H NMR (600 MHz, $CDCl_3$) δ 7.73 – 7.67 (m, 2H), 7.30 – 7.24 (m, 4H), 7.24 – 7.16 (m, 2H), 7.16 – 7.11 (m, 2H), 6.95 (d, *J* = 7.7 Hz, 1H), 6.85 (ddd, *J* = 11.0, 4.1, 1.4 Hz, 2H), 4.32 (t, *J* = 6.2 Hz, 1H), 3.79 (t, *J* = 7.9 Hz, 1H), 2.95 (q, *J* = 6.8 Hz, 2H), 2.42 (s, 3H), 2.02 – 1.94 (m, 2H), 1.48 – 1.35 (m, 2H). ^{19}F NMR (565 MHz, $CDCl_3$) δ -113.05 (td, *J* = 9.4, 6.2 Hz). ^{13}C NMR (151 MHz, $CDCl_3$) δ 163.08 (d, *J* = 245.8 Hz), 147.28 (d, *J* = 6.7 Hz), 143.69 (d, *J* = 35.1 Hz), 137.08 (s), 130.04 (d, *J* = 8.3 Hz), 129.86 (s), 128.77 (s), 127.81 (s), 127.21 (s), 126.70 (s), 123.55 (d, *J* = 2.7 Hz), 114.68 (d, *J* = 21.4 Hz), 113.30 (d, *J* = 21.1 Hz), 50.63 (s), 43.20 (s), 32.46 (s), 28.17 (s), 21.65 (s). IR (cm^{-}): 3277, 3061, 3027, 2925, 2867, 1613, 1588, 1485, 1445, 1321, 1305, 1155, 1092, 1018, 908.
HRMS (ESI) calcd. For $[M+Na]^+$ 420.1404, found: 420.1400.
R_f = 0.45 (DCM).

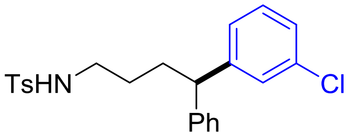

N-(4-(3-chlorophenyl)-4-phenylbutyl)-4-methylbenzenesulfonamide (25)

Prepared following general procedure B using substrate (64.2 mg, 0.2 mmol). After 16 hours, the reaction mixture was purified by column chromatography using 5% hexane in DCM to provide the title compound (56.1 mg, 68% yield) as a colorless oil. ^{1}H NMR (600 MHz, $CDCl_3$) δ 7.73 – 7.68 (m, 2H), 7.27 (ddd, *J* = 7.7, 5.7, 2.6 Hz, 4H), 7.21 – 7.16 (m, 2H), 7.16 – 7.12 (m, 4H), 7.05 (dt, *J* = 7.6, 1.2 Hz, 1H), 4.37 (t, *J* = 6.2 Hz, 1H), 3.77 (t, *J* = 7.9 Hz, 1H), 2.95 (q, *J* = 6.8 Hz, 2H), 2.42 (s, 3H), 2.03 – 1.93 (m, 2H), 1.40 (dq, *J* = 14.3, 7.1 Hz, 2H). ^{13}C NMR (151 MHz, $CDCl_3$) δ 146.74 (s), 143.69 (s), 143.57 (s), 137.06 (s), 134.43 (s), 129.90 (s), 129.86 (s), 128.79 (s), 127.97 (s), 127.82 (s), 127.21 (s), 126.72 (s), 126.63 (s), 126.08 (s), 50.64 (s), 43.18 (s), 32.41 (s), 28.15 (s), 21.66 (s). IR (cm^{-}): 3277, 2943, 2360, 2341, 1636, 1593, 1494, 1473, 1451, 1427, 1321, 1155, 1092, 1017, 813.
HRMS (ESI) calcd. For $[M+Na]^+$ 436.1108, found:436.1105.
R_f = 0.45 (DCM).

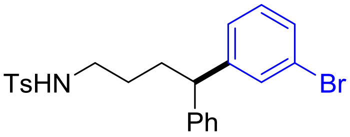

N-(4-(3-bromophenyl)-4-phenylbutyl)-4-methylbenzenesulfonamide (26)

Prepared following general procedure B using substrate (64.2 mg, 0.2 mmol). After 16 hours, the reaction mixture was purified by column chromatography using 5% hexane in DCM to provide the title compound (61.2 mg, 67% yield) as a colorless oil. ^{1}H NMR (600 MHz, $CDCl_3$) δ 7.74 – 7.69 (m, 2H), 7.31 – 7.24 (m, 6H), 7.18 (ddd, *J* = 6.8, 2.4, 1.2 Hz, 1H), 7.15 – 7.07 (m, 4H), 4.52 (t, *J* = 6.2 Hz, 1H), 3.75 (t, *J* = 7.9 Hz, 1H), 2.94 (q, *J* = 6.8 Hz, 2H), 2.42 (s, 3H), 2.04 – 1.88 (m, 2H), 1.45 – 1.34 (m, 2H). ^{13}C NMR (151 MHz, $CDCl_3$) δ 147.06 (s), 143.66 (s), 143.54 (s), 137.03 (s), 130.87 (s), 130.20 (s), 129.84 (s), 129.53 (s), 128.77 (s), 127.81 (s), 127.19 (s), 126.69 (s), 126.52 (s), 122.72 (s), 50.61 (s), 43.14 (s), 32.40 (s), 28.09 (s), 21.66 (s). IR (cm^{-}): 3276, 3059, 3025, 2939, 2865, 2360, 2341, 1592, 1565, 1473, 1451, 1424, 1321, 1304, 1155, 1093, 1073, 813.
HRMS (ESI) calcd. For $[M+Na]^+$ 480.0603, found:480.0597.
R_f = 0.45 (DCM).

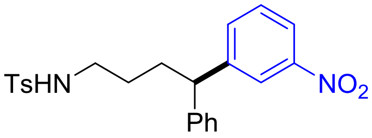

4-methyl-N-(4-(3-nitrophenyl)-4-phenylbutyl)benzenesulfonamide (27)

Prepared following general procedure B using substrate (64.2 mg, 0.2 mmol). Adjust reaction tempreture to 25 ℃, after 18 hours, the reaction mixture was purified by column chromatography using 1% Et_2O in DCM to provide the title compound (54.2 mg, 64% yield) as a yellow oil. ^{1}H NMR (600 MHz, $CDCl_3$) δ 8.05 – 8.00 (m, 2H), 7.73 – 7.68 (m, 2H), 7.51 (d, *J* = 7.7 Hz, 1H), 7.41 (t, *J* = 7.9 Hz, 1H), 7.30 – 7.26 (m, 4H), 7.22 – 7.18 (m, 1H), 7.17 – 7.13 (m, 2H), 4.67 (t, *J* = 6.2 Hz, 1H), 3.92 (t, *J* = 7.9 Hz, 1H), 2.95 (q, *J* = 6.7 Hz, 2H), 2.41 (s, 3H), 2.13 – 1.97 (m, 2H), 1.49 – 1.37 (m, 2H). ^{13}C NMR (151 MHz, $CDCl_3$) δ 148.51 (s), 146.93 (s), 143.61 (s), 142.89 (s), 136.97 (s), 134.18 (s), 129.85 (s), 129.55 (s), 128.98 (s), 127.82 (s), 127.17 (s), 127.03 (s), 122.57 (s), 121.57 (s), 50.55 (s), 43.04 (s), 32.29 (s), 28.04 (s), 21.62 (s). IR (cm^{-}): 3299, 3062, 3027, 2928, 2867, 2360, 1598, 1525, 1494, 1420, 1347, 1324, 1305, 1155, 1093, 967, 907, 814.
HRMS (ESI) calcd. For $[M+Na]^+$ 447.1349, found: 447.1346.
R_f = 0.35 (DCM).

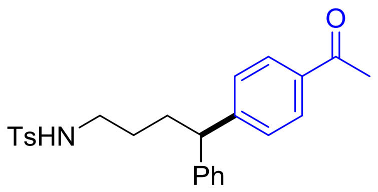

N-(4-(4-acetylphenyl)-4-phenylbutyl)-4-methylbenzenesulfonamide (28)

Prepared following general procedure B using substrate (64.2 mg, 0.2 mmol). After 18 hours, the reaction mixture was purified by column chromatography using 5% hexane in DCM to provide the title compound (53.0 mg, 63% yield) as a colorless oil. ^{1}H NMR (600 MHz, $CDCl_3$) δ 7.86 – 7.80 (m, 2H), 7.71 (d, J = 8.3 Hz, 2H), 7.27 – 7.23 (m, 6H), 7.19 – 7.12 (m, 3H), 4.88 (t, J = 6.2 Hz, 1H), 3.86 (t, J = 7.8 Hz, 1H), 2.94 (q, J = 6.7 Hz, 2H), 2.54 (s, 3H), 2.40 (s, 3H), 2.03 (dd, J = 15.6, 7.9 Hz, 2H), 1.45 – 1.36 (m, 2H). ^{13}C NMR (151 MHz, $CDCl_3$) δ 197.90 (s), 150.29 (s), 143.55 (s), 143.45 (s), 137.00 (s), 135.39 (s), 129.78 (s), 128.75 (s), 128.73 (s), 128.05 (s), 127.81 (s), 127.13 (s), 126.66 (s), 50.83 (s), 43.09 (s), 32.20 (s), 28.01 (s), 26.63 (s), 21.59 (s). IR (cm$^-$): 3275, 3027, 2943, 2868, 2360, 2342, 2254, 1675, 1598, 1493, 1412, 1359, 1322, 1304, 1269, 1156, 1119, 1092, 1016, 958, 906, 813.
HRMS (ESI) calcd. For $[M+Na]^+$ 444.1604, found: 444.1599.
R_f = 0.25 (5% Et_2O/DCM).

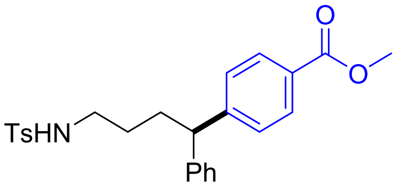

Methyl-4-(4-((4-methylphenyl)sulfonamido)-1-phenylbutyl)benzoate (29)

Prepared following general procedure B using substrate (64.2 mg, 0.2 mmol). After 16 hours, the reaction mixture was purified by column chromatography using 5% hexane in DCM to provide the title compound (56.8 mg, 65% yield) as a colorless oil. ^{1}H NMR (600 MHz, $CDCl_3$) δ 7.93 – 7.89 (m, 2H), 7.72 – 7.69 (m, 2H), 7.26 (d, J = 7.7 Hz, 3H), 7.24 – 7.21 (m, 3H), 7.19 – 7.15 (m, 1H), 7.14 (dd, J = 8.1, 1.0 Hz, 2H), 4.63 (t, J = 6.2 Hz, 1H), 3.88 (s, 3H), 3.85 (t, J = 7.9 Hz, 1H), 2.94 (q, J = 6.8 Hz, 2H), 2.41 (s, 3H), 2.05 – 1.98 (m, 2H), 1.44 – 1.36 (m, 2H). ^{13}C NMR (151 MHz, $CDCl_3$) δ 167.07 (s), 149.98 (s), 143.63 (s), 143.51 (s), 137.05 (s), 129.97 (s), 129.82 (s), 128.75 (s), 128.33 (s), 127.89 (s), 127.84 (s), 127.17 (s), 126.68 (s), 52.14 (s), 50.87 (s), 43.14 (s), 32.30 (s), 28.08 (s), 21.62 (s). IR (cm$^-$): 3276, 3027, 2998, 2867, 2360, 2341, 1716, 1609, 1598, 1493, 1451, 1439, 1415, 1321, 1278, 1182, 1155, 1109, 1093, 1018, 963, 907, 814.
HRMS (ESI) calcd. For $[M+Na]^+$ 460.1553, found: 460.1537.
R_f = 0.35 (5% Et_2O/DCM).

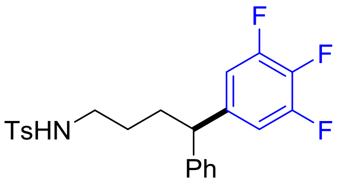

4-methyl-N-(4-phenyl-4-(3,4,5-trifluorophenyl)butyl)benzenesulfonamide (31)

Prepared following general procedure B using substrate (64.2 mg, 0.2 mmol). Adjust reaction tempreture to 25 ℃, after 18 hours, the reaction mixture was purified by column chromatography using 5% hexane in DCM to provide the title compound (47.6 mg, 55% yield) as a yellow oil. ^{1}H NMR (600 MHz, $CDCl_3$) δ 7.72 (d, J = 8.2 Hz, 2H), 7.31 – 7.24 (m, 4H), 7.24 – 7.18 (m, 1H), 7.10 (d, J = 7.3 Hz, 2H), 6.79 – 6.72 (m, 2H), 4.71 (t, J = 6.2 Hz, 1H), 3.73 (t, J = 7.9 Hz, 1H), 2.95 (ddd, J = 13.1, 6.6, 2.1 Hz, 2H), 2.42 (s, 3H), 2.00 – 1.87 (m, 2H), 1.42 – 1.33 (m, 2H). ^{19}F NMR (565 MHz, $CDCl_3$) δ -134.32 (dd, J = 20.6, 8.7 Hz, 2F), -162.65 – -165.19 (m, 1F). ^{13}C NMR (151 MHz, $CDCl_3$) δ 151.17 (ddd, J = 249.8, 9.8, 4.0 Hz), 143.65 (s), 142.78 (s), 141.19 (dd, J = 11.1, 6.5 Hz), 138.29 (dt, J = 250.2, 15.4 Hz), 137.01 (s), 129.85 (s), 128.95 (s), 127.71 (s), 127.17 (s), 127.06 (s), 111.70 (dd, J = 16.6, 4.2 Hz), 50.11 (s), 43.00 (s), 32.22 (s), 27.95 (s), 21.60 (s). IR (cm$^-$): 3276, 3028, 2928, 2868, 2360, 2341, 1617, 1598, 1526, 1494, 1444, 1321, 1305, 1234, 1155, 1092, 1038, 907, 813.

HRMS (ESI) calcd. For $[M+Na]^+$ 456.1216, found:456.1209.
R_f = 0.45 (DCM).

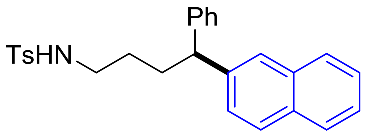

4-methyl-N-(4-(naphthalen-2-yl)-4-phenylbutyl)benzenesulfonamide (32)
Prepared following general procedure B using substrate (64.2 mg, 0.2 mmol). After 16 hours, the reaction mixture was purified by column chromatography using 5% hexane in DCM to provide the title compound (60.9 mg, 71% yield) as a colorless oil. ^{1}H NMR (600 MHz, $CDCl_3$) δ 7.78 (dd, J = 8.1, 2.8 Hz, 2H), 7.72 (d, J = 8.5 Hz, 1H), 7.71 – 7.68 (m, 2H), 7.64 (s, 1H), 7.48 – 7.40 (m, 2H), 7.26 (ddd, J = 5.3, 3.2, 1.6 Hz, 3H), 7.22 (t, J = 7.8 Hz, 4H), 7.19 – 7.15 (m, 1H), 4.46 (t, J = 6.2 Hz, 1H), 3.96 (t, J = 7.8 Hz, 1H), 2.98 (q, J = 6.9 Hz, 2H), 2.38 (s, 3H), 2.18 – 2.04 (m, 2H), 1.50 – 1.39 (m, 2H). ^{13}C NMR (151 MHz, $CDCl_3$) δ 144.45 (s), 143.46 (s), 141.98 (s), 137.07 (s), 133.62 (s), 132.32 (s), 129.79 (s), 128.64 (s), 128.31 (s), 127.97 (s), 127.84 (s), 127.69 (s), 127.18 (s), 126.63 (s), 126.46 (s), 126.17 (s), 125.95 (s), 125.63 (s), 50.91 (s), 43.27 (s), 32.36 (s), 28.21 (s), 21.60 (s). IR (cm^-): 3277, 3055, 3024, 2939, 2865, 2360, 1597, 1493, 1450, 1420, 1321, 1304, 1155, 1092, 1018, 965, 907, 858, 813.
HRMS (ESI) calcd. For $[M+Na]^+$ 452.1655, found: 452.1659.
R_f = 0.45 (DCM).

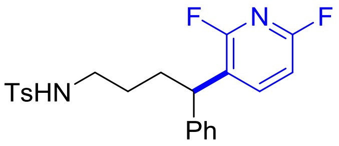

N-(4-(2,6-difluoropyridin-3-yl)-4-phenylbutyl)-4-methylbenzenesulfonamide (33)
Prepared following general procedure B using substrate (64.2 mg, 0.2 mmol). After 16 hours, the reaction mixture was purified by column chromatography using 2% Et_2O in DCM to provide the title compound (60.0 mg, 72% yield) as a light yellow oil. ^{1}H NMR (600 MHz, $CDCl_3$) δ 7.70 (dd, J = 16.1, 8.2 Hz, 3H), 7.29 – 7.24 (m, 4H), 7.22 – 7.17 (m, 1H), 7.17 – 7.11 (m, 2H), 6.72 (dd, J = 8.1, 2.5 Hz, 1H), 5.00 (t, J = 6.2 Hz, 1H), 4.04 (t, J = 7.9 Hz, 1H), 2.94 (q, J = 6.6 Hz, 2H), 2.41 (s, 3H), 2.12 – 1.90 (m, 2H), 1.51 – 1.35 (m, 2H). ^{19}F NMR (565 MHz, $CDCl_3$) δ -71.81 – -71.95 (m, 2F). ^{13}C NMR (151 MHz, $CDCl_3$) δ 159.81 (dd, J = 245.9, 14.9 Hz), 158.73 (dd, J = 246.4, 14.3 Hz), 143.53 (s), 143.21 (dd, J = 7.0, 5.8 Hz), 141.71 (s), 136.83 (s), 129.75 (s), 128.81 (s), 127.69 (s), 127.04 (s), 126.99 (s), 123.69 – 121.10 (m), 106.18 (dd, J = 33.4, 6.4 Hz), 42.89 (s), 42.71 (s), 31.35 (s), 27.75 (s), 21.51 (s). IR (cm^-): 3276, 2943, 2869, 2360, 2341, 1607, 1465, 1408, 1321, 1309, 1298, 1154, 1093, 992, 903, 814.
HRMS (ESI) calcd. For $[M+Na]^+$ 439.1262, found:439.1255.
R_f = 0.25 (DCM).

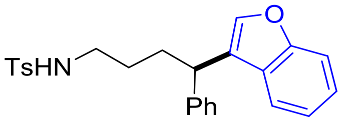

N-(4-(benzofuran-3-yl)-4-phenylbutyl)-4-methylbenzenesulfonamide (34)
Prepared following general procedure B using substrate (64.2 mg, 0.2 mmol). After 16 hours, the reaction mixture was purified by column chromatography using 5% hexane in DCM to provide the title compound (61.2 mg, 73% yield) as a light yellow oil. ^{1}H NMR (600 MHz, $CDCl_3$) δ 7.72 (d, J = 8.3 Hz, 2H), 7.46 – 7.39 (m, 2H), 7.31 – 7.16 (m, 9H), 7.12 – 7.07 (m, 1H), 4.61 (t, J = 6.1 Hz, 1H), 3.93 (dd, J = 16.4, 9.2 Hz, 1H), 3.00 – 2.92 (m, 2H), 2.40 (s, 3H), 2.14 (ddt, J = 13.2, 10.5, 5.9 Hz, 1H), 1.99 – 1.88 (m, 1H), 1.55 – 1.38 (m, 2H). ^{13}C NMR (101 MHz, $CDCl_3$) δ 155.64 (s), 143.53 (s), 142.90 (s), 141.21 (s), 137.03 (s), 129.82 (s), 128.69 (s), 127.91 (s), 127.50 (s), 127.18 (s), 126.77 (s), 124.32 (s), 123.75 (s), 122.42 (s), 120.37 (s), 111.53 (s), 43.16 (s), 41.54 (s), 32.16 (s), 27.91 (s), 21.60 (s). IR (cm^-): 3275, 3027, 2927, 2866, 1598, 1493, 1452, 1420, 1321, 1304, 1156, 1092, 963, 907, 857, 813.
HRMS (ESI) calcd. For $[M+Na]^+$ 442.1447, found: 442.1449.
R_f = 0.45 (DCM).

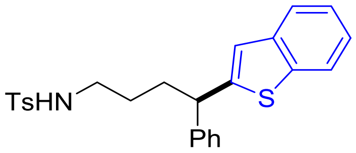

N-(4-(benzo[b]thiophen-2-yl)-4-phenylbutyl)-4-methylbenzenesulfonamide (35)

Prepared following general procedure B using substrate (64.2 mg, 0.2 mmol). Adjust the $Cu(OTf)_2$ loading to 10 mol %, Ligand 15 mol%, after 48 hours, the reaction mixture was purified by column chromatography using 5% hexane in DCM to provide the title compound (56.5 mg, 65% yield) as a colorless oil. ^{1}H NMR (600 MHz, $CDCl_3$) δ 8.01 – 7.95 (m, 3H), 7.92 (d, J = 7.8 Hz, 1H), 7.59 – 7.54 (m, 3H), 7.53 – 7.46 (m, 7H), 4.77 (t, J = 6.2 Hz, 1H), 4.35 (t, J = 7.7 Hz, 1H), 3.24 (q, J = 6.8 Hz, 2H), 2.65 (s, 3H), 2.42 (dddd, J = 13.0, 10.2, 7.5, 5.5 Hz, 1H), 2.32 (dddd, J = 13.6, 10.1, 8.2, 5.5 Hz, 1H), 1.82 – 1.67 (m, 2H). ^{13}C NMR (151 MHz, $CDCl_3$) δ 149.75 (s), 143.52 (s), 143.32 (s), 139.88 (s), 139.46 (s), 137.04 (s), 129.83 (s), 128.80 (s), 127.77 (s), 127.18 (s), 127.07 (s), 124.30 (s), 123.87 (s), 123.22 (s), 122.27 (s), 120.52 (s), 47.14 (s), 43.09 (s), 33.60 (s), 28.06 (s), 21.62 (s). IR (cm^-): 3277, 3058, 3026, 2923, 2863, 2360, 2342, 1598, 1493, 1452, 1435, 1322, 1304, 1155, 1093, 907, 813.
HRMS (ESI) calcd. For $[M+Na]^+$ 458.1219, found:458.1215.
R_f = 0.45 (DCM).

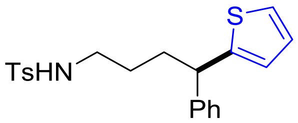

4-methyl-N-(4-phenyl-4-(thiophen-2-yl)butyl)benzenesulfonamide (36)

Prepared following general procedure B using substrate (64.2 mg, 0.2 mmol). Adjust the $Cu(OTf)_2$ loading to 10 mol %, Ligand 15 mol%, after 48 hours, the reaction mixture was purified by prepare plate using DCM to provide the title compound as a white powder.

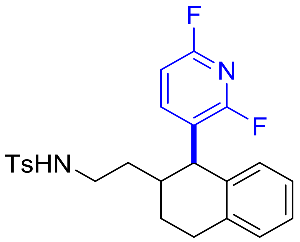

N-(2-1-(2,6-difluoropyridin-3-yl)-1,2,3,4-tetrahydronaphthalen-2-yl)ethyl)-4-methylbenzenesulfonamide (38)

Prepared following general procedure B using substrate (65.8 mg, 0.2 mmol). After 16 hours, the reaction mixture was purified by column chromatography using 2% Et_2O in DCM to provide the title compound (39.8 mg, 45% yield) as a light yellow oil. ^{1}H NMR (600 MHz, $CDCl_3$) δ 7.70 (d, J = 8.3 Hz, 2H), 7.33 – 7.24 (m, 3H), 7.18 – 7.09 (m, 2H), 7.07 – 7.00 (m, 1H), 6.71 – 6.65 (m, 2H), 4.63 (t, J = 6.2 Hz, 1H), 4.00 (d, J = 7.0 Hz, 1H), 3.06 – 2.96 (m, 2H), 2.87 – 2.75 (m, 2H), 2.42 (s, 3H), 1.96 – 1.89 (m, 1H), 1.89 – 1.82 (m, 1H), 1.54 – 1.40 (m, 3H). ^{19}F NMR (565 MHz, $CDCl_3$) δ -71.40 – -71.49 (m, 1F), -73.24 (d, J = 9.0 Hz, 1F). ^{13}C NMR (151 MHz, $CDCl_3$) δ 159.95 (dd, J = 245.7, 14.3 Hz), 159.04 (dd, J = 245.3, 13.8 Hz), 145.98 – 144.94 (m), 143.64 (s), 137.03 (s), 136.90 (s), 136.04 (s), 130.09 (s), 129.86 (s), 129.21 (s), 127.13 (s), 126.76 (s), 126.46 (s), 124.47 (dd, J = 26.5, 5.8 Hz), 106.40 (dd, J = 34.3, 5.5 Hz), 43.21 (s), 41.01 (s), 37.49 (s), 33.04 (s), 27.28 (s), 24.99 (s), 21.62 (s). IR (cm^-): 3277, 2926, 2360, 2342, 2255, 1606, 1589, 1463, 1406, 1320, 1304, 1288, 1259, 1155, 1092, 992, 907, 814.
HRMS (ESI) calcd. For $[M+Na]^+$ 465.1419, found:465.1414.
R_f = 0.25 (DCM).

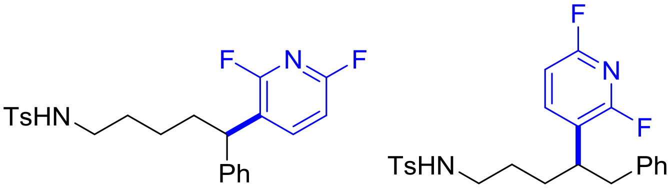

Prepared following general procedure B using substrate (67 mg. 0.2 mmol). After 30 hours, the reaction mixture was purified by column chromatography using 70% DCM in hexanes to 2% Et2O in hexanes to give the title compound as a yellow oil as an inseperable mixture of regioisomers (56.3 mg, 0.13 mmol 65%).

N-(5-(2,6-difluoropyridin-3-yl)-5-phenylpentyl)-4-methylbenzenesulfonamide (39, major)

^{1}H NMR (600 MHz, $CDCl_3$) δ 7.73 – 7.66 (m, 3H), 7.31 – 7.24 (m, 4H), 7.22 – 7.19 (m, 1H), 7.19 – 7.16 (m, 2H), 6.74 (dd, *J* = 8.1, 2.1 Hz, 1H), 4.59 – 4.48 (m, 1H), 4.06 (t, *J* = 7.8, 1H), 2.93 – 2.82 (m, 2H), 2.40 (s, 3H), 2.03-1.86 (m, 2H), 1.52 – 1.42 (m, 2H), 1.28 – 1.19 (m, 2H).

N-(4-(2,6-difluoropyridin-3-yl)-5-phenylpentyl)-4-methylbenzenesulfonamide (39, minor)

^{1}H NMR (600 MHz, $CDCl_3$) δ 7.73 – 7.66 (m, 2H), 7.50 (dd, *J* = 16.9, 8.0, 1H) 7.31 – 7.24 (m, 4H), 7.23 –7.19 (m, 1H), 7.19 – 7.16 (m, 2H), 6.67 (dd, *J* = 8.0, 2.8 Hz, 1H), 4.59 – 4.48 (m, 1H), 3.10 –3.00 (m, 1H), 2.93 – 2.82 (m, 2H), 2.84 – 2.73 (m, 1H), 2.40 (s, 3H), 1.75 – 1.55 (m, 2H), 1.37 – 1.19 (m, 2H).

^{13}C NMR (151 MHz, $CDCl_3$) δ 159.86 (dd, *J* = 245.5, 14.5 Hz), 159.21 (dd, *J* = 244.6, 13.2 Hz), 158.85 (dd, *J* = 246.5, 14.4 Hz), 143.97 – 143.82 (m), 143.65 (s), 143.59 (s), 143.29 (t, *J* = 6.4 Hz), 142.05 (s), 138.99 (s), 137.01 (s), 129.85 (s), 129.00 (s), 128.93 (s), 128.51 (s), 127.83 (s), 127.18 (s), 127.15 (s), 127.09 (s), 126.51 (s), 123.40 (dd, *J* = 25.1, 7.7 Hz), 122.06 (dd, *J* = 27.0, 5.8 Hz), 106.48 – 105.96 (m), 43.11 (s), 43.03 (s), 42.98 (s), 41.67 (s), 40.23 (s), 34.00 (s), 31.13 (s), 29.83 (s), 29.49 (s), 27.70 (s), 24.80 (s), 21.63 (s).^{19}F NMR (565 MHz, $CDCl_3$) δ -71.59 – -71.73 (m), -71.91 – -72.01 (m), -72.02 – -72.09 (m). IR (flim) cm^{-1} 3287, 3068, 3025, 2946, 2865, 1617, 1454, 1164.
HRMS (ESI-TOF) *m/z* calcd for $[M+Na]^+$ 452.1424 found 453.1410.
R_f = 0.5 (DCM)

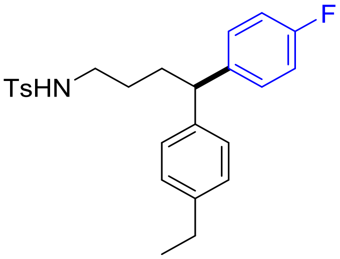

N-(4-(4-ethylphenyl)-4-(4-fluorophenyl)butyl)-4-methylbenzenesulfonamide (40)

Prepared following general procedure B using substrate (66.2 mg, 0.2 mmol). After 16 hours, the reaction mixture was purified by column chromatography using 10% hexane in DCM to provide the title compound (51.9 mg, 61% yield) as a colorless oil. ^{1}H NMR (600 MHz, $CDCl_3$) δ 7.73 – 7.68 (m, 2H), 7.27 (dt, *J* = 3.8, 1.9 Hz, 2H), 7.10 (ddd, *J* = 14.8, 6.7, 4.7 Hz, 4H), 7.06 – 7.03 (m, 2H), 6.95 – 6.90 (m, 2H), 4.32 (t, *J* = 6.2 Hz, 1H), 3.75 (t, *J* = 7.9 Hz, 1H), 2.94 (q, *J* = 6.8 Hz, 2H), 2.60 (q, *J* = 7.6 Hz, 2H), 2.42 (s, 3H), 2.03 – 1.89 (m, 2H), 1.44 – 1.36 (m, 2H), 1.20 (t, *J* = 7.6 Hz, 3H). ^{19}F NMR (565 MHz, $CDCl_3$) δ -117.08 – -117.16 (m). ^{13}C NMR (151 MHz, $CDCl_3$) δ 161.45 (d, *J* = 244.3 Hz), 143.53 (s), 142.42 (s), 141.56 (s), 140.59 (d, *J* = 3.2 Hz), 137.10 (s), 129.83 (s), 129.18 (d, *J* = 7.8 Hz), 128.17 (s), 127.64 (s), 127.21 (s), 115.34 (d, *J* = 21.2 Hz), 49.77 (s), 43.24 (s), 32.82 (s), 28.49 (s), 28.22 (s), 21.65 (s), 15.57 (s). IR (cm^-): 3277, 2963, 2927, 2870, 2360, 2341, 2256, 1599, 1506, 1456, 1415, 1321, 1305, 1221, 1156, 1094, 906, 813.
HRMS (ESI) calcd. For $[M+Na]^+$ 448.1717, found:448.1712.
R_f = 0.45 (DCM).

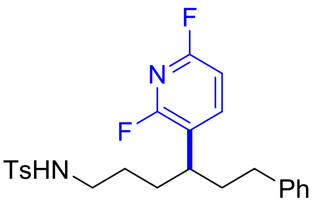

N-(4-(2,6-difluoropyridin-3-yl)-6-phenylhexyl)-4-methylbenzenesulfonamide (42)

Prepared following general procedure B using substrate (66.2 mg, 0.2 mmol). After 16 hours, the reaction mixture was purified by column chromatography using 2% Et_2O in DCM to provide the title compound (48.0 mg, 54% yield) as a light yellow oil. ^{1}H NMR (600 MHz, $CDCl_3$) δ 7.70 (d, *J* = 8.3 Hz, 2H), 7.63 (dd, *J* = 16.9, 8.0 Hz, 1H), 7.26 (ddt, *J* = 14.9, 13.3, 7.4 Hz, 4H), 7.16 (t, *J* = 7.4 Hz, 1H), 7.07 – 7.02 (m, 2H), 6.76 (dd, *J* = 8.1, 2.8 Hz, 1H), 4.76 (t, *J* = 6.2 Hz, 1H), 2.87 (q, *J* = 6.7 Hz, 2H), 2.82 – 2.75 (m, 1H), 2.44 (ddd, *J* = 12.1, 11.0, 6.0 Hz, 2H), 2.41 (s, 3H), 1.97 – 1.79 (m, 2H), 1.71 – 1.63 (m, 1H), 1.60 – 1.50 (m, 1H), 1.41 – 1.28 (m, 1H), 1.28 – 1.17 (m, 1H). ^{19}F NMR (565 MHz, $CDCl_3$) δ -71.70 – -71.79 (m, 1F), -72.26 (t, *J* = 10.0 Hz, 1F). ^{13}C NMR (151 MHz, $CDCl_3$) δ 159.85 (dd, *J* = 244.8, 14.1 Hz), 159.35 (dd, *J* = 245.2, 13.5 Hz), 143.68 (t, *J* = 6.2 Hz), 143.62 (s), 141.42 (s), 136.98 (s), 129.83 (s), 128.53 (s), 128.31 (s), 127.12 (s), 126.10 (s), 122.54 (dd, *J* = 27.1, 5.8 Hz), 106.42 (dd, *J* = 34.3, 5.6 Hz), 43.02 (d, *J* = 7.1 Hz), 37.56 (d, *J* = 1.8 Hz), 36.84 (s), 33.63 (s), 32.28 (s), 27.50 (s), 21.59 (s). IR (cm$^-$): 3278, 3026, 2936, 2861, 2360, 2341, 1608, 1495, 1466, 1408, 1322, 1303, 1155, 1092, 991, 908, 814.
HRMS (ESI) calcd. For $[M+Na]^+$ 467.1575, found:467.1569.
R_f = 0.25 (DCM).

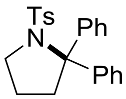

2,2-diphenyl-1-tosylpyrrolidine (43)

Prepared according to literature with modifications and matches reported compound.[3] To substrate **10** (38 mg, 0.1 mmol, 1 equiv), PhI(*m*CBA)$_2$ (51.5 mg, 0.1 mmol, 1 equiv), and I_2 (12.7 mg, 0.05 mmol, 0.5 equiv) was added degassed 1,2-dichloroethane (0.15 M) under a nitrogen atmosphere. The mixture was stirred and irradiated using two 23 W compact fluorescent light bulbs at room temperature for 6 hours. The solvent was removed *in vacuo* and the crude mixture was purified by column chromatography eluting with 10% EtOAc in hexanes to give the product as a white solid. (28.1 mg, 0.070 mmol, 70%). ^{1}H NMR (600 MHz, $CDCl_3$) δ 7.36-7.21 (m, 4H), 7.27-7.22 (m, 6H), 6.98 (d, *J* = 8.1 Hz, 2H), 6.87 (d, *J* = 8.1 Hz, 2H), 3.82 (t, *J* = 6.8 Hz, 2H), 2.60 (t, *J* = 6.7 Hz, 2H), (2.35 (s, 3H), 1.84 (p, *J* = 6.7 Hz, 2H). IR (flim) cm^{-1} 3073, 2924, 2869, 1340, 1154, 1092. ^{13}C NMR (150 MHz, $CDCl_3$) 142.84 (s), 142.15 (s), 138.31 (s), 129.55 (s), 128.86 (s), 127.58 (s), 127.13 (s), 126.71 (s), 76.11 (s), 50.54 (s), 46.24 (s), 22.78 (s).
R_f = 0.2 (10% EtOAc in hexanes).

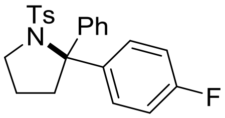

2-(4-fluorophenyl)-2-phenyl-1-tosylpyrrolidine (45)

Prepared according to literature with modifications.[3] To substrate **1** (10 mg, 0.025 mmol, 1 equiv), PhI(*m*CBA)$_2$ (12.9 mg, 0.025 mmol, 1 equiv), and I_2 (3.19 mg, 0.0125 mmol, 0.5 equiv) was added to degassed 1,2-dichloroethane (0.1 M) under a nitrogen atmosphere. The mixture was stirred and irradiated using two 23 W compact fluorescent light bulbs at room temperature for 6 hours. The solvent was removed *in vacuo* and the crude mixture was purified by column chromatography eluting with 20% EtOAc in hexanes to give the product as a yellow solid (7.3 mg, 0.068 mmol, 74%). ^{1}H NMR (600 MHz, $CDCl_3$) δ 7.35-7.30 (m, 2H), 7.30-7.26 (m, 5H), 7.01 (d, J = 8.0 Hz, 2H), 6.93-6.88 (m, 4H), 3.87-3.78 (m, 2H), 2.57 (t, J = 6.7 Hz, 2H), 2.35 (s, 3H), 1.83 (p, J = 6.6 Hz, 2H). ^{19}F NMR (565 MHz, $CDCl_3$) δ -115.92 (tt, *J* = 8.4, 5.3) ^{13}C NMR (151 MHz, $CDCl_3$) δ 142.86 (s), 142.36 (s), 138.39 (s), 131.43, 131.38 (s), 129.30 (s), 128.93 (s), 127.72 (s), 127.27 (s), 126.62 (s), 114.33 (s), 114.19 (s), 75.55 (s), 50.60 (s), 46.24 (s), 22.65 (s), 21.56 (s). IR (flim) cm^{-1}. 2923, 2853, 1508, 1339, 1157, 1095. HRMS (ESI) calcd. For $[M+Na]^+$ 418.1253, found: 418.1246.
R_f = 0.2 (10% EtOAc in hexanes).

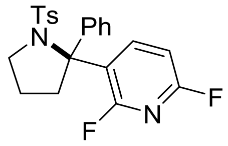

2,6-difluoro-3-(2-phenyl-1-tosylpyrrolidin-2-yl)pyridine (46)

Prepared according to literature with modifications.[4] To substrate **33** (60 mg, 0.144 mmol, 1 equiv), $PhI(OAc)_2$ (187 mg, 0.576 mmol, 4 equiv), and NaI (86.3 mg, 0.567 mmol, 4 equiv) was added dry, degassed MeCN (0.1 M) under a nitrogen atmosphere. The mixture was stirred and irradiated using two 23 W compact fluorescent light bulbs at room temperature for 6 hours. The solvent was removed *in vacuo* and the crude mixture was purified by column chromatography eluting with 20% EtOAc in hexanes to give the product as a yellow solid (29.4 mg, 0.068 mmol, 49%). ^{1}H NMR (600 MHz, $CDCl_3$) δ 8.29 (dt, *J* = 9.8, 8.0 Hz, 1H), 7.33 – 7.26 (m, 2H), 7.24 – 7.13 (m, 3H), 7.03 (d, *J* = 7.9 Hz, 2H), 6.90 (dd, *J* = 8.2, 2.9 Hz, 1H), 6.87 (d, *J* = 8.3 Hz, 2H), 3.92 (ddd, *J* = 9.0, 7.3, 5.5 Hz, 1H), 3.78 (dt, *J* = 9.0, 7.1 Hz, 1H), 2.70 – 2.65 (m, 2H), 2.37 (s, 3H), 1.98 – 1.89 (m, 1H), 1.87 – 1.77 (m, 1H). ^{19}F NMR (565 MHz, $CDCl_3$) δ -57.37 (t, *J* = 9.9 Hz), -71.08 – -71.28 (m).^{13}C NMR (151 MHz, $CDCl_3$) δ 160.95 (dd, *J* = 247.1, 14.3 Hz), 157.67 (dd, *J* = 248.0, 14.2 Hz), 147.39 – 147.22 (m), 142.97 (s), 139.82 (s), 137.40 (s), 129.16 (s), 127.98 (s), 127.81 (s), 126.52 (s), 123.11 (dd, *J* = 22.3, 5.8 Hz), 105.51 (dd, *J* = 33.9, 5.4 Hz), 72.84 (d, *J* = 6.1 Hz), 50.60 (s), 44.05 (d, *J* = 4.2 Hz), 23.12 (s), 21.60 (s).IR (flim) cm^{-1} 3103, 3054, 2931, 2862, 1731, 1609, 1354, 1169, 1006. HRMS (ESI) calcd. For $[M+Na]^+$ 415.1292, found: 415.0142.
$R_f = 0.3$ (20% EtOAc in hexanes).

IV. References

S1. Wang D.; Wu, L.; Wang, F.; Wan, X.; Chen, P.; Lin, Z.; Liu, G. *J. Am. Chem. Soc*. **2017**, *139*, 6811.

S2. Patel, H. H.; Sigman, M. S. *J. Am. Chem. Soc*. **2016**, *138*, 14226.

S3. Martinez, C.; Muniz, K, *ACS Catalysis.* **2015**, *54*, 8297.

S4. Wappes, E. A.; Fosu, S. C.; Chopko, T. C.; Nagib, D. A. *Angew. Chem. Int. Ed.* **2016**, *55*, 10257.

V. NMR Spectra for new compounds

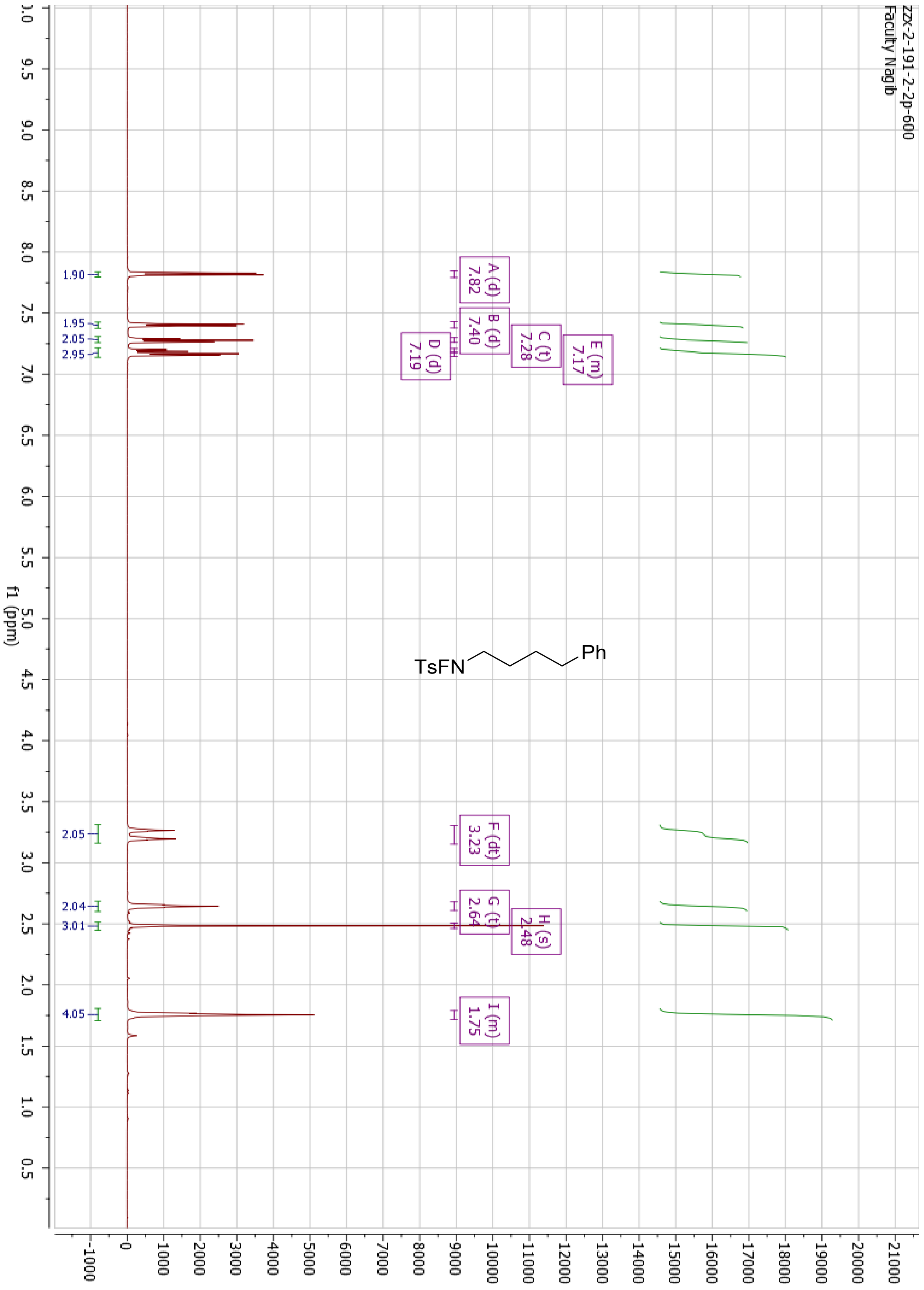

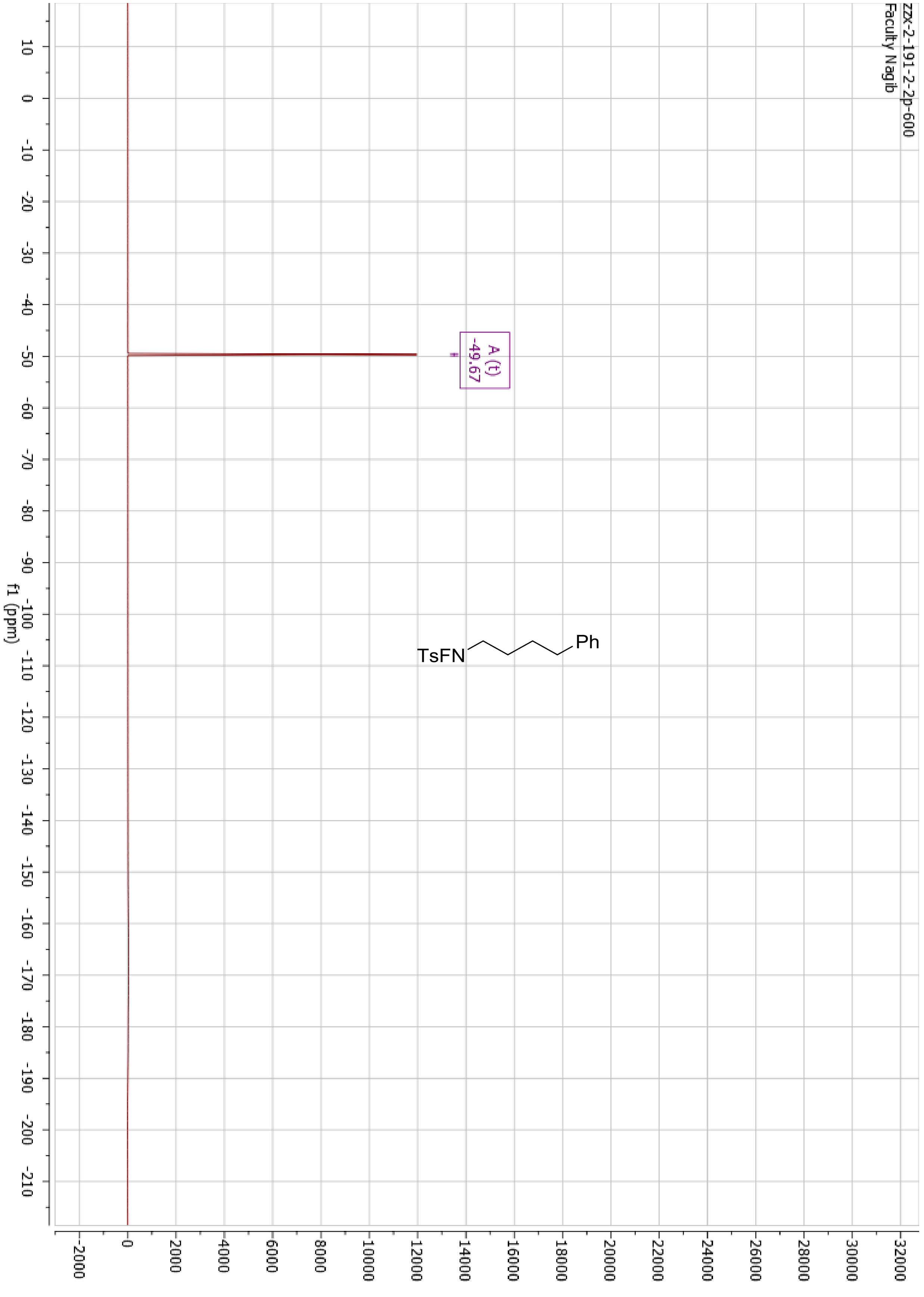
zzx-2-191-2-2p-600
Faculty Nagib
A (t)
-49.67
TsFN
Ph
f1 (ppm)

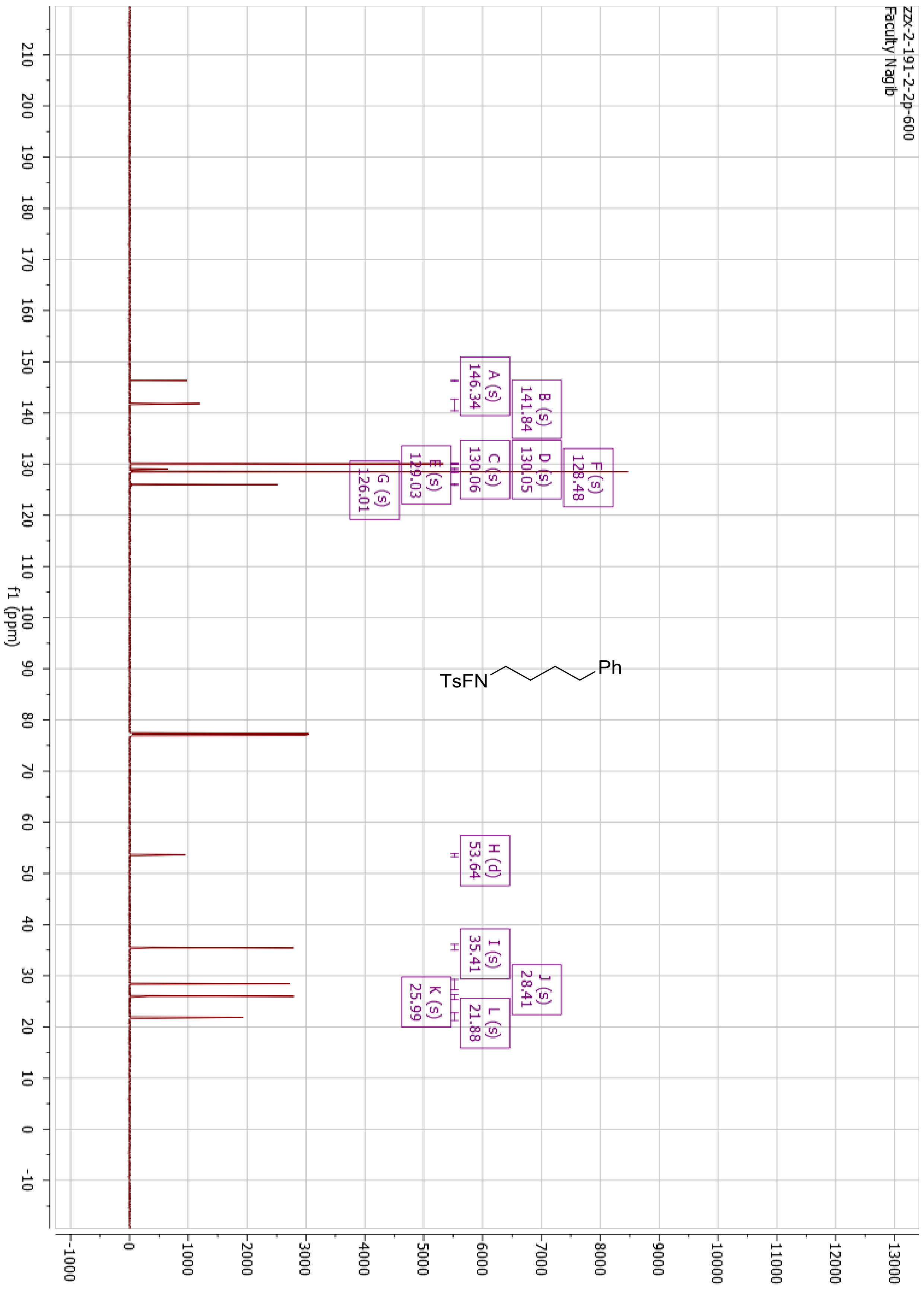

ZZX-2-191-2-2p-600
Faculty Nagib
A (s) 146.34
B (s) 141.84
C (s) 130.06
D (s) 130.05
E (s) 129.03
F (s) 128.48
G (s) 126.01
H (d) 53.64
I (s) 35.41
J (s) 28.41
K (s) 25.99
L (s) 21.88
TsFN
Ph
f1 (ppm)

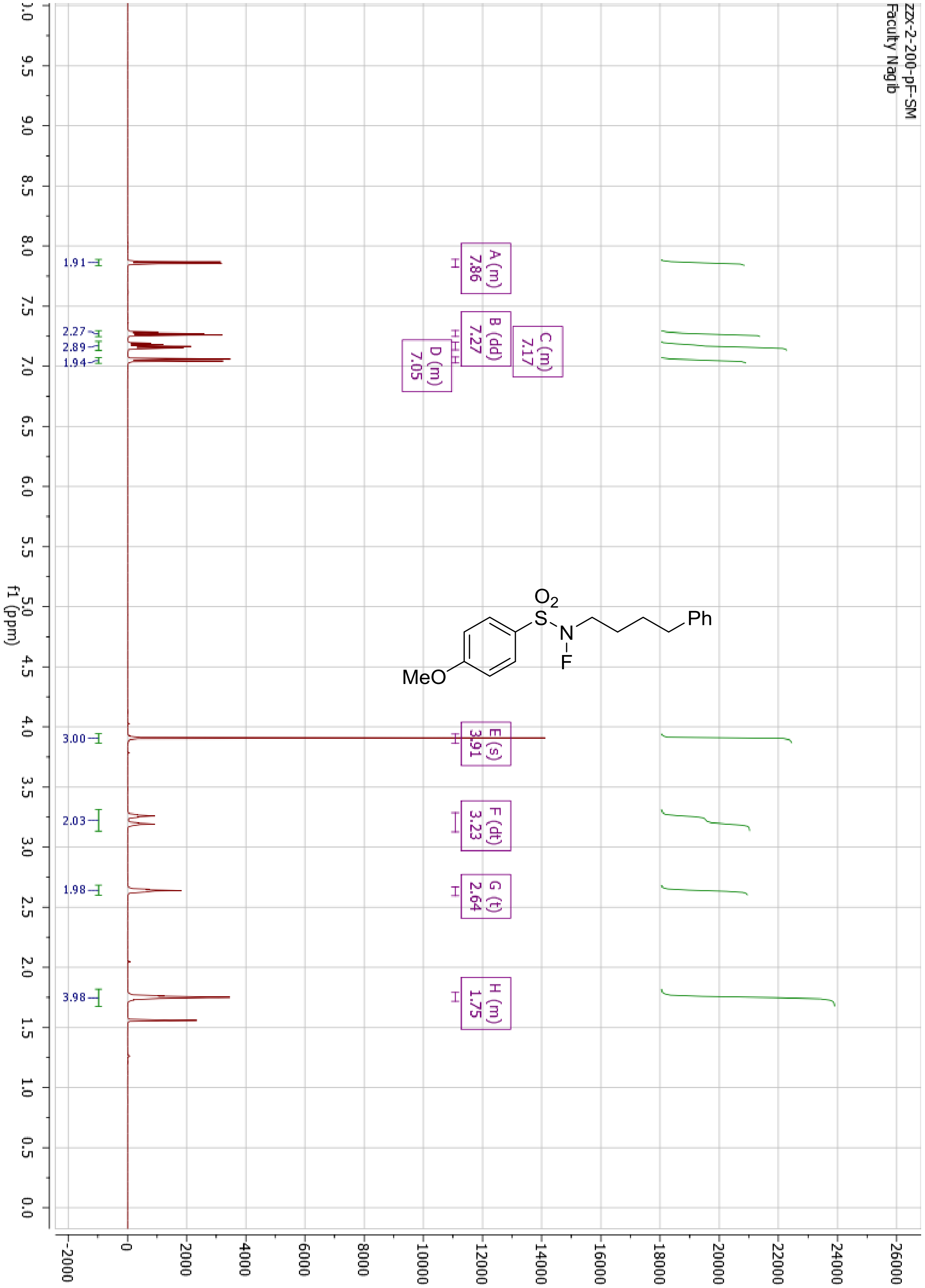
zzx-2-200-pF-SM
Faculty Nagib
A (m) 7.86
B (dd) 7.27
C (m) 7.17
D (m) 7.05
E (s) 3.91
F (dt) 3.23
G (t) 2.64
H (m) 1.75
1.91
2.27
2.89
1.94
3.00
2.03
1.98
3.98
MeO
O_2
S
N
F
Ph
f1 (ppm)

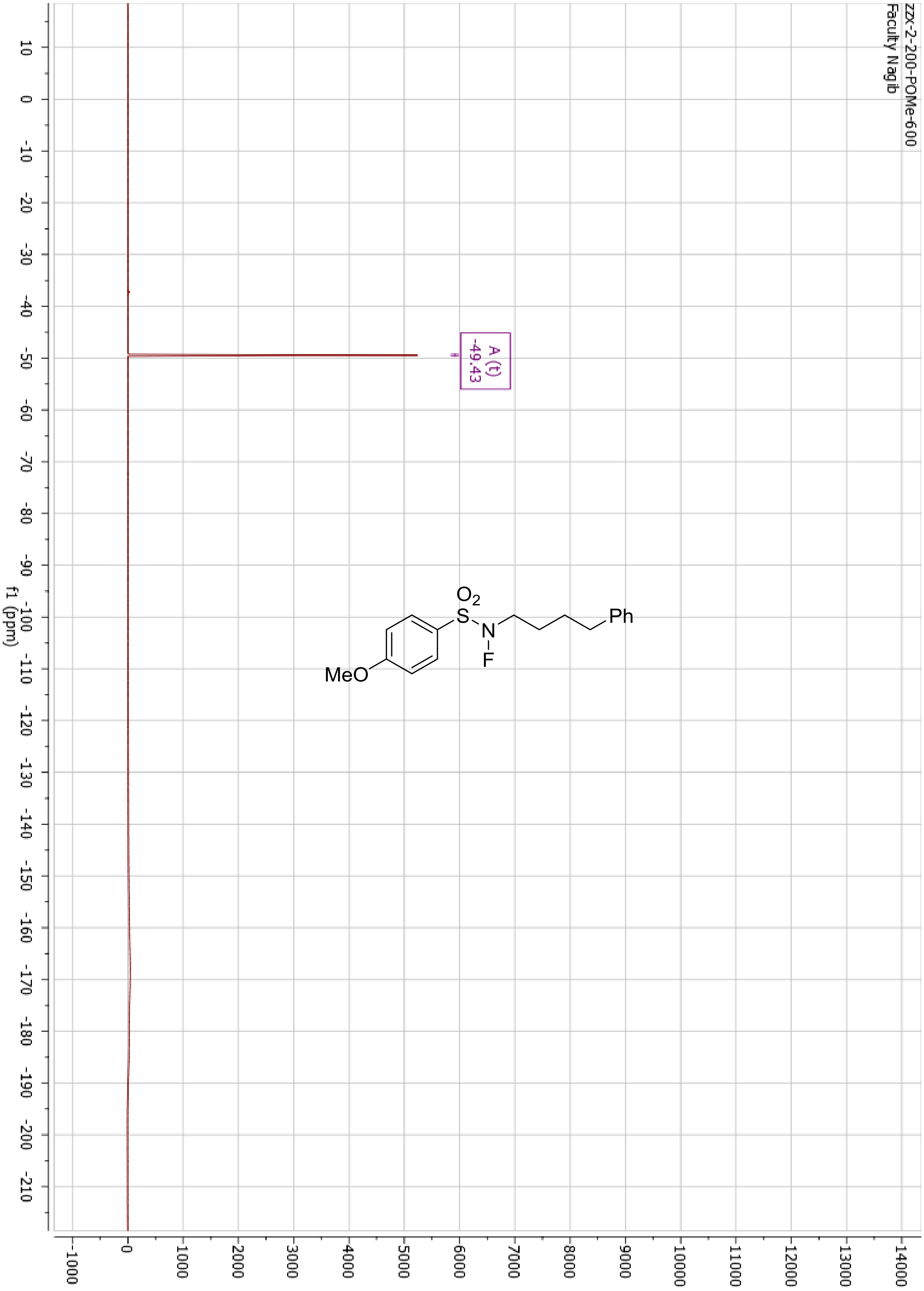
zzx-2-200-POMe-600
Faculty Nagib
A (t)
-49.43
f1 (ppm)
MeO
O_2
S
N
F
Ph

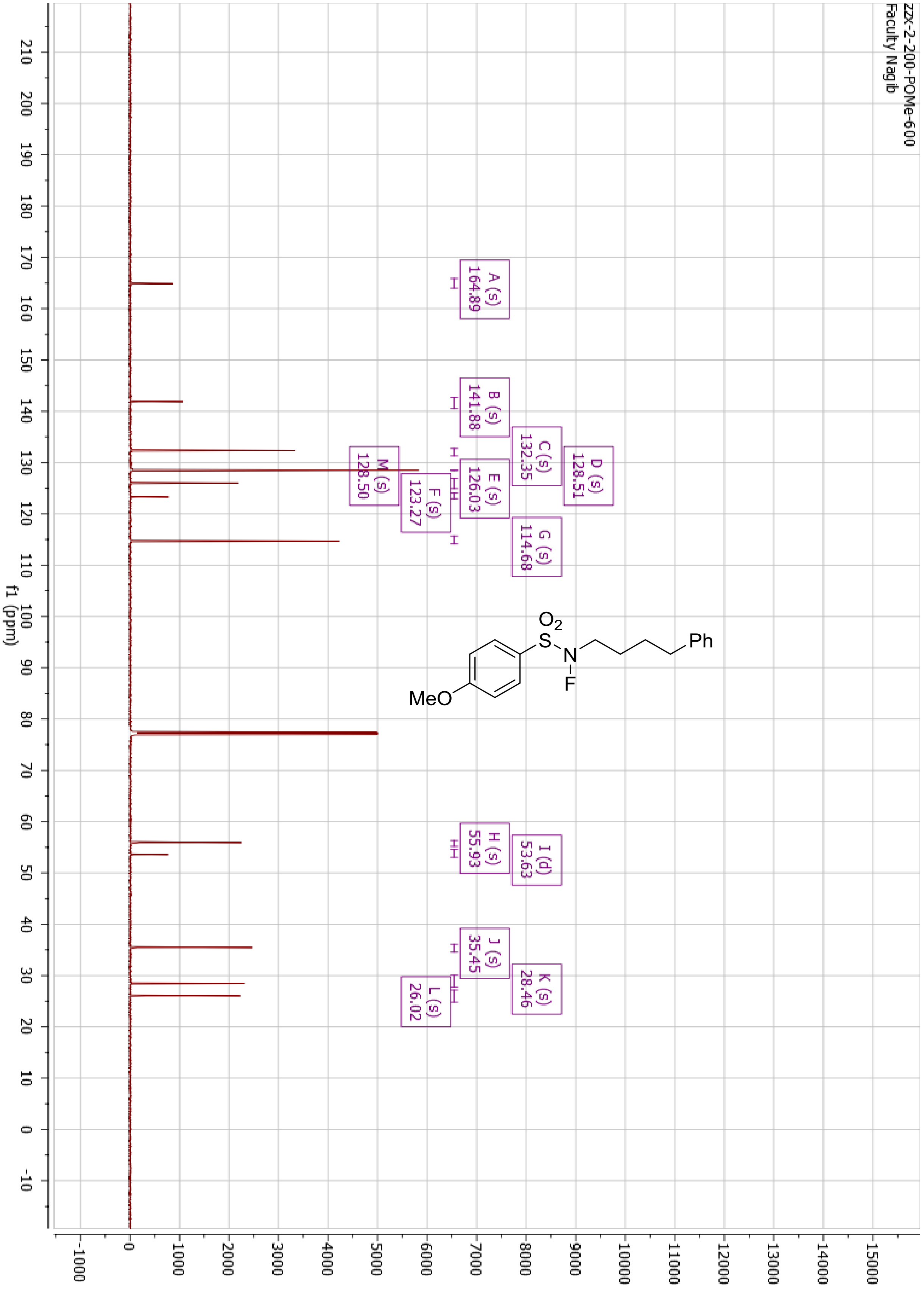
ZZX-2-200-POMe-600
Faculty Nagib
A (s) 164.89
B (s) 141.88
C (s) 132.35
D (s) 128.51
M (s) 128.50
E (s) 126.03
F (s) 123.27
G (s) 114.68
H (s) 55.93
I (d) 53.63
J (s) 35.45
K (s) 28.46
L (s) 26.02
MeO
O2
S
N
F
Ph
f1 (ppm)

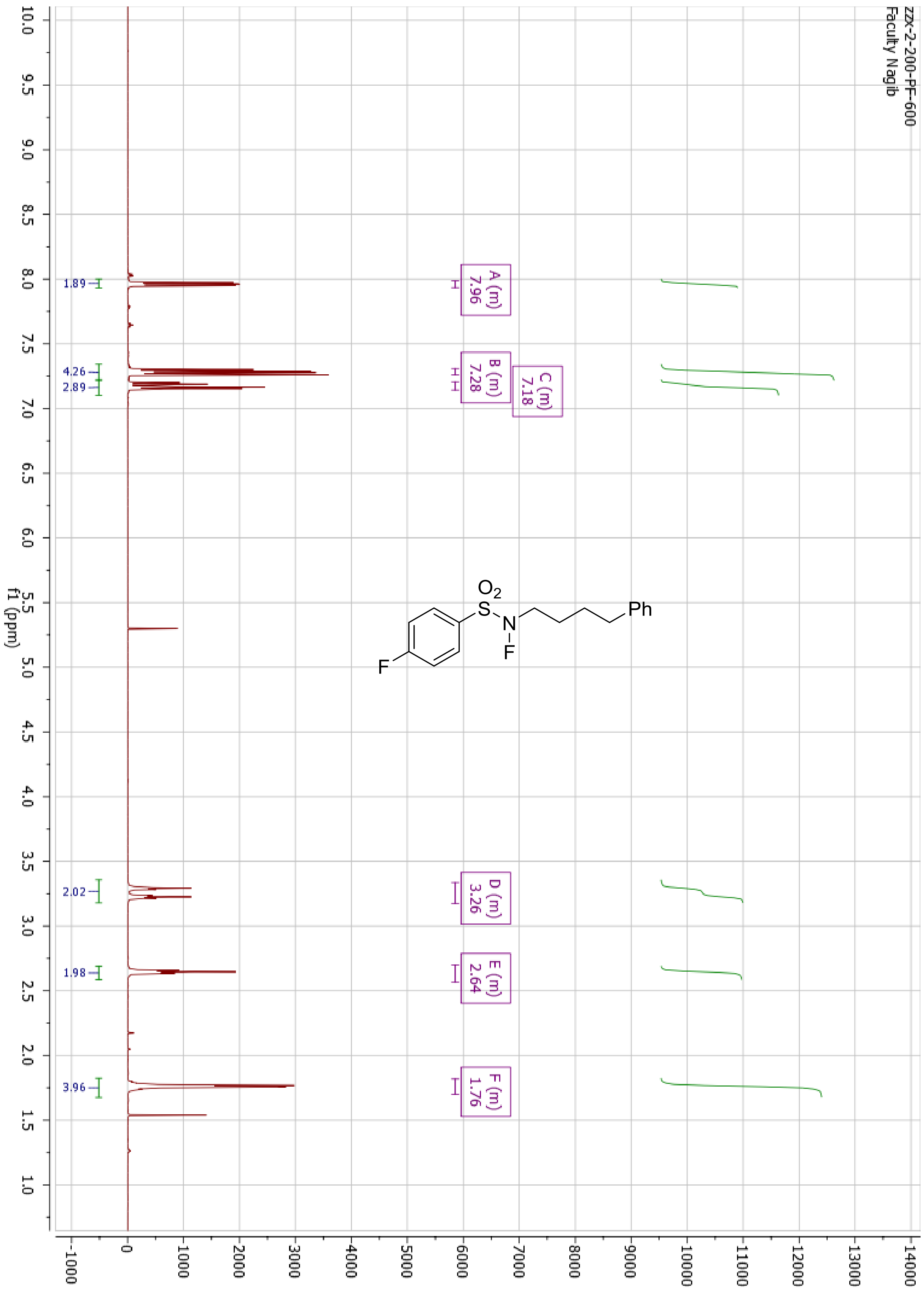
zzx-2-200-PF-600
Faculty Nagib
A (m) 7.96
B (m) 7.28
C (m) 7.18
D (m) 3.26
E (m) 2.64
F (m) 1.76
1.89
4.26
2.89
2.02
1.98
3.96
O2
S
N
F
F
Ph
f1 (ppm)

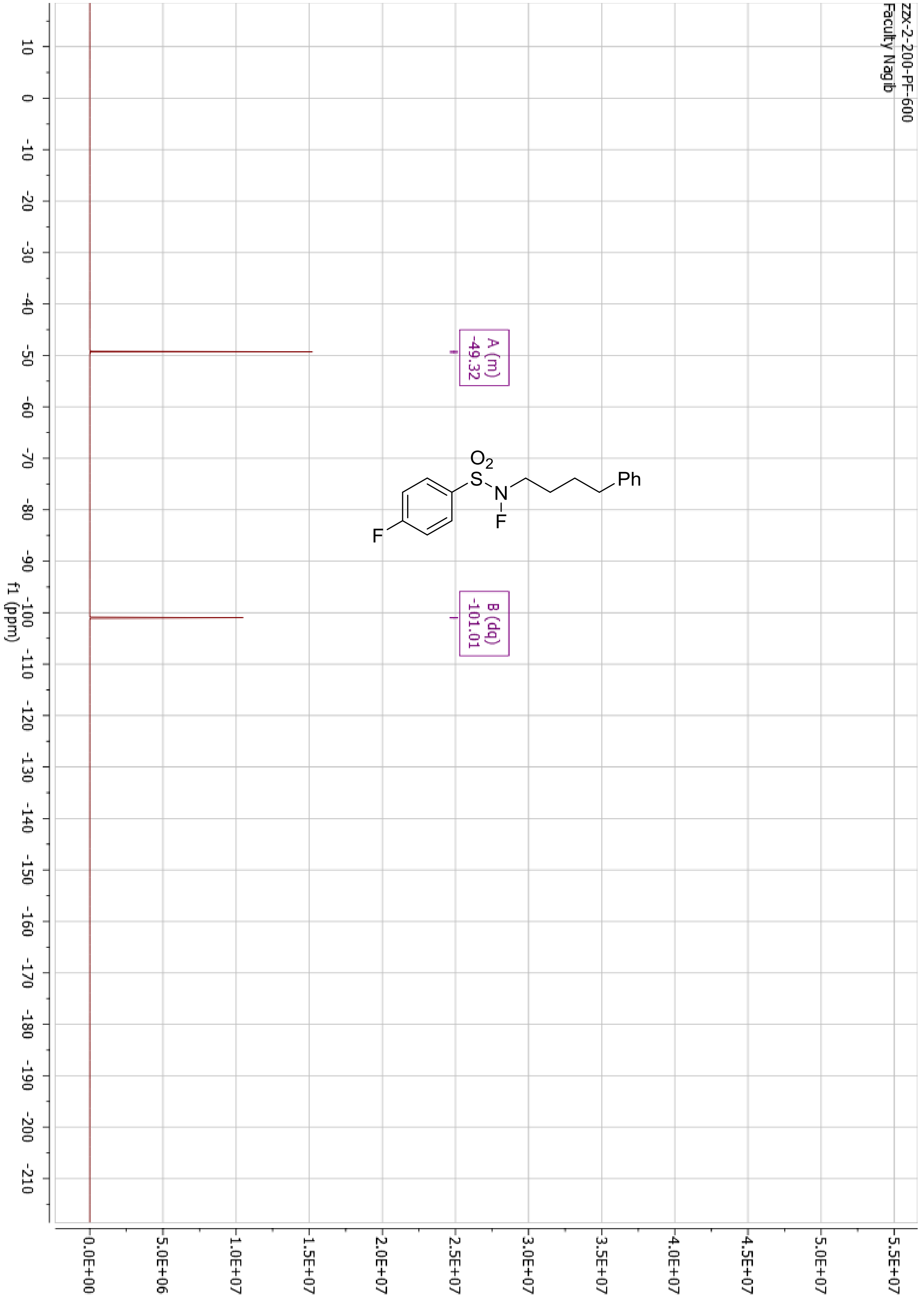
ZZX-2-200-PF-600
Faculty Nagib
A (m)
-49.32
B (dq)
-101.01
f1 (ppm)
Ph
F

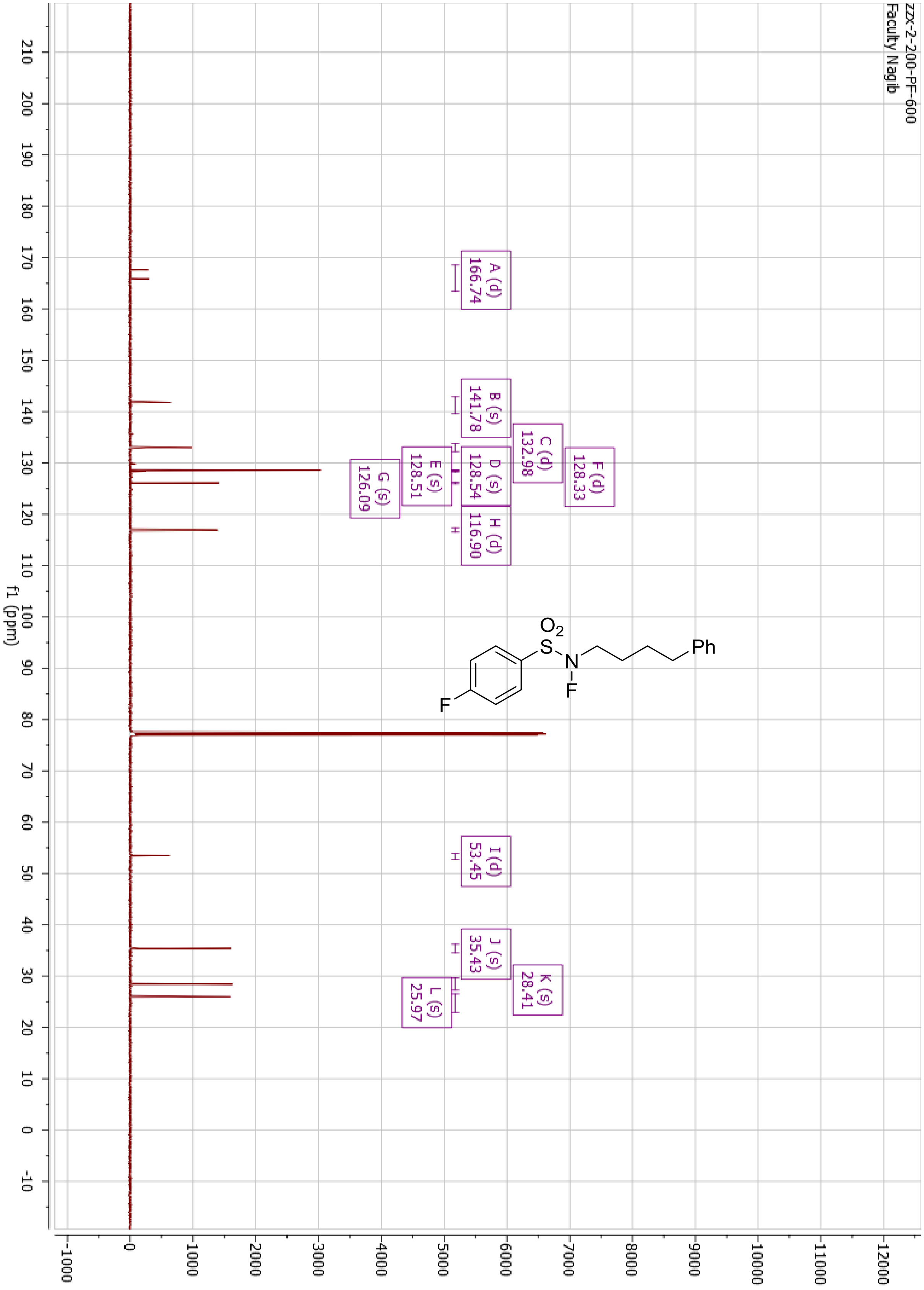
zzx-2-200-PF-600
Faculty Nagib
A (d) 166.74
B (s) 141.78
C (d) 132.98
D (s) 128.54
E (s) 128.51
F (d) 128.33
G (s) 126.09
H (d) 116.90
I (d) 53.45
J (s) 35.43
K (s) 28.41
L (s) 25.97
f1 (ppm)

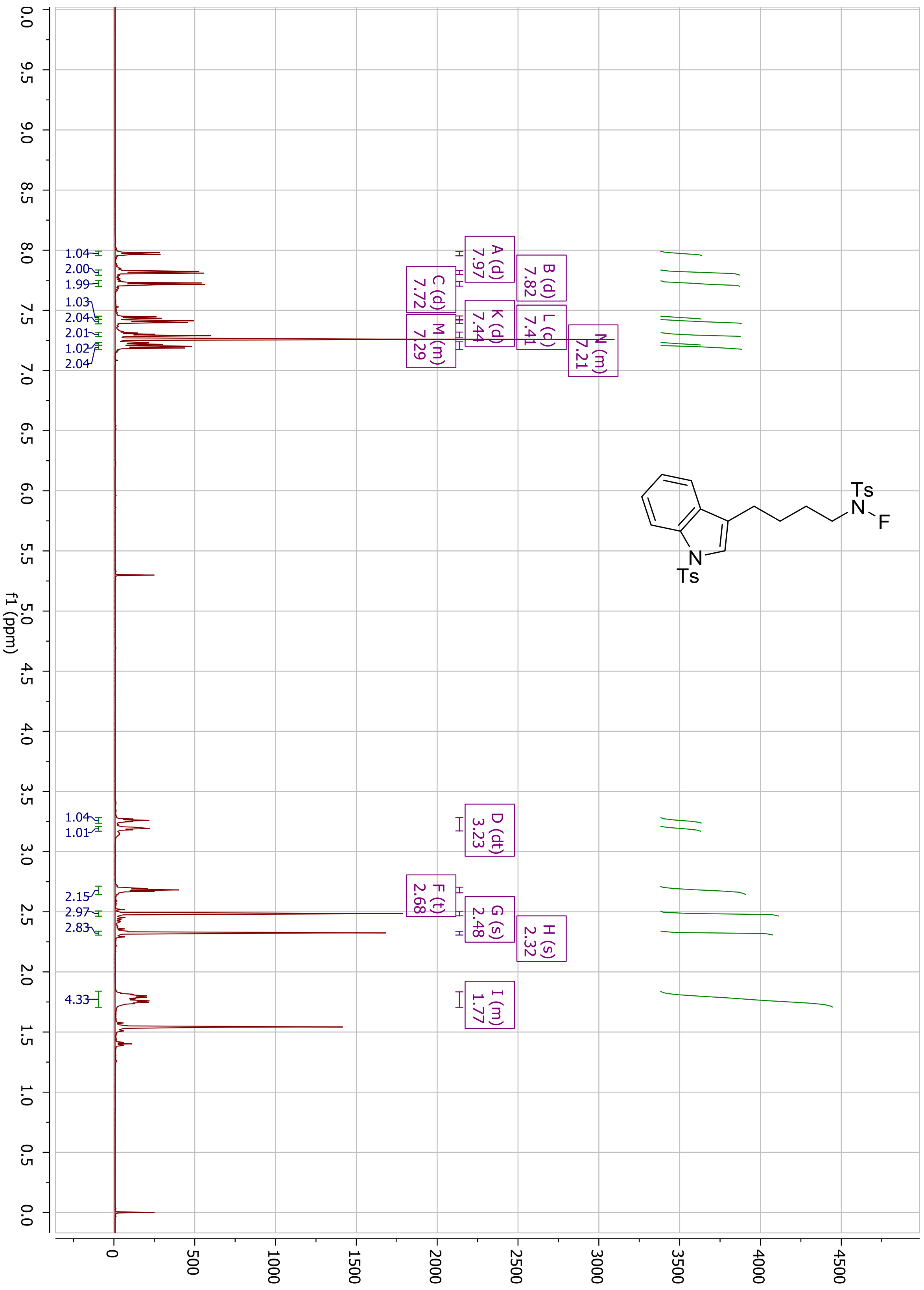
A (d) 7.97
B (d) 7.82
C (d) 7.72
K (d) 7.44
L (d) 7.41
M (m) 7.29
N (m) 7.21
D (dt) 3.23
F (t) 2.68
G (s) 2.48
H (s) 2.32
I (m) 1.77
1.04
2.00
1.99
1.03
2.04
2.01
1.02
2.04
1.04
1.01
2.15
2.97
2.83
4.33
f1 (ppm)
Ts
N
F
N
Ts

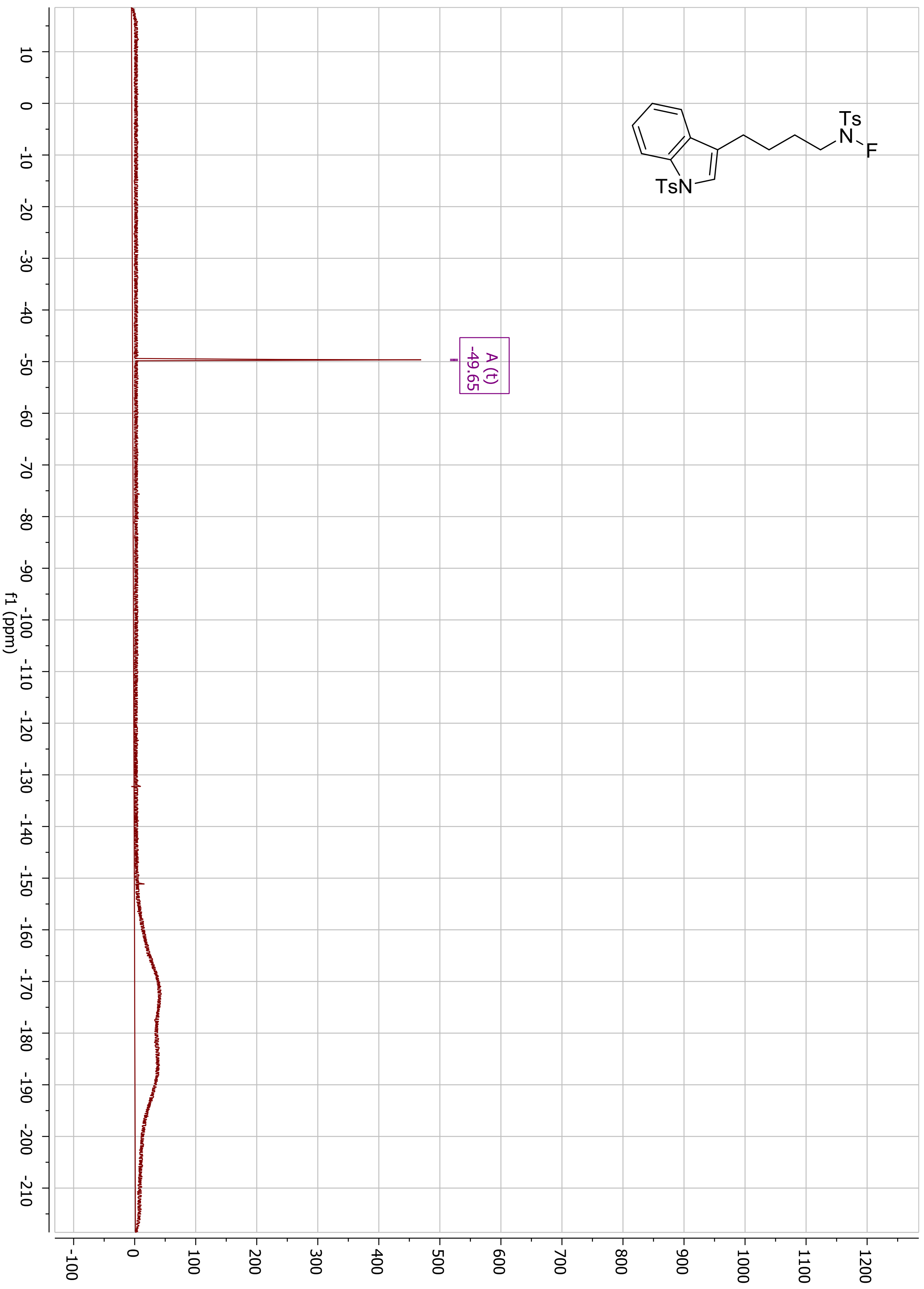
Ts
N
F
TsN
A (t)
-49.65
f1 (ppm)

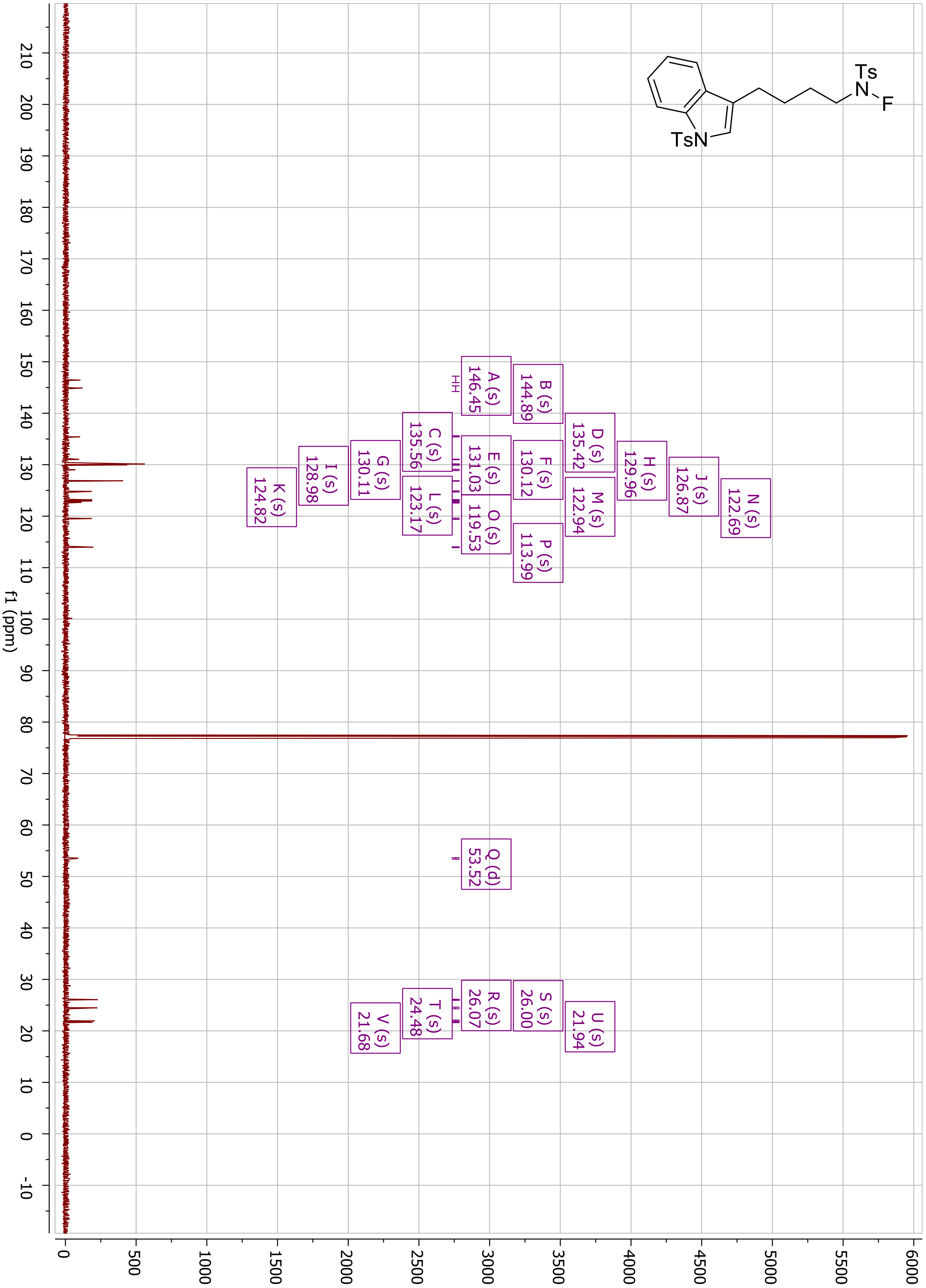
TsN
Ts
N
F
A (s) 146.45
B (s) 144.89
C (s) 135.56
D (s) 135.42
E (s) 131.03
F (s) 130.12
G (s) 130.11
H (s) 129.96
I (s) 128.98
J (s) 126.87
K (s) 124.82
L (s) 123.17
M (s) 122.94
N (s) 122.69
O (s) 119.53
P (s) 113.99
Q (d) 53.52
R (s) 26.07
S (s) 26.00
T (s) 24.48
U (s) 21.94
V (s) 21.68
f1 (ppm)

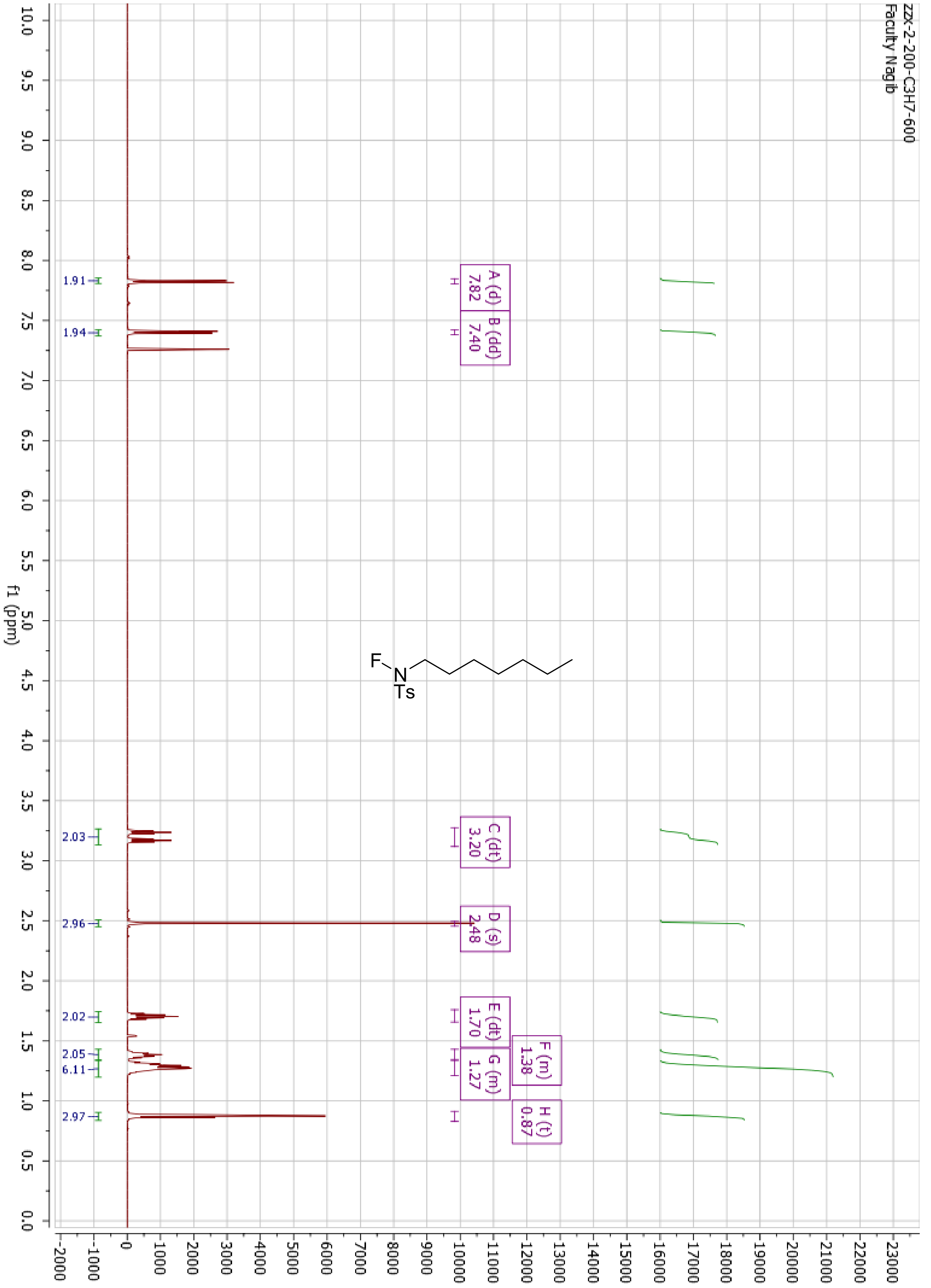
zzx-2-200-C3H7-600
Faculty Nagib
A (d) 7.82
B (dd) 7.40
C (dt) 3.20
D (s) 2.48
E (dt) 1.70
F (m) 1.38
G (m) 1.27
H (t) 0.87
1.91
1.94
2.03
2.96
2.02
2.05
6.11
2.97
F
N
Ts
f1 (ppm)

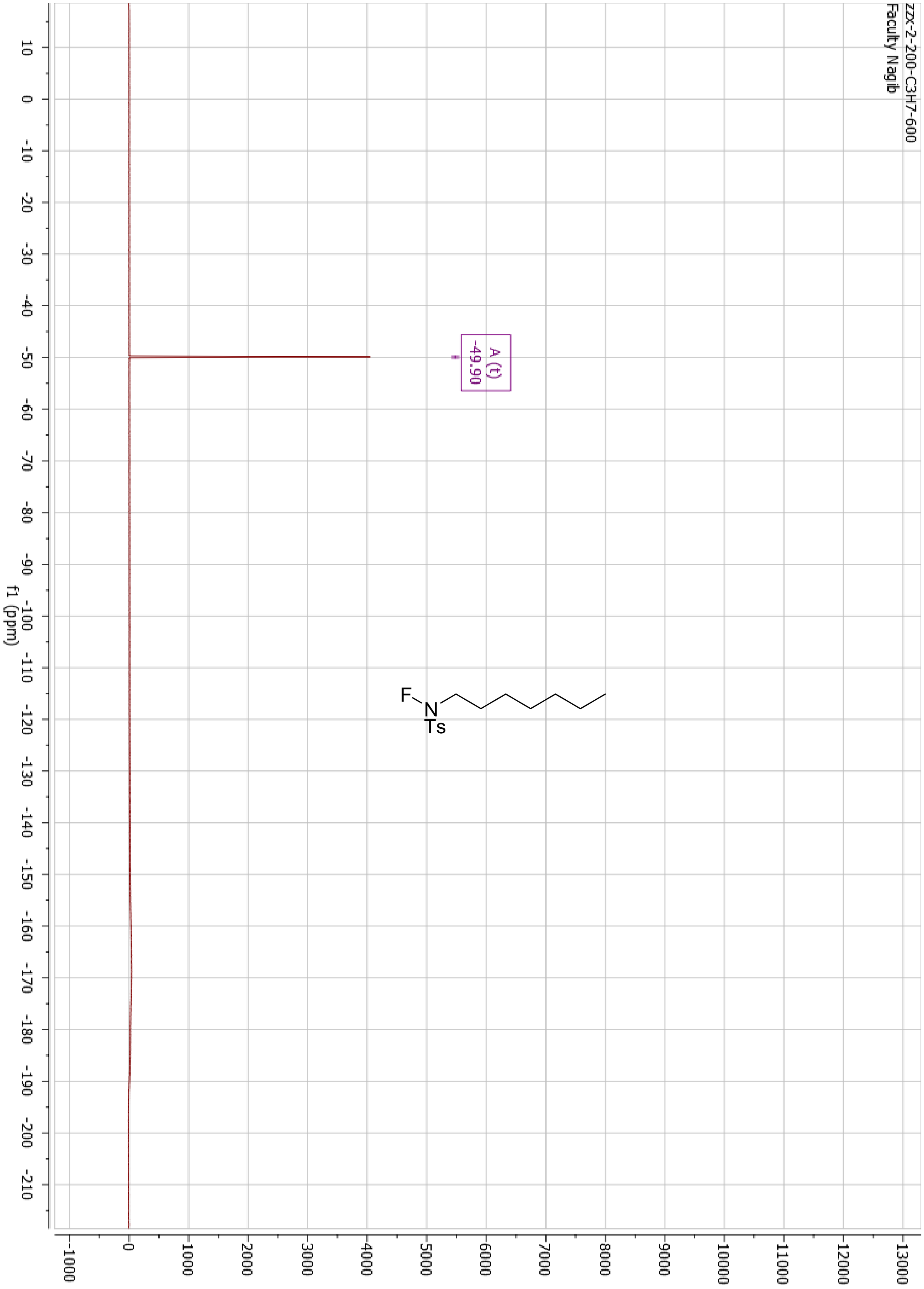
zzx-2-200-C3H7-600
Faculty Nagib
A (t)
-49.90
f1 (ppm)
F
N
Ts

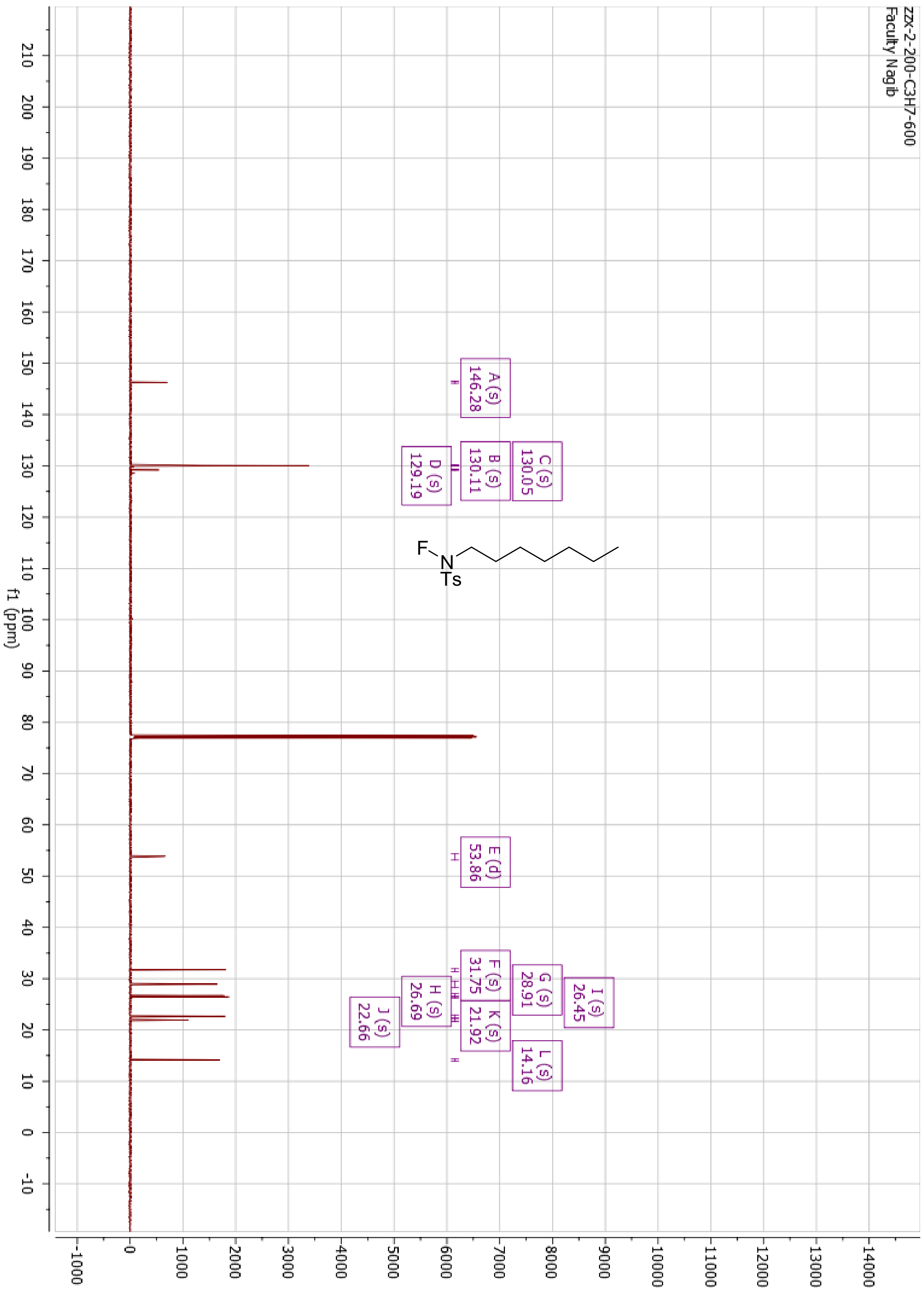
zzx-2-200-C3H7-600
Faculty Nagib
A (s)
146.28
B (s)
130.11
C (s)
130.05
D (s)
129.19
E (d)
53.86
F (s)
31.75
G (s)
28.91
H (s)
26.69
I (s)
26.45
J (s)
22.66
K (s)
21.92
L (s)
14.16
F
N
Ts
f1 (ppm)

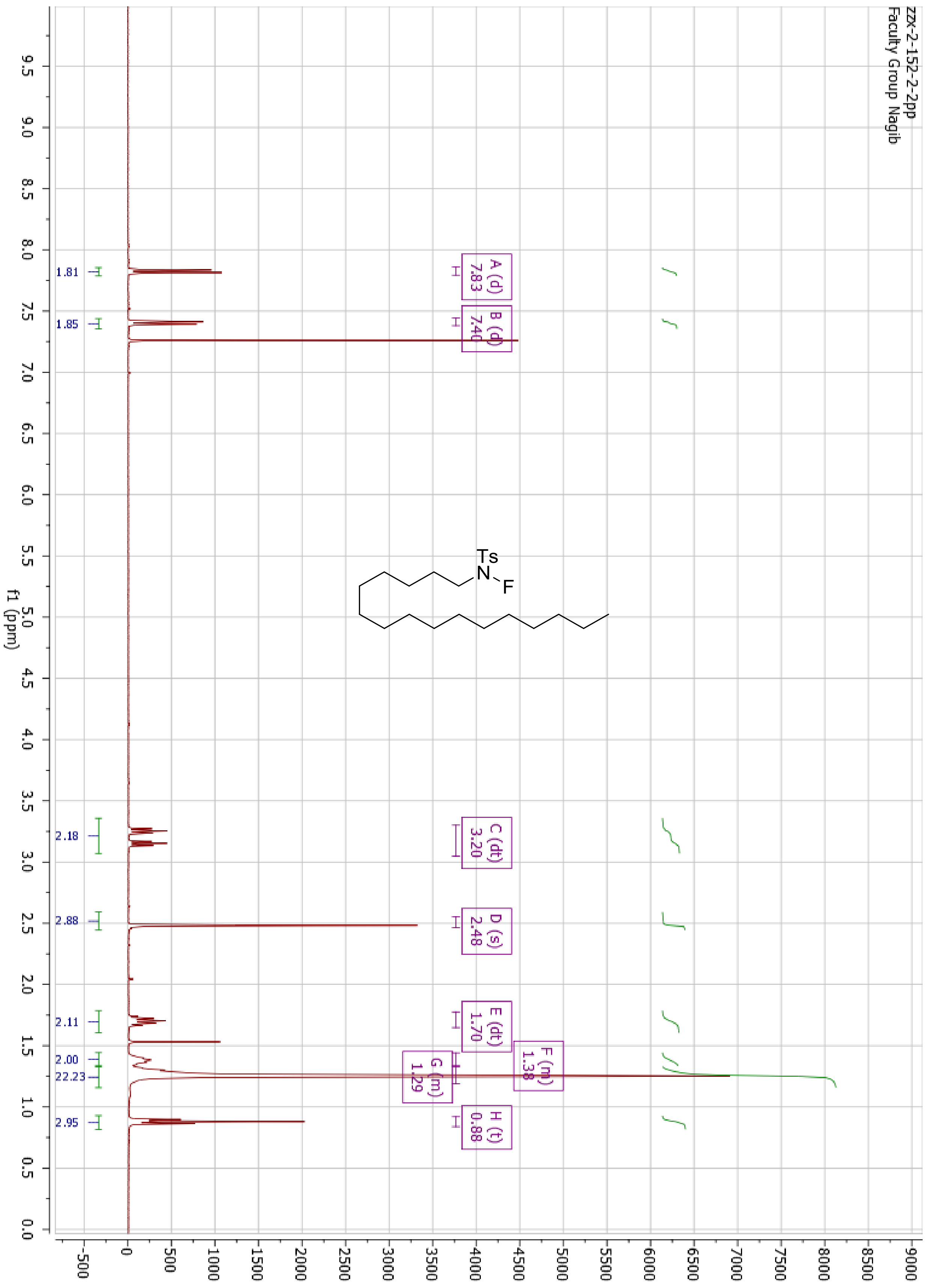
zzx-2-152-2-2pp
Faculty Group Nagib
A (d) 7.83
B (d) 7.40
C (dt) 3.20
D (s) 2.48
E (dt) 1.70
F (m) 1.38
G (m) 1.29
H (t) 0.88
1.81
1.85
2.18
2.88
2.11
2.00
22.23
2.95
Ts
N
F
f1 (ppm)

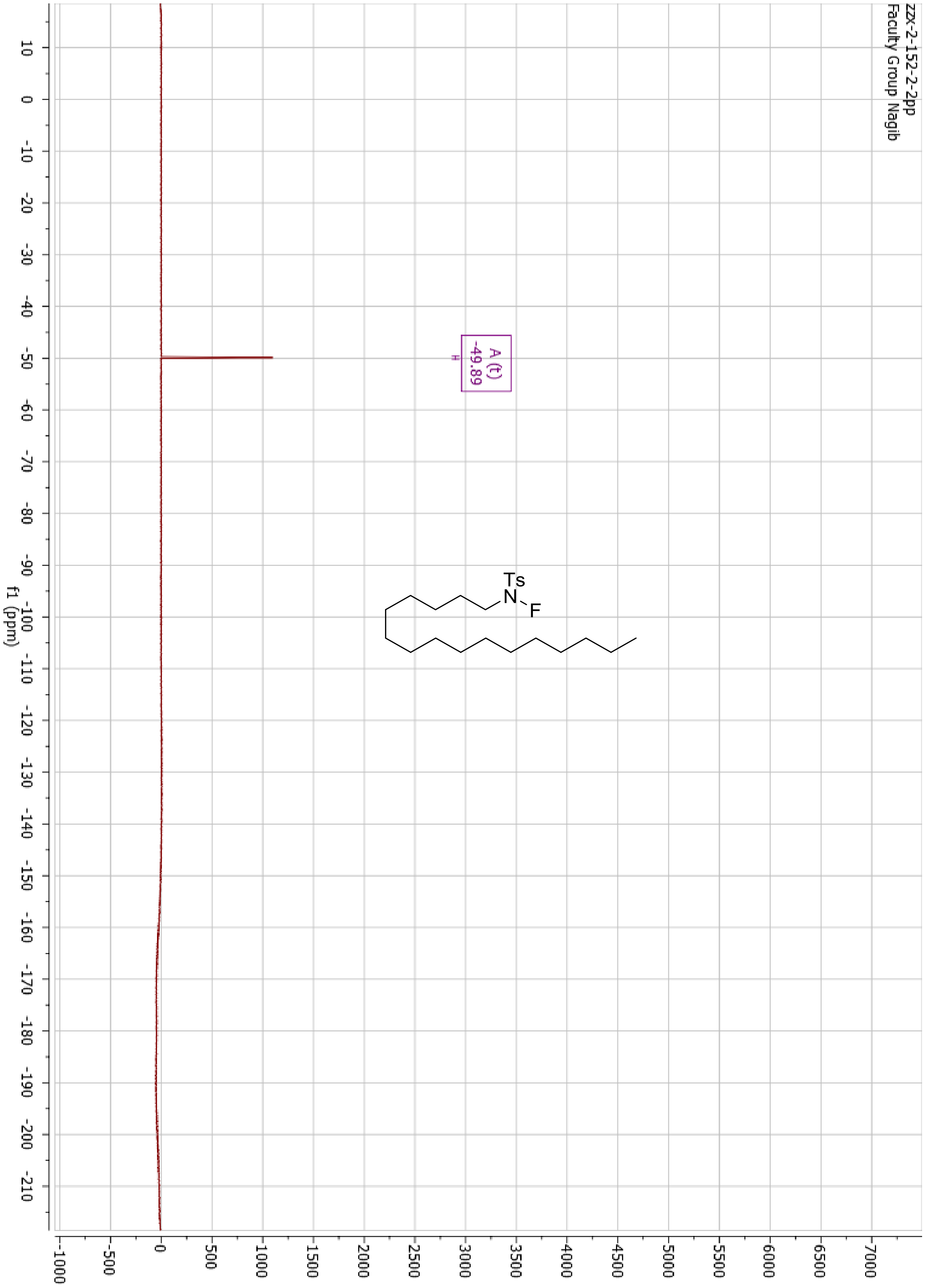
zzx-2-152-2-2pp
Faculty Group Nagib
A (t)
-49.89
Ts
N
F
f1 (ppm)

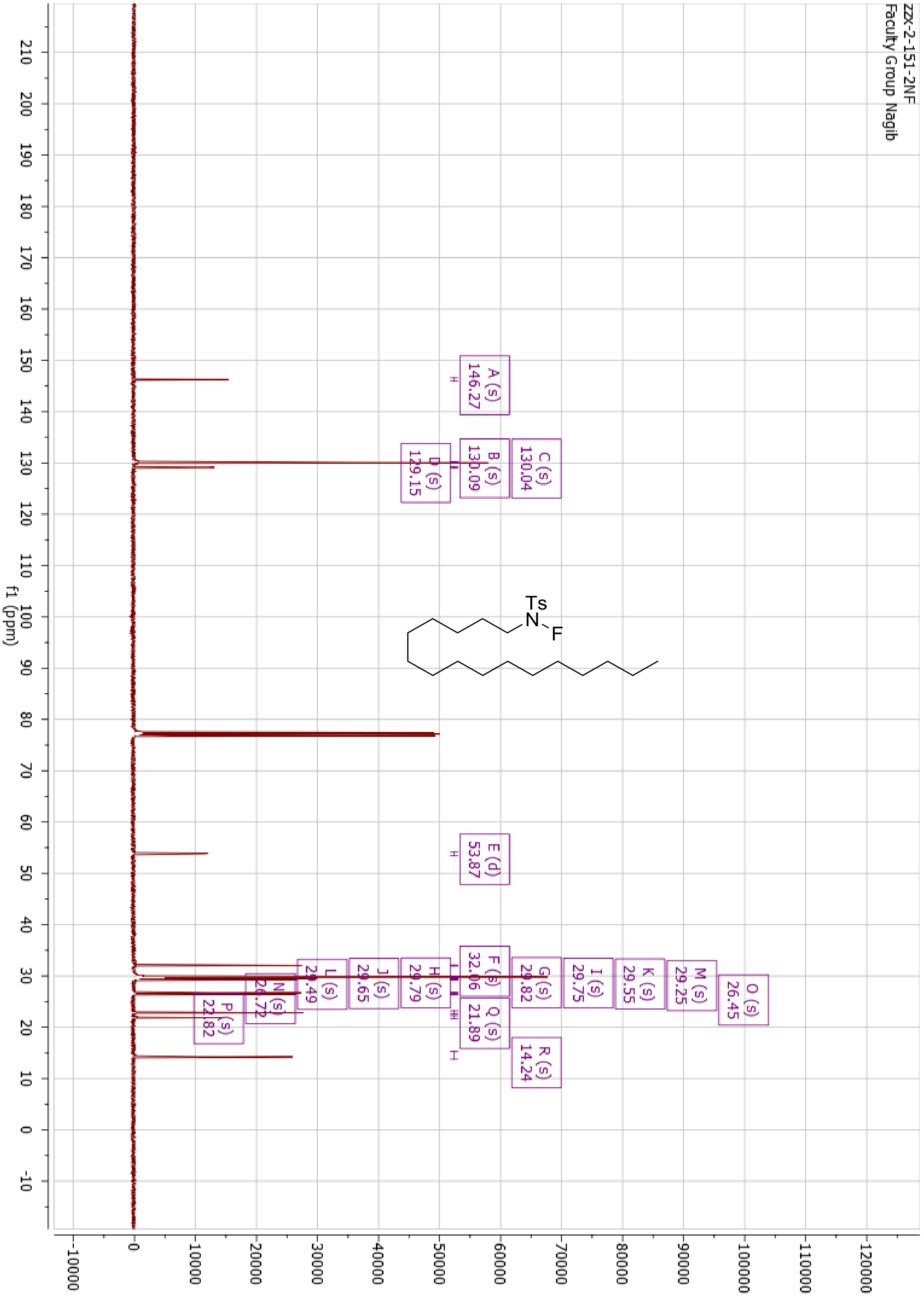
zzx-2-151-2NF
Faculty Group Nagib
A (s) 146.27
B (s) 130.09
C (s) 130.04
D (s) 129.15
E (d) 53.87
F (s) 32.06
G (s) 29.82
H (s) 29.79
I (s) 29.75
J (s) 29.65
K (s) 29.55
L (s) 29.49
M (s) 29.25
N (s) 26.72
O (s) 26.45
P (s) 22.82
Q (s) 21.89
R (s) 14.24
Ts
N
F
f1 (ppm)

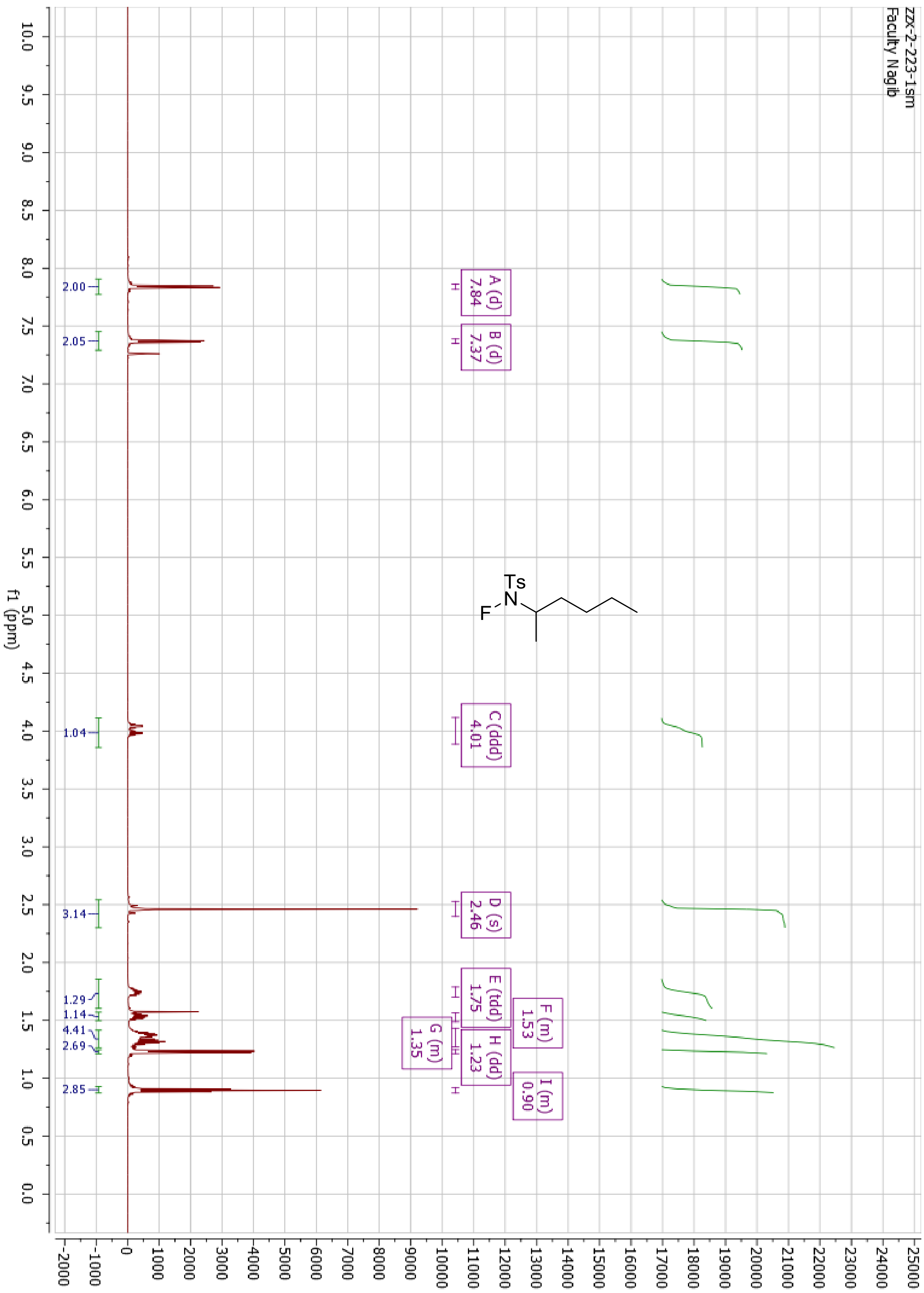
zzx-2-223-1sm
Faculty Nagib
Ts
N
F
A (d) 7.84
B (d) 7.37
C (ddd) 4.01
D (s) 2.46
E (tdd) 1.75
F (m) 1.53
G (m) 1.35
H (dd) 1.23
I (m) 0.90
2.00
2.05
1.04
3.14
1.29
1.14
4.41
2.69
2.85
f1 (ppm)

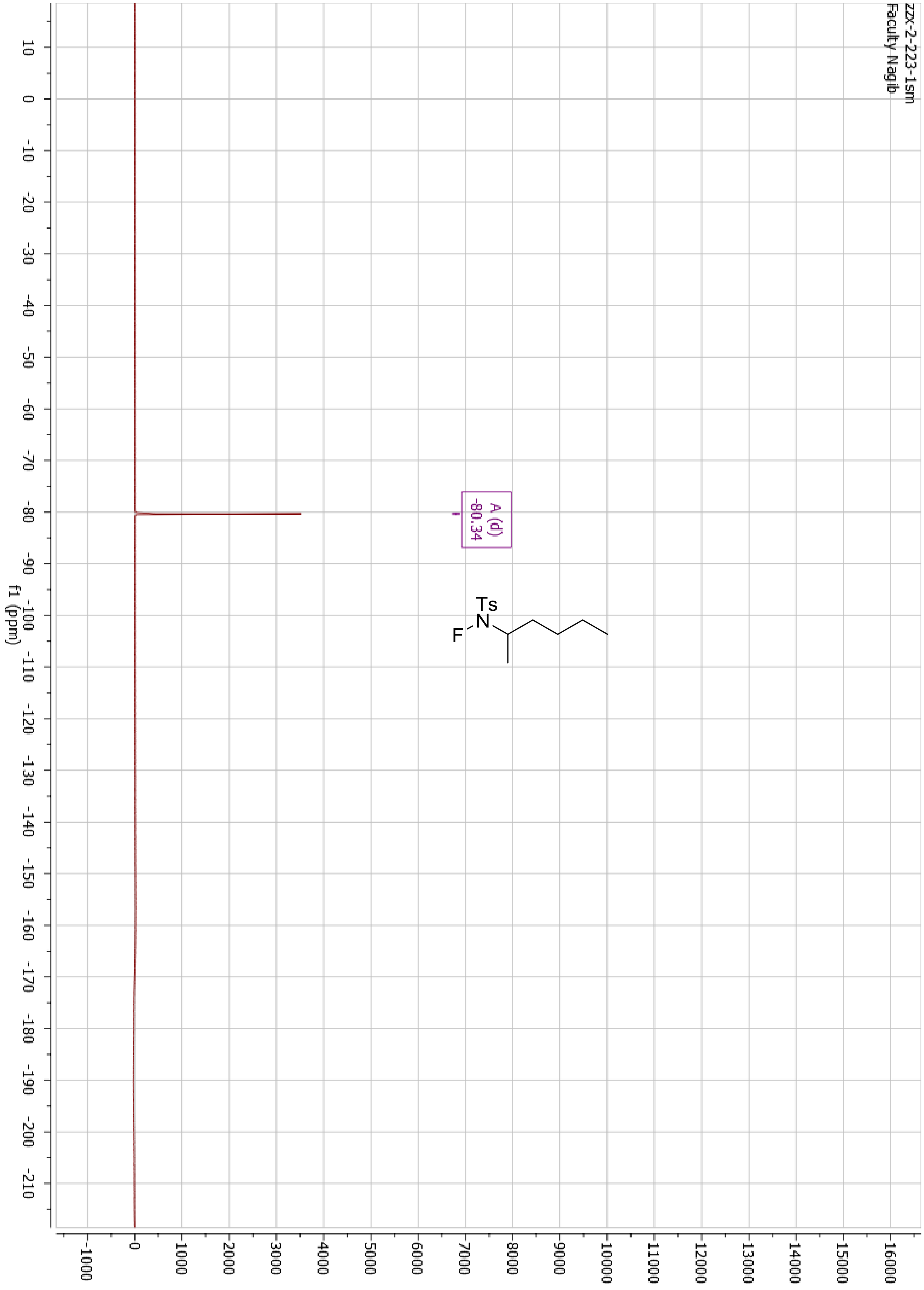
zzx-2-223-1sm
Faculty Nagib
A (d)
-80.34
Ts
N
F
f1 (ppm)

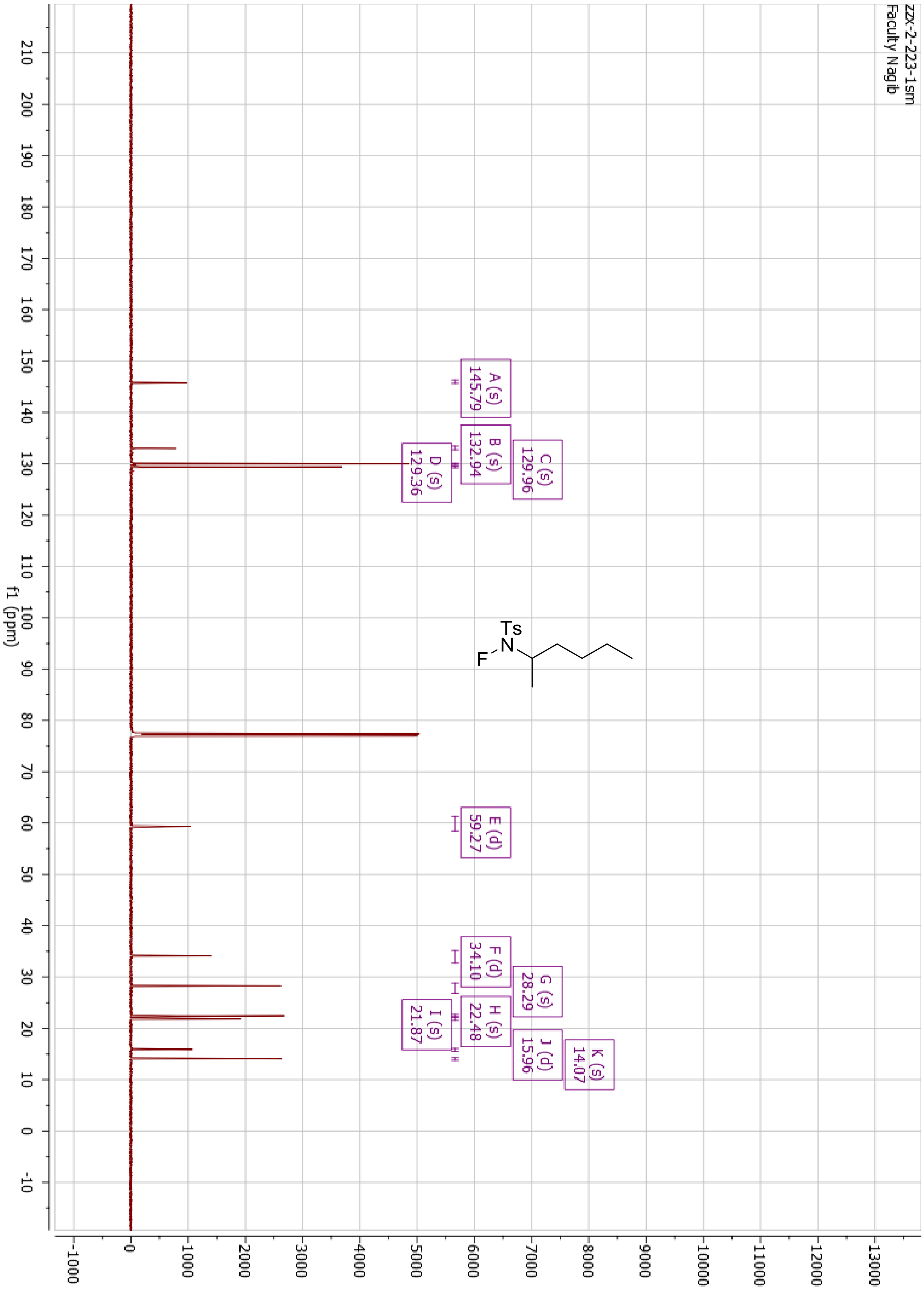
zzx-2-223-1sm
Faculty Nagib
A (s) 145.79
B (s) 132.94
C (s) 129.96
D (s) 129.36
E (d) 59.27
F (d) 34.10
G (s) 28.29
H (s) 22.48
I (s) 21.87
J (d) 15.96
K (s) 14.07
Ts
N
F
f1 (ppm)

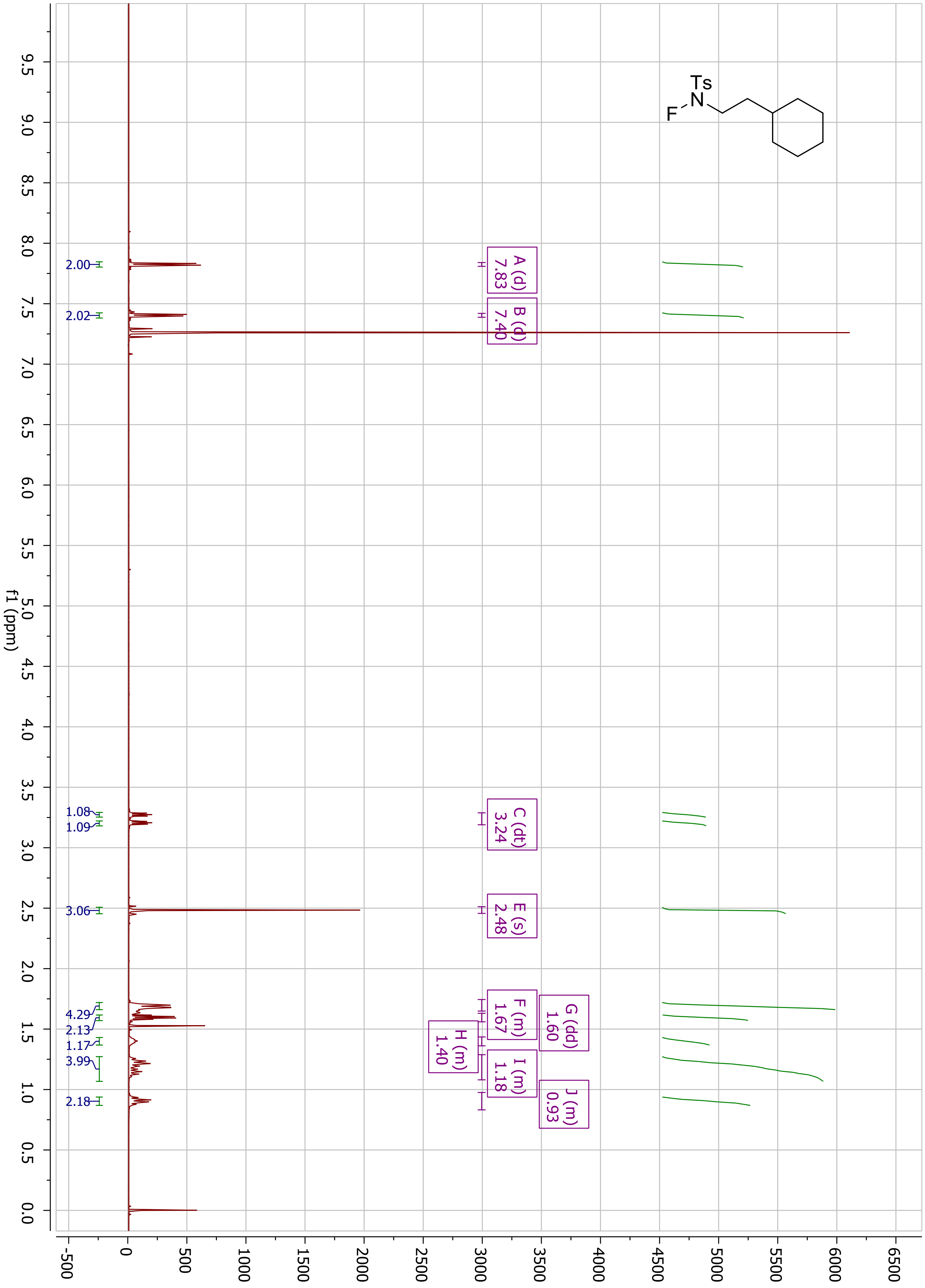
Ts
N
F
A (d) 7.83
B (d) 7.40
C (dt) 3.24
E (s) 2.48
F (m) 1.67
G (dd) 1.60
H (m) 1.40
I (m) 1.18
J (m) 0.93
f1 (ppm)

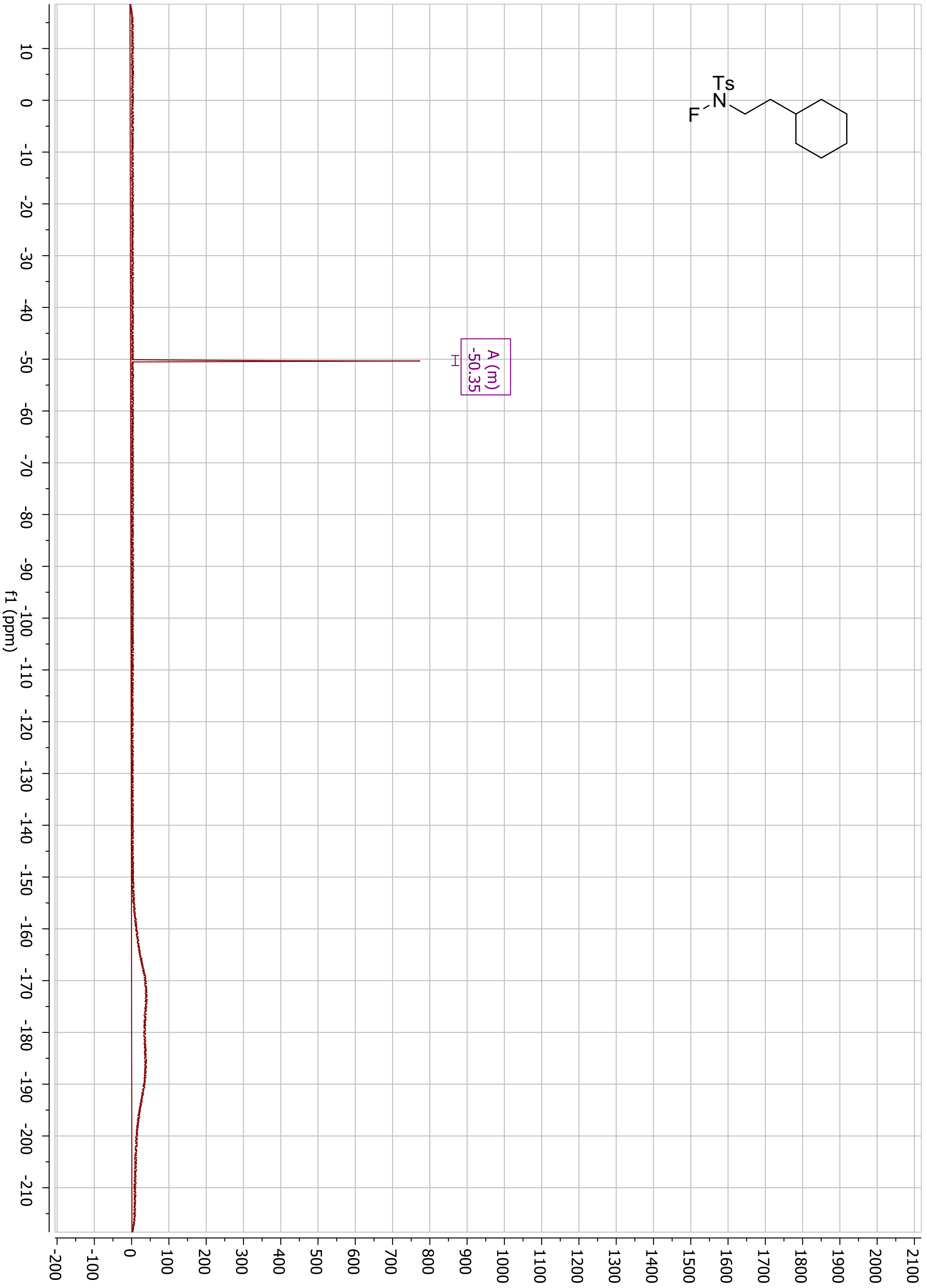
Ts
N
F
A (m)
-50.35
f1 (ppm)

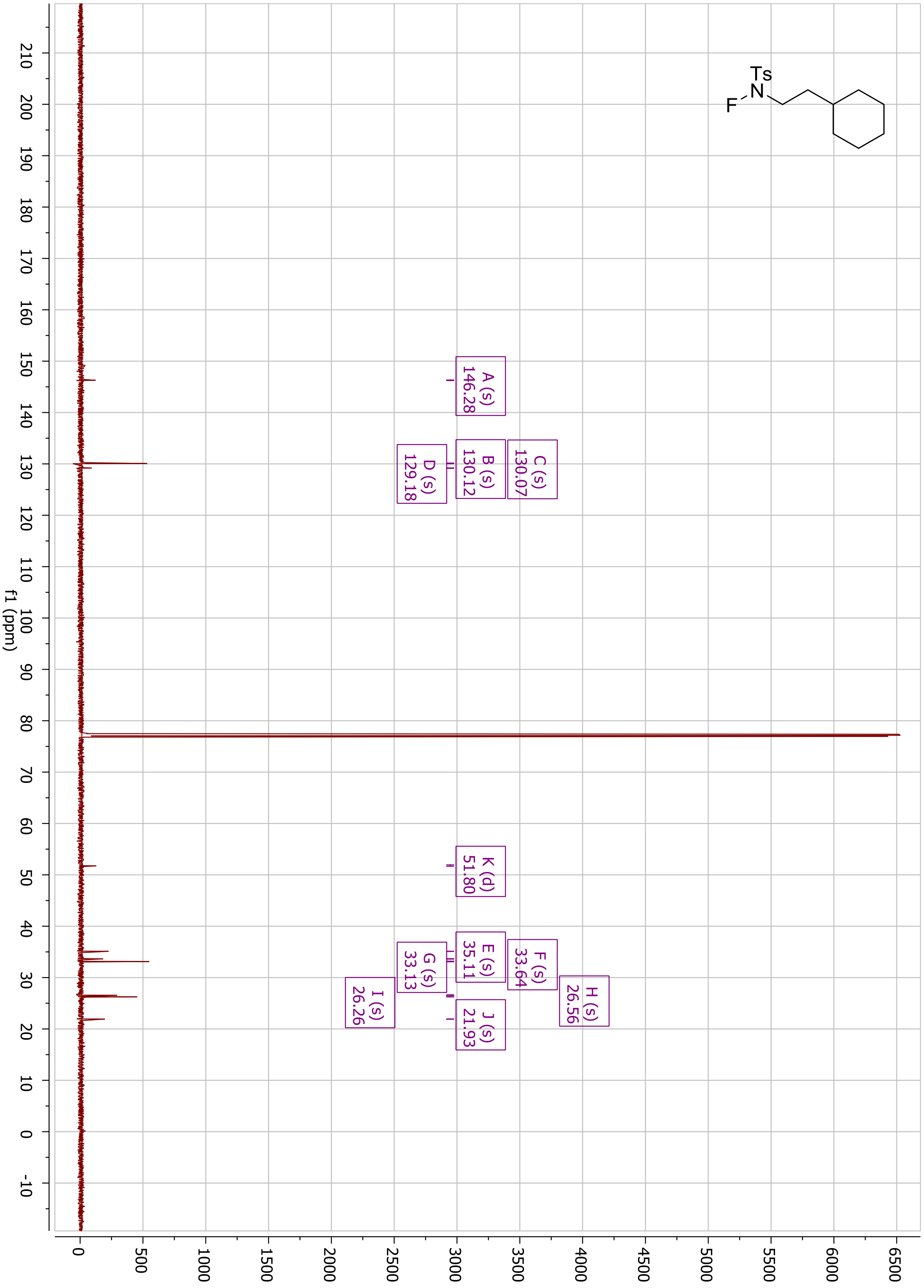
Ts
N
F
A (s)
146.28
B (s)
130.12
C (s)
130.07
D (s)
129.18
K (d)
51.80
E (s)
35.11
F (s)
33.64
G (s)
33.13
H (s)
26.56
I (s)
26.26
J (s)
21.93
f1 (ppm)

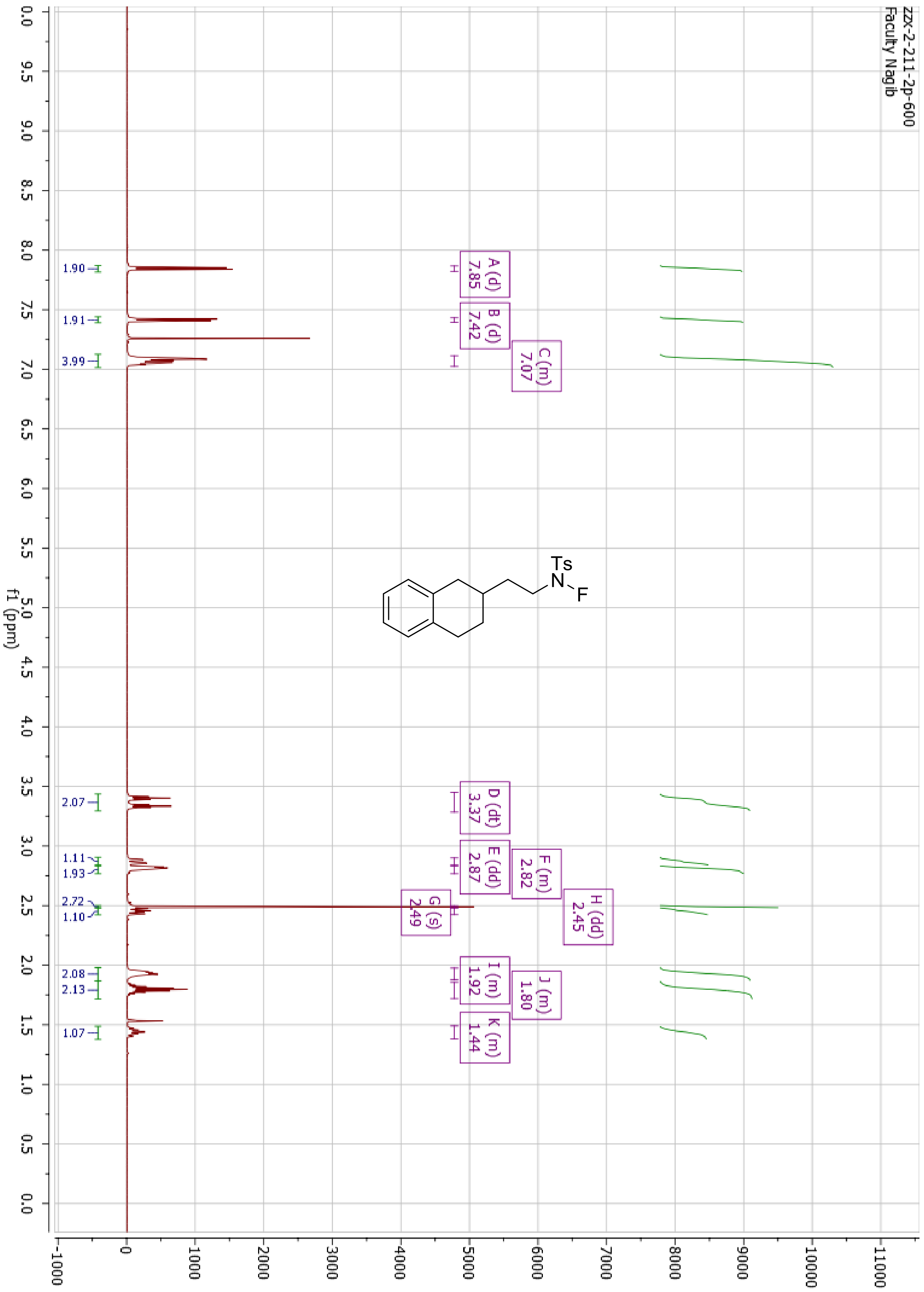

ZZX-2-211-2p-600
Faculty Nagib
Ts
N
F
A (d) 7.85
B (d) 7.42
C (m) 7.07
D (dt) 3.37
E (dd) 2.87
F (m) 2.82
G (s) 2.49
H (dd) 2.45
I (m) 1.92
J (m) 1.80
K (m) 1.44
1.90
1.91
3.99
2.07
1.11
1.93
2.72
1.10
2.08
2.13
1.07
f1 (ppm)

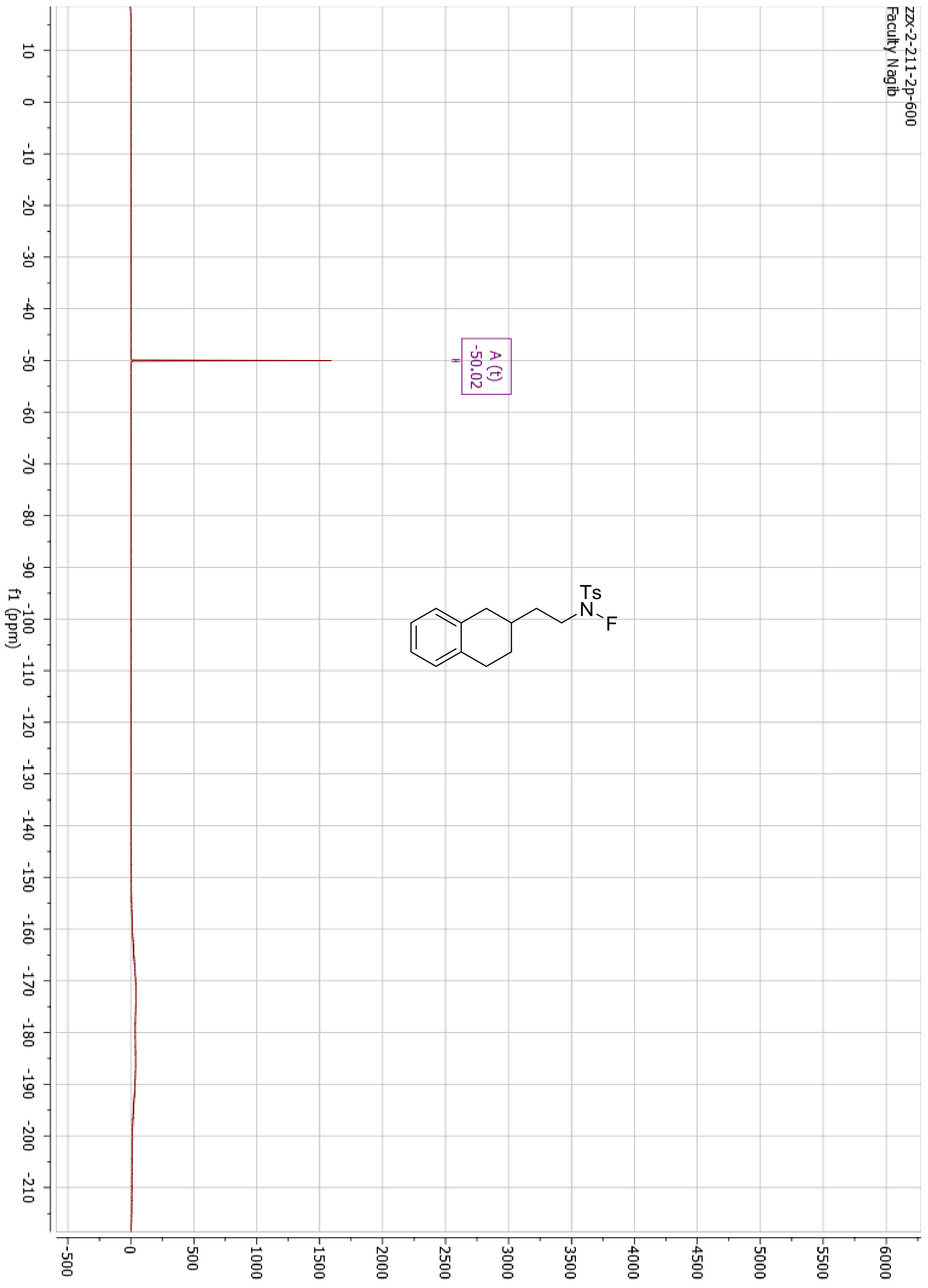
zzx-2-211-2p-600
Faculty Nagib
A (t)
-50.02
Ts
N
F
f1 (ppm)

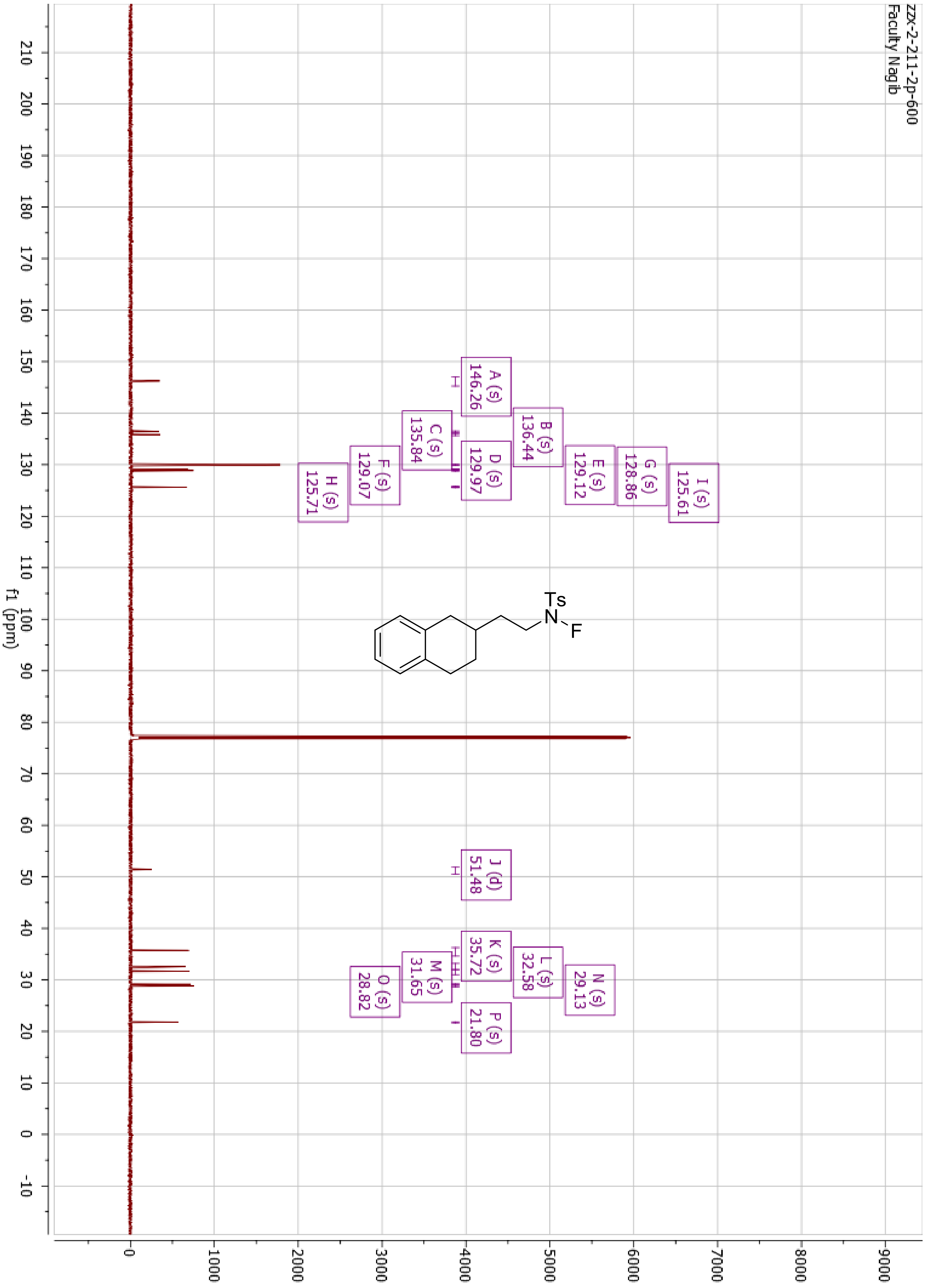
zzx-2-211-2p-600
Faculty Nagib
A (s) 146.26
B (s) 136.44
C (s) 135.84
D (s) 129.97
E (s) 129.12
F (s) 129.07
G (s) 128.86
H (s) 125.71
I (s) 125.61
J (d) 51.48
K (s) 35.72
L (s) 32.58
M (s) 31.65
N (s) 29.13
O (s) 28.82
P (s) 21.80
Ts
N
F
f1 (ppm)

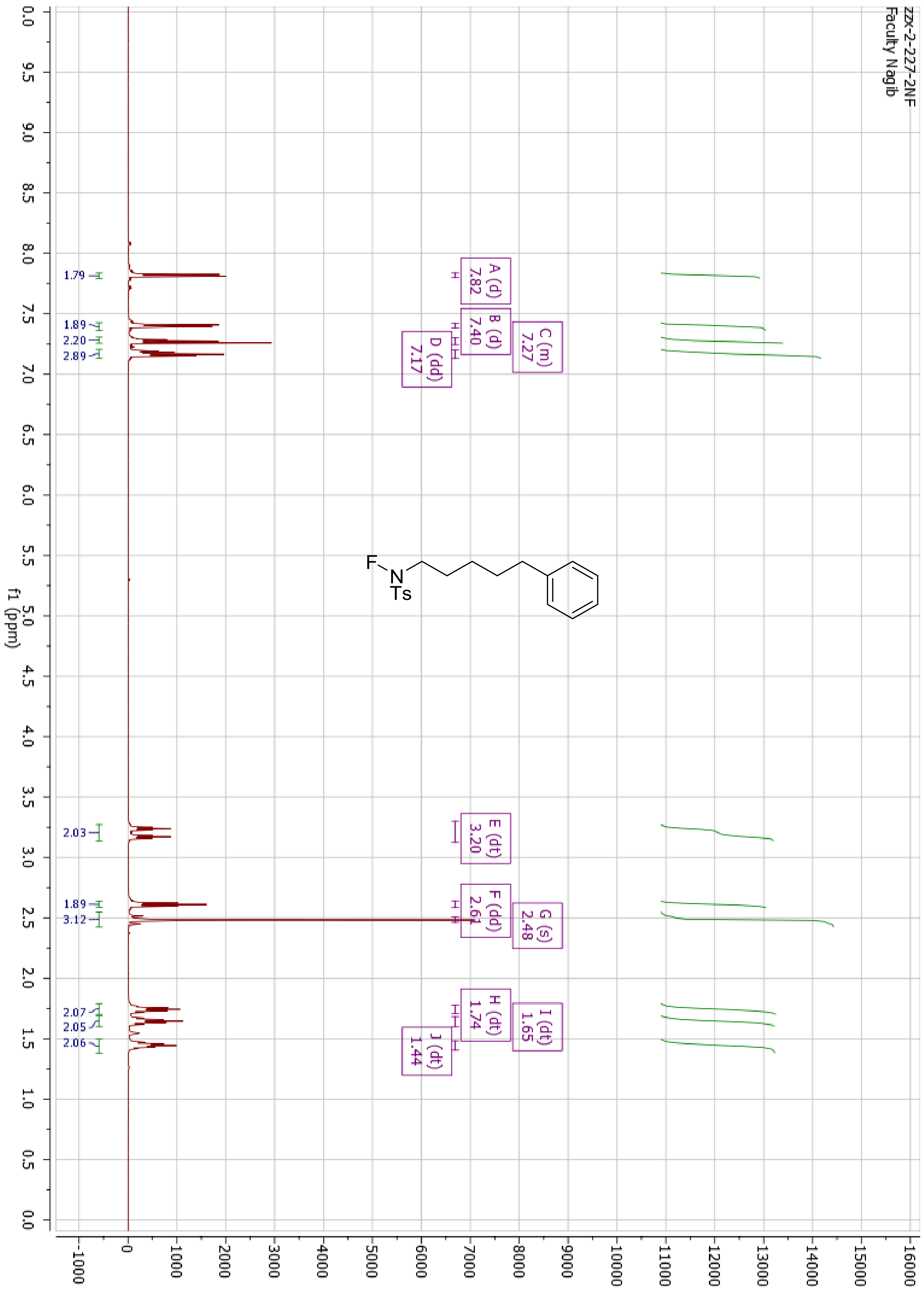

zzx-2-227-2NF
Faculty Nagib
A (d) 7.82
B (d) 7.40
C (m) 7.27
D (dd) 7.17
E (dt) 3.20
F (dd) 2.61
G (s) 2.48
H (dt) 1.74
I (dt) 1.65
J (dt) 1.44
1.79
1.89
2.20
2.89
2.03
1.89
3.12
2.07
2.05
2.06
F
N
Ts
f1 (ppm)

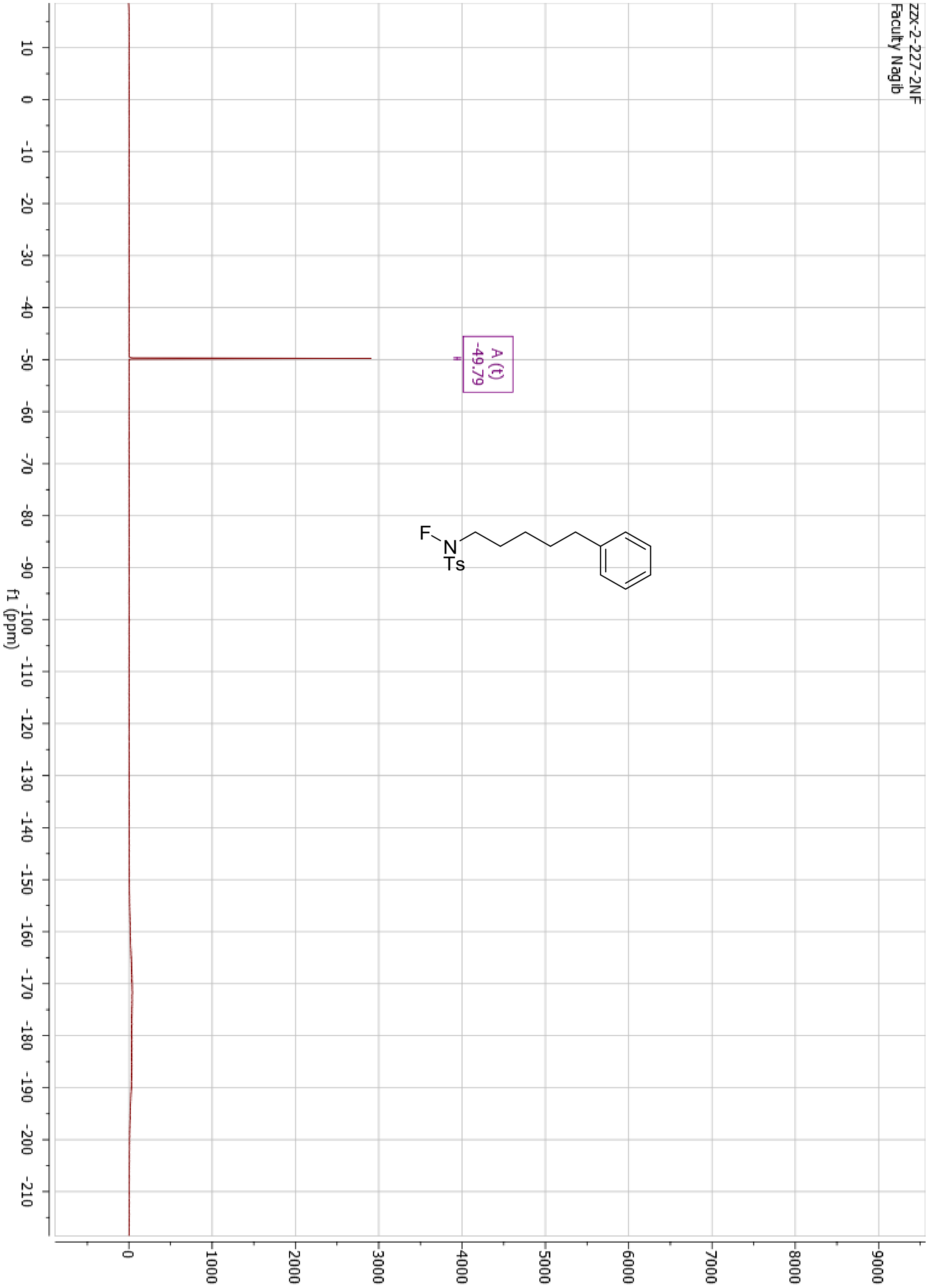
zzx-2-227-2NF
Faculty Nagib
A (t)
-49.79
F
N
Ts
f1 (ppm)

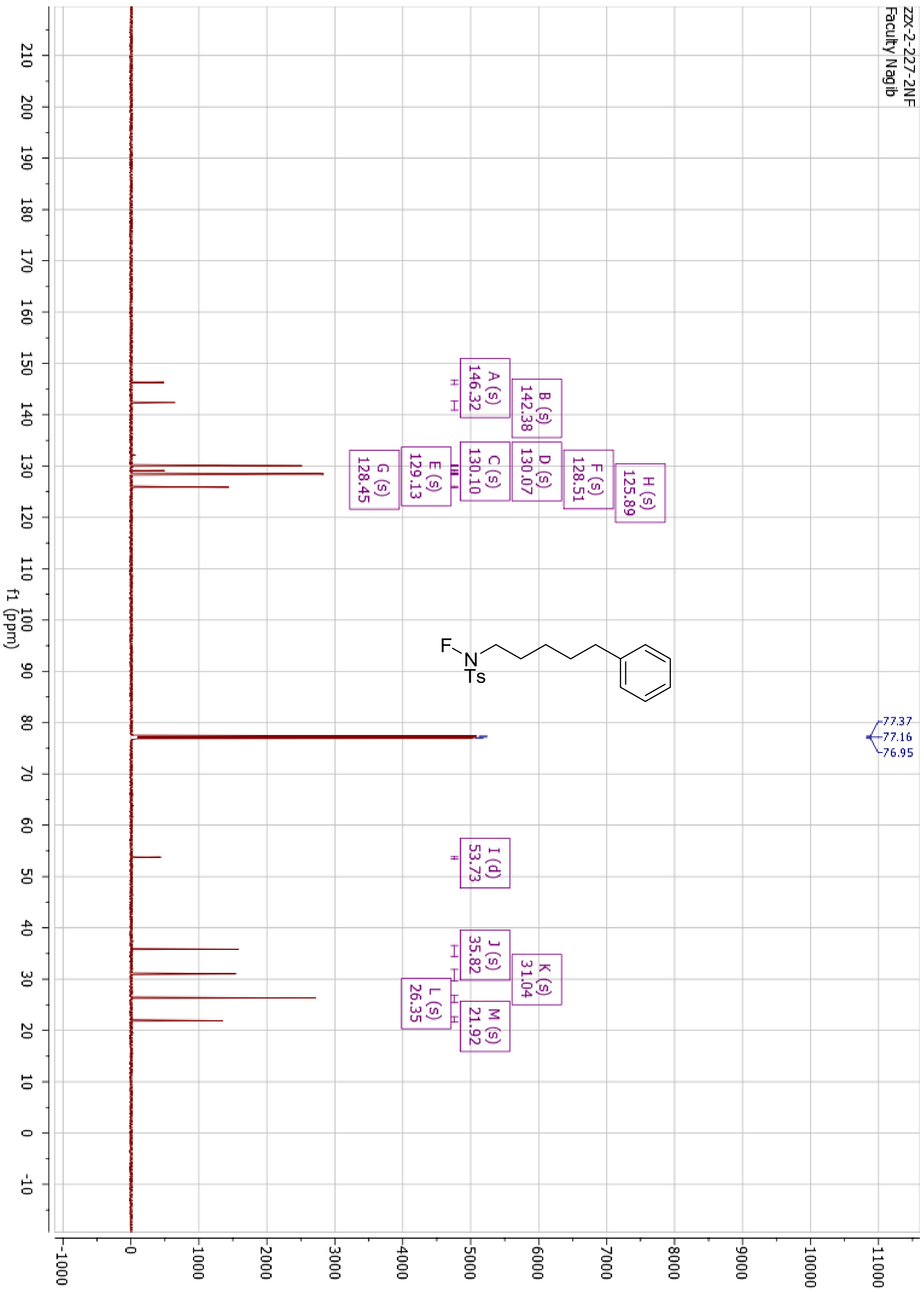
ZZX-2-227-2NF
Faculty Nagib
A (s) 146.32
B (s) 142.38
C (s) 130.10
D (s) 130.07
E (s) 129.13
F (s) 128.51
G (s) 128.45
H (s) 125.89
I (d) 53.73
J (s) 35.82
K (s) 31.04
L (s) 26.35
M (s) 21.92
77.37
77.16
76.95
F
N
Ts
f1 (ppm)

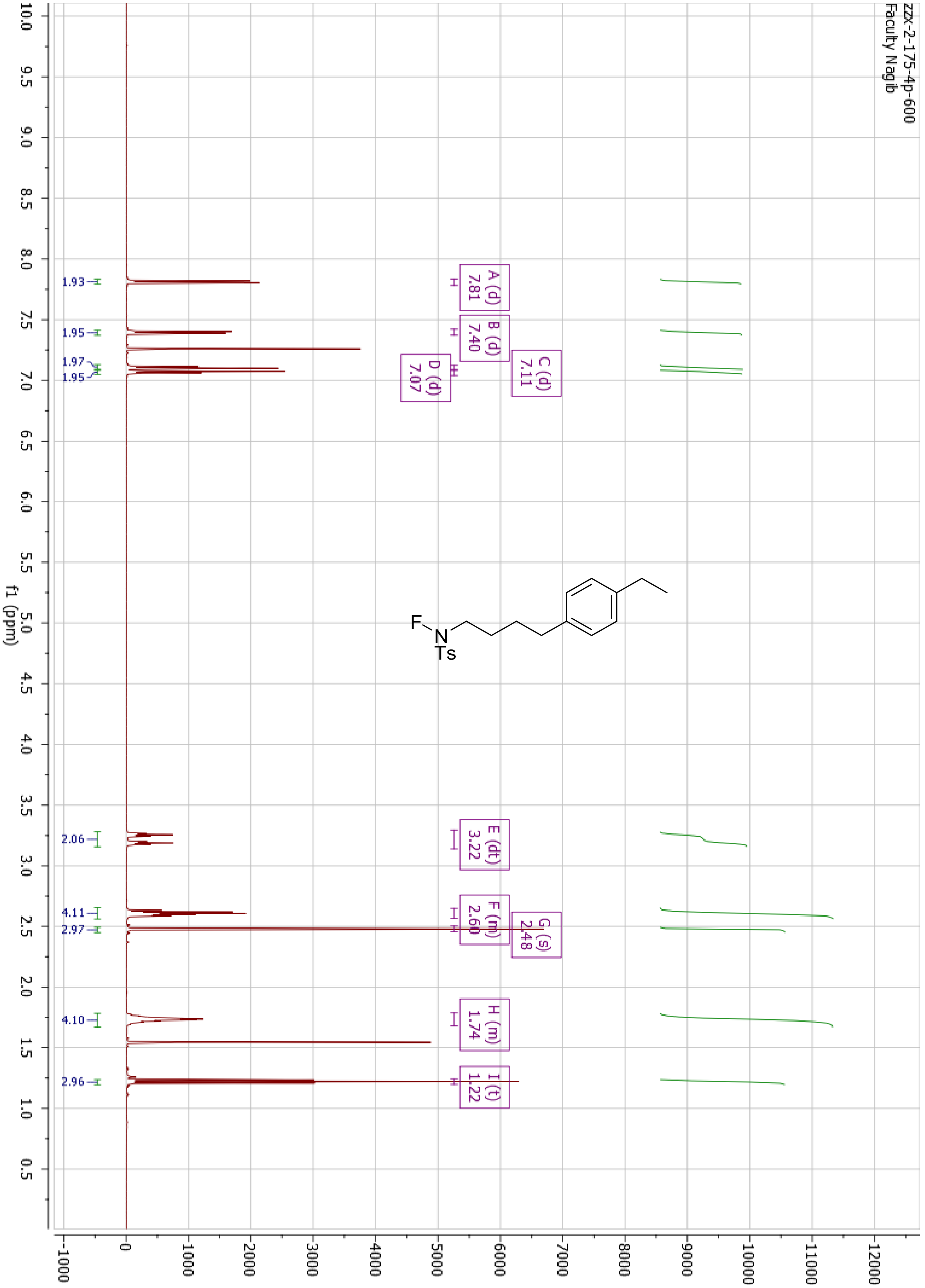
ZZX-2-175-4p-600
Faculty Nagib
A (d) 7.81
B (d) 7.40
C (d) 7.11
D (d) 7.07
E (dt) 3.22
F (m) 2.60
G (s) 2.48
H (m) 1.74
I (t) 1.22
1.93
1.95
1.97
1.95
2.06
4.11
2.97
4.10
2.96
f1 (ppm)
F
N
Ts

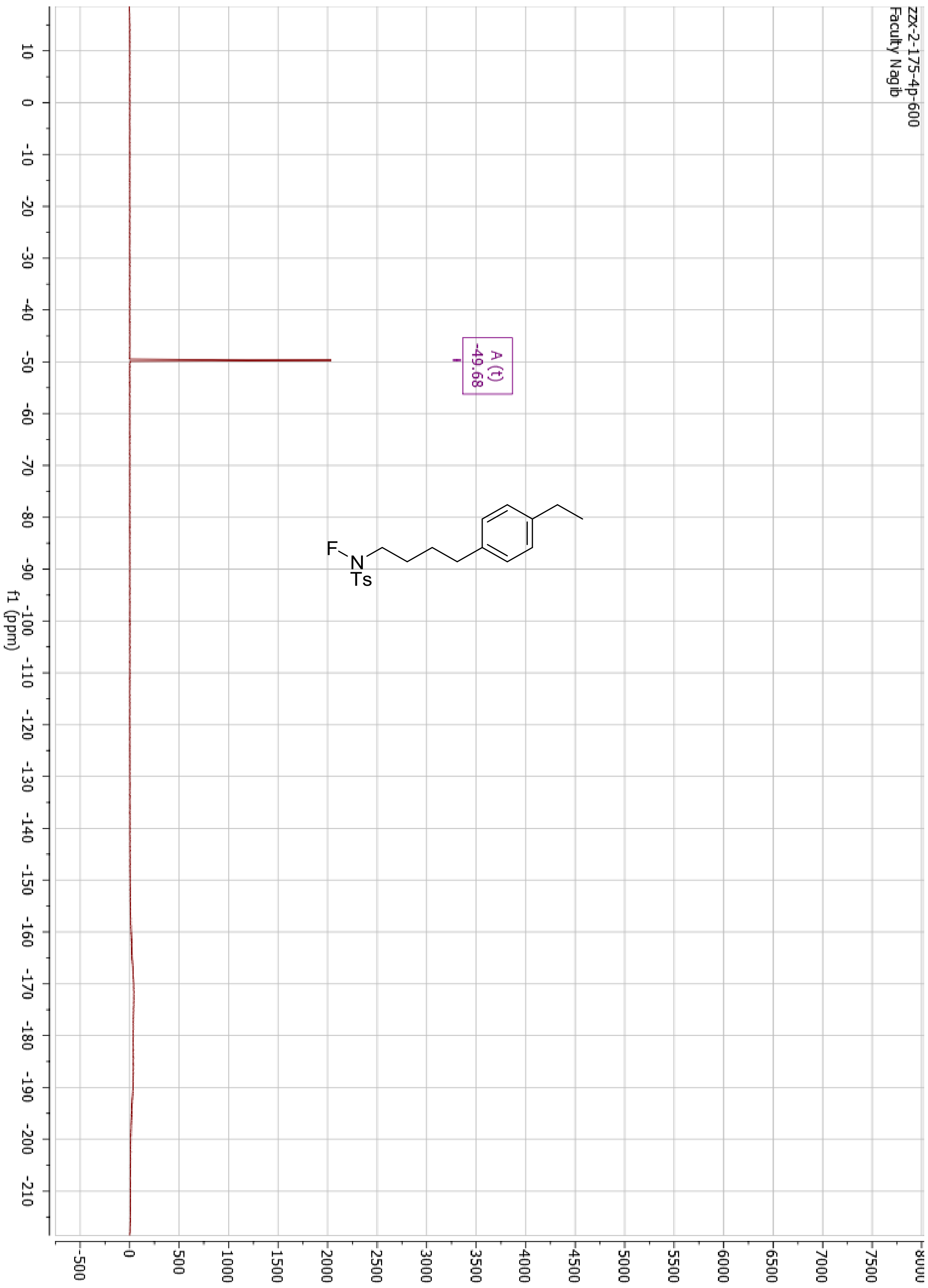
zzx-2-175-4p-600
Faculty Nagib
A (t)
-49.68
F
N
Ts
f1 (ppm)

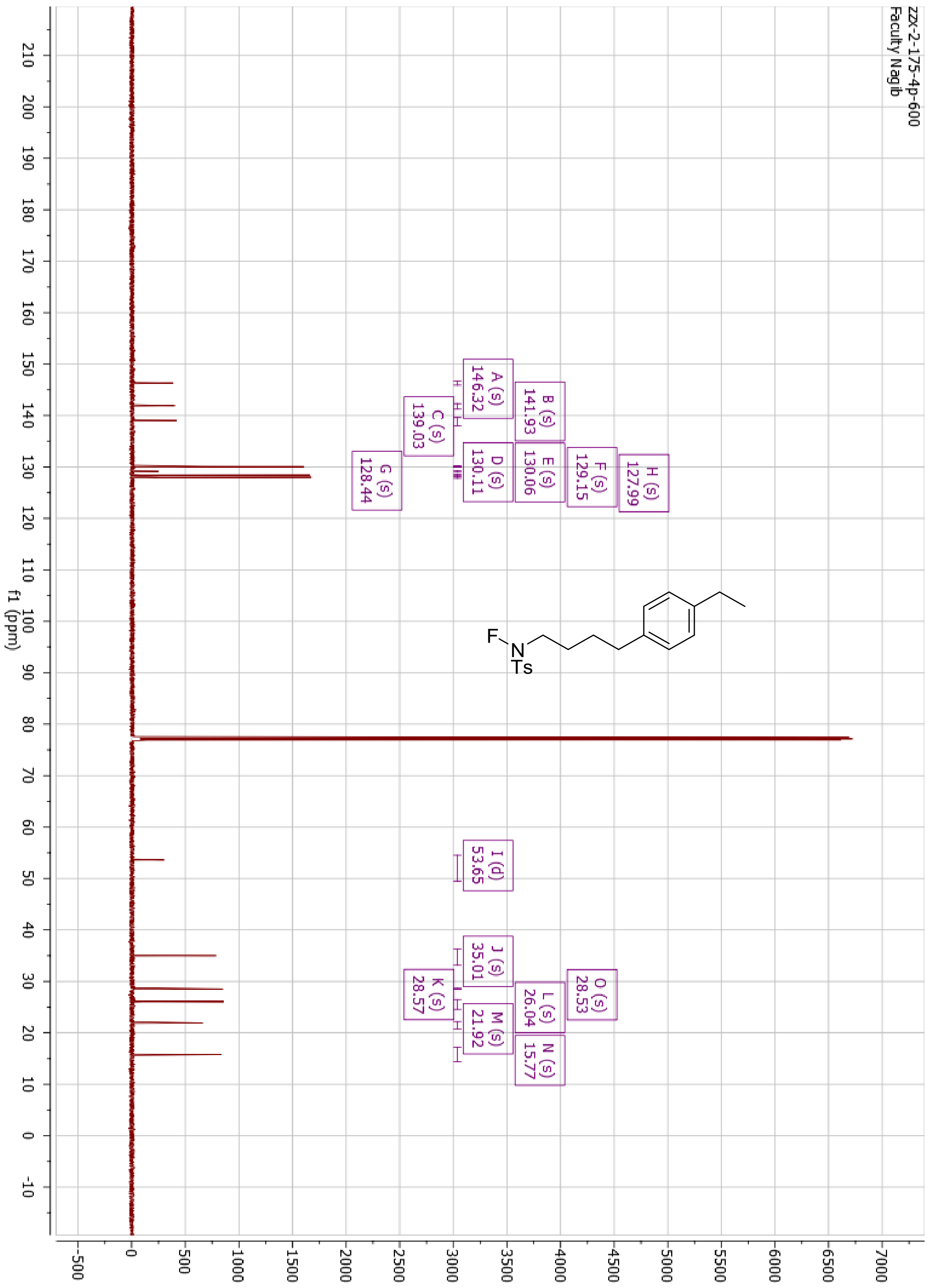
ZZX-2-175-4p-600
Faculty Nagib
A (s) 146.32
B (s) 141.93
C (s) 139.03
D (s) 130.11
E (s) 130.06
F (s) 129.15
G (s) 128.44
H (s) 127.99
I (d) 53.65
J (s) 35.01
K (s) 28.57
L (s) 26.04
M (s) 21.92
N (s) 15.77
O (s) 28.53
F
N
Ts
f1 (ppm)

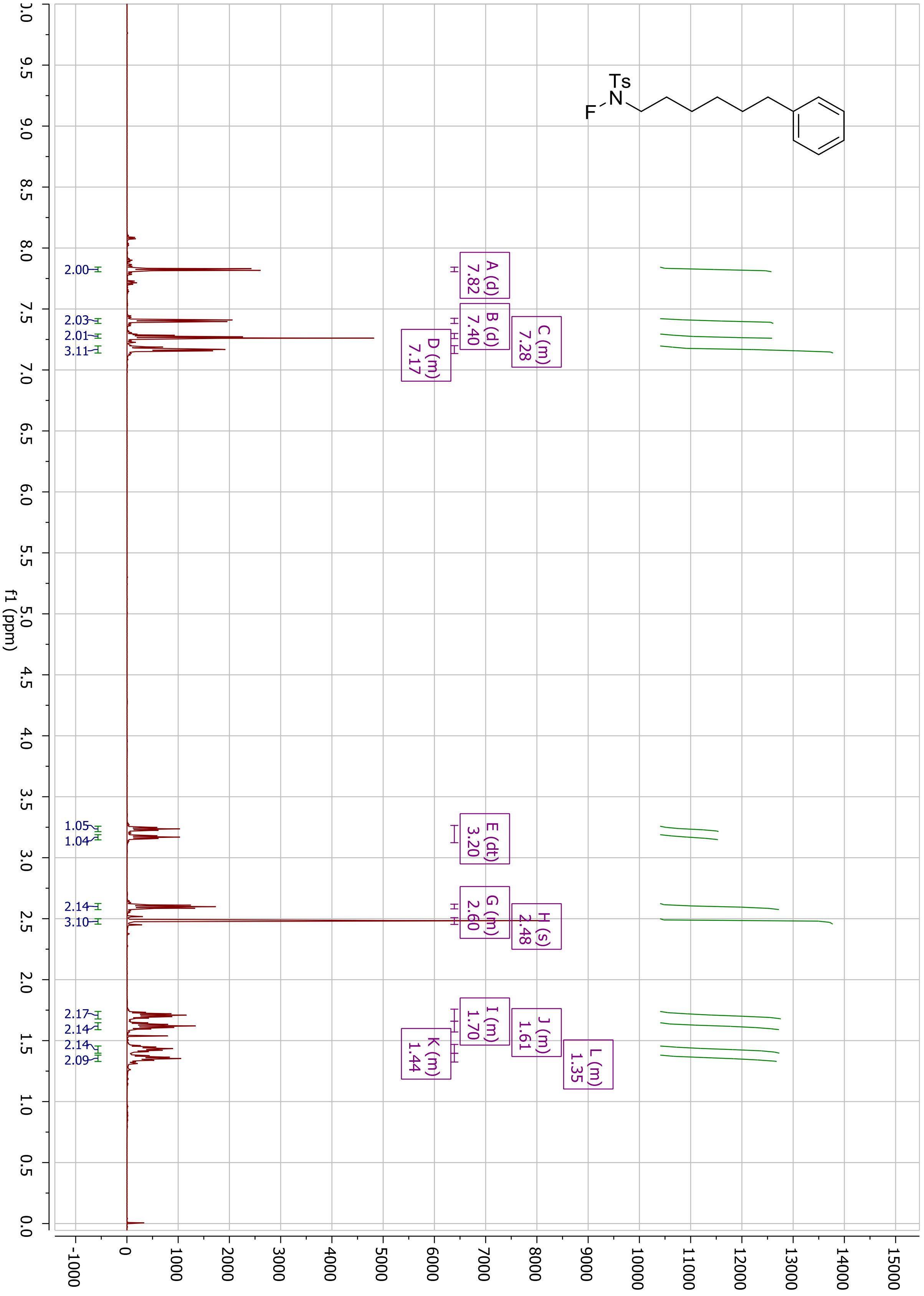

Ts
N
F
f1 (ppm)
A (d) 7.82
B (d) 7.40
C (m) 7.28
D (m) 7.17
E (dt) 3.20
G (m) 2.60
H (s) 2.48
I (m) 1.70
J (m) 1.61
K (m) 1.44
L (m) 1.35
2.00
2.03
2.01
3.11
1.05
1.04
2.14
3.10
2.17
2.14
2.14
2.09

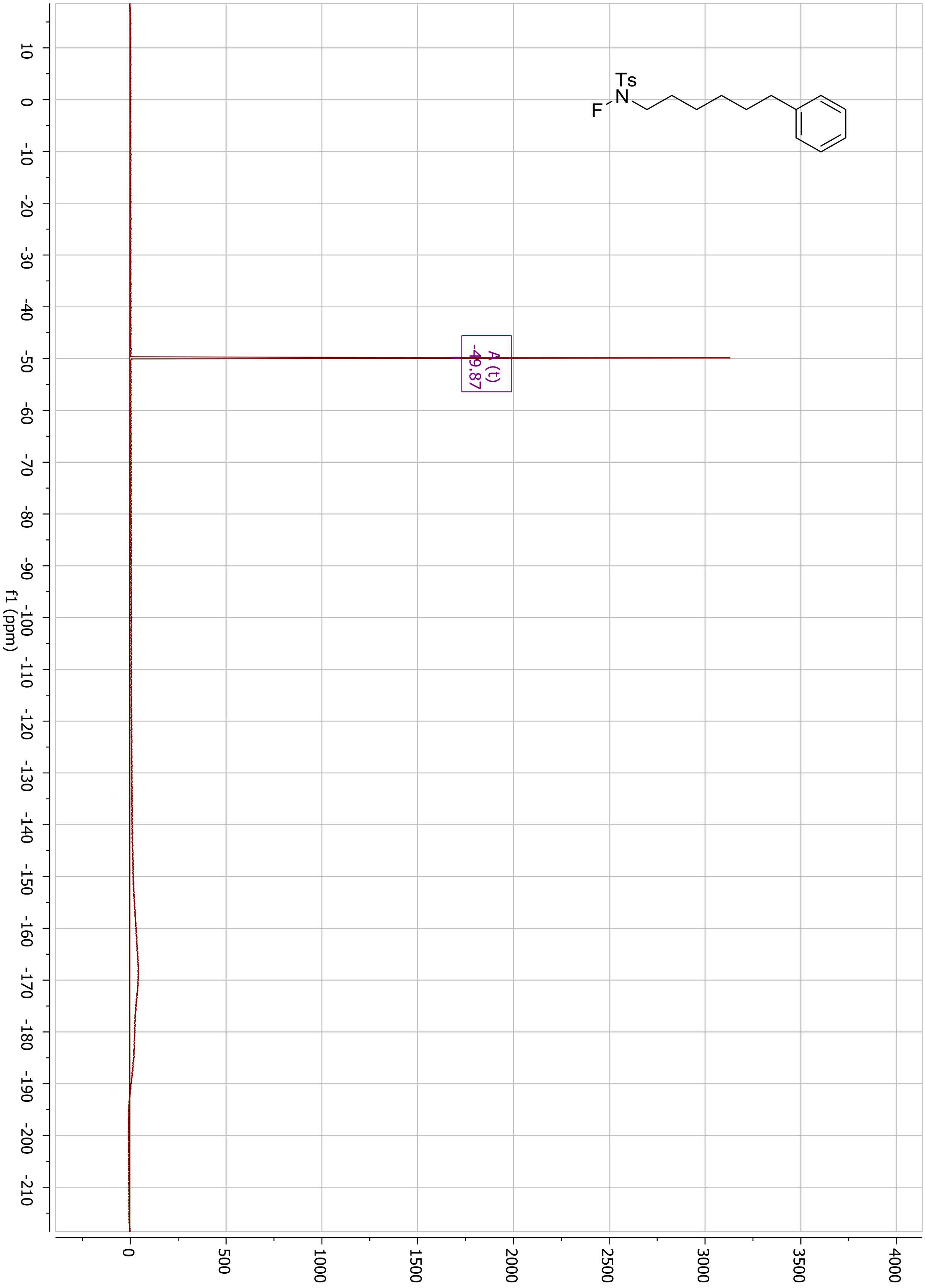
Ts
N
F
A (t)
-49.87
f1 (ppm)

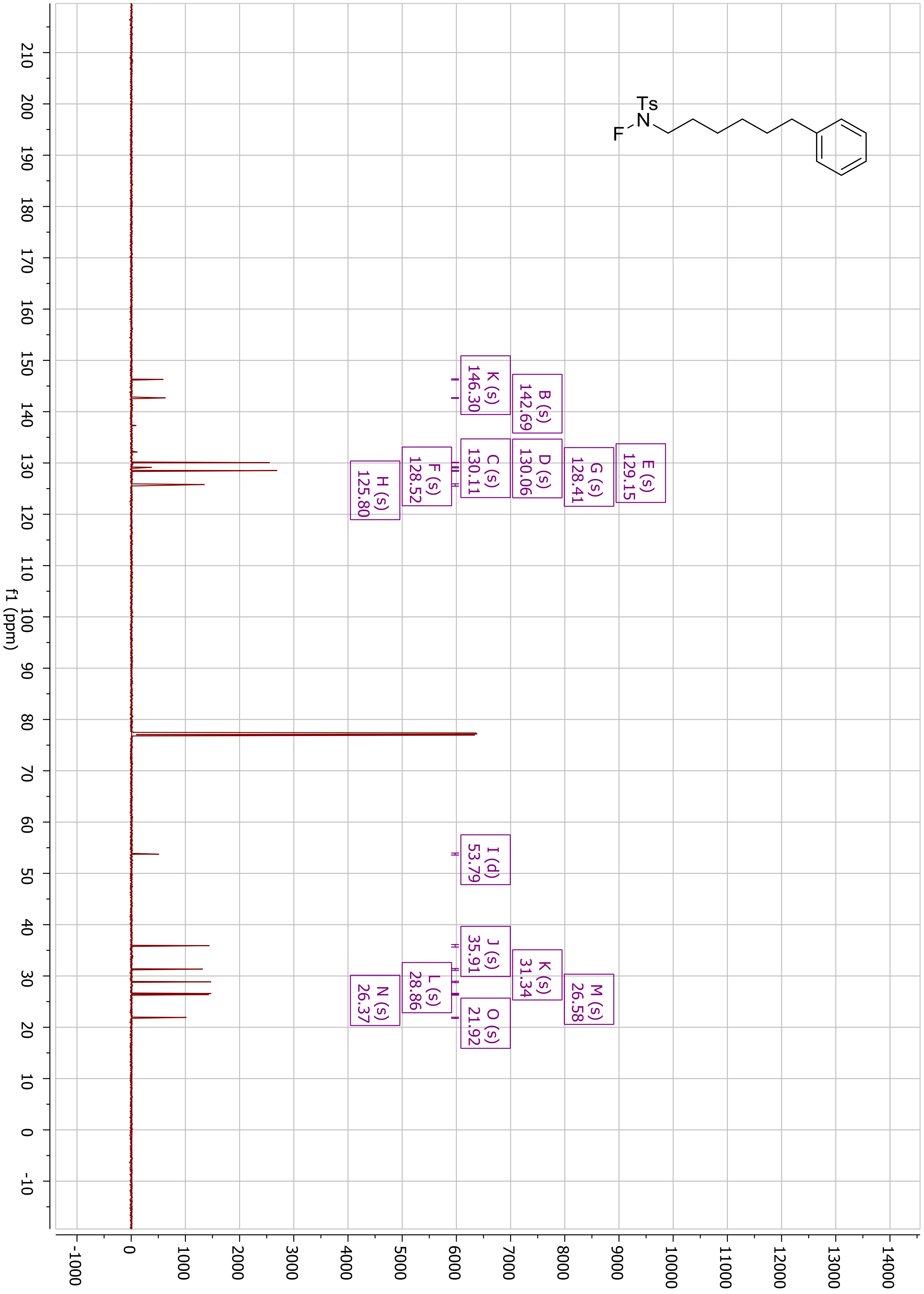
Ts
N
F
K (s) 146.30
B (s) 142.69
C (s) 130.11
D (s) 130.06
E (s) 129.15
F (s) 128.52
G (s) 128.41
H (s) 125.80
I (d) 53.79
J (s) 35.91
K (s) 31.34
L (s) 28.86
M (s) 26.58
N (s) 26.37
O (s) 21.92
f1 (ppm)

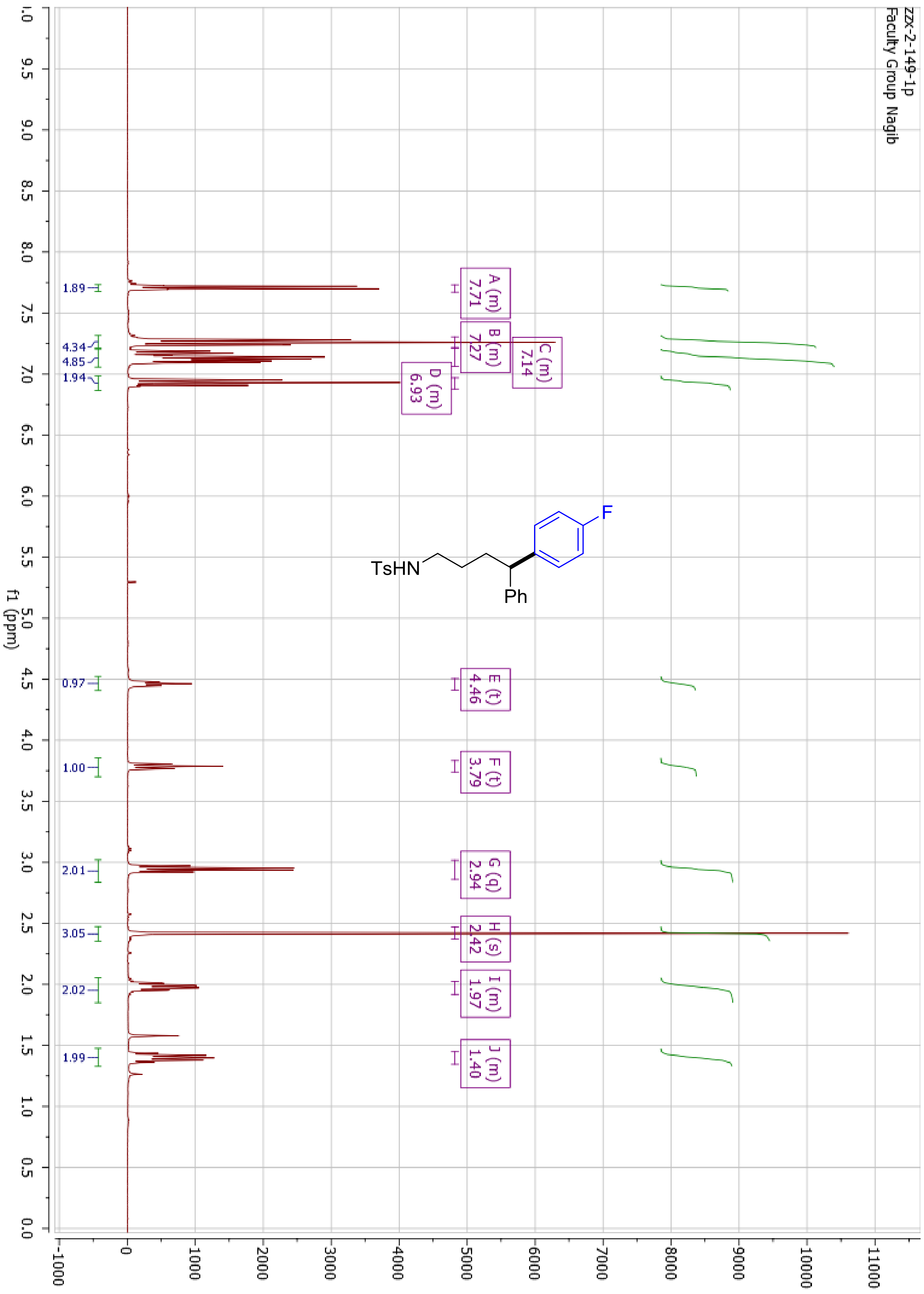
zzx-2-149-1p
Faculty Group Nagib
A (m) 7.71
B (m) 7.27
C (m) 7.14
D (m) 6.93
E (t) 4.46
F (t) 3.79
G (q) 2.94
H (s) 2.42
I (m) 1.97
J (m) 1.40
1.89
4.34
4.85
1.94
0.97
1.00
2.01
3.05
2.02
1.99
TsHN
Ph
F
f1 (ppm)

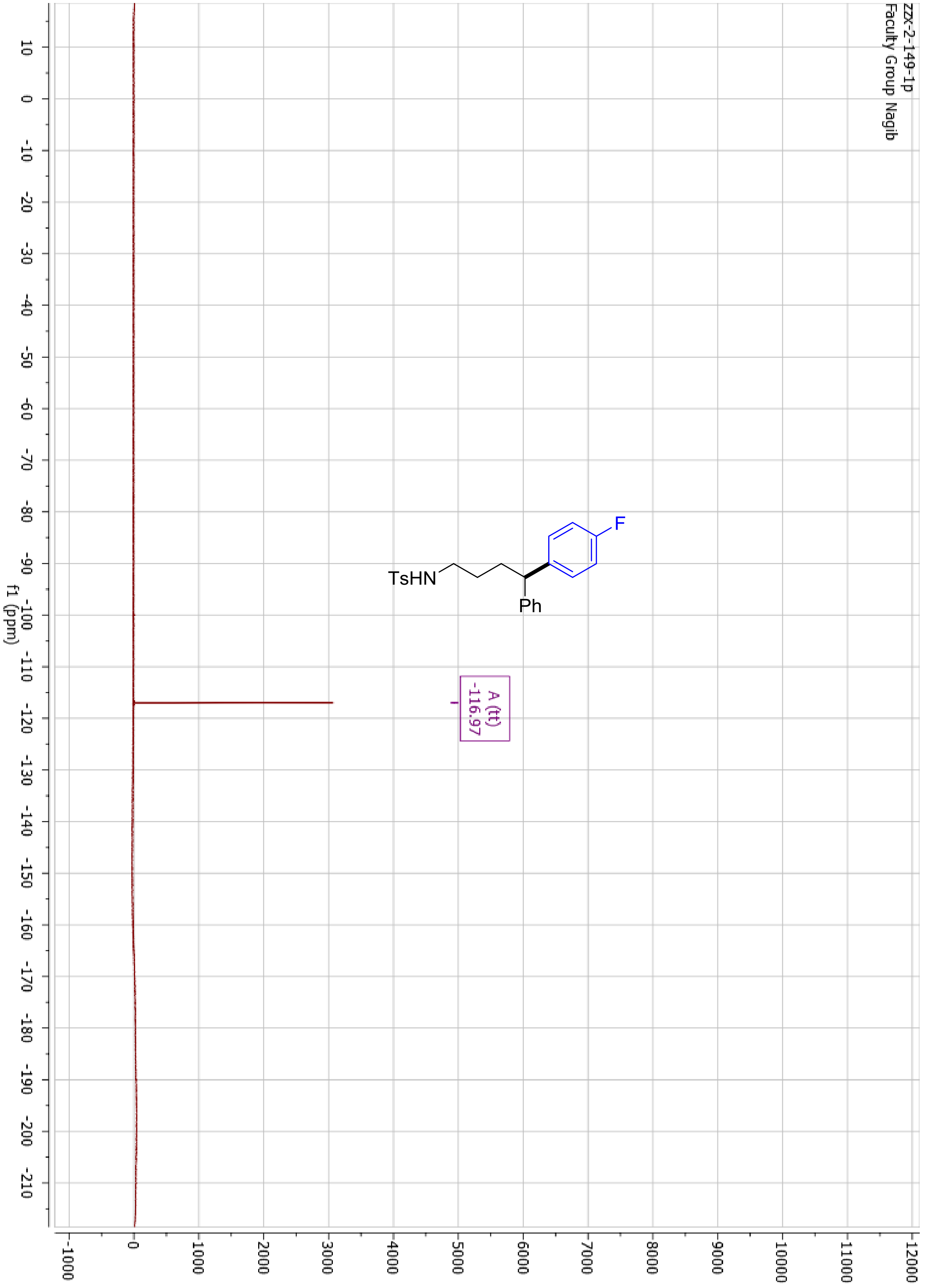
zzx-2-149-1p
Faculty Group Nagib
TsHN
Ph
F
A (tt)
-116.97
f1 (ppm)

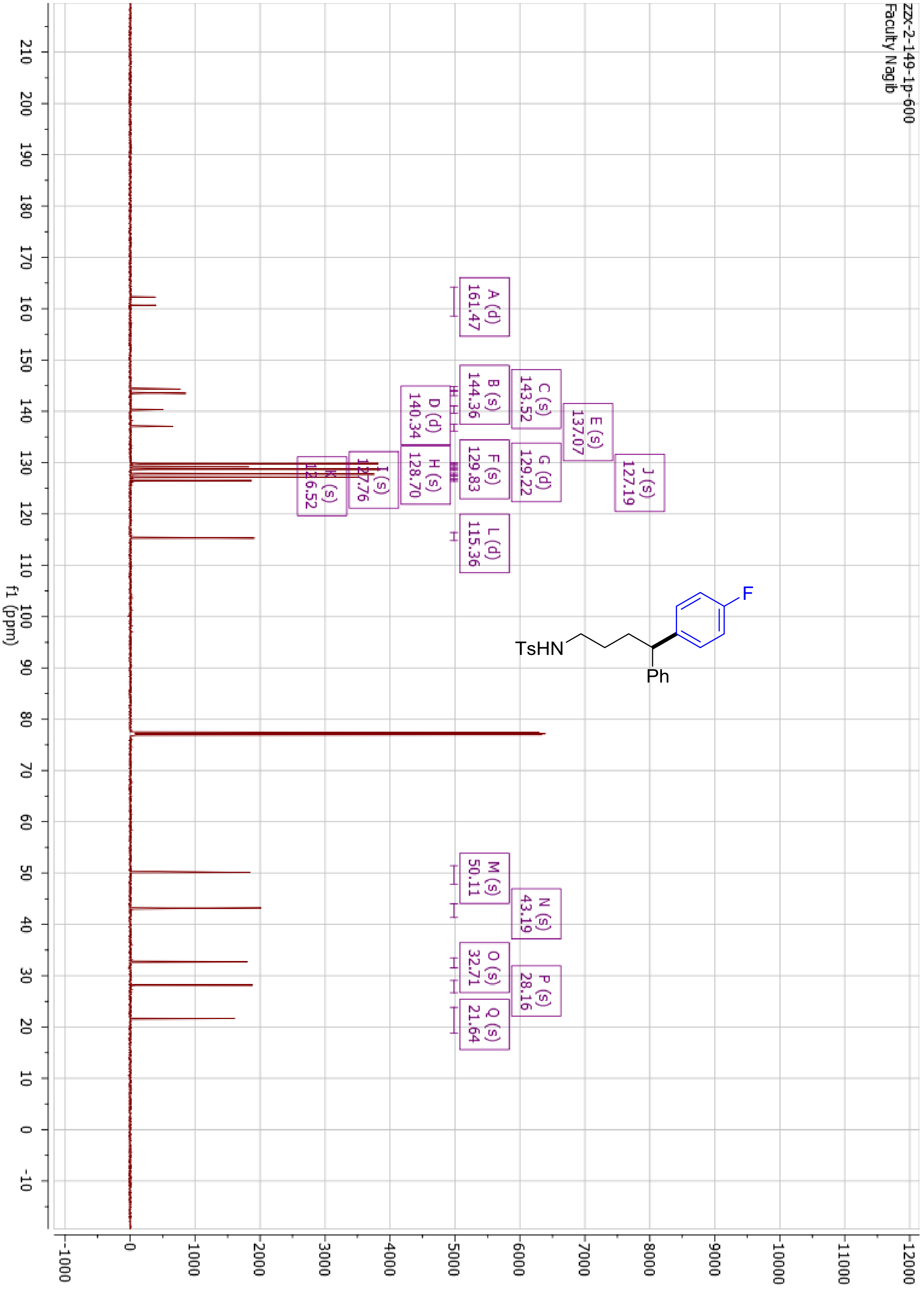

ZZX-2-149-1p-600
Faculty Nagib
A (d) 161.47
B (s) 144.36
C (s) 143.52
D (d) 140.34
E (s) 137.07
F (s) 129.83
G (d) 129.22
H (s) 128.70
I (s) 127.76
J (s) 127.19
K (s) 126.52
L (d) 115.36
M (s) 50.11
N (s) 43.19
O (s) 32.71
P (s) 28.16
Q (s) 21.64
TsHN
Ph
F
f1 (ppm)

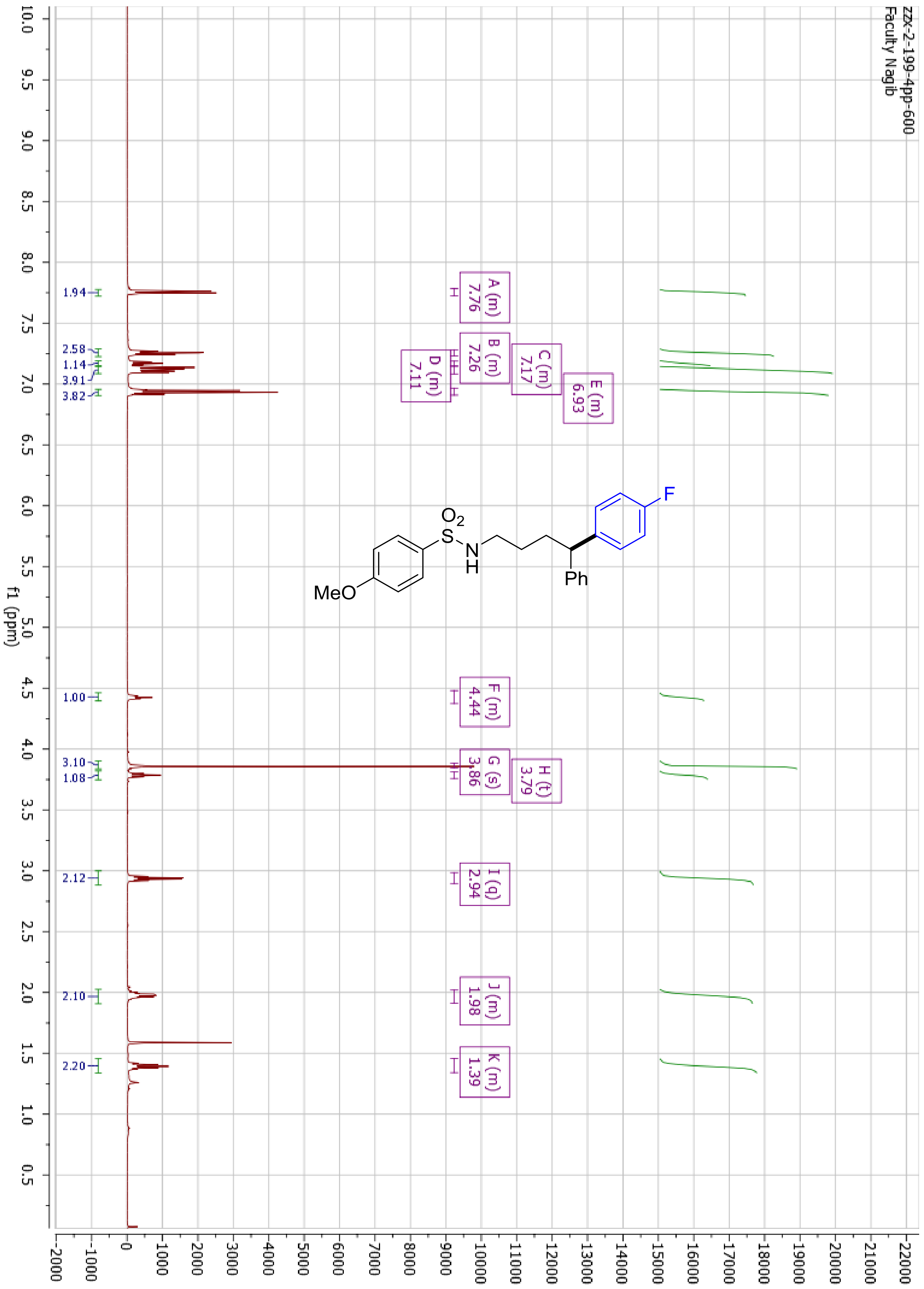
zzx-2-199-4pp-600
Faculty Nagib
A (m) 7.76
B (m) 7.26
C (m) 7.17
D (m) 7.11
E (m) 6.93
F (m) 4.44
G (s) 3.86
H (t) 3.79
I (q) 2.94
J (m) 1.98
K (m) 1.39
1.94
2.58
1.14
3.91
3.82
1.00
3.10
1.08
2.12
2.10
2.20
MeO
O2
S
N
H
Ph
F
f1 (ppm)

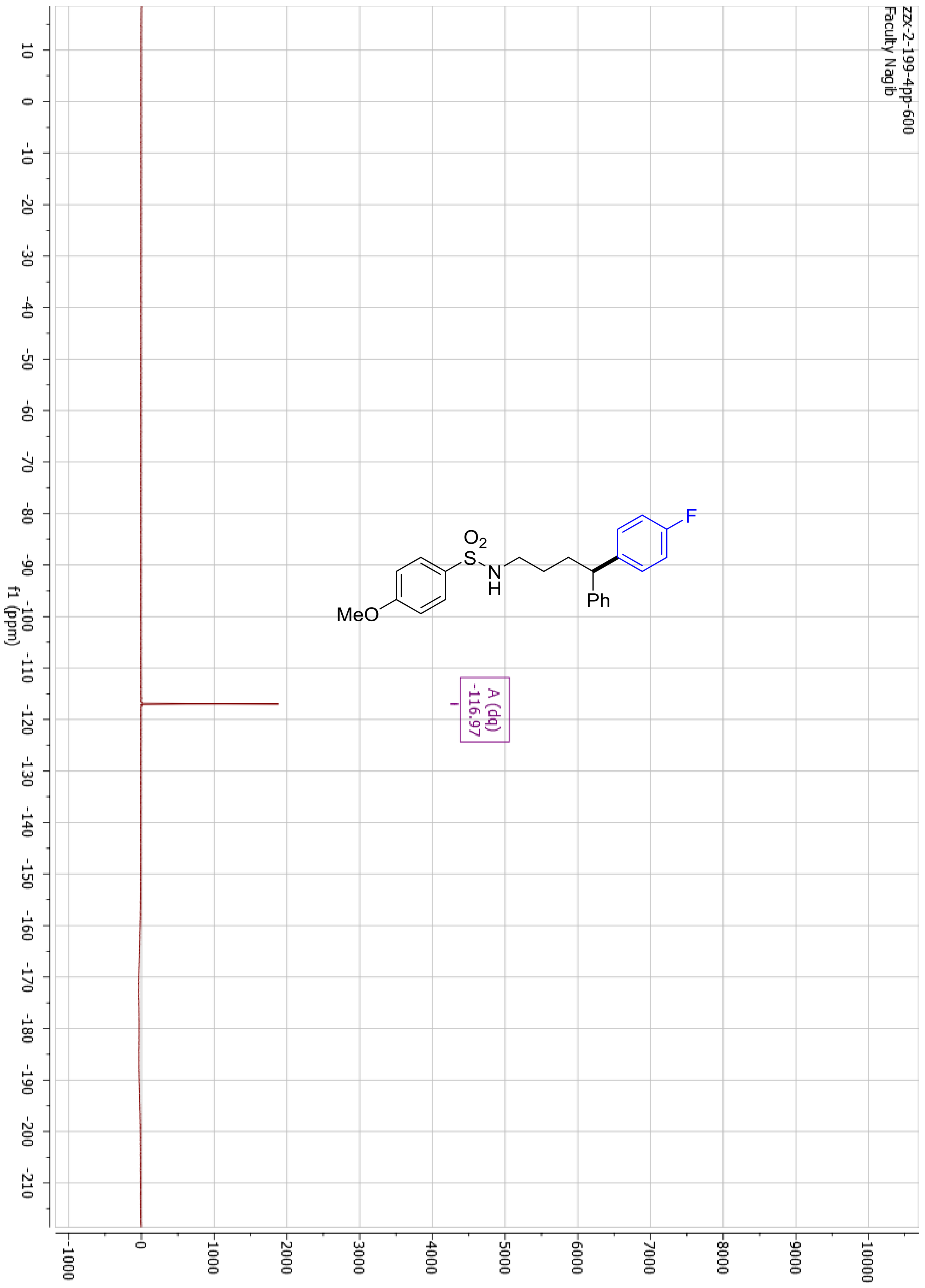
zzx-2-199-4pp-600
Faculty Nagib
MeO
O_2
S
N
H
Ph
F
A (dq)
-116.97
f1 (ppm)

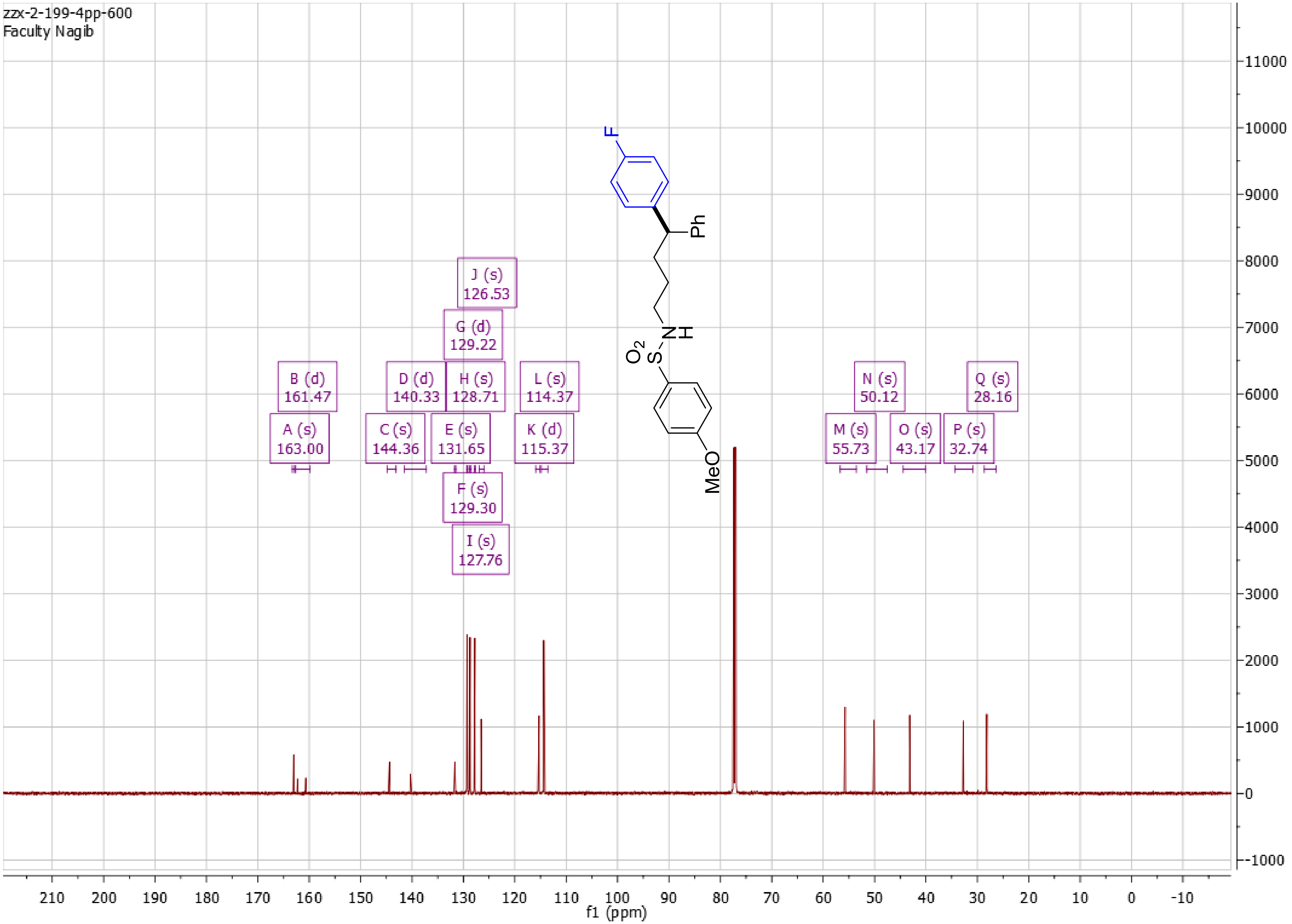
zzx-2-199-4pp-600
Faculty Nagib
A (s)
163.00
B (d)
161.47
C (s)
144.36
D (d)
140.33
E (s)
131.65
F (s)
129.30
G (d)
129.22
H (s)
128.71
I (s)
127.76
J (s)
126.53
K (d)
115.37
L (s)
114.37
M (s)
55.73
N (s)
50.12
O (s)
43.17
P (s)
32.74
Q (s)
28.16
F
Ph
O_2
S
N
H
MeO
f1 (ppm)

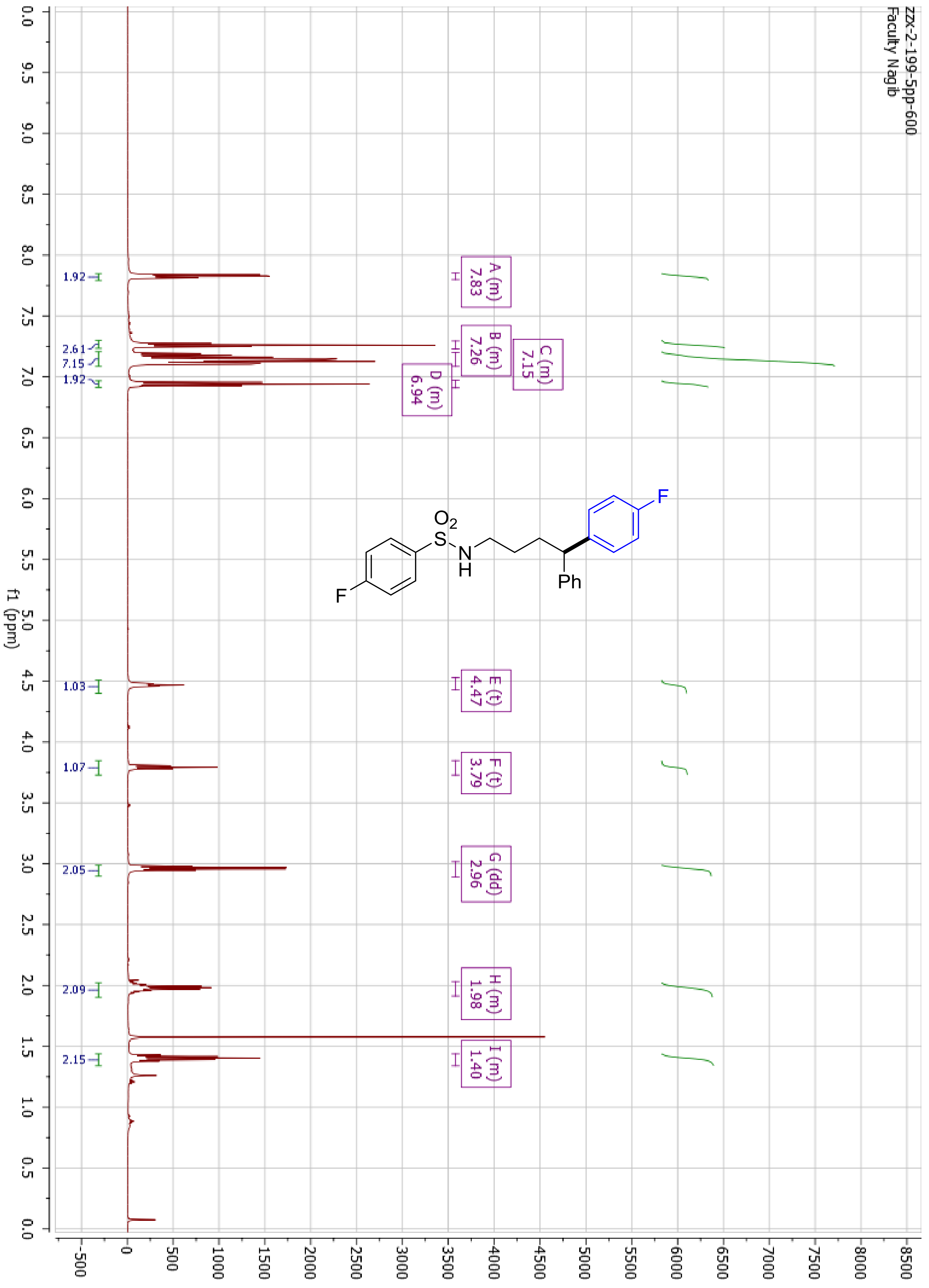

zzx-2-199-5pp-600
Faculty Nagib
A (m) 7.83
B (m) 7.26
C (m) 7.15
D (m) 6.94
E (t) 4.47
F (t) 3.79
G (dd) 2.96
H (m) 1.98
I (m) 1.40
1.92
2.61
7.15
1.92
1.03
1.07
2.05
2.09
2.15
f1 (ppm)

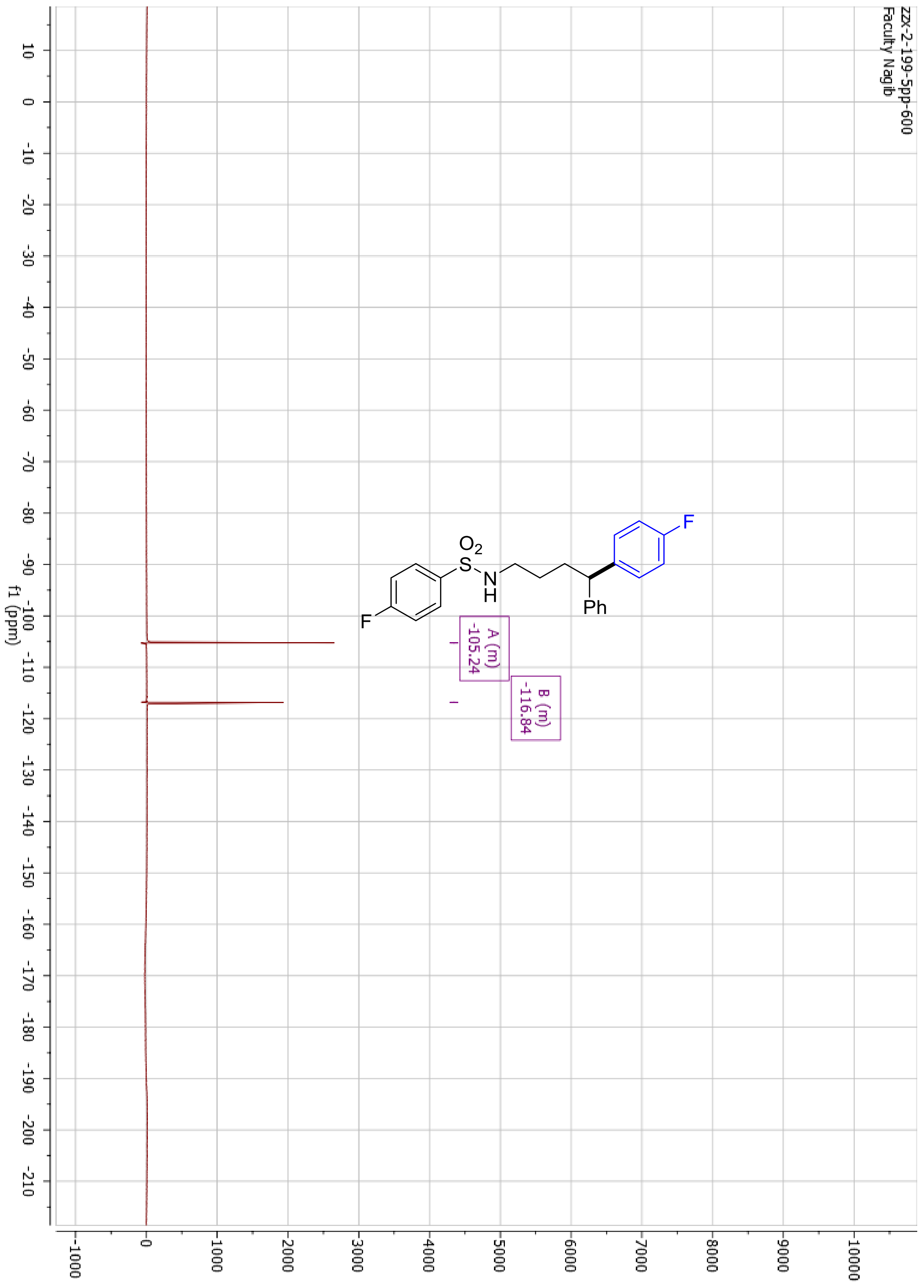
ZZX-2-199-5pp-600
Faculty Nagib
A (m)
-105.24
B (m)
-116.84
f1 (ppm)

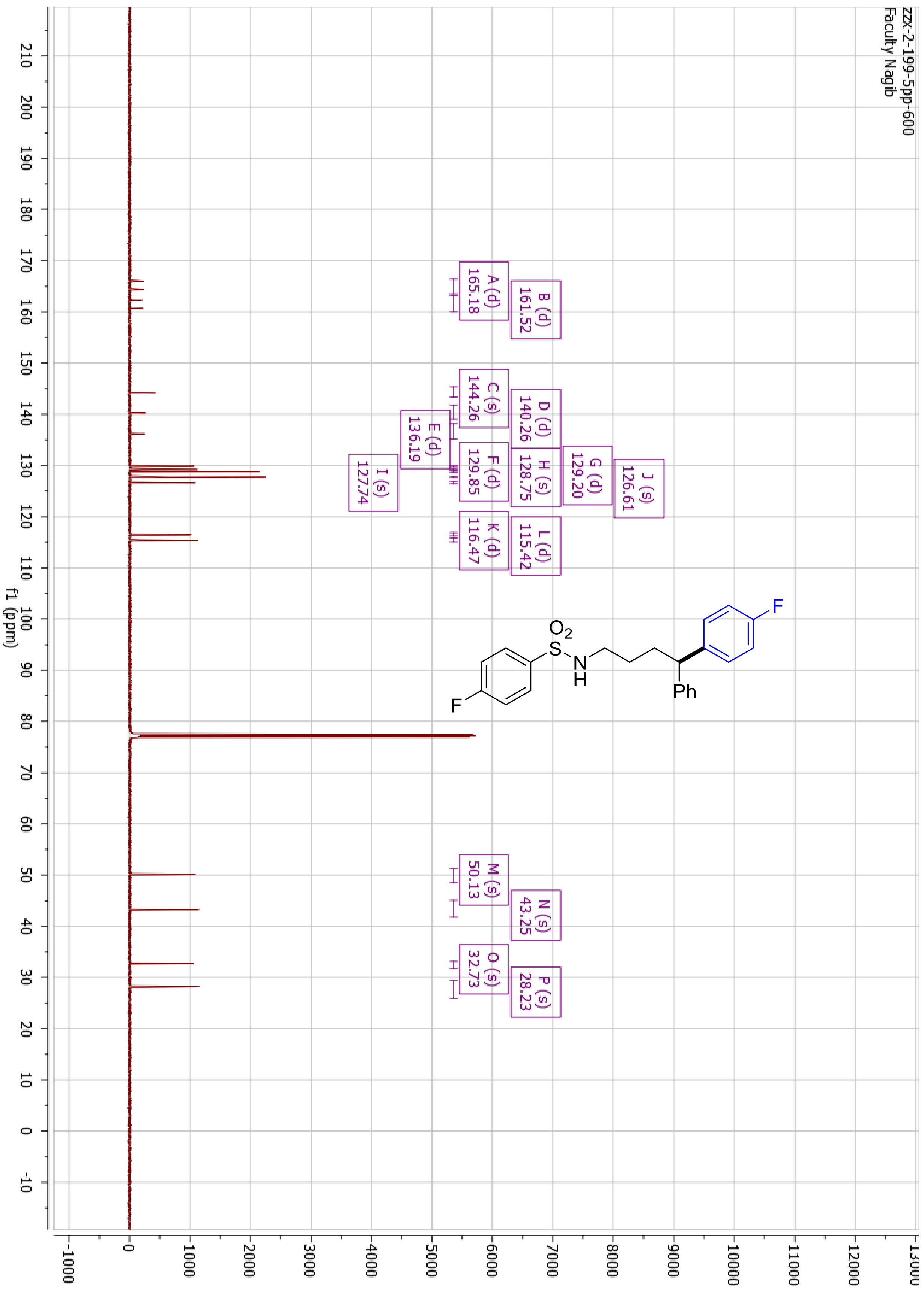

zzx-2-199-5pp-600
Faculty Nagib
A (d) 165.18
B (d) 161.52
C (s) 144.26
D (d) 140.26
E (d) 136.19
F (d) 129.85
G (d) 129.20
H (s) 128.75
I (s) 127.74
J (s) 126.61
K (d) 116.47
L (d) 115.42
M (s) 50.13
N (s) 43.25
O (s) 32.73
P (s) 28.23
f1 (ppm)

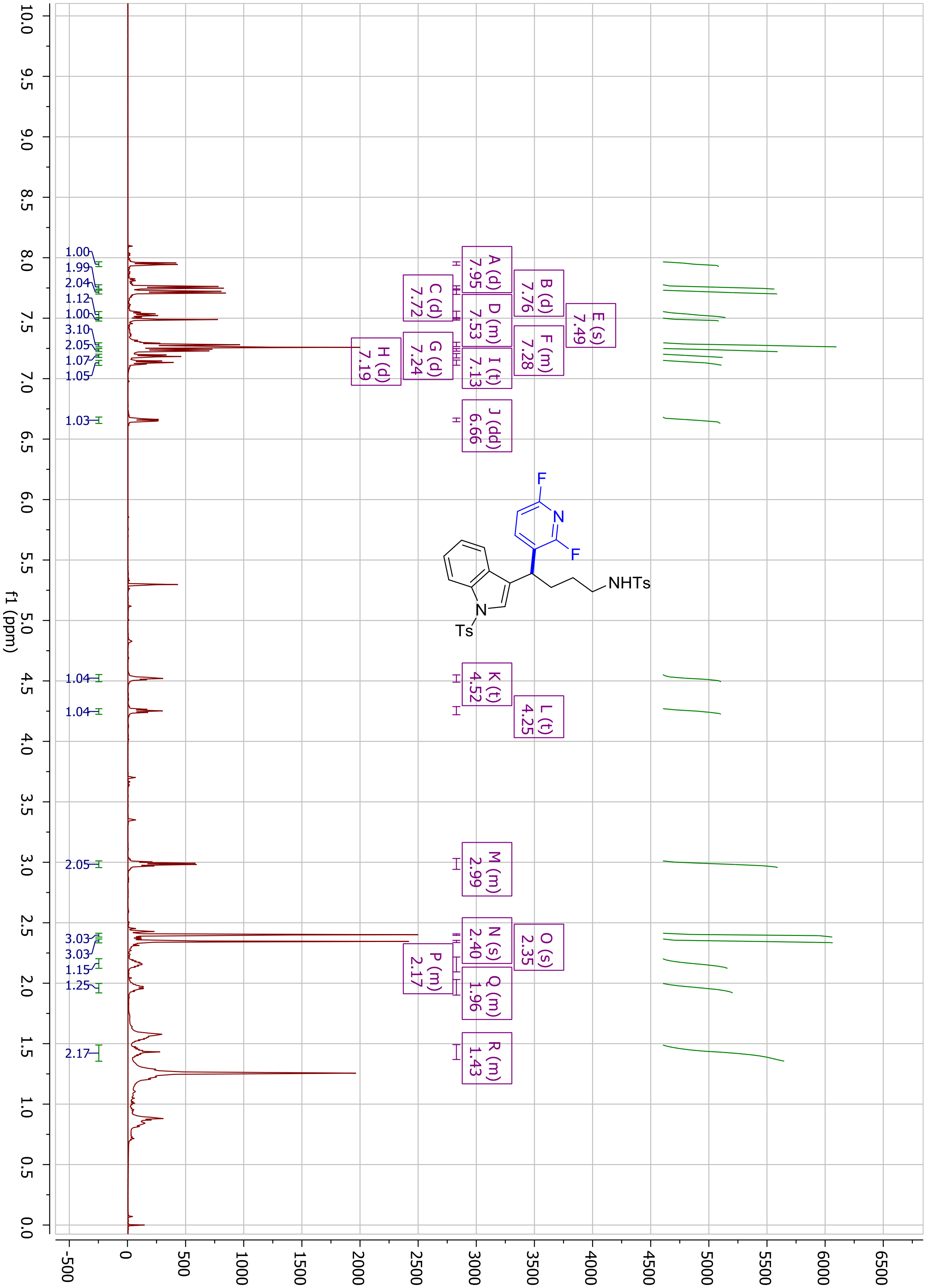
f1 (ppm)
A (d) 7.95
B (d) 7.76
C (d) 7.72
D (m) 7.53
E (s) 7.49
F (m) 7.28
G (d) 7.24
H (d) 7.19
I (t) 7.13
J (dd) 6.66
K (t) 4.52
L (t) 4.25
M (m) 2.99
N (s) 2.40
O (s) 2.35
P (m) 2.17
Q (m) 1.96
R (m) 1.43
NHTs
Ts
N
F

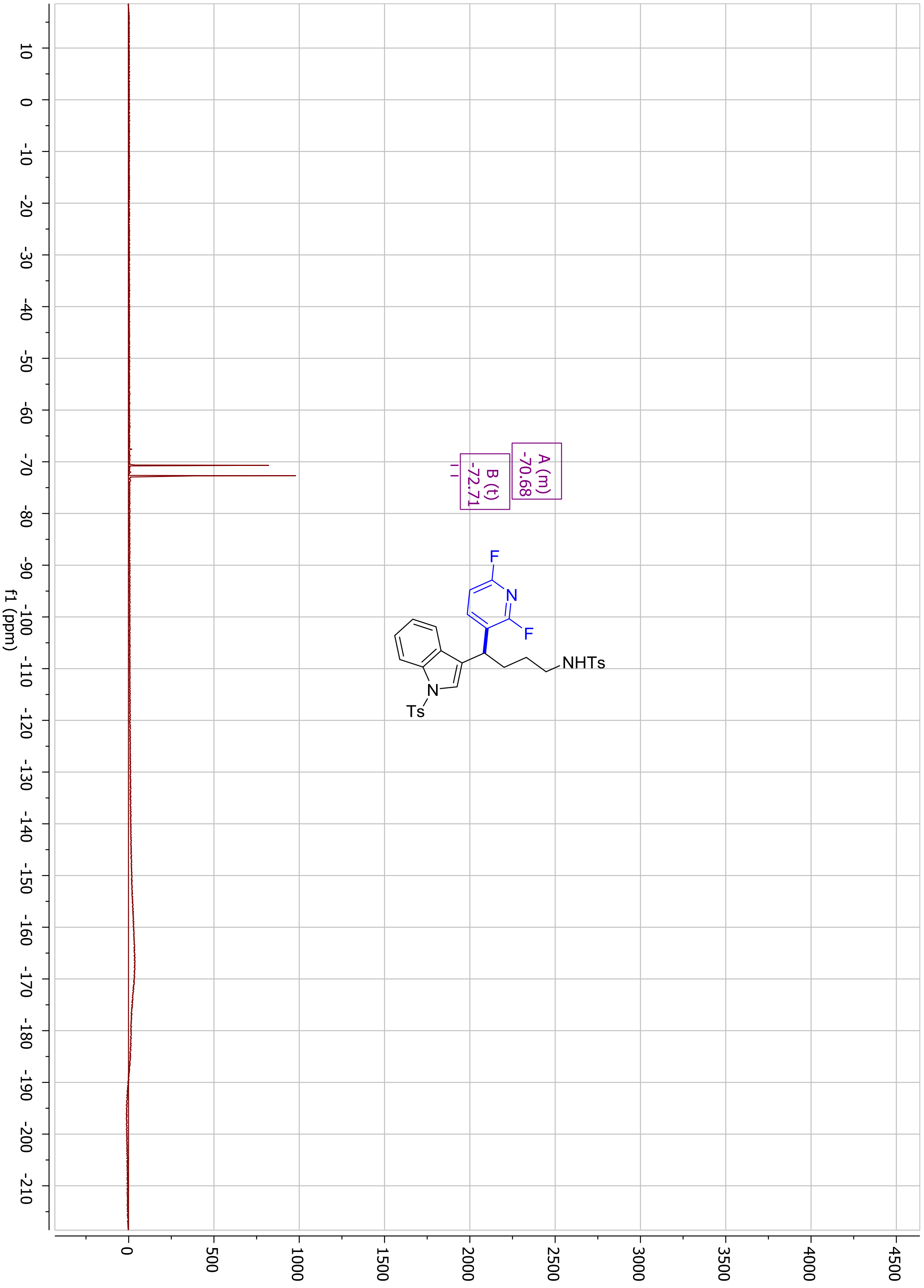
A (m)
-70.68
B (t)
-72.71
F
N
F
NHTs
N
Ts
f1 (ppm)

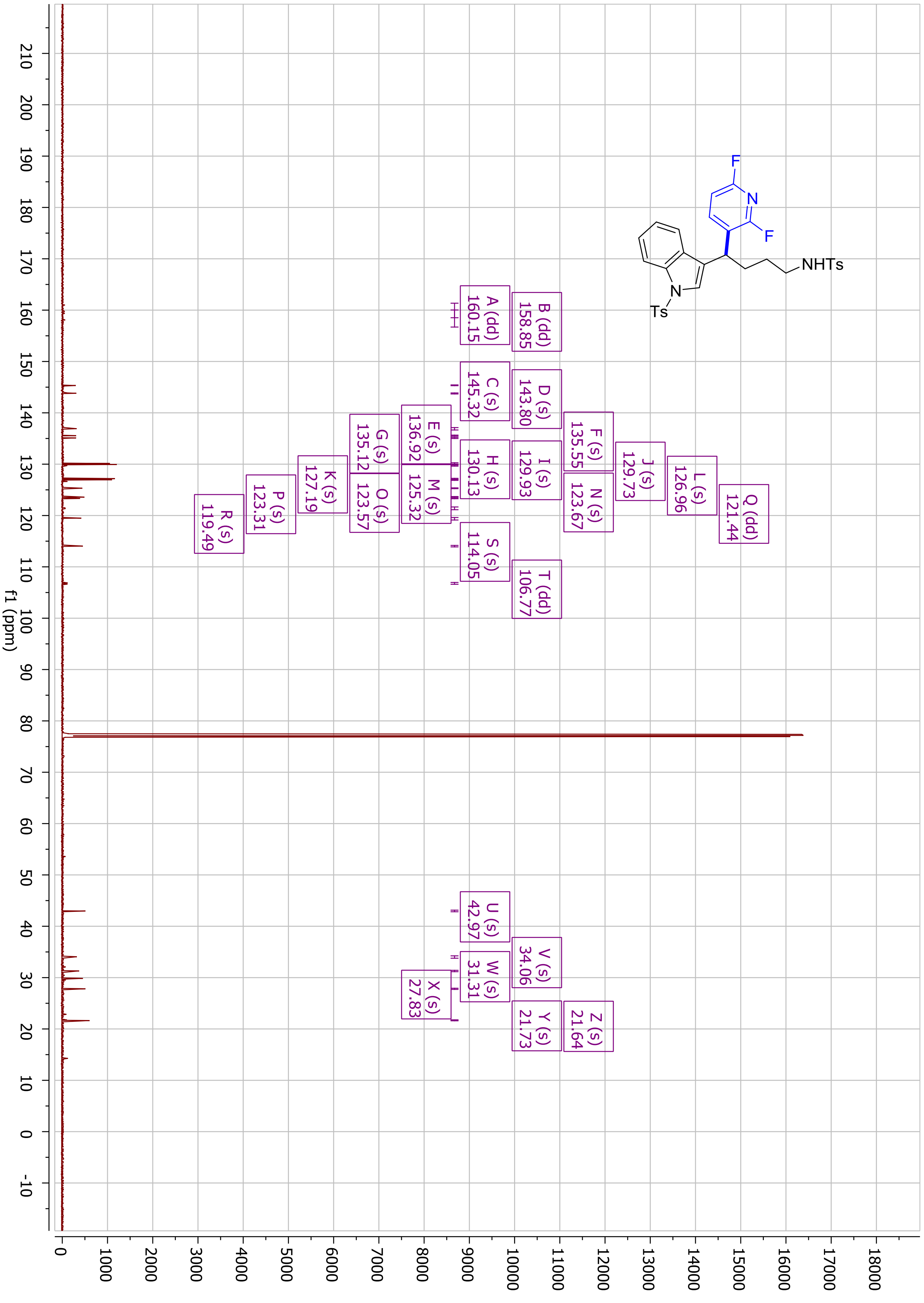
A (dd) 160.15
B (dd) 158.85
C (s) 145.32
D (s) 143.80
E (s) 136.92
F (s) 135.55
G (s) 135.12
H (s) 130.13
I (s) 129.93
J (s) 129.73
K (s) 127.19
L (s) 126.96
M (s) 125.32
N (s) 123.67
O (s) 123.57
P (s) 123.31
Q (dd) 121.44
R (s) 119.49
S (s) 114.05
T (dd) 106.77
U (s) 42.97
V (s) 34.06
W (s) 31.31
X (s) 27.83
Y (s) 21.73
Z (s) 21.64
f1 (ppm)
NHTs
Ts
N
F

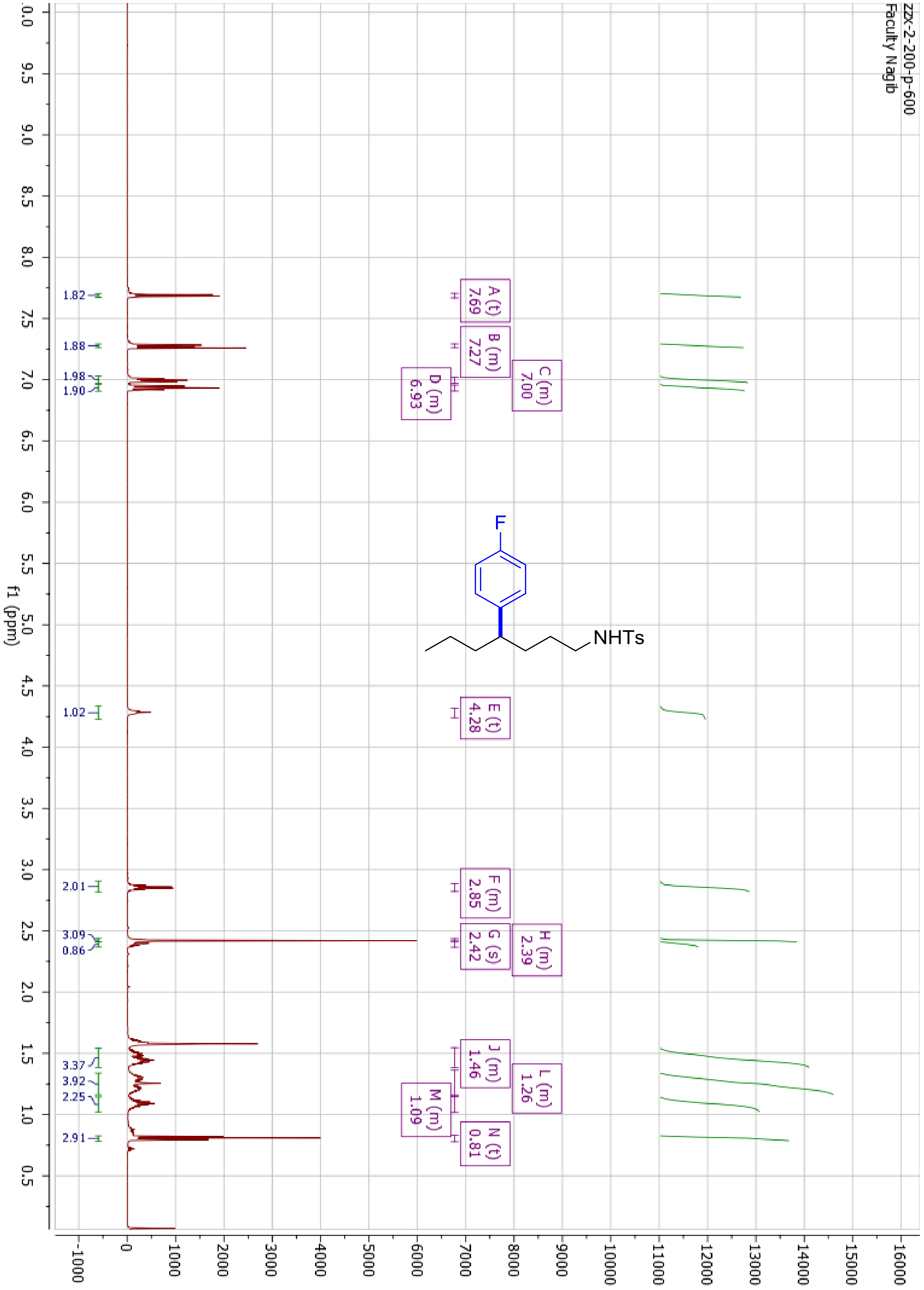
ZZX-2-200-p-600
Faculty Nagib
NHTs
F
A (t) 7.69
B (m) 7.27
C (m) 7.00
D (m) 6.93
E (t) 4.28
F (m) 2.85
G (s) 2.42
H (m) 2.39
J (m) 1.46
L (m) 1.26
M (m) 1.09
N (t) 0.81
1.82
1.88
1.98
1.90
1.02
2.01
3.09
0.86
3.37
3.92
2.25
2.91
f1 (ppm)

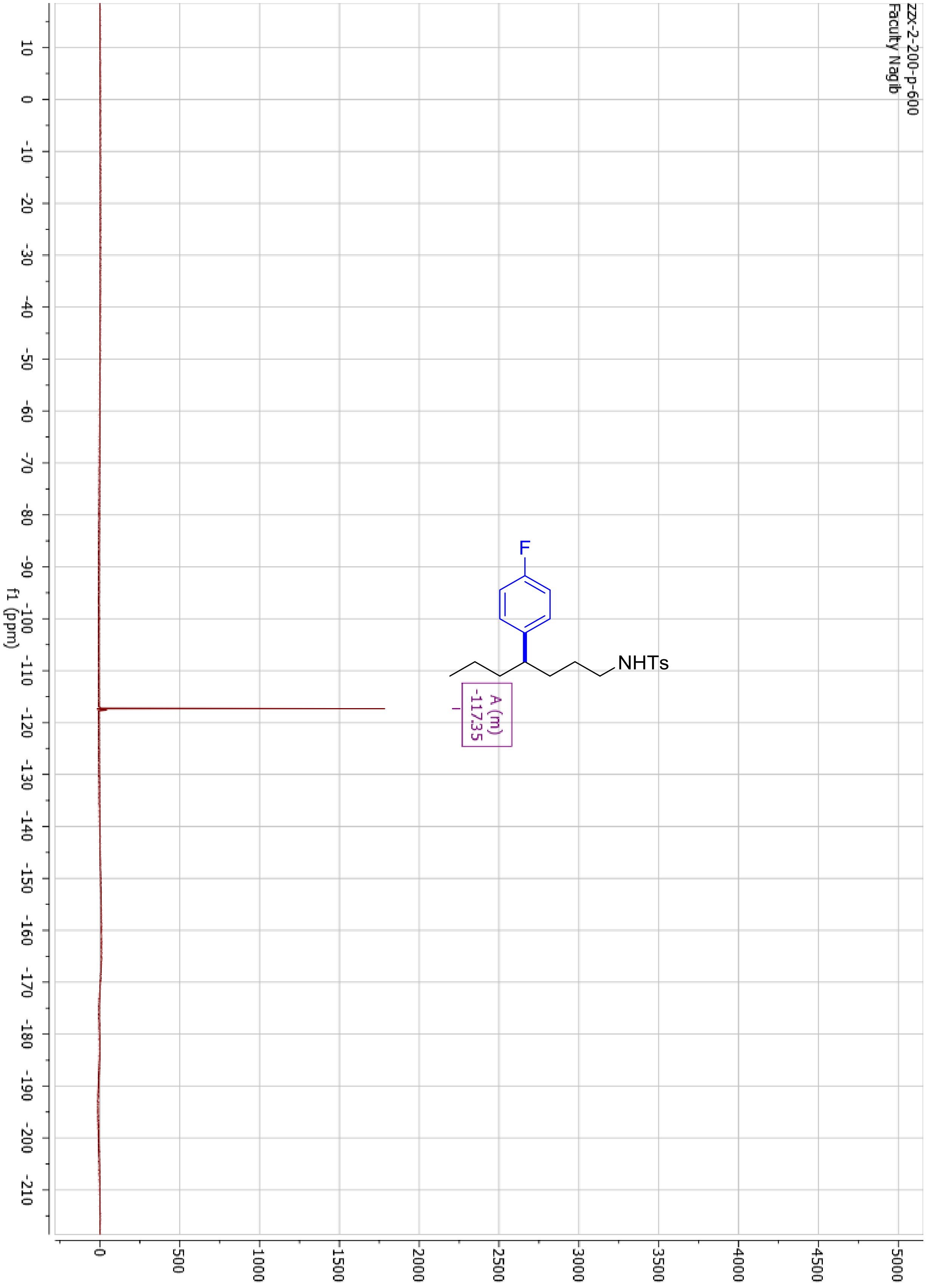
ZZX-2-200-p-600
Faculty Nagib
F
NHTs
A (m)
-117.35
f1 (ppm)

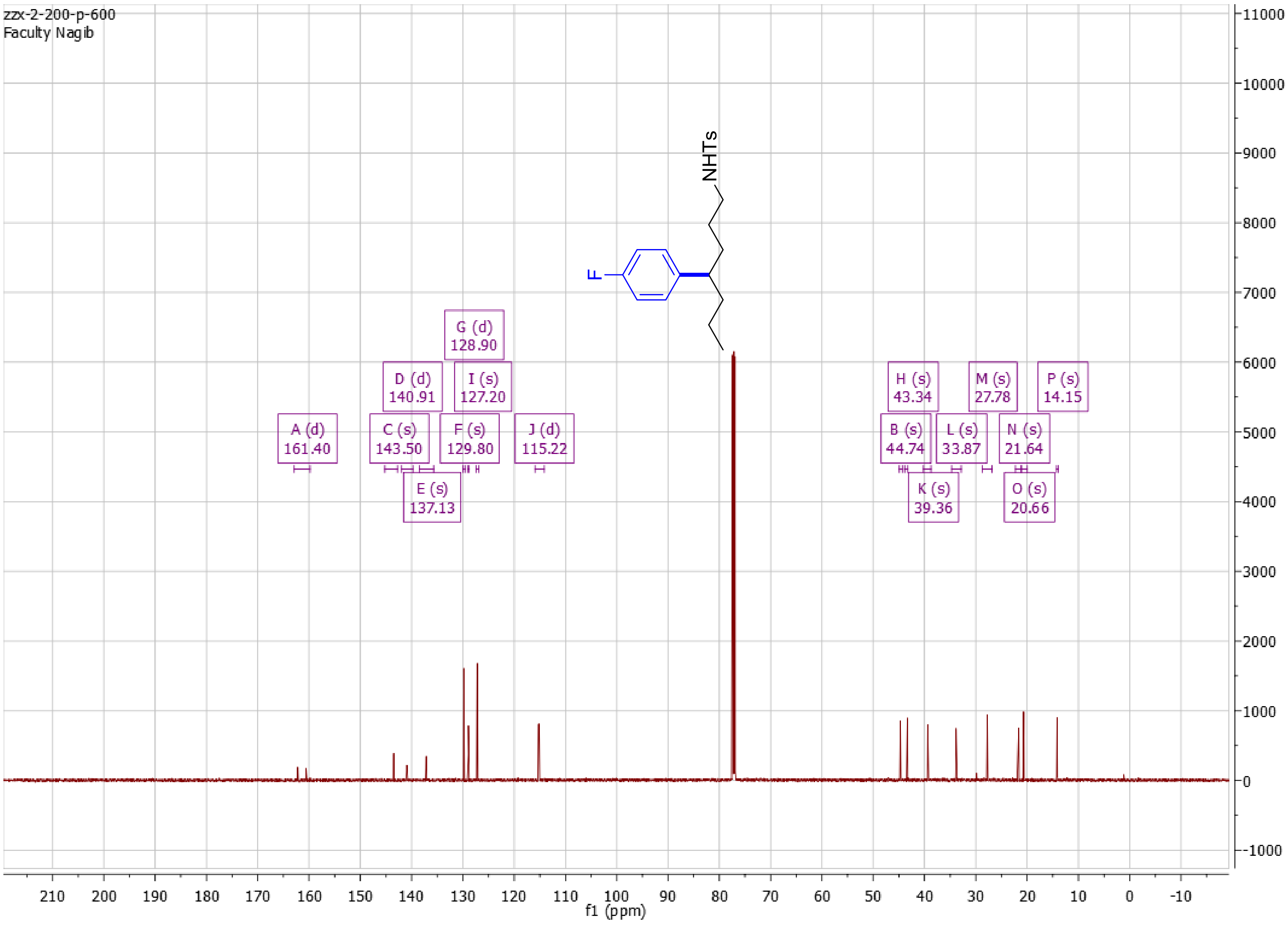
zzx-2-200-p-600
Faculty Nagib
NHTs
F
A (d) 161.40
C (s) 143.50
D (d) 140.91
E (s) 137.13
F (s) 129.80
G (d) 128.90
I (s) 127.20
J (d) 115.22
B (s) 44.74
H (s) 43.34
K (s) 39.36
L (s) 33.87
M (s) 27.78
N (s) 21.64
O (s) 20.66
P (s) 14.15
f1 (ppm)

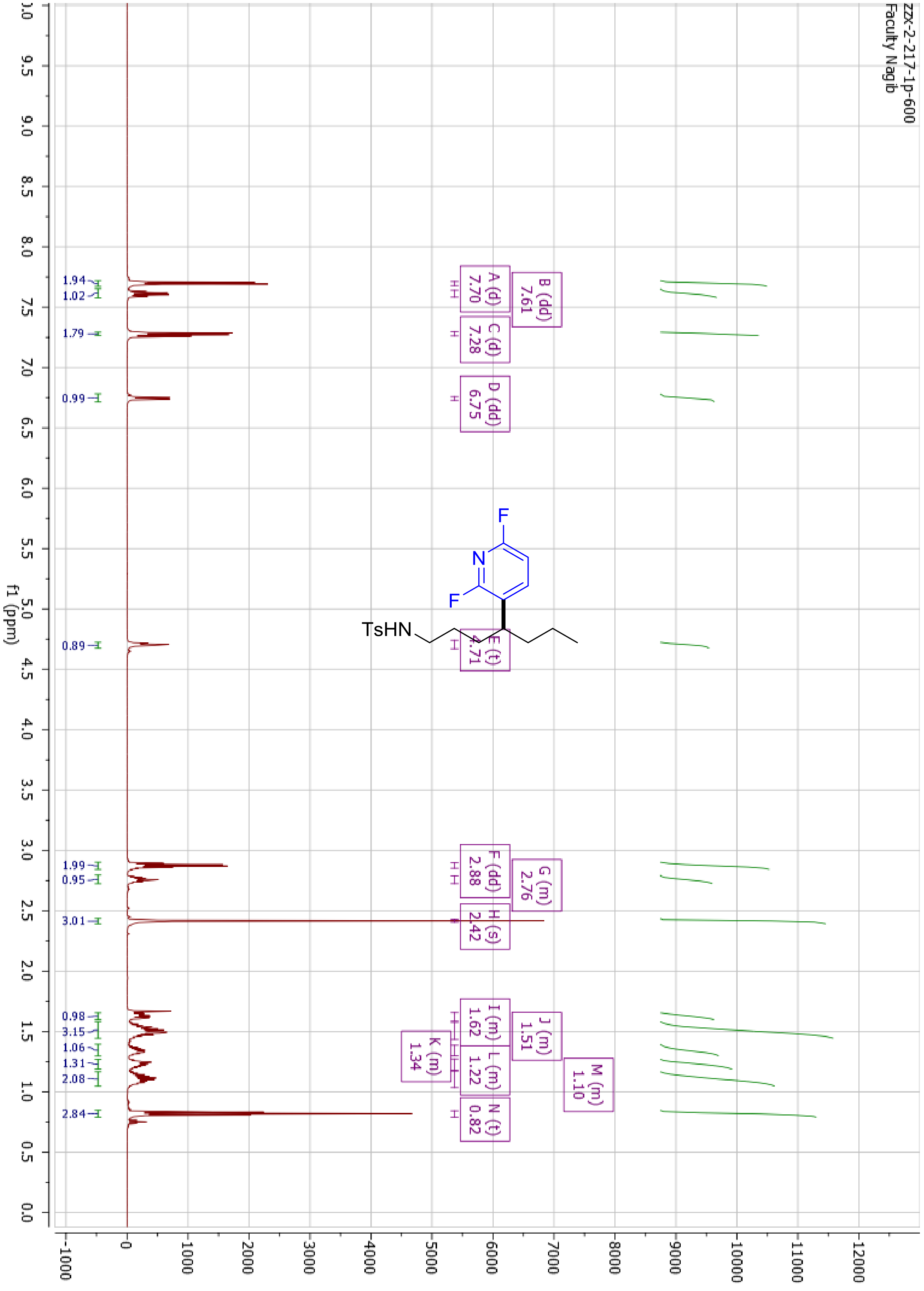
zzx-2-217-1p-600
Faculty Nagib
A (d) 7.70
B (dd) 7.61
C (d) 7.28
D (dd) 6.75
E (t) 4.71
F (dd) 2.88
G (m) 2.76
H (s) 2.42
I (m) 1.62
J (m) 1.51
K (m) 1.34
L (m) 1.22
M (m) 1.10
N (t) 0.82
TsHN
F
N
F
f1 (ppm)

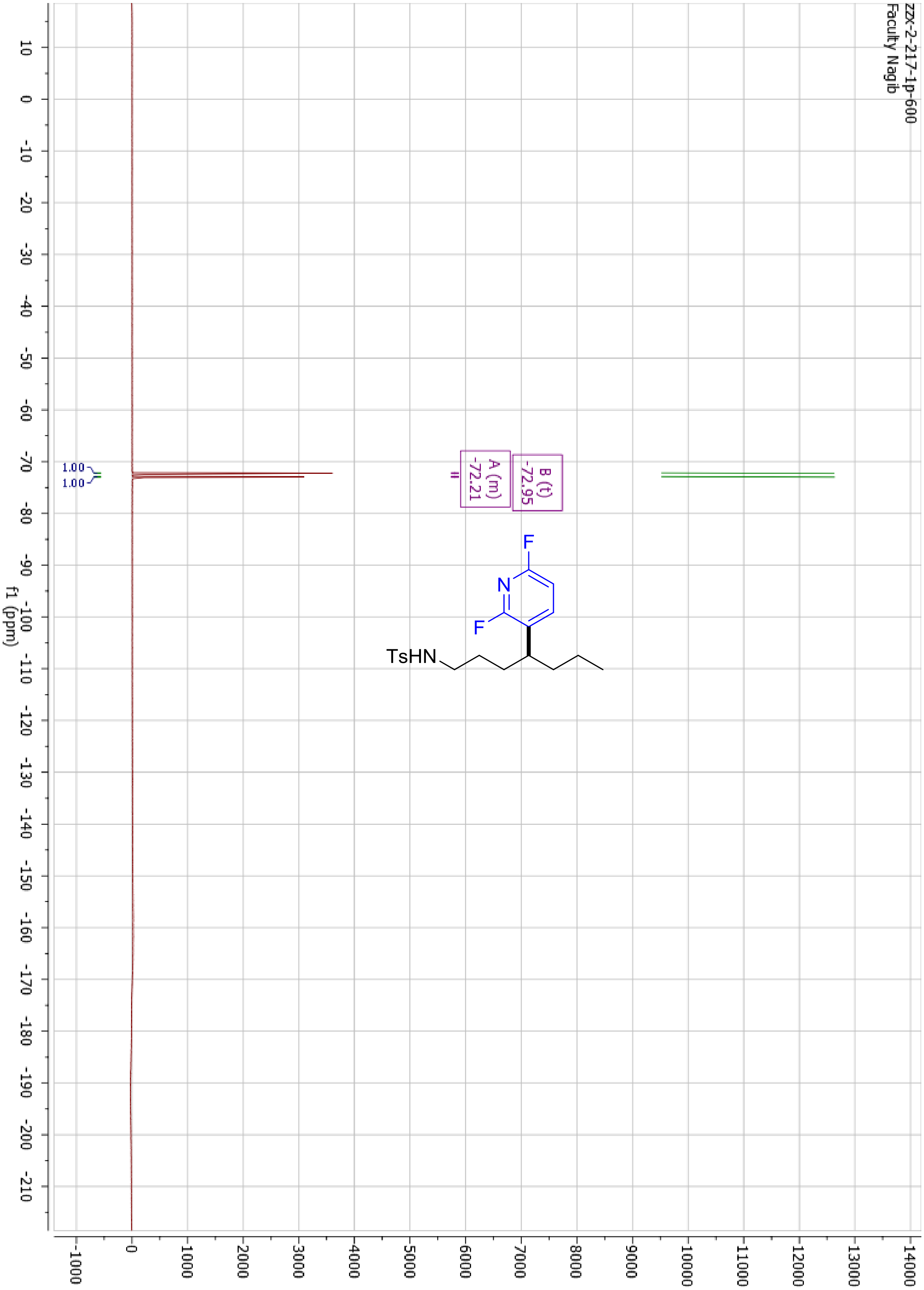

zzx-2-217-1p-600
Facuity Nagib
A (m)
-72.21
B (t)
-72.95
1.00
1.00
f1 (ppm)
TsHN
N
F
F

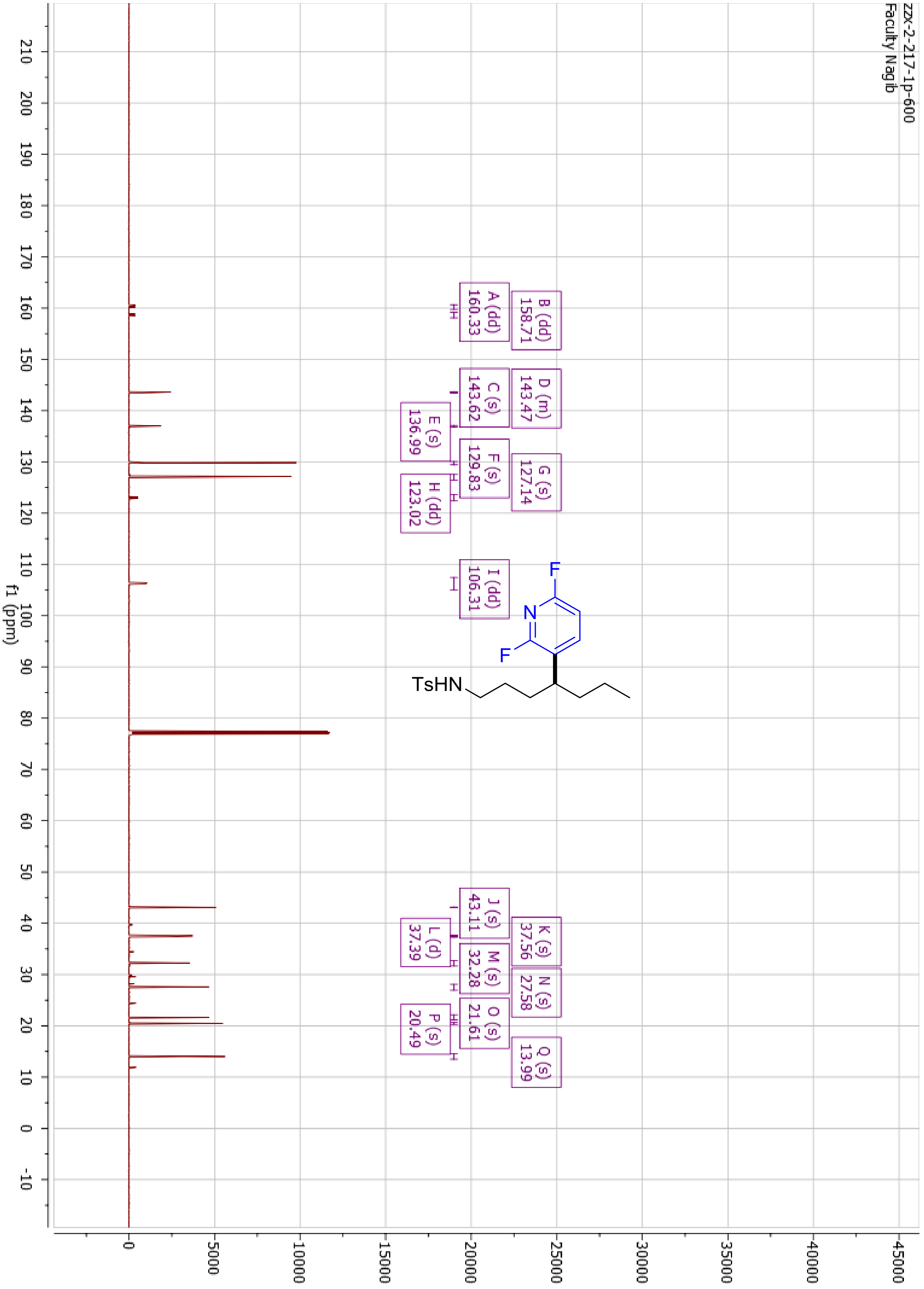
zzx-2-217-1p-600
Faculty Nagib
A (dd) 160.33
B (dd) 158.71
C (s) 143.62
D (m) 143.47
E (s) 136.99
F (s) 129.83
G (s) 127.14
H (dd) 123.02
I (dd) 106.31
J (s) 43.11
K (s) 37.56
L (d) 37.39
M (s) 32.28
N (s) 27.58
O (s) 21.61
P (s) 20.49
Q (s) 13.99
TsHN
N
F
F
f1 (ppm)

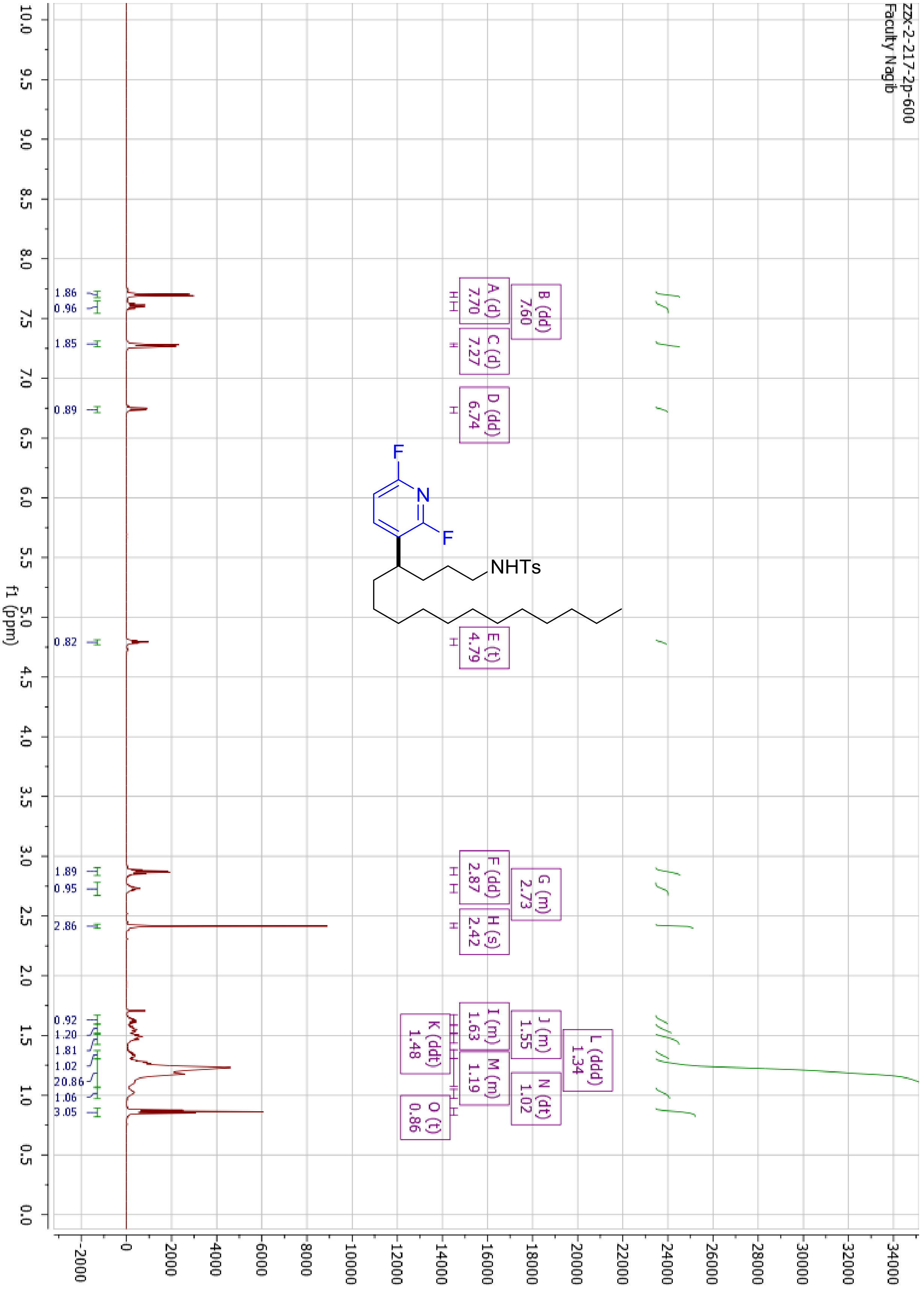
zzx-2-217-2p-600
Faculty Nagib
A (d) 7.70
B (dd) 7.60
C (d) 7.27
D (dd) 6.74
E (t) 4.79
F (dd) 2.87
G (m) 2.73
H (s) 2.42
I (m) 1.63
J (m) 1.55
K (ddt) 1.48
L (ddd) 1.34
M (m) 1.19
N (dt) 1.02
O (t) 0.86
1.86
0.96
1.85
0.89
0.82
1.89
0.95
2.86
0.92
1.20
1.81
1.02
20.86
1.06
3.05
F
N
F
NHTs
f1 (ppm)

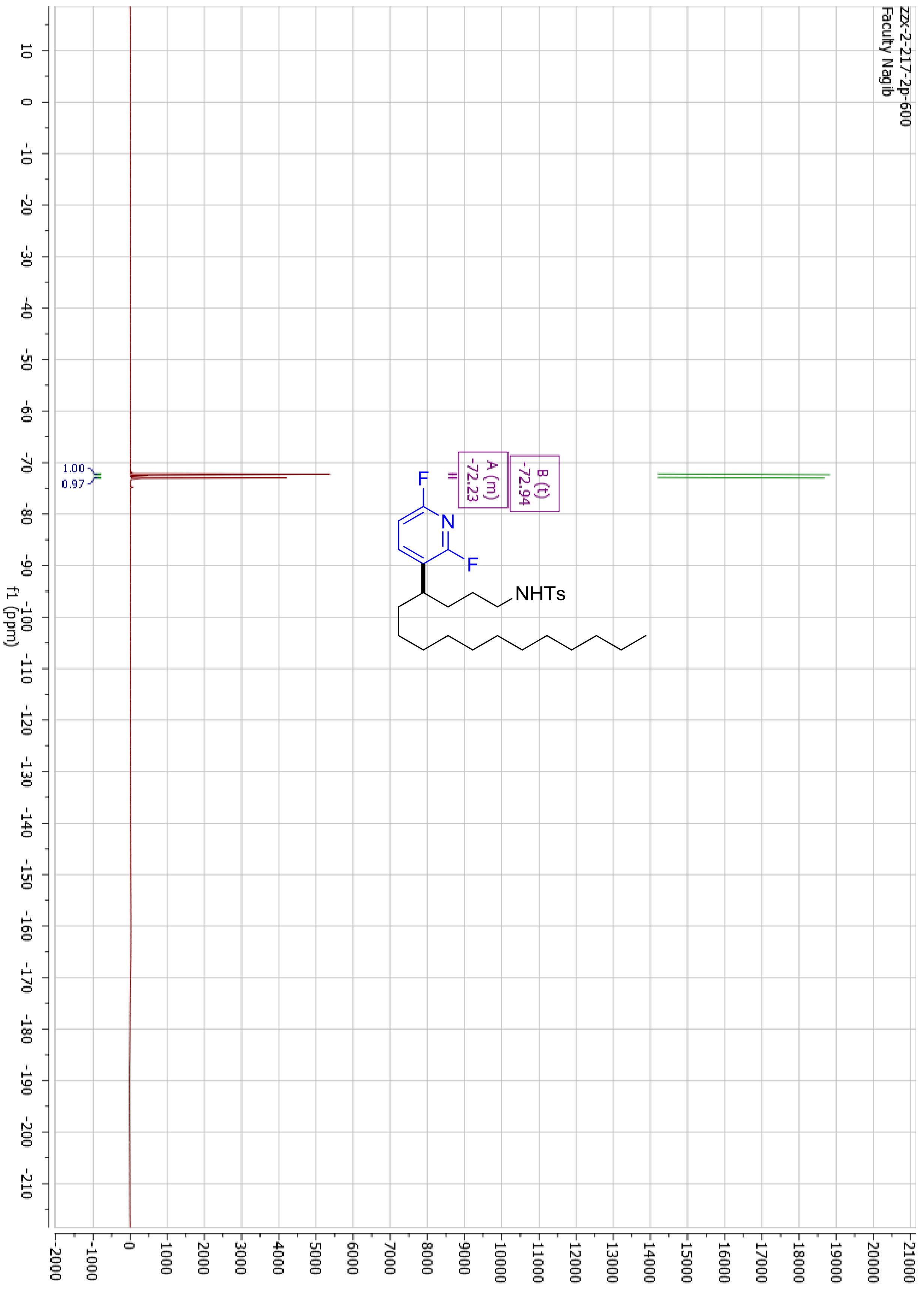

ZZX-2-217-2p-600
Faculty Nagib
A (m)
-72.23
B (t)
-72.94
1.00
0.97
NHTs
F
N
F
f1 (ppm)

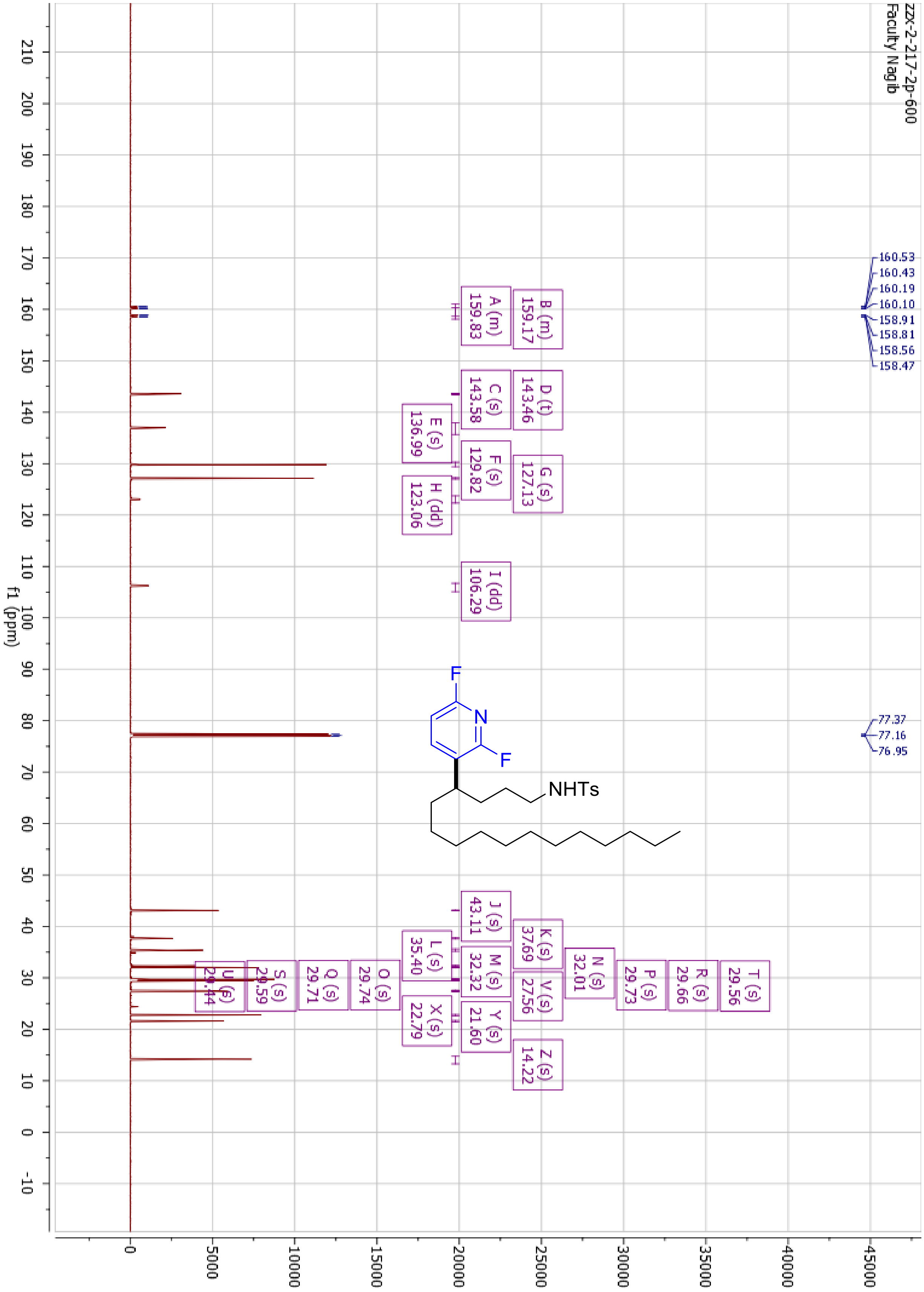

ZZX-2-217-2p-600
Faculty Nagib
160.53
160.43
160.19
160.10
158.91
158.81
158.56
158.47
77.37
77.16
76.95
A (m) 159.83
B (m) 159.17
C (s) 143.58
D (t) 143.46
E (s) 136.99
F (s) 129.82
G (s) 127.13
H (dd) 123.06
I (dd) 106.29
J (s) 43.11
K (s) 37.69
L (s) 35.40
M (s) 32.32
N (s) 32.01
O (s) 29.74
P (s) 29.73
Q (s) 29.71
R (s) 29.66
S (s) 29.59
T (s) 29.56
U (s) 29.44
V (s) 27.56
X (s) 22.79
Y (s) 21.60
Z (s) 14.22
F
N
F
NHTs
f1 (ppm)

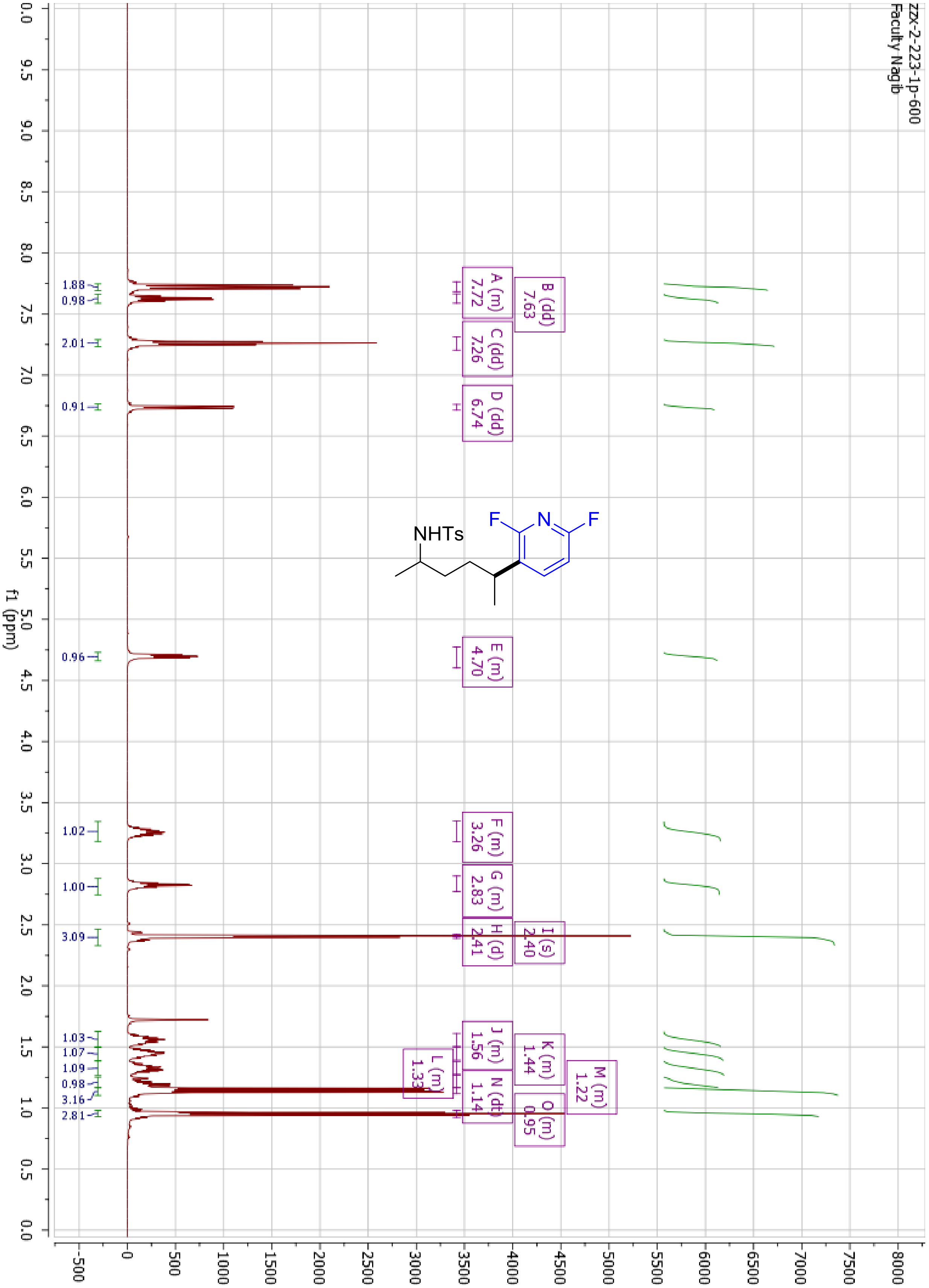
ZZX-2-223-1p-600
Faculty Nagib
NHTs
F
N
F
A (m) 7.72
B (dd) 7.63
C (dd) 7.26
D (dd) 6.74
E (m) 4.70
F (m) 3.26
G (m) 2.83
H (d) 2.41
I (s) 2.40
J (m) 1.56
K (m) 1.44
L (m) 1.33
M (m) 1.22
N (dt) 1.14
O (m) 0.95
1.88
0.98
2.01
0.91
0.96
1.02
1.00
3.09
1.03
1.07
1.09
0.98
3.16
2.81
f1 (ppm)

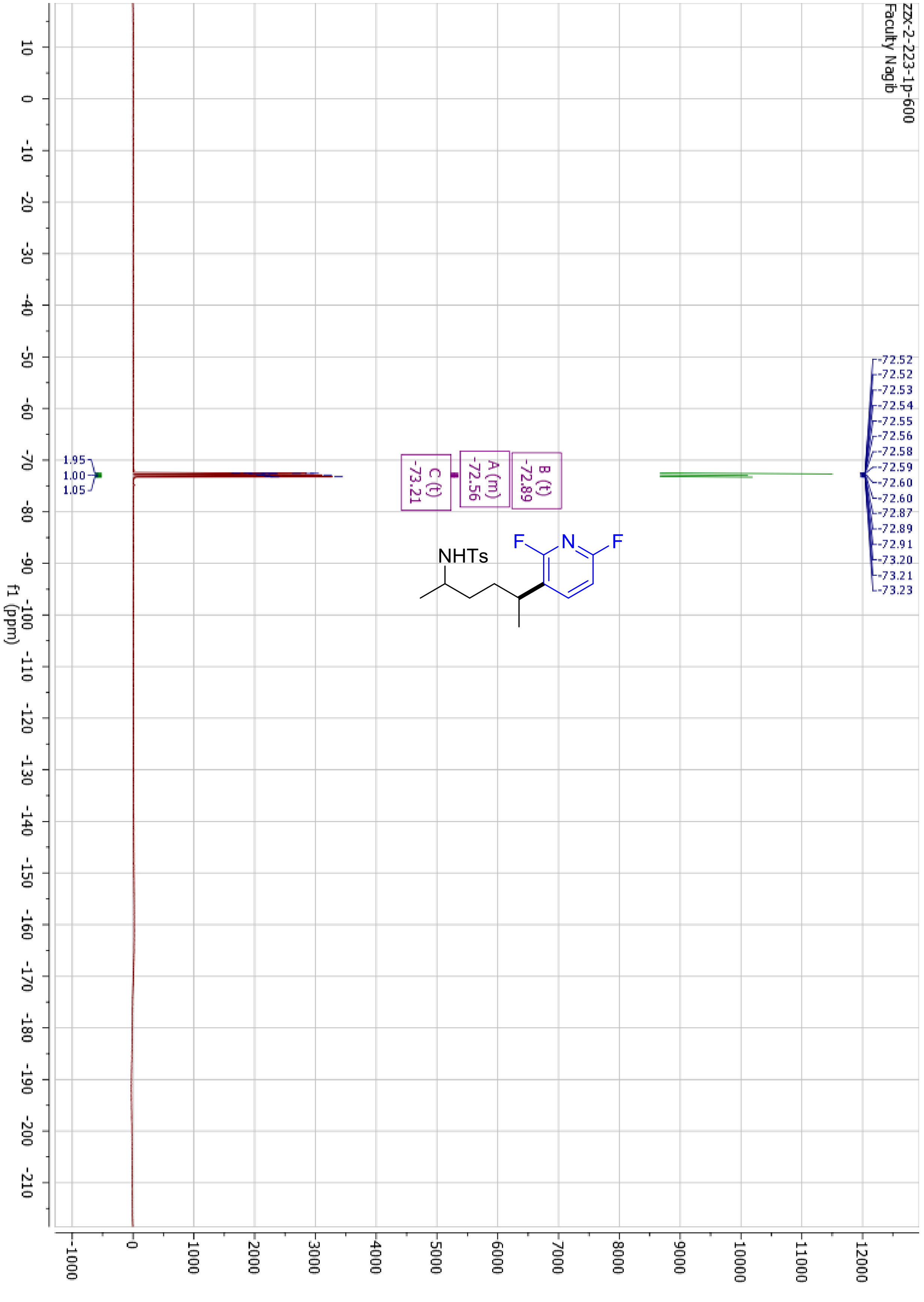
zzx-2-223-1p-600
Faculty Nagib
-72.52
-72.52
-72.53
-72.54
-72.55
-72.56
-72.58
-72.59
-72.60
-72.60
-72.87
-72.89
-72.91
-73.20
-73.21
-73.23
B (t)
-72.89
A (m)
-72.56
C (t)
-73.21
NHTs
F
N
F
1.95
1.00
1.05
f1 (ppm)

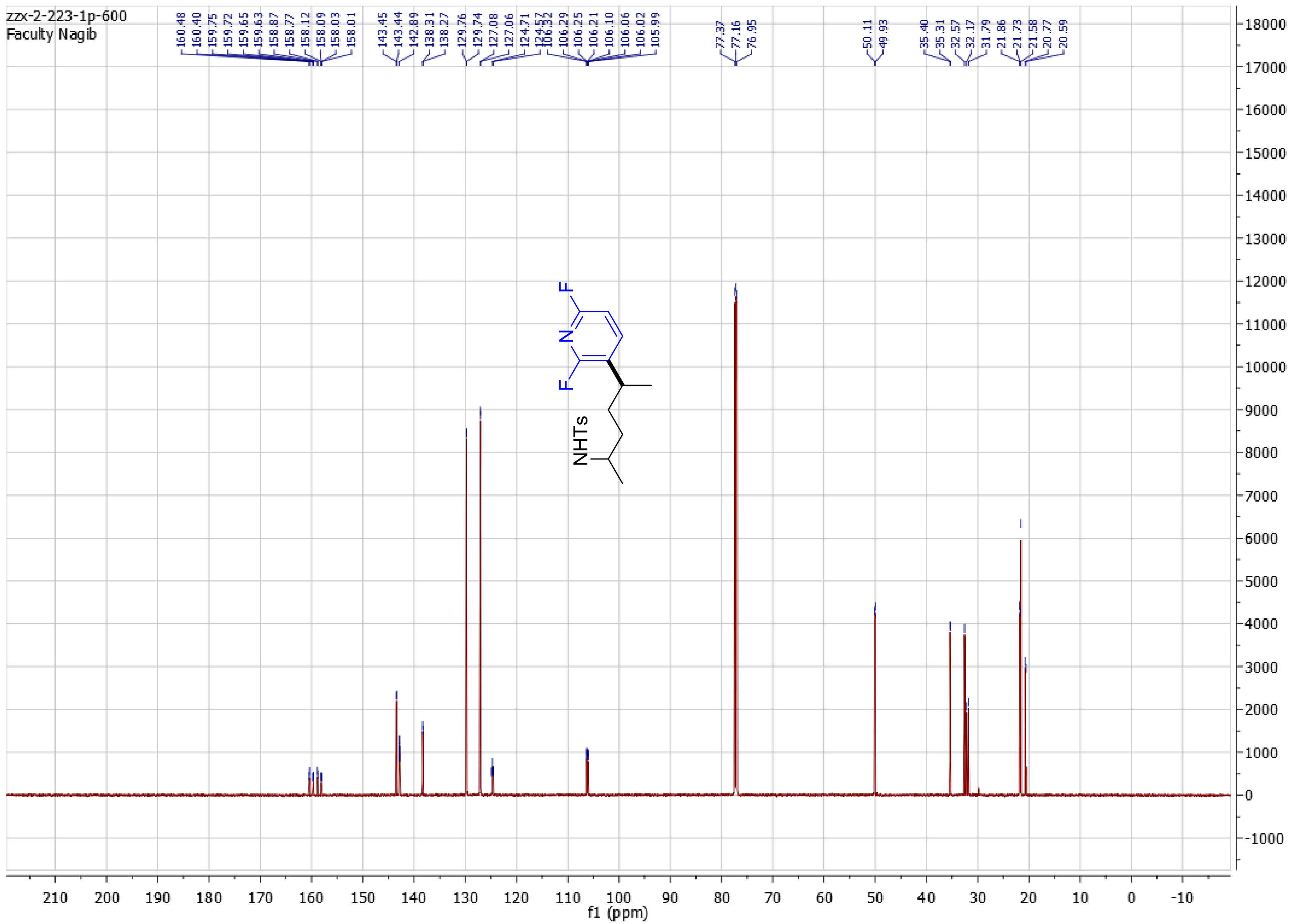

zzx-2-223-1p-600
Faculty Nagib
160.48
160.40
159.75
159.72
159.65
159.63
158.87
158.77
158.12
158.09
158.03
158.01
143.45
143.44
142.89
138.31
138.27
129.76
129.74
127.08
127.06
124.71
124.57
106.32
106.29
106.25
106.21
106.10
106.06
106.02
105.99
77.37
77.16
76.95
50.11
49.93
35.40
35.31
32.57
32.17
31.79
21.86
21.73
21.58
20.77
20.59
NHTs
F
N
F
f1 (ppm)

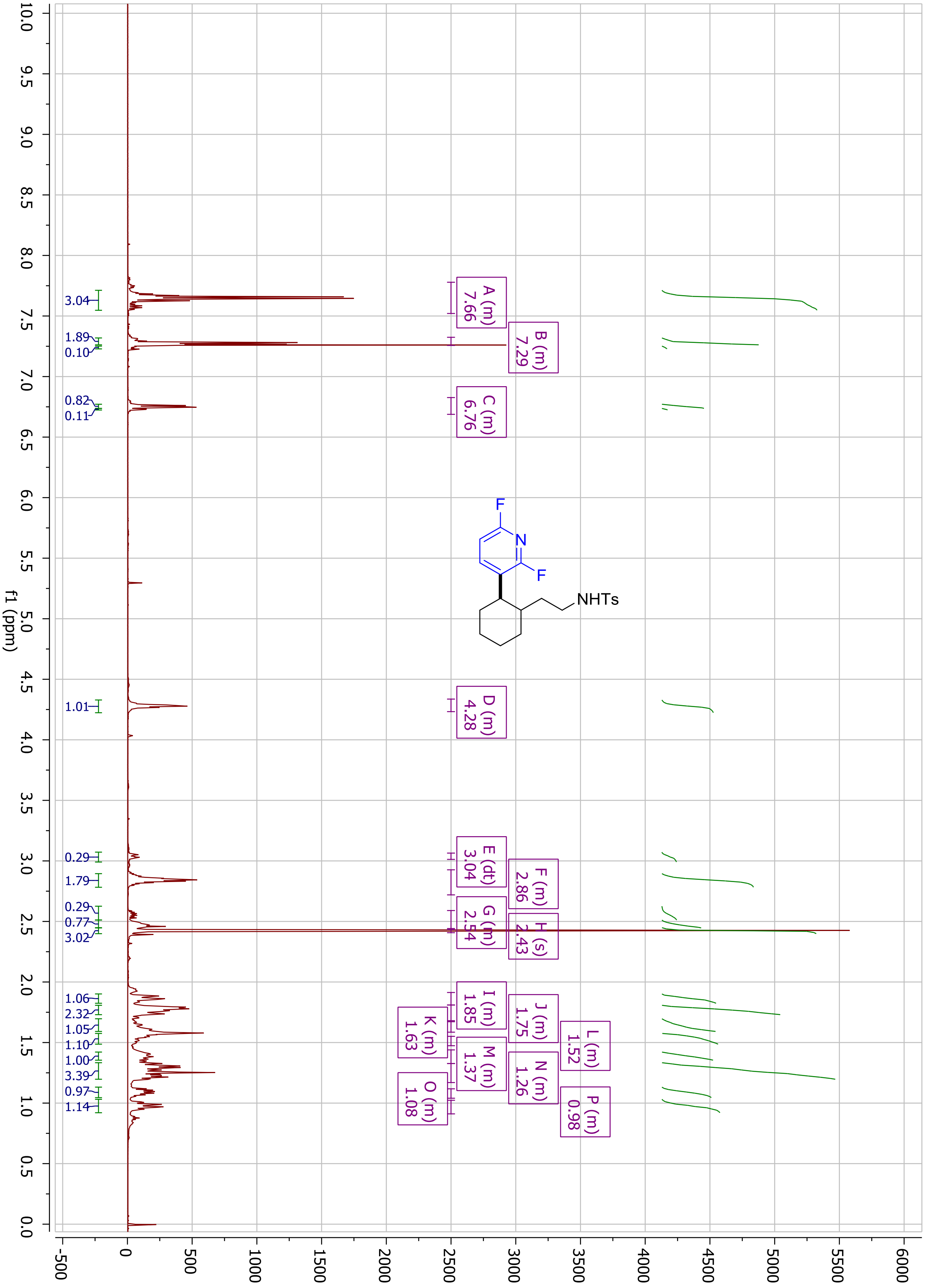
f1 (ppm)
A (m) 7.66
B (m) 7.29
C (m) 6.76
D (m) 4.28
E (dt) 3.04
F (m) 2.86
G (m) 2.54
H (s) 2.43
I (m) 1.85
J (m) 1.75
K (m) 1.63
L (m) 1.52
M (m) 1.37
N (m) 1.26
O (m) 1.08
P (m) 0.98
F
N
F
NHTs

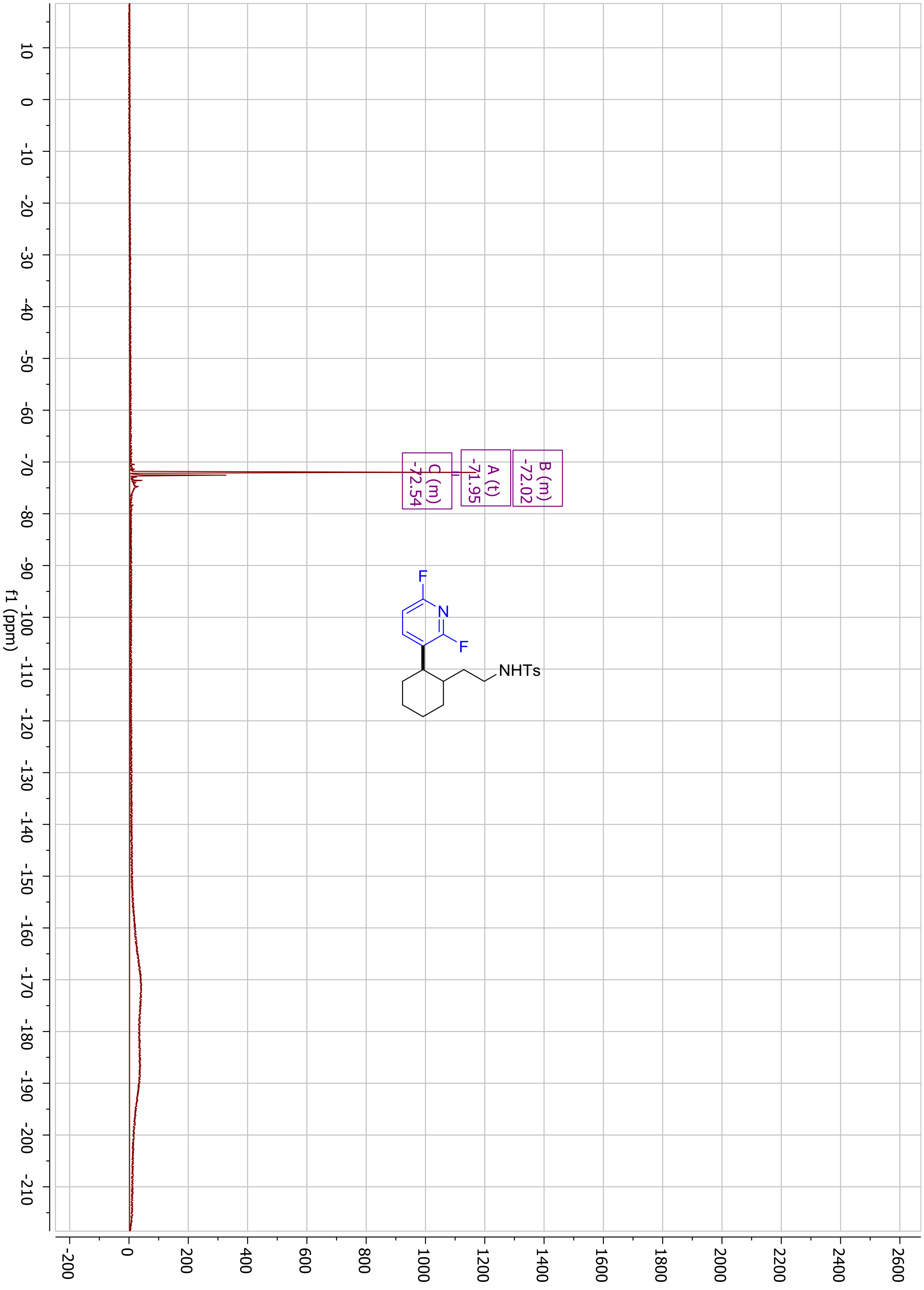
B (m)
-72.02
A (t)
-71.95
C (m)
-72.54
F
N
F
NHTs
f1 (ppm)

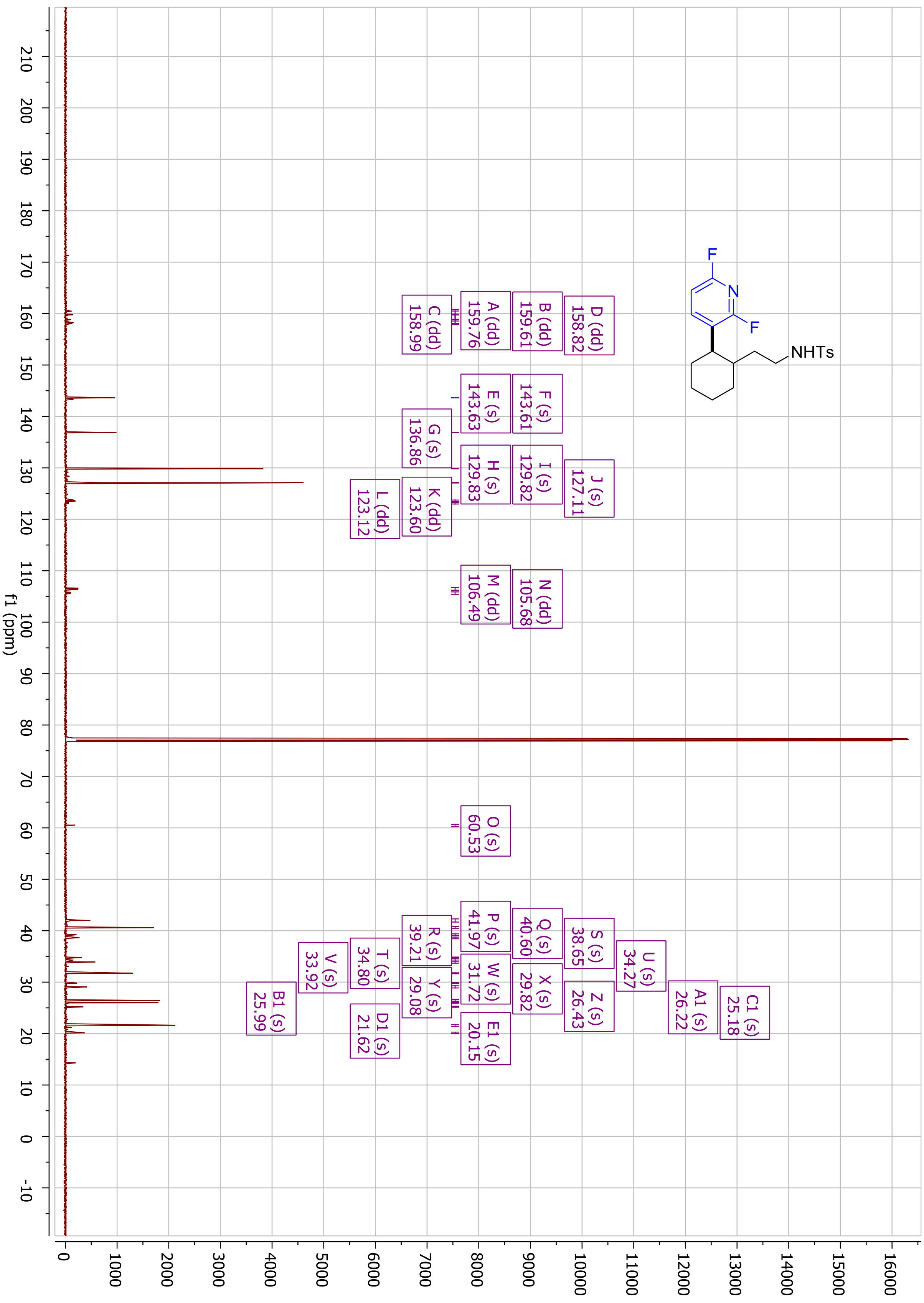

NHTs
F
N
F
A (dd) 159.76
B (dd) 159.61
C (dd) 158.99
D (dd) 158.82
E (s) 143.63
F (s) 143.61
G (s) 136.86
H (s) 129.83
I (s) 129.82
J (s) 127.11
K (dd) 123.60
L (dd) 123.12
M (dd) 106.49
N (dd) 105.68
O (s) 60.53
P (s) 41.97
Q (s) 40.60
R (s) 39.21
S (s) 38.65
T (s) 34.80
U (s) 34.27
V (s) 33.92
W (s) 31.72
X (s) 29.82
Y (s) 29.08
Z (s) 26.43
A1 (s) 26.22
B1 (s) 25.99
C1 (s) 25.18
D1 (s) 21.62
E1 (s) 20.15
f1 (ppm)

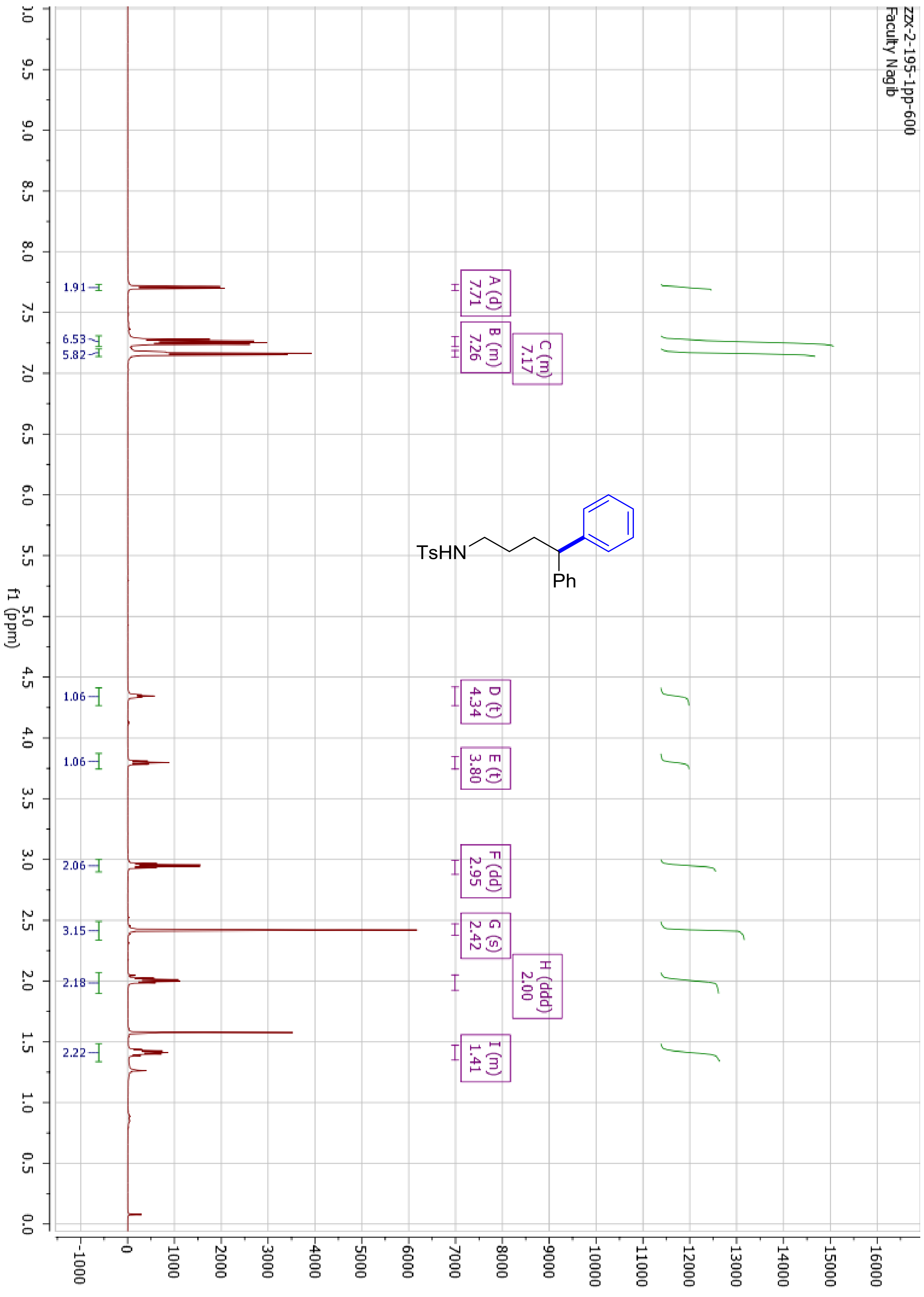

zzx-2-195-1pp-600
Faculty Nagib
TsHN
Ph
A (d) 7.71
B (m) 7.26
C (m) 7.17
D (t) 4.34
E (t) 3.80
F (dd) 2.95
G (s) 2.42
H (ddd) 2.00
I (m) 1.41
1.91
6.53
5.82
1.06
1.06
2.06
3.15
2.18
2.22
f1 (ppm)

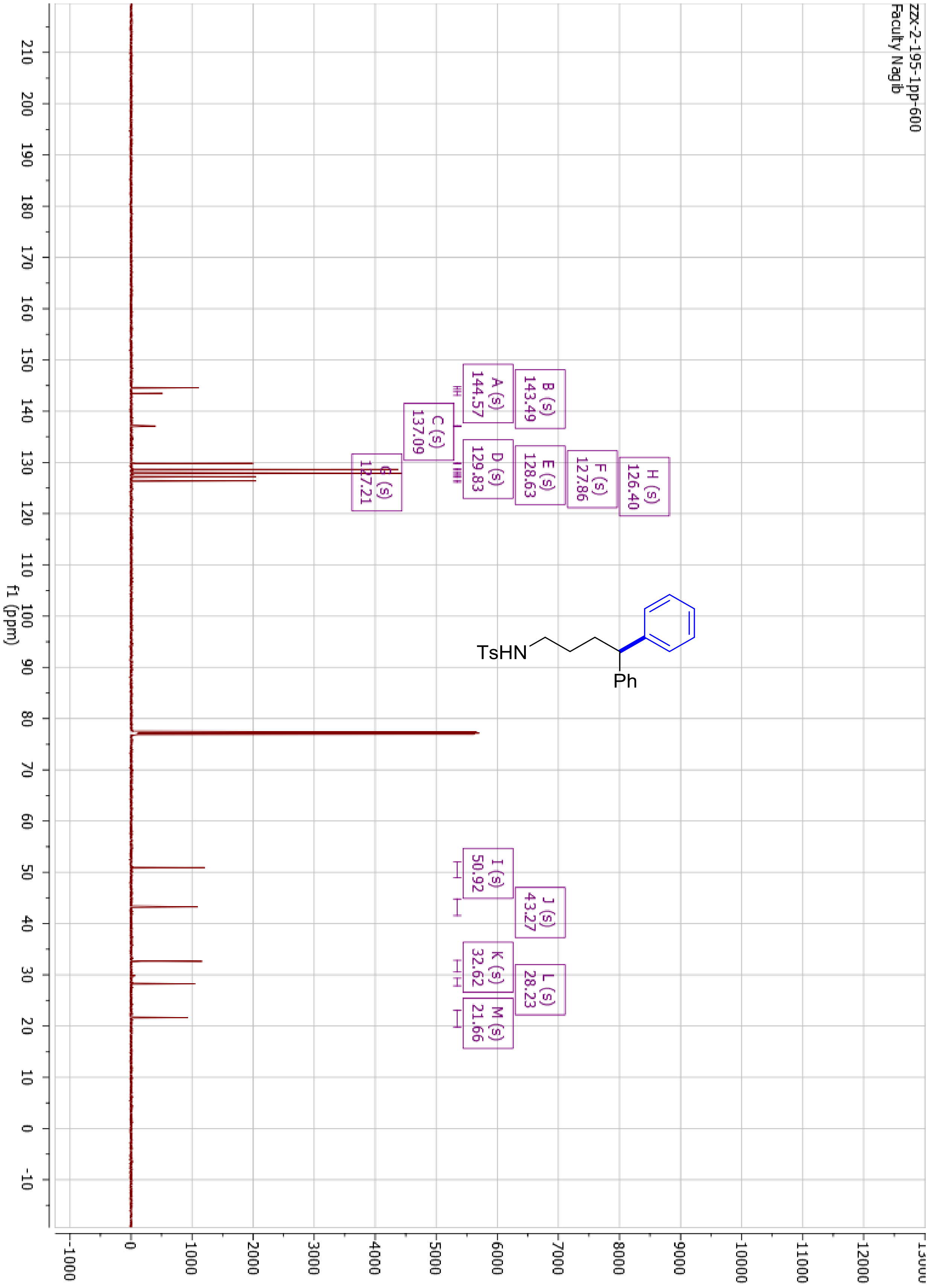
zzx-2-195-1pp-600
Faculty Nagib
A (s) 144.57
B (s) 143.49
C (s) 137.09
D (s) 129.83
E (s) 128.63
F (s) 127.86
G (s) 127.21
H (s) 126.40
I (s) 50.92
J (s) 43.27
K (s) 32.62
L (s) 28.23
M (s) 21.66
TsHN
Ph
f1 (ppm)

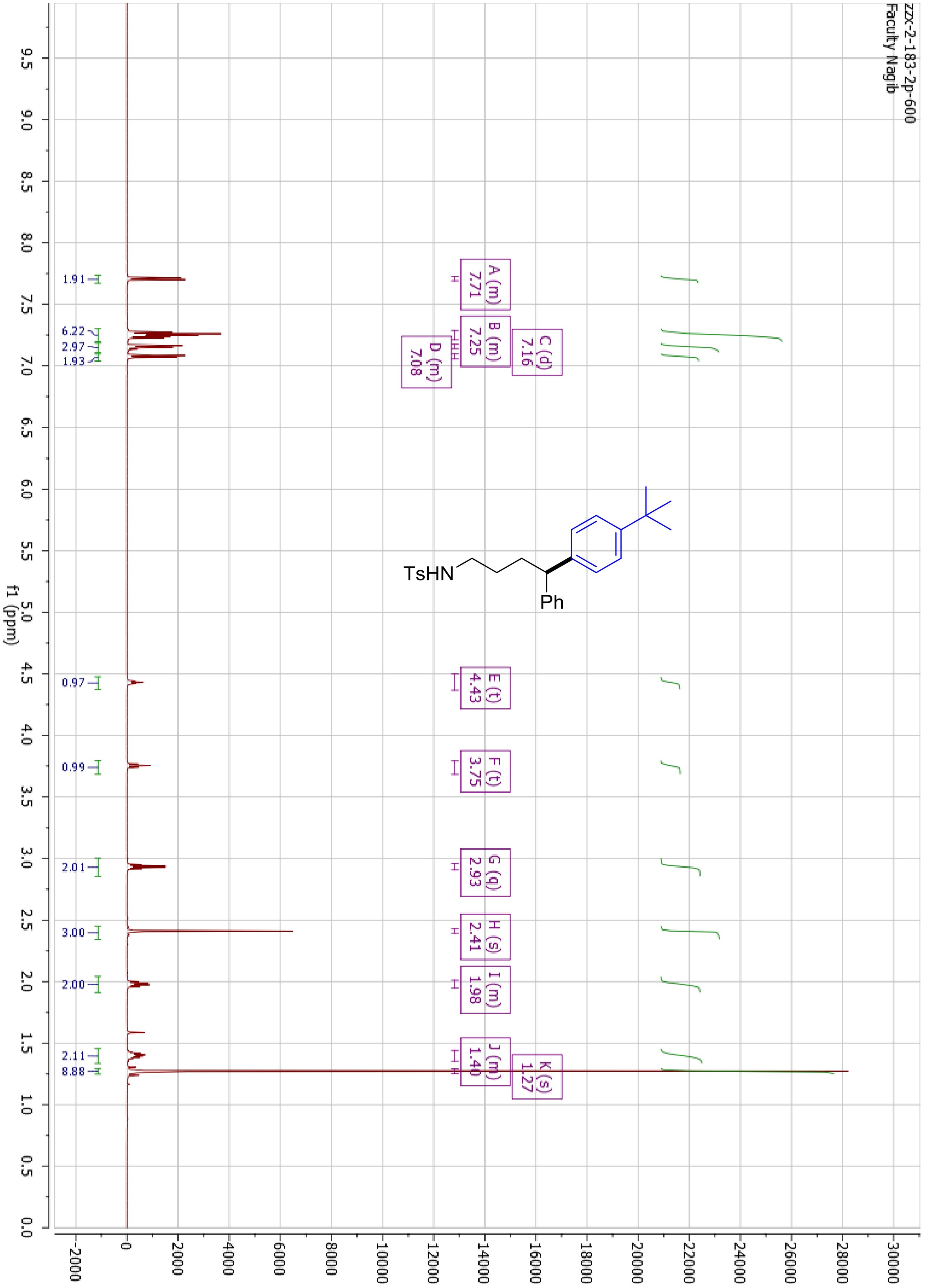
ZZX-2-183-2p-600
Faculty Nagib
TsHN
Ph
A (m) 7.71
B (m) 7.25
C (d) 7.16
D (m) 7.08
E (t) 4.43
F (t) 3.75
G (q) 2.93
H (s) 2.41
I (m) 1.98
J (m) 1.40
K (s) 1.27
1.91
6.22
2.97
1.93
0.97
0.99
2.01
3.00
2.00
2.11
8.88
f1 (ppm)

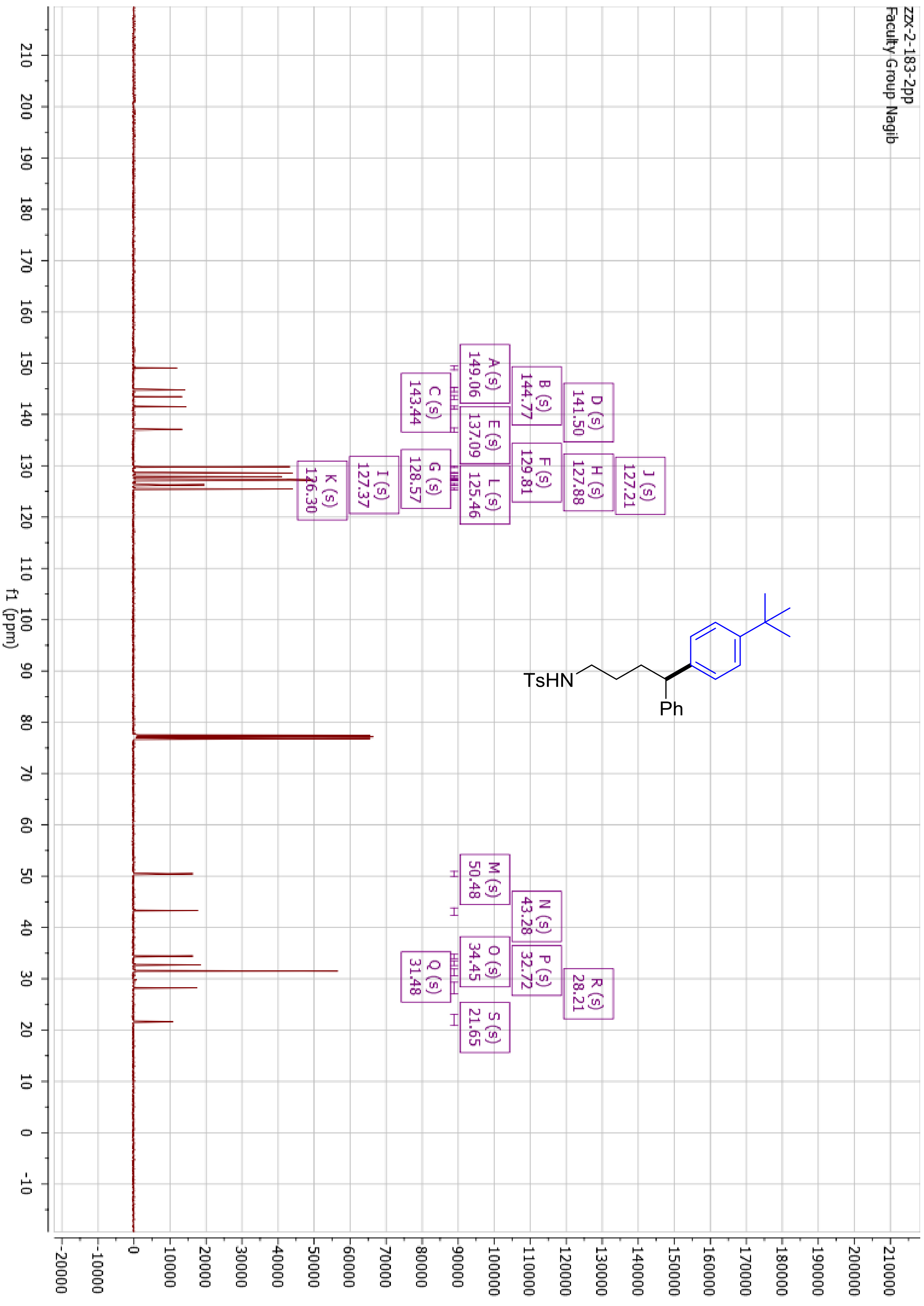
zzx-2-183-2pp
Faculty Group Nagib
TsHN
Ph
A (s) 149.06
B (s) 144.77
C (s) 143.44
D (s) 141.50
E (s) 137.09
F (s) 129.81
G (s) 128.57
H (s) 127.88
I (s) 127.37
J (s) 127.21
K (s) 126.30
L (s) 125.46
M (s) 50.48
N (s) 43.28
O (s) 34.45
P (s) 32.72
Q (s) 31.48
R (s) 28.21
S (s) 21.65
f1 (ppm)

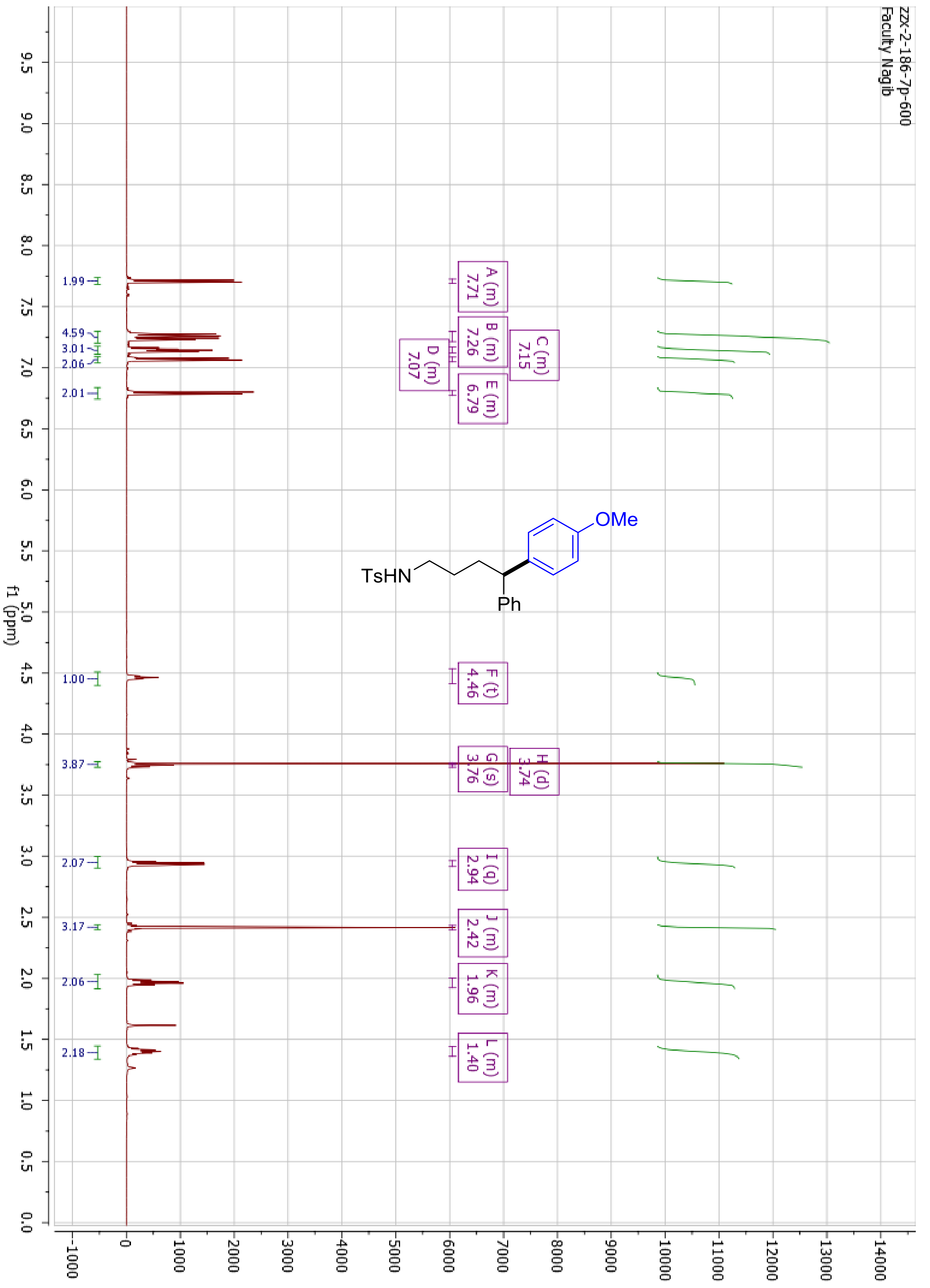
ZZX-2-186-7p-600
Faculty Nagib
TsHN
OMe
Ph
A (m) 7.71
B (m) 7.26
C (m) 7.15
D (m) 7.07
E (m) 6.79
F (t) 4.46
G (s) 3.76
H (d) 3.74
I (q) 2.94
J (m) 2.42
K (m) 1.96
L (m) 1.40
1.99
4.59
3.01
2.06
2.01
1.00
3.87
2.07
3.17
2.06
2.18
f1 (ppm)

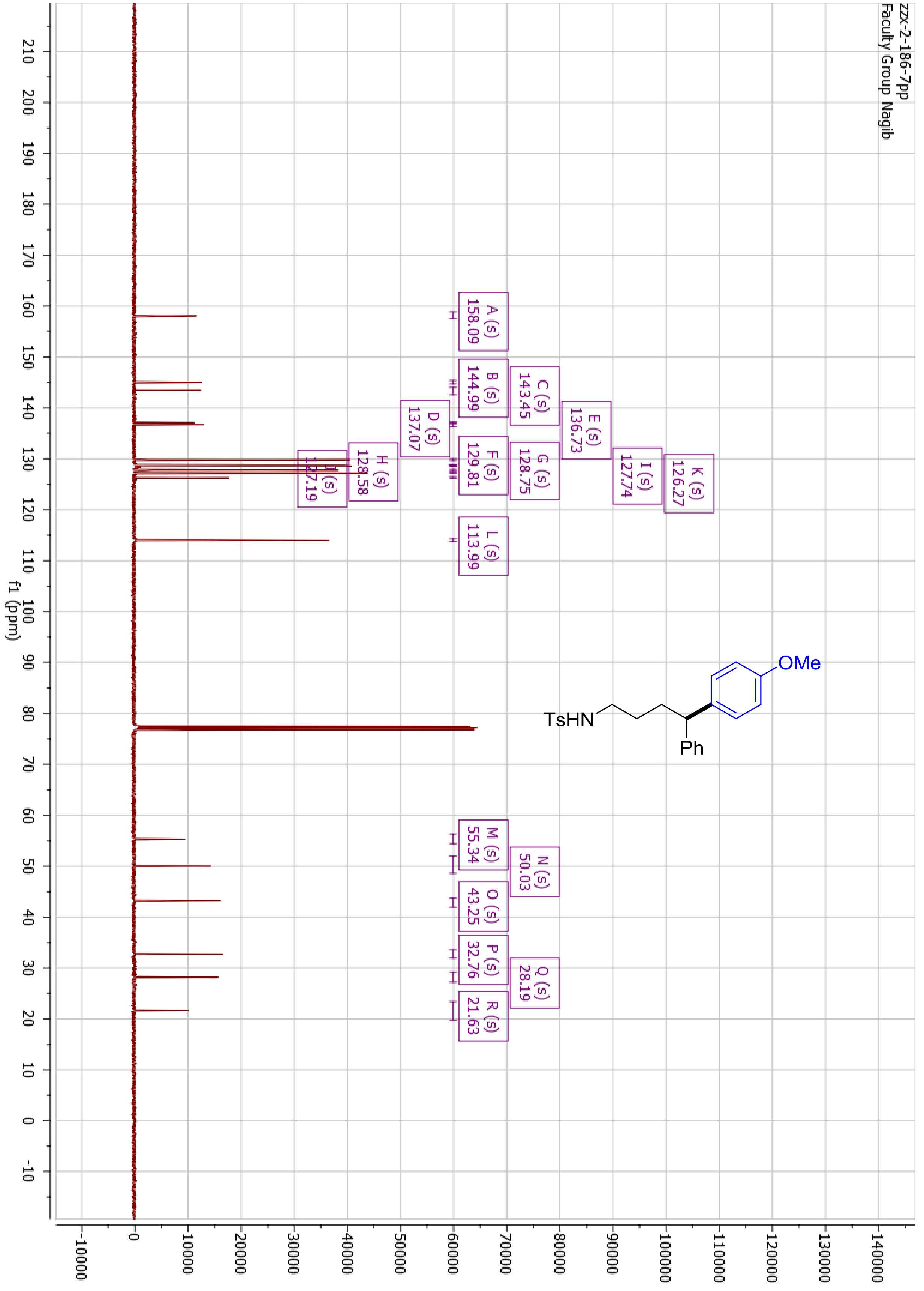

ZZX-2-186-7pp
Faculty Group Nagib
A (s) 158.09
B (s) 144.99
C (s) 143.45
D (s) 137.07
E (s) 136.73
F (s) 129.81
G (s) 128.75
H (s) 128.58
I (s) 127.74
(s) 127.19
K (s) 126.27
L (s) 113.99
M (s) 55.34
N (s) 50.03
O (s) 43.25
P (s) 32.76
Q (s) 28.19
R (s) 21.63
TsHN
Ph
OMe
f1 (ppm)

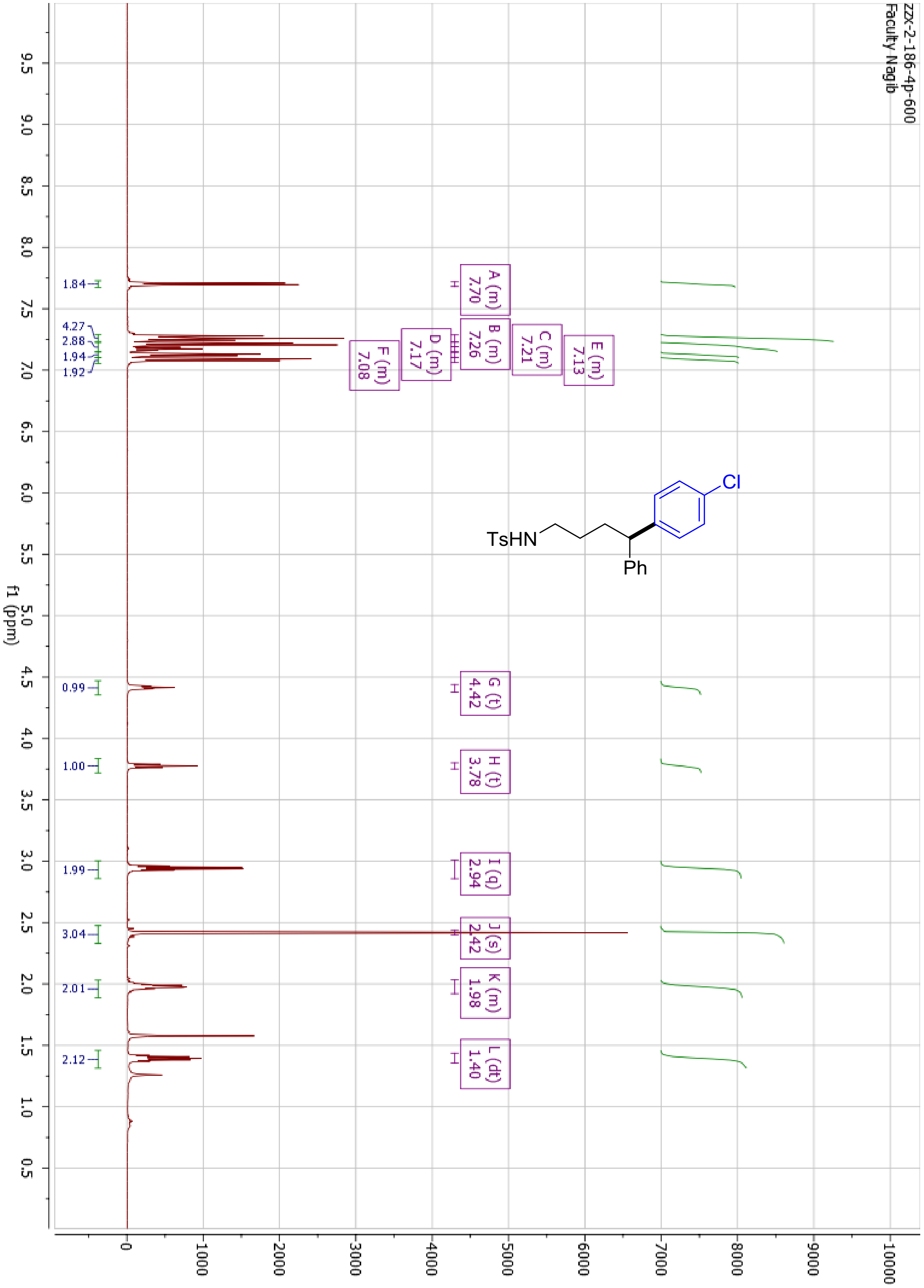
ZZX-2-186-4p-600
Faculty Nagib
A (m) 7.70
B (m) 7.26
C (m) 7.21
D (m) 7.17
E (m) 7.13
F (m) 7.08
G (t) 4.42
H (t) 3.78
I (q) 2.94
J (s) 2.42
K (m) 1.98
L (dt) 1.40
TsHN
Ph
Cl
f1 (ppm)

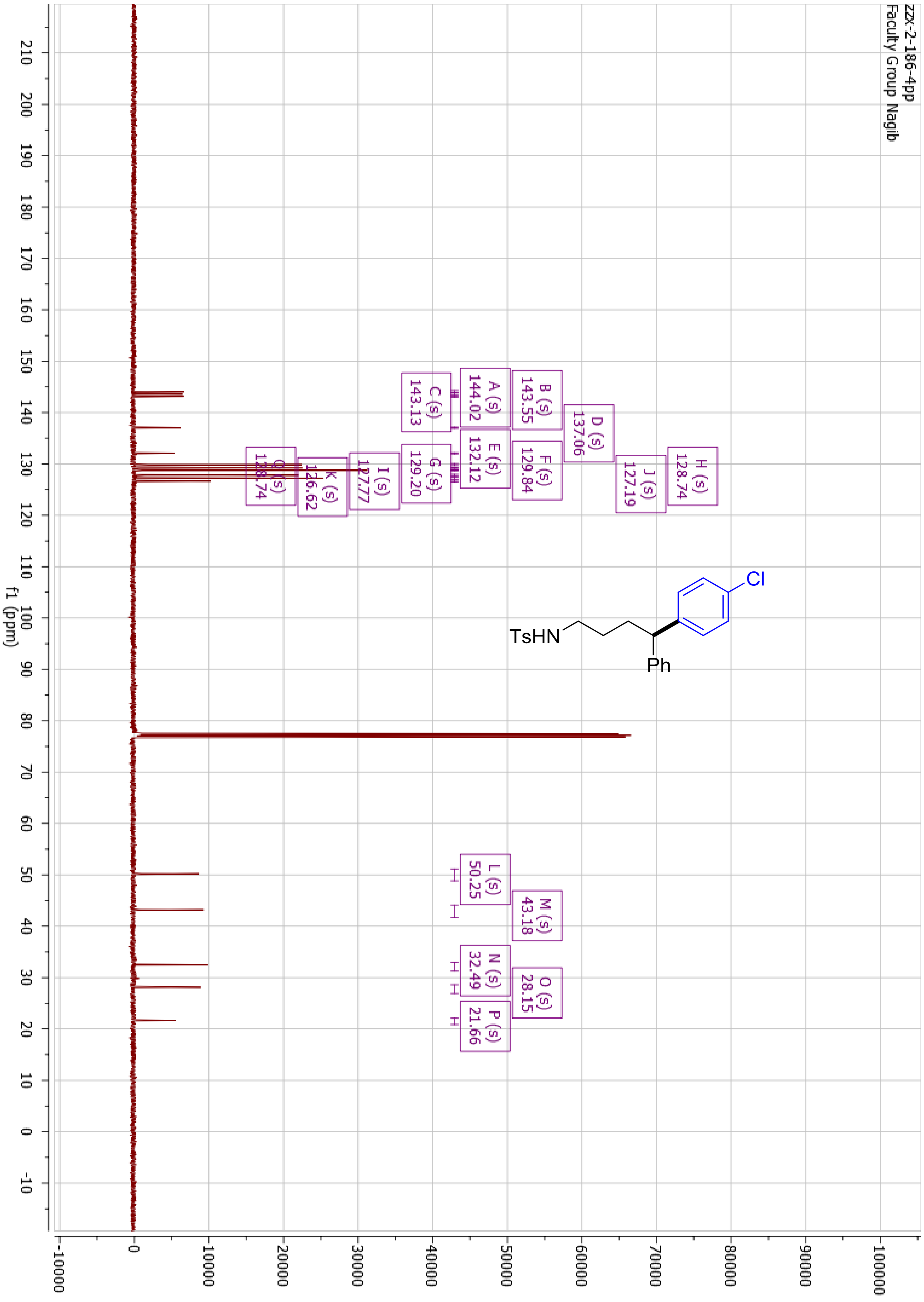
ZZX-2-186-4pp
Faculty Group Nagib
A (s) 144.02
B (s) 143.55
C (s) 143.13
D (s) 137.06
E (s) 132.12
F (s) 129.84
G (s) 129.20
H (s) 128.74
I (s) 127.77
J (s) 127.19
K (s) 126.62
Q (s) 128.74
L (s) 50.25
M (s) 43.18
N (s) 32.49
O (s) 28.15
P (s) 21.66
TsHN
Ph
Cl
f1 (ppm)

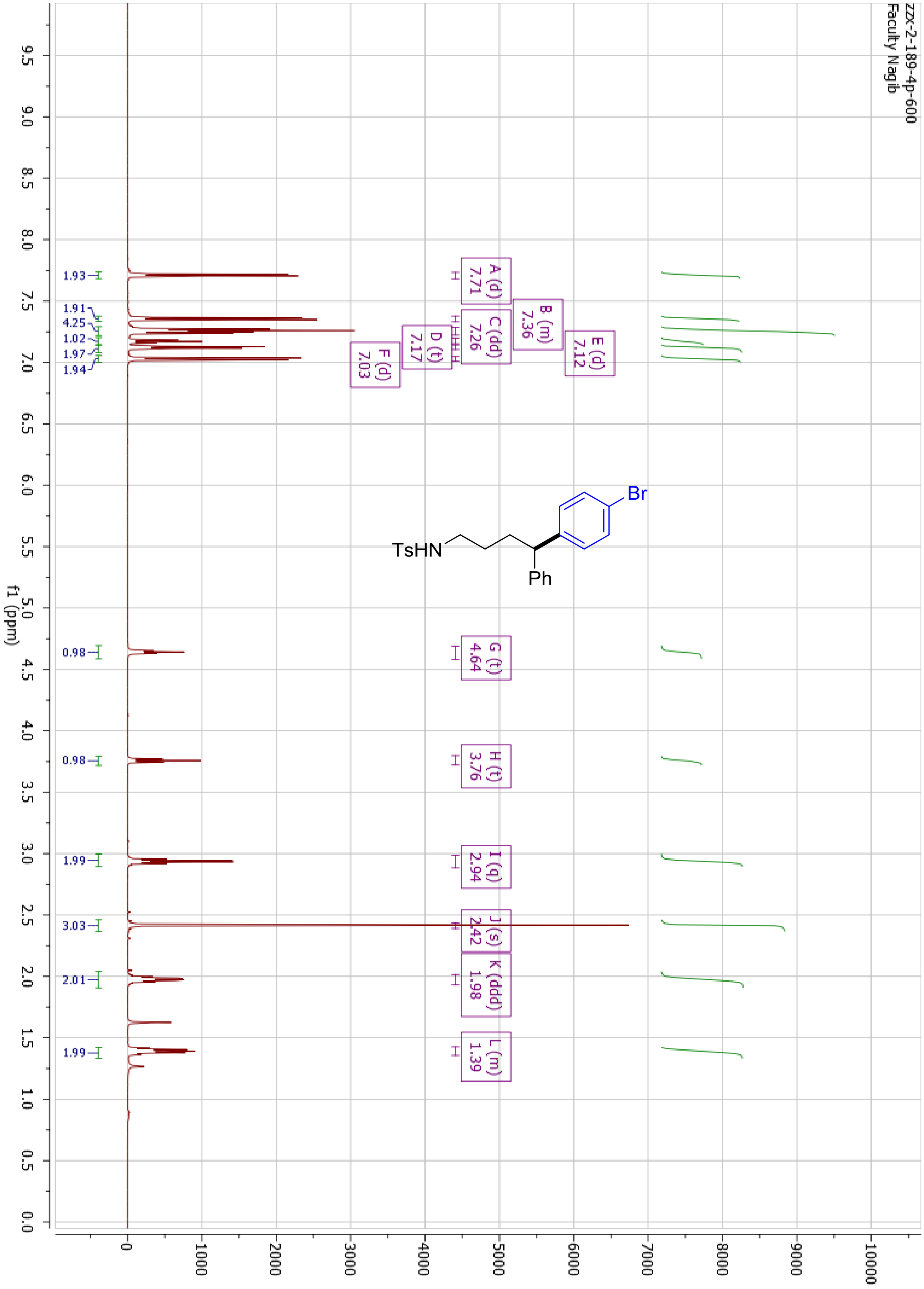
zzx-2-189-4p-600
Faculty Nagib
A (d) 7.71
B (m) 7.36
C (dd) 7.26
D (t) 7.17
E (d) 7.12
F (d) 7.03
G (t) 4.64
H (t) 3.76
I (q) 2.94
J (s) 2.42
K (ddd) 1.98
L (m) 1.39
1.93
1.91
4.25
1.02
1.97
1.94
0.98
0.98
1.99
3.03
2.01
1.99
TsHN
Br
Ph
f1 (ppm)

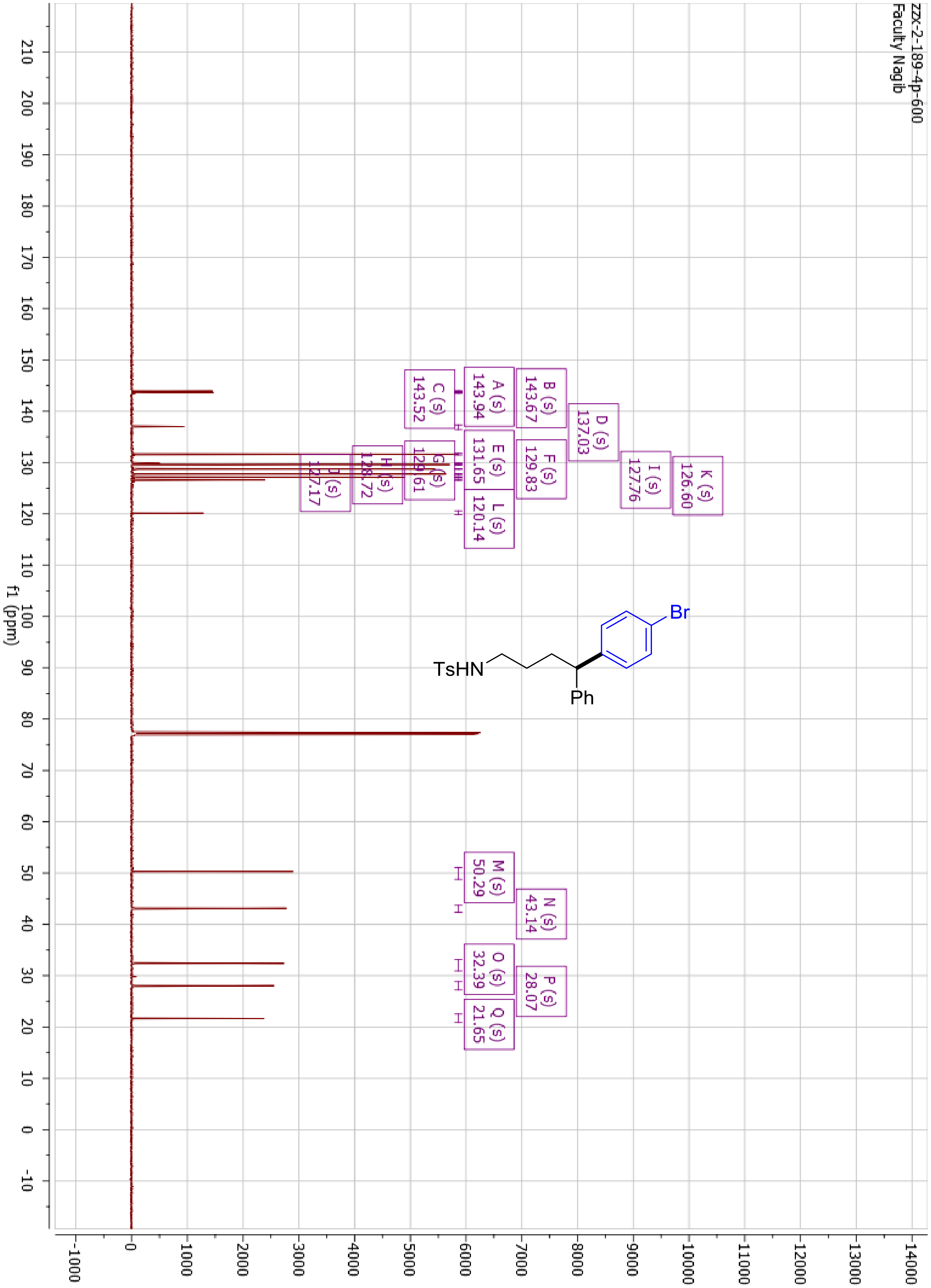

zzx-2-189-4p-600
Faculty Nagib
A (s) 143.94
B (s) 143.67
C (s) 143.52
D (s) 137.03
E (s) 131.65
F (s) 129.83
G (s) 129.61
H (s) 128.72
I (s) 127.76
J (s) 127.17
K (s) 126.60
L (s) 120.14
M (s) 50.29
N (s) 43.14
O (s) 32.39
P (s) 28.07
Q (s) 21.65
Br
TsHN
Ph
f1 (ppm)

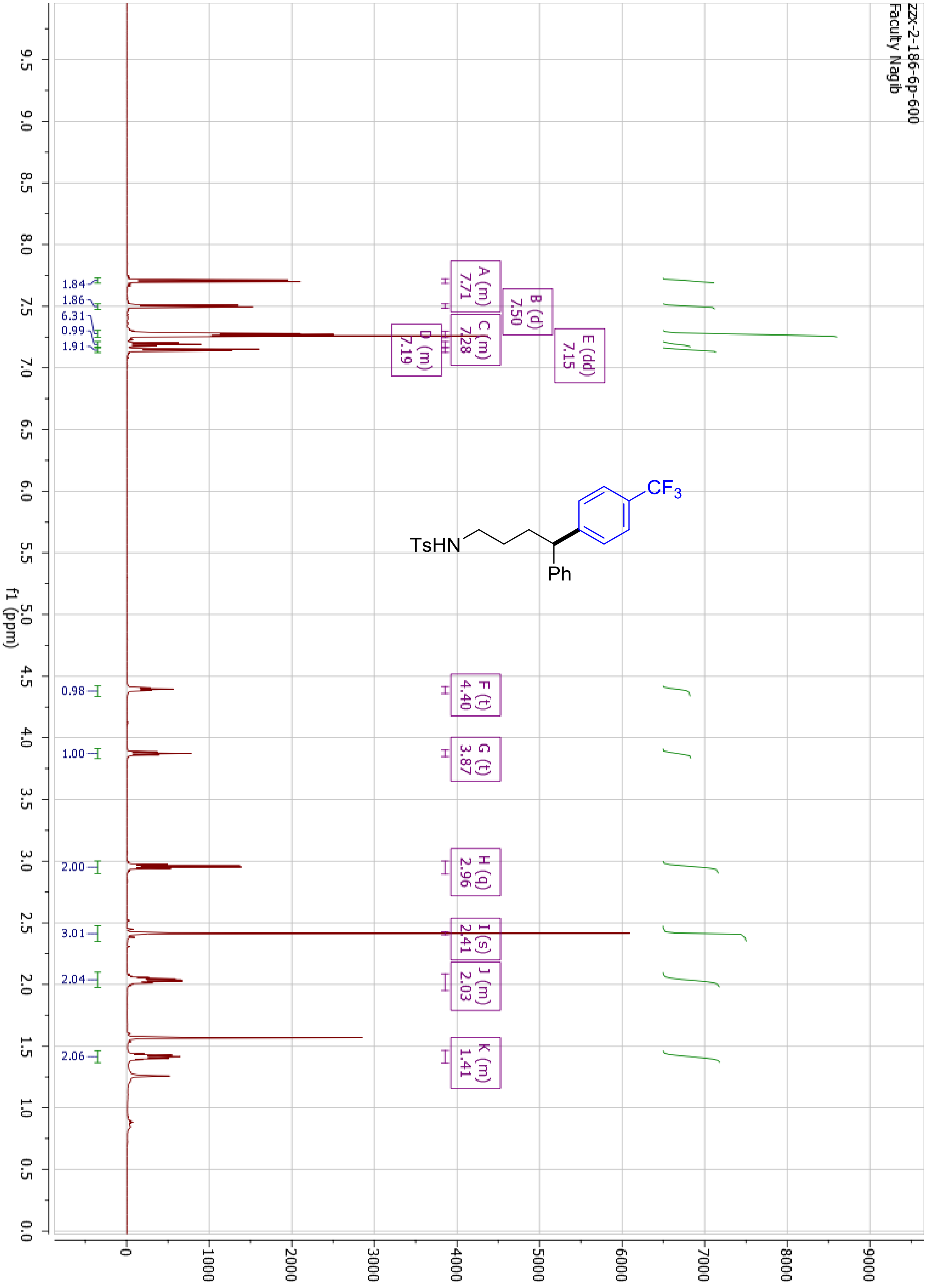
ZZX-2-186-6p-600
Faculty Nagib
TsHN
Ph
CF3
A (m) 7.71
B (d) 7.50
C (m) 7.28
D (m) 7.19
E (dd) 7.15
F (t) 4.40
G (t) 3.87
H (q) 2.96
I (s) 2.41
J (m) 2.03
K (m) 1.41
f1 (ppm)

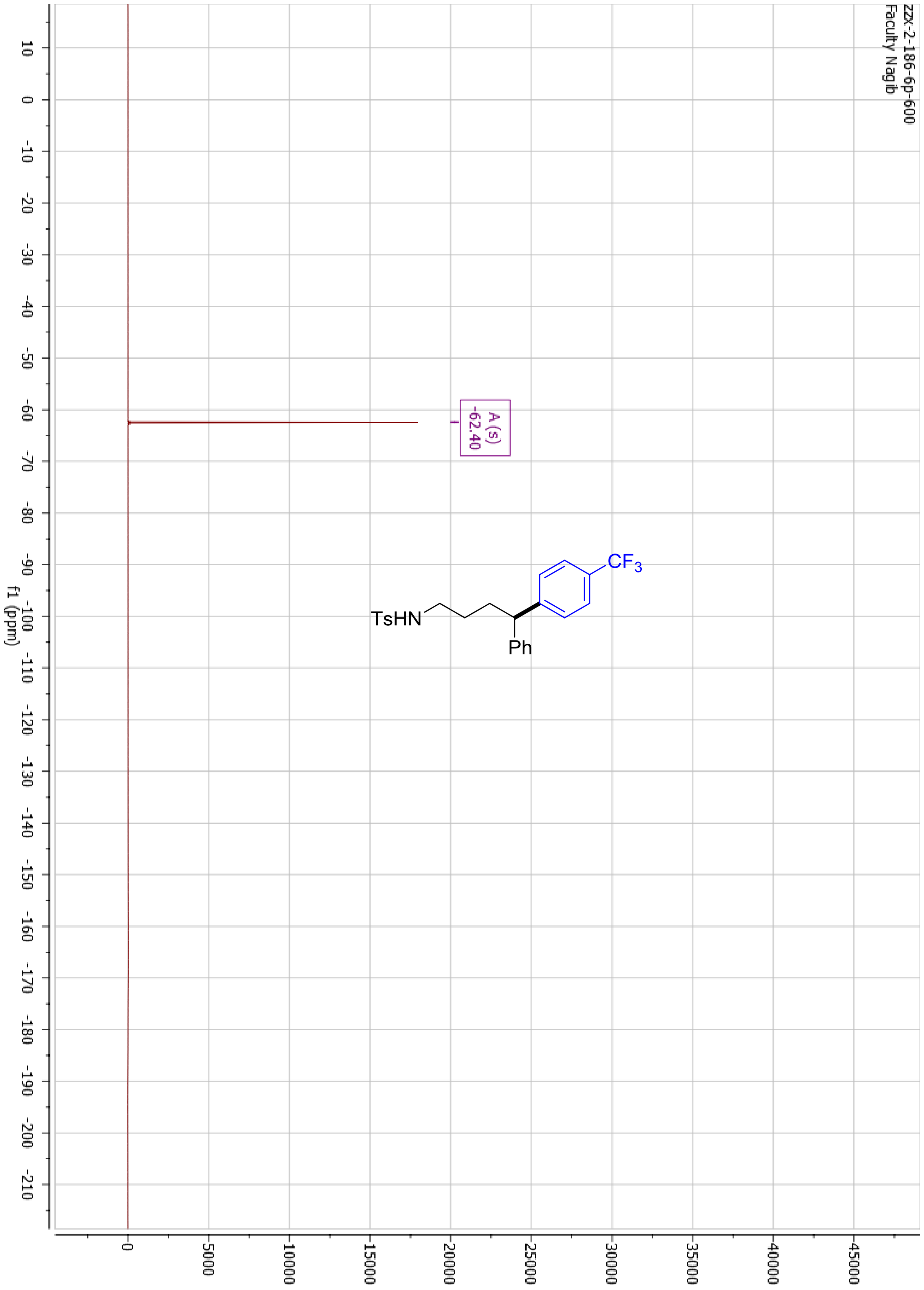
ZZX-2-186-6p-600
Faculty Nagib
A (s)
-62.40
TsHN
Ph
CF3
f1 (ppm)

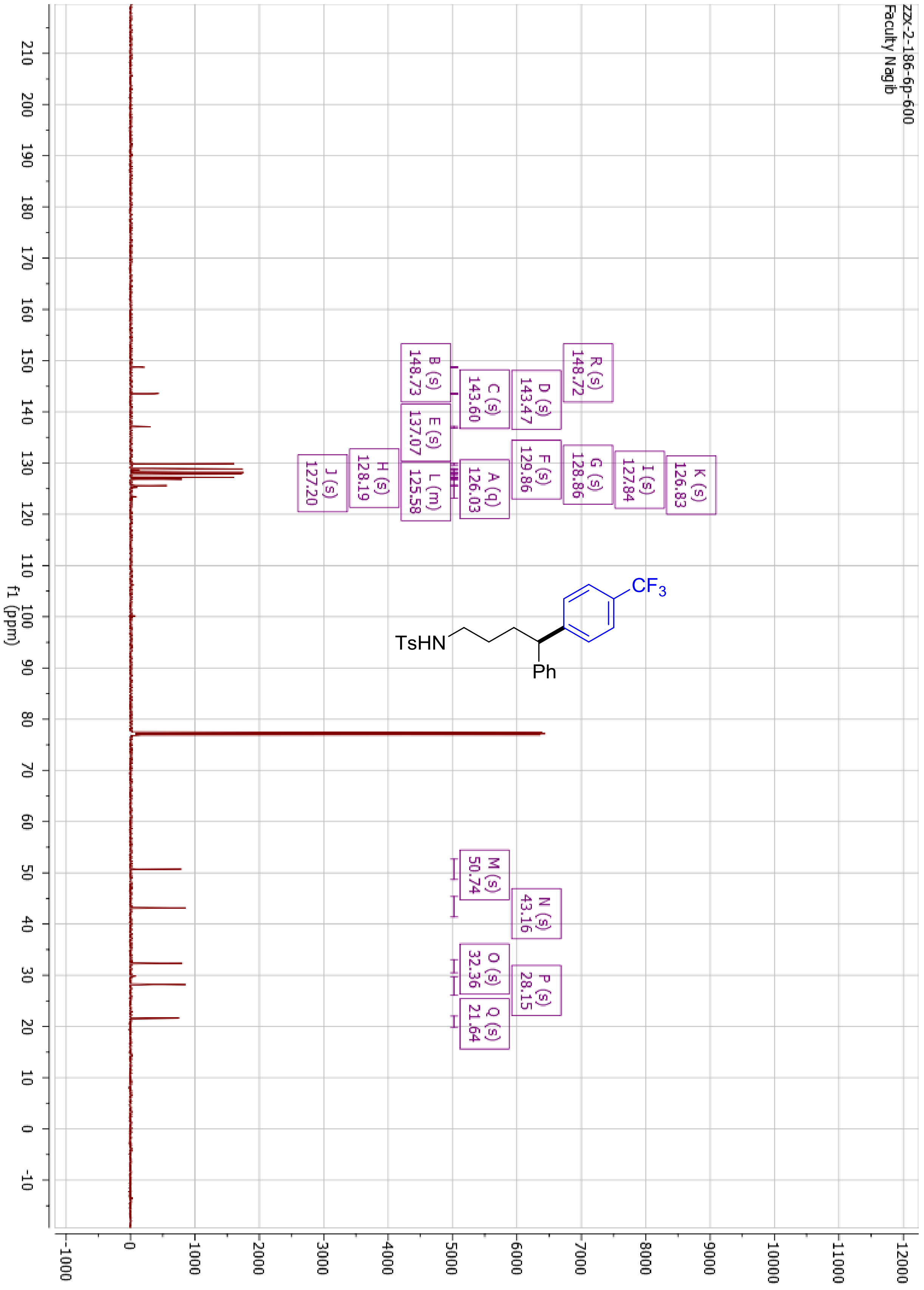
zzx-2-186-6p-600
Faculty Nagib
R (s) 148.72
B (s) 148.73
C (s) 143.60
D (s) 143.47
E (s) 137.07
F (s) 129.86
G (s) 128.86
I (s) 127.84
K (s) 126.83
A (q) 126.03
L (m) 125.58
H (s) 128.19
J (s) 127.20
M (s) 50.74
N (s) 43.16
O (s) 32.36
P (s) 28.15
Q (s) 21.64
CF_3
TsHN
Ph
f1 (ppm)

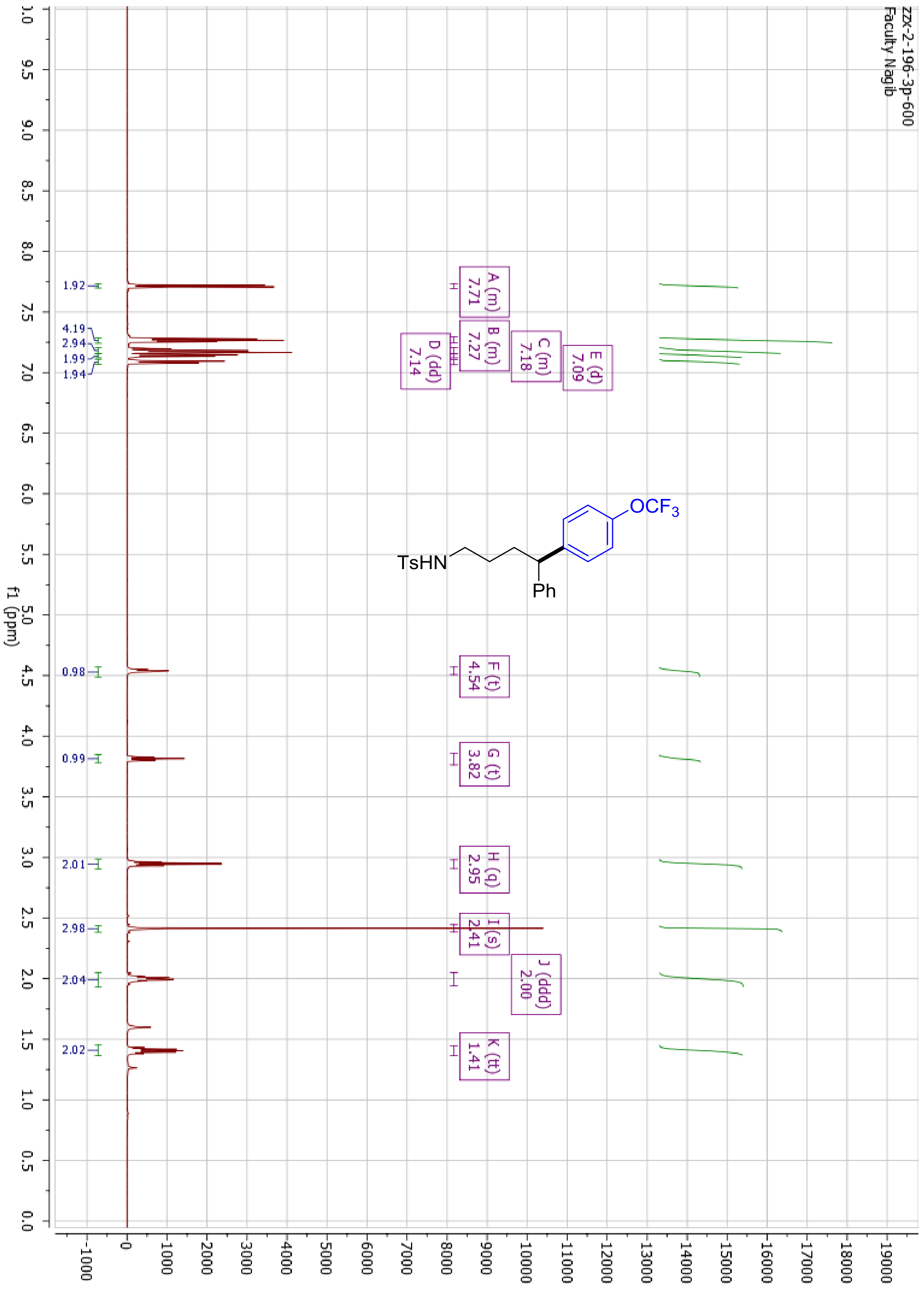
zzx-2-196-3p-600
Faculty Nagib
TsHN
Ph
OCF3
A (m) 7.71
B (m) 7.27
C (m) 7.18
D (dd) 7.14
E (d) 7.09
F (t) 4.54
G (t) 3.82
H (q) 2.95
I (s) 2.41
J (ddd) 2.00
K (tt) 1.41
1.92
4.19
2.94
1.99
1.94
0.98
0.99
2.01
2.98
2.04
2.02
f1 (ppm)

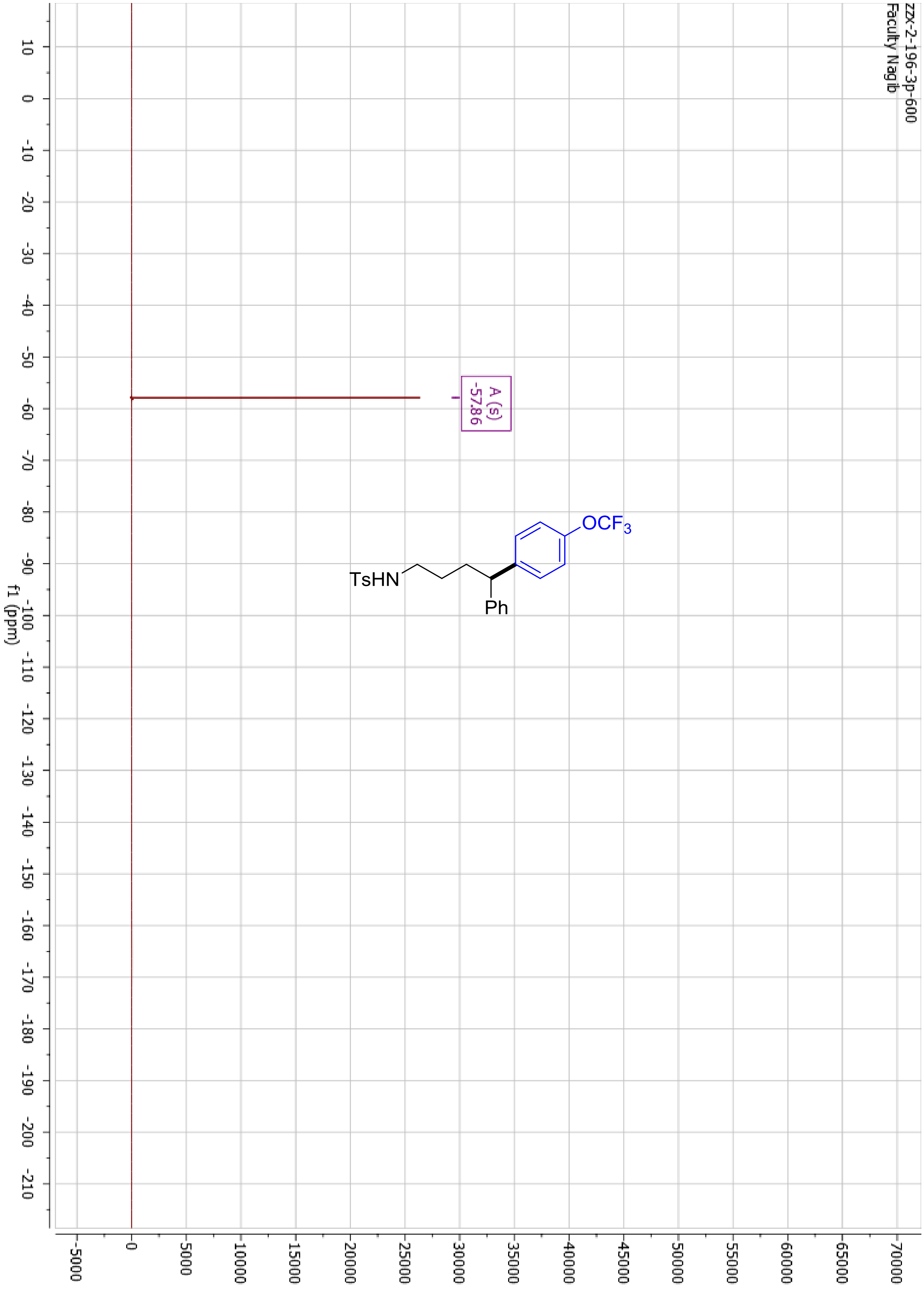

ZZX-2-196-3p-600
Faculty Nagib
A (s)
-57.86
TsHN
Ph
OCF3
f1 (ppm)

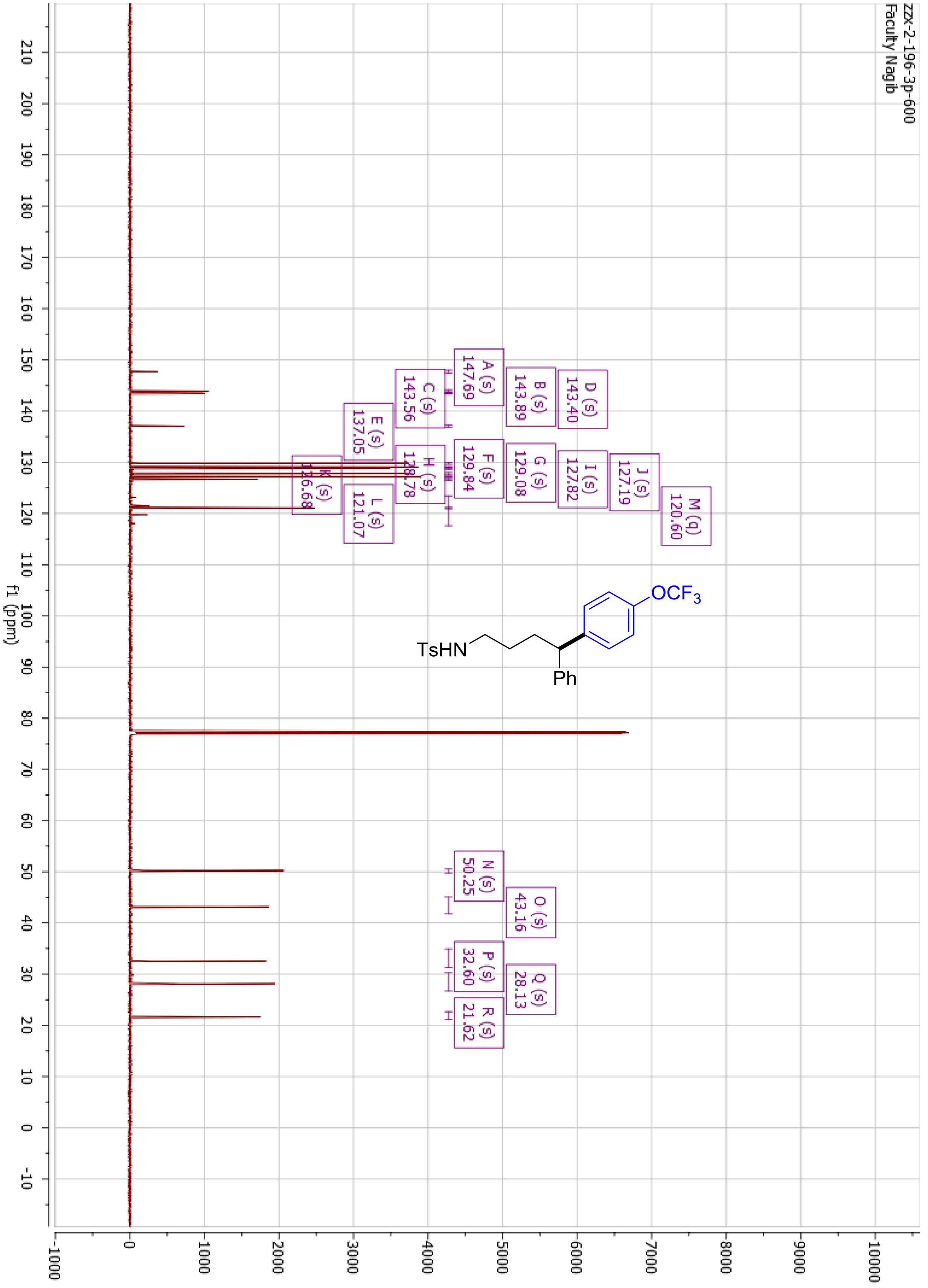
ZZX-2-196-3p-600
Faculty Nagib
A (s) 147.69
B (s) 143.89
C (s) 143.56
D (s) 143.40
E (s) 137.05
F (s) 129.84
G (s) 129.08
H (s) 128.78
I (s) 127.82
J (s) 127.19
K (s) 126.68
L (s) 121.07
M (q) 120.60
N (s) 50.25
O (s) 43.16
P (s) 32.60
Q (s) 28.13
R (s) 21.62
OCF3
TsHN
Ph
f1 (ppm)

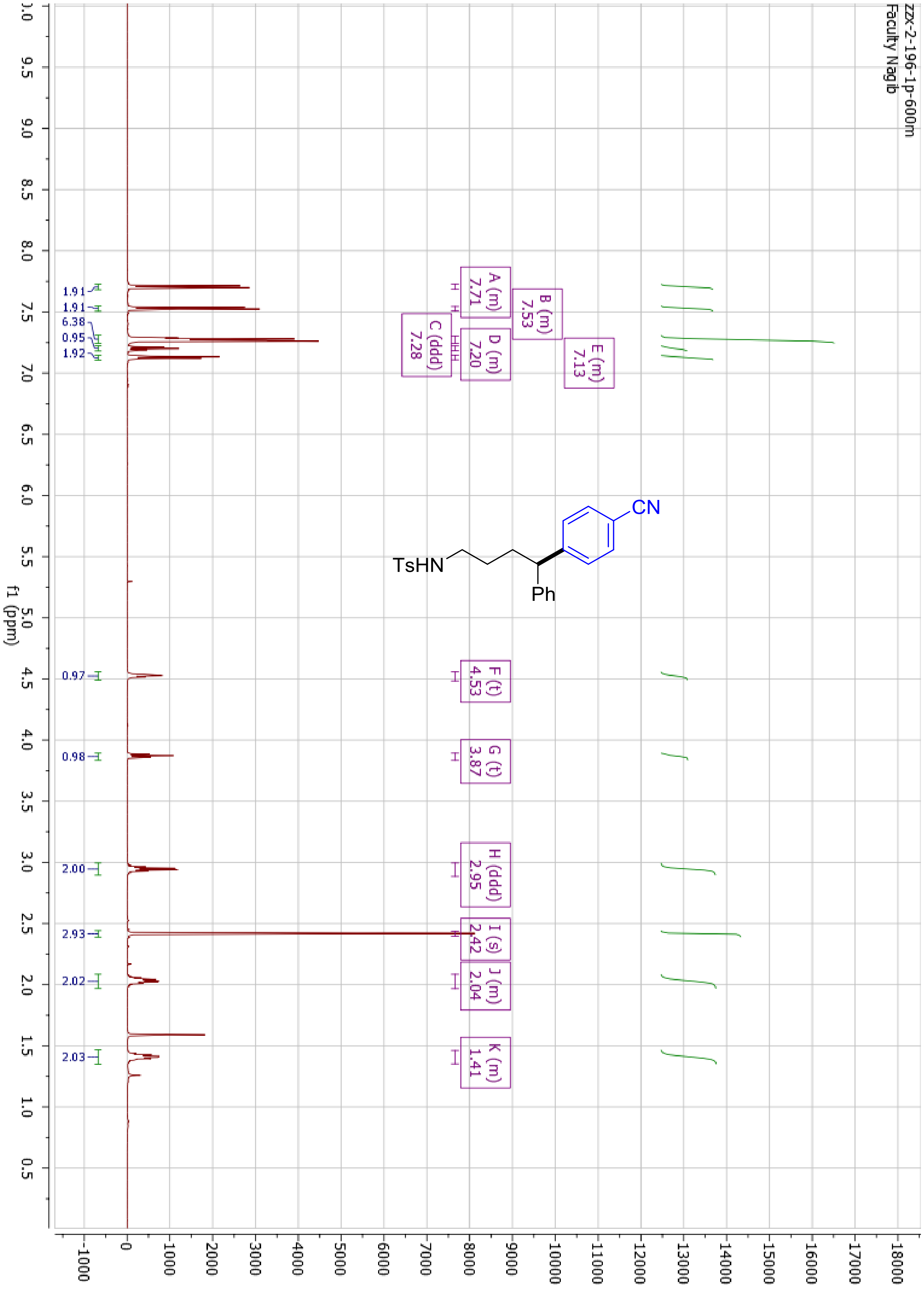
zzx-2-196-1p-600m
Faculty Nagib
TsHN
CN
Ph
A (m) 7.71
B (m) 7.53
C (ddd) 7.28
D (m) 7.20
E (m) 7.13
F (t) 4.53
G (t) 3.87
H (ddd) 2.95
I (s) 2.42
J (m) 2.04
K (m) 1.41
1.91
1.91
6.38
0.95
1.92
0.97
0.98
2.00
2.93
2.02
2.03
f1 (ppm)

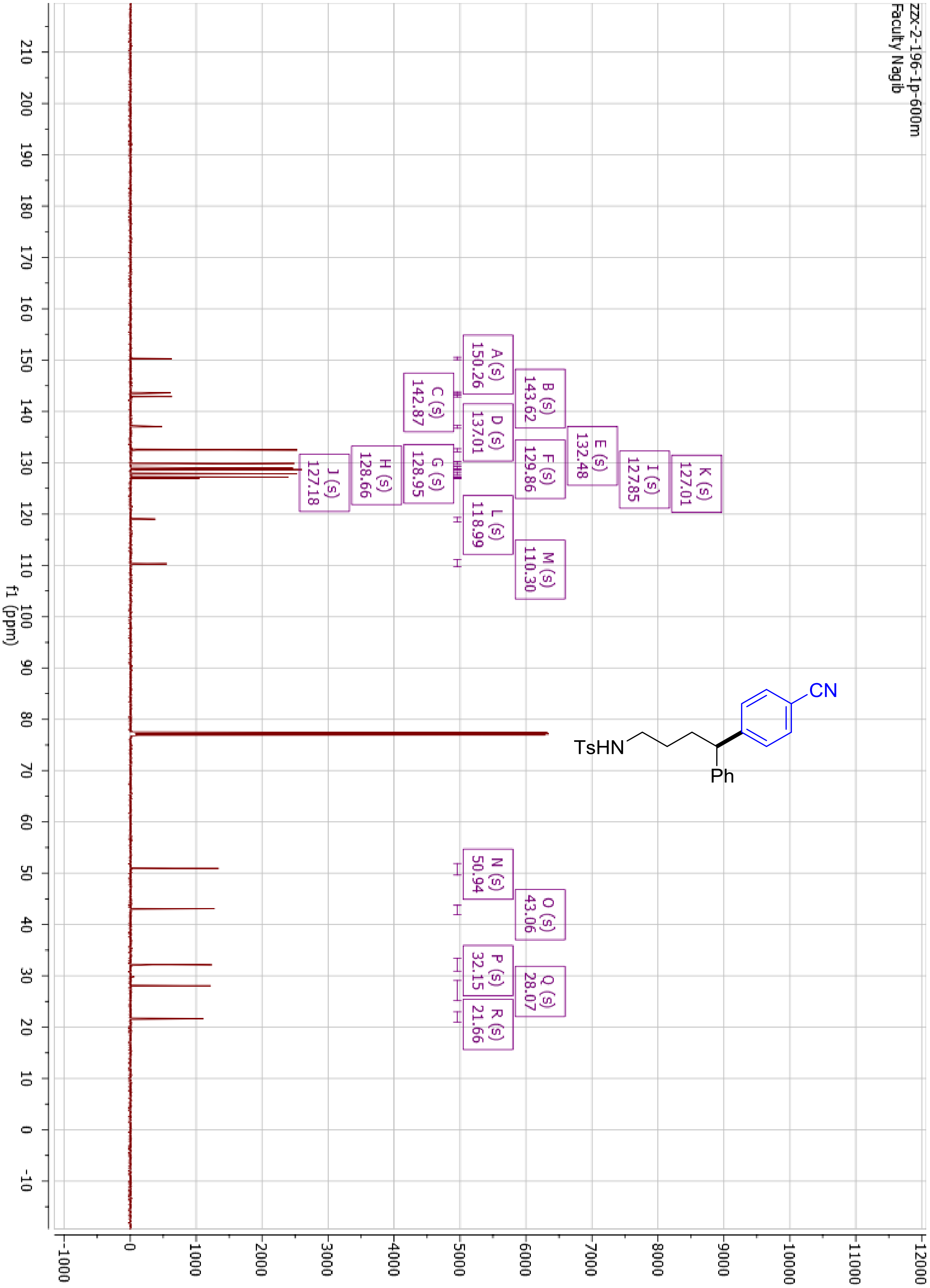
zzx-2-196-1p-600m
Faculty Nagib
A (s) 150.26
B (s) 143.62
C (s) 142.87
D (s) 137.01
E (s) 132.48
F (s) 129.86
G (s) 128.95
H (s) 128.66
I (s) 127.85
J (s) 127.18
K (s) 127.01
L (s) 118.99
M (s) 110.30
N (s) 50.94
O (s) 43.06
P (s) 32.15
Q (s) 28.07
R (s) 21.66
CN
TsHN
Ph
f1 (ppm)

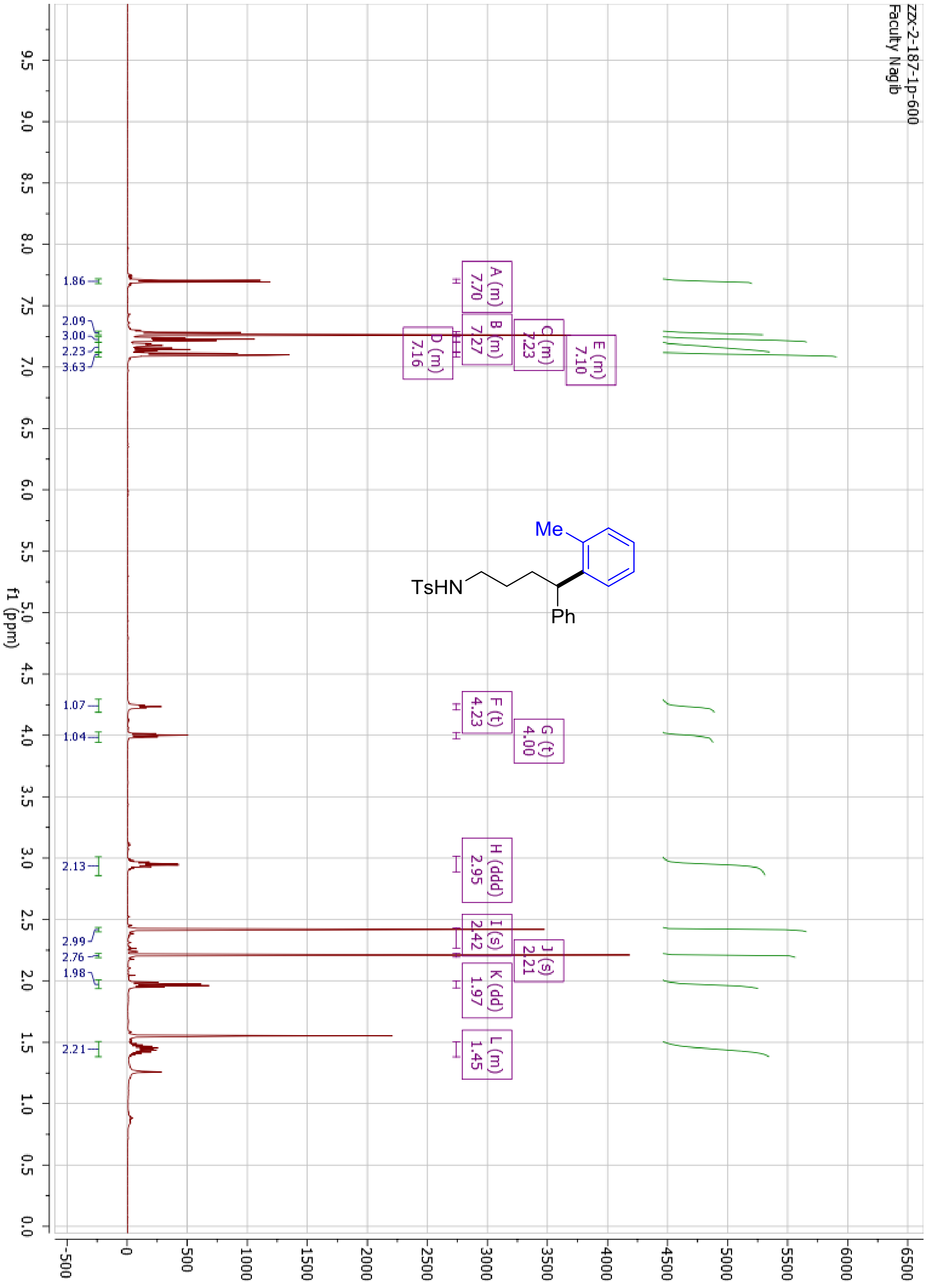
zzx-2-187-1p-600
Faculty Nagib
A (m) 7.70
B (dd) 7.27
C (m) 7.23
D (m) 7.16
E (m) 7.10
F (t) 4.23
G (t) 4.00
H (ddd) 2.95
I (s) 2.42
J (s) 2.21
K (dd) 1.97
L (m) 1.45
1.86
2.09
3.00
2.23
3.63
1.07
1.04
2.13
2.99
2.76
1.98
2.21
Me
TsHN
Ph
f1 (ppm)

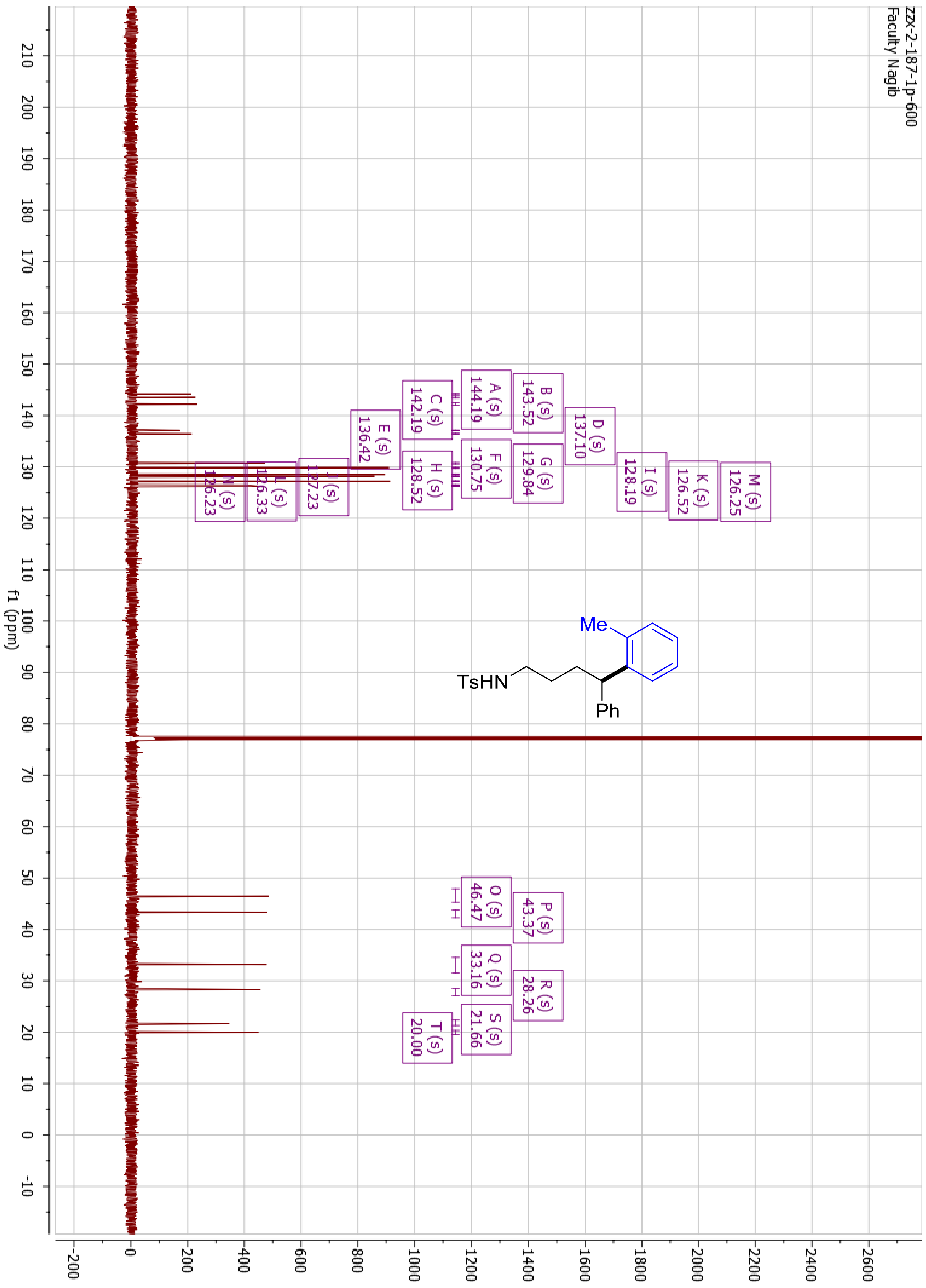
zzx-2-187-1p-600
Faculty Nagib
A (s) 144.19
B (s) 143.52
C (s) 142.19
D (s) 137.10
E (s) 136.42
F (s) 130.75
G (s) 129.84
H (s) 128.52
I (s) 128.19
(s) 127.23
K (s) 126.52
L (s) 126.33
M (s) 126.25
N (s) 126.23
O (s) 46.47
P (s) 43.37
Q (s) 33.16
R (s) 28.26
S (s) 21.66
T (s) 20.00
Me
TsHN
Ph
f1 (ppm)

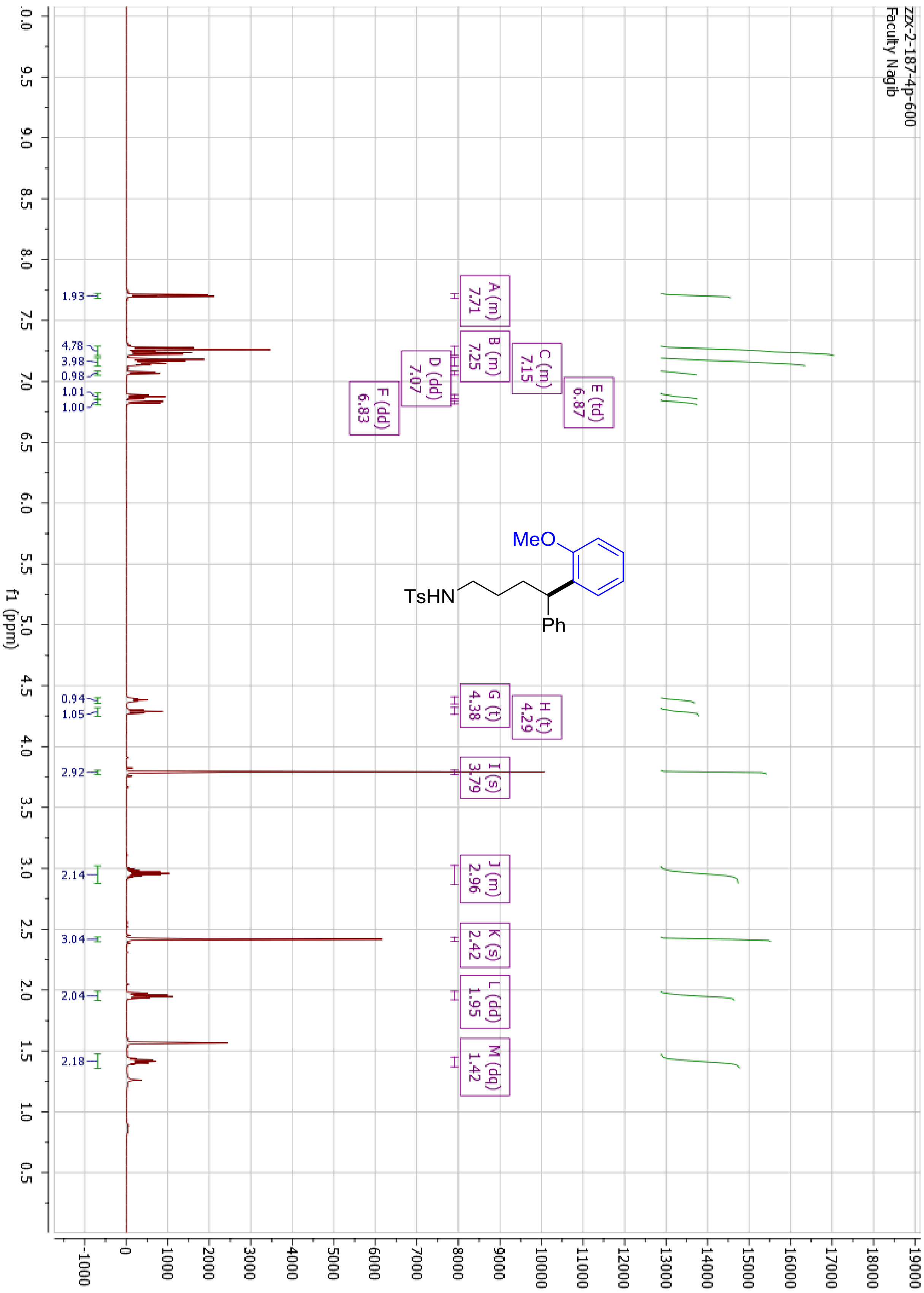
zzx-2-187-4p-600
Faculty Nagib
A (m) 7.71
B (m) 7.25
C (m) 7.15
D (dd) 7.07
E (td) 6.87
F (dd) 6.83
G (t) 4.38
H (t) 4.29
I (s) 3.79
J (m) 2.96
K (s) 2.42
L (dd) 1.95
M (dq) 1.42
1.93
4.78
3.98
0.98
1.01
1.00
0.94
1.05
2.92
2.14
3.04
2.04
2.18
MeO
TsHN
Ph
f1 (ppm)

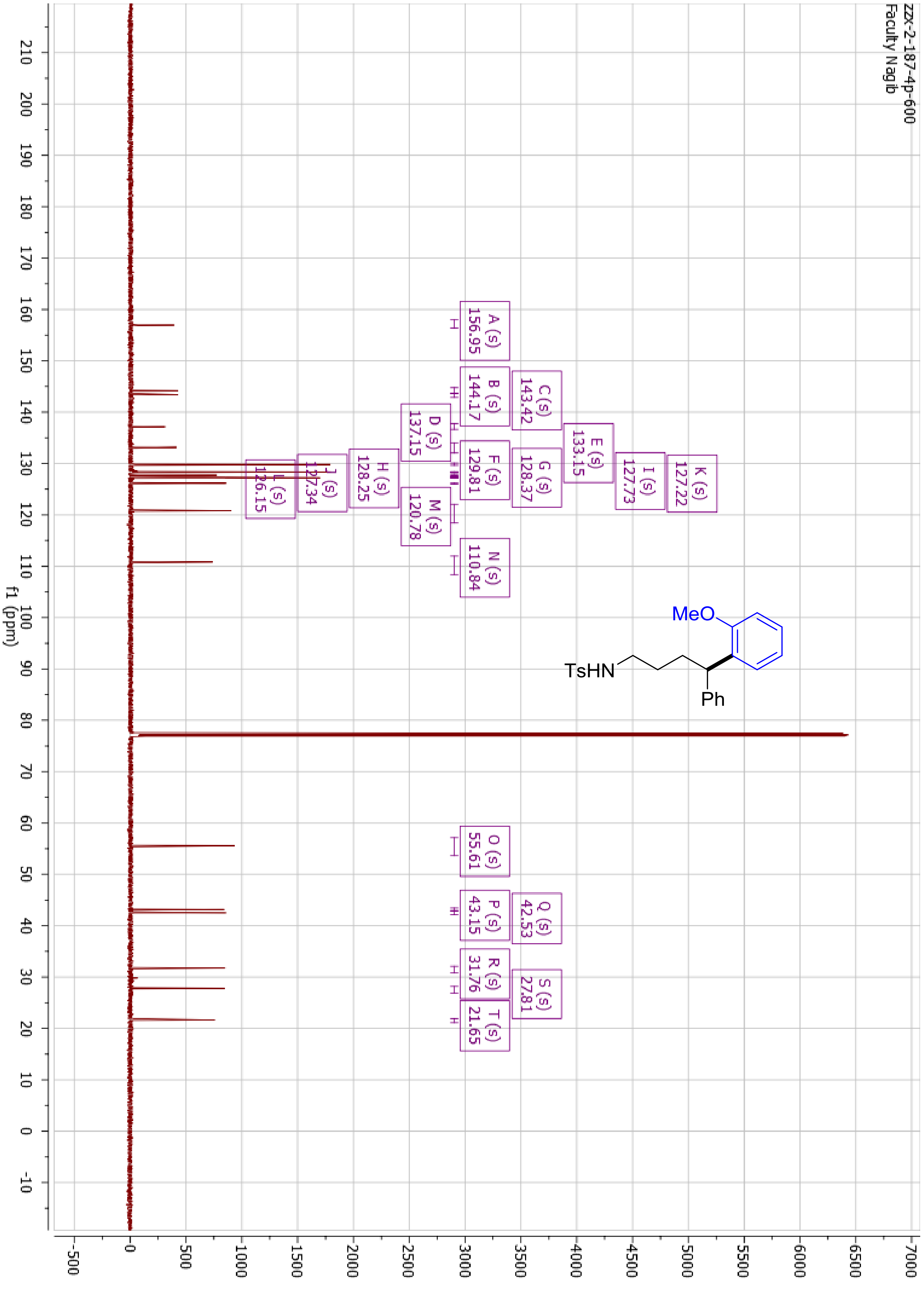
ZZX-2-187-4p-600
Faculty Nagib
A (s) 156.95
B (s) 144.17
C (s) 143.42
D (s) 137.15
E (s) 133.15
F (s) 129.81
G (s) 128.37
H (s) 128.25
I (s) 127.73
J (s) 127.34
K (s) 127.22
L (s) 126.15
M (s) 120.78
N (s) 110.84
O (s) 55.61
P (s) 43.15
Q (s) 42.53
R (s) 31.76
S (s) 27.81
T (s) 21.65
MeO
TsHN
Ph
f1 (ppm)

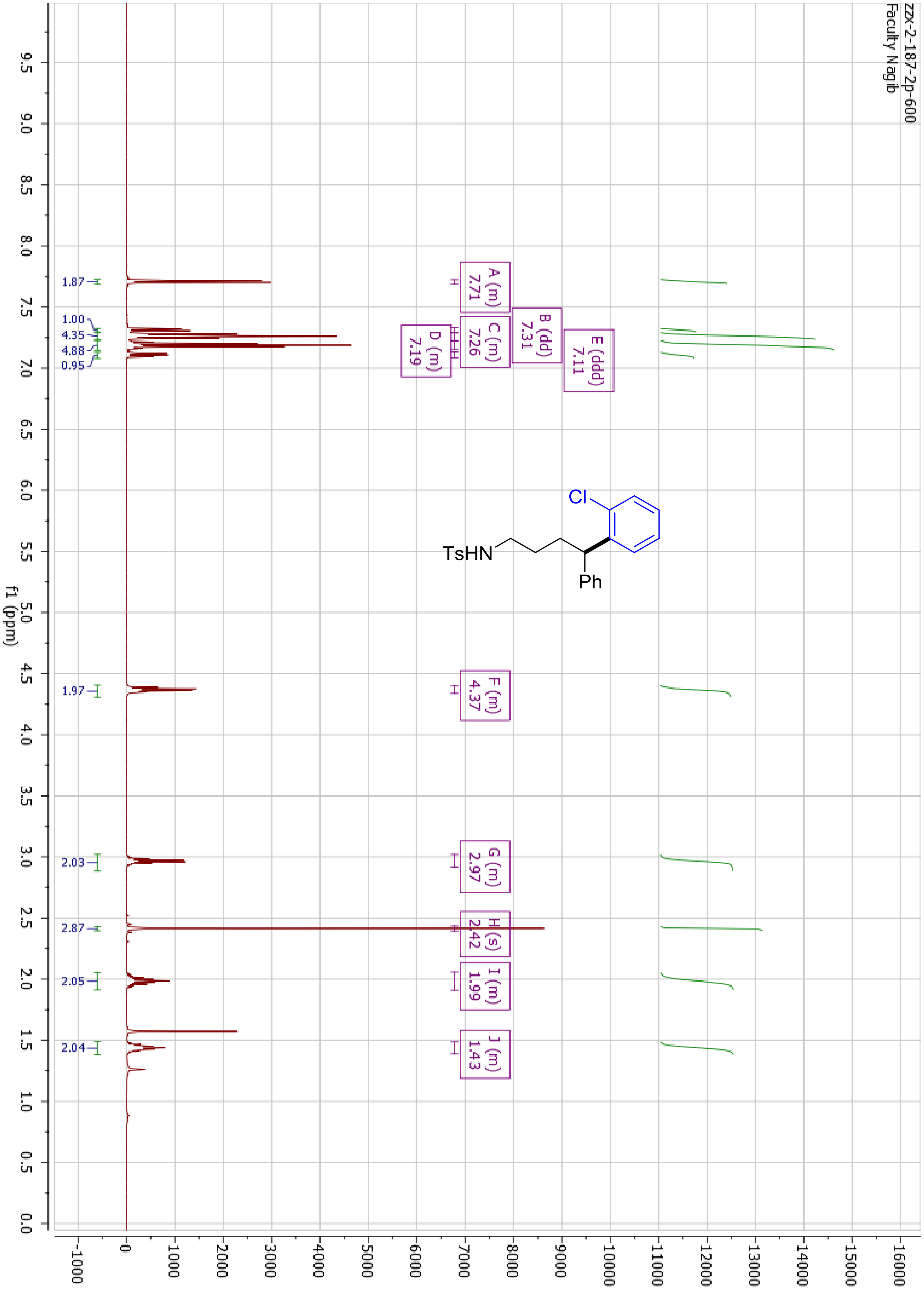

zzx-2-187-2p-600
Faculty Nagib
A (m) 7.71
B (dd) 7.31
C (m) 7.26
D (m) 7.19
E (ddd) 7.11
F (m) 4.37
G (m) 2.97
H (s) 2.42
I (m) 1.99
J (m) 1.43
1.87
1.00
4.35
4.88
0.95
1.97
2.03
2.87
2.05
2.04
TsHN
Cl
Ph
f1 (ppm)

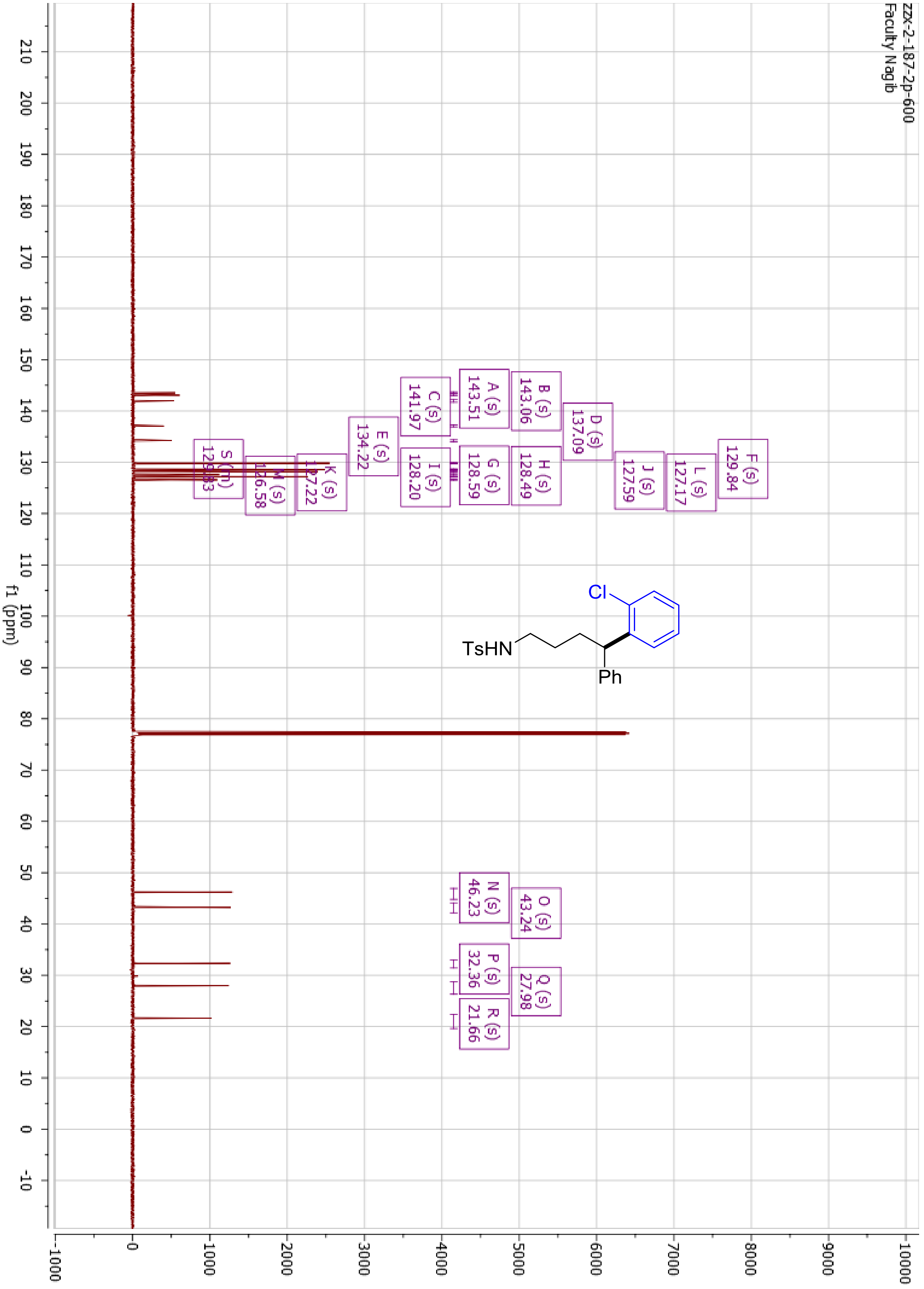
zzx-2-187-2p-600
Faculty Nagib
A (s) 143.51
B (s) 143.06
C (s) 141.97
D (s) 137.09
E (s) 134.22
F (s) 129.84
G (s) 128.59
H (s) 128.49
I (s) 128.20
J (s) 127.59
K (s) 127.22
L (s) 127.17
M (s) 126.58
N (s) 46.23
O (s) 43.24
P (s) 32.36
Q (s) 27.98
R (s) 21.66
S (m)
Cl
TsHN
Ph
f1 (ppm)

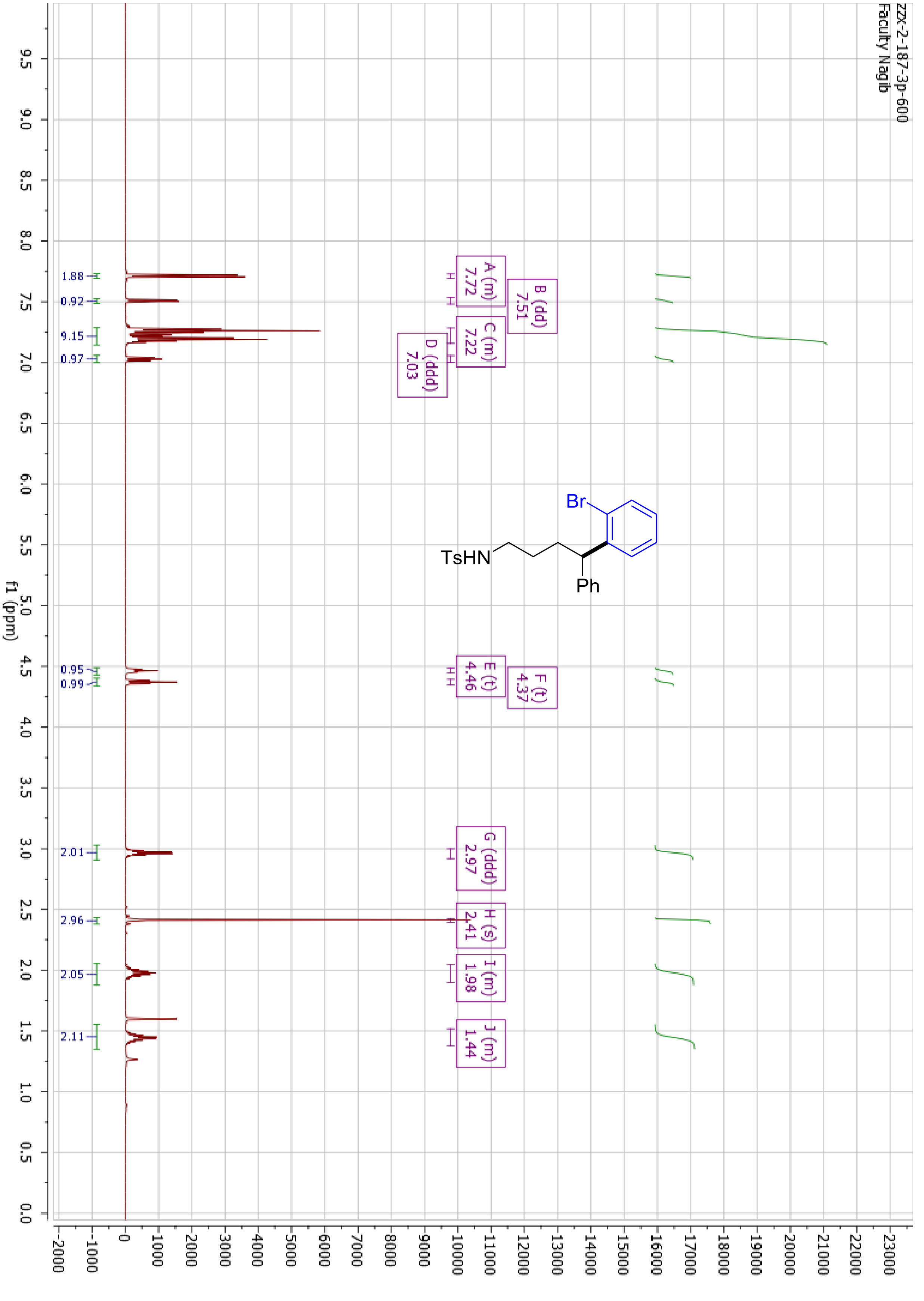
ZZX-2-187-3p-600
Faculty Nagib
TsHN
Br
Ph
A (m) 7.72
B (dd) 7.51
C (m) 7.22
D (ddd) 7.03
E (t) 4.46
F (t) 4.37
G (ddd) 2.97
H (s) 2.41
I (m) 1.98
J (m) 1.44
1.88
0.92
9.15
0.97
0.95
0.99
2.01
2.96
2.05
2.11
f1 (ppm)

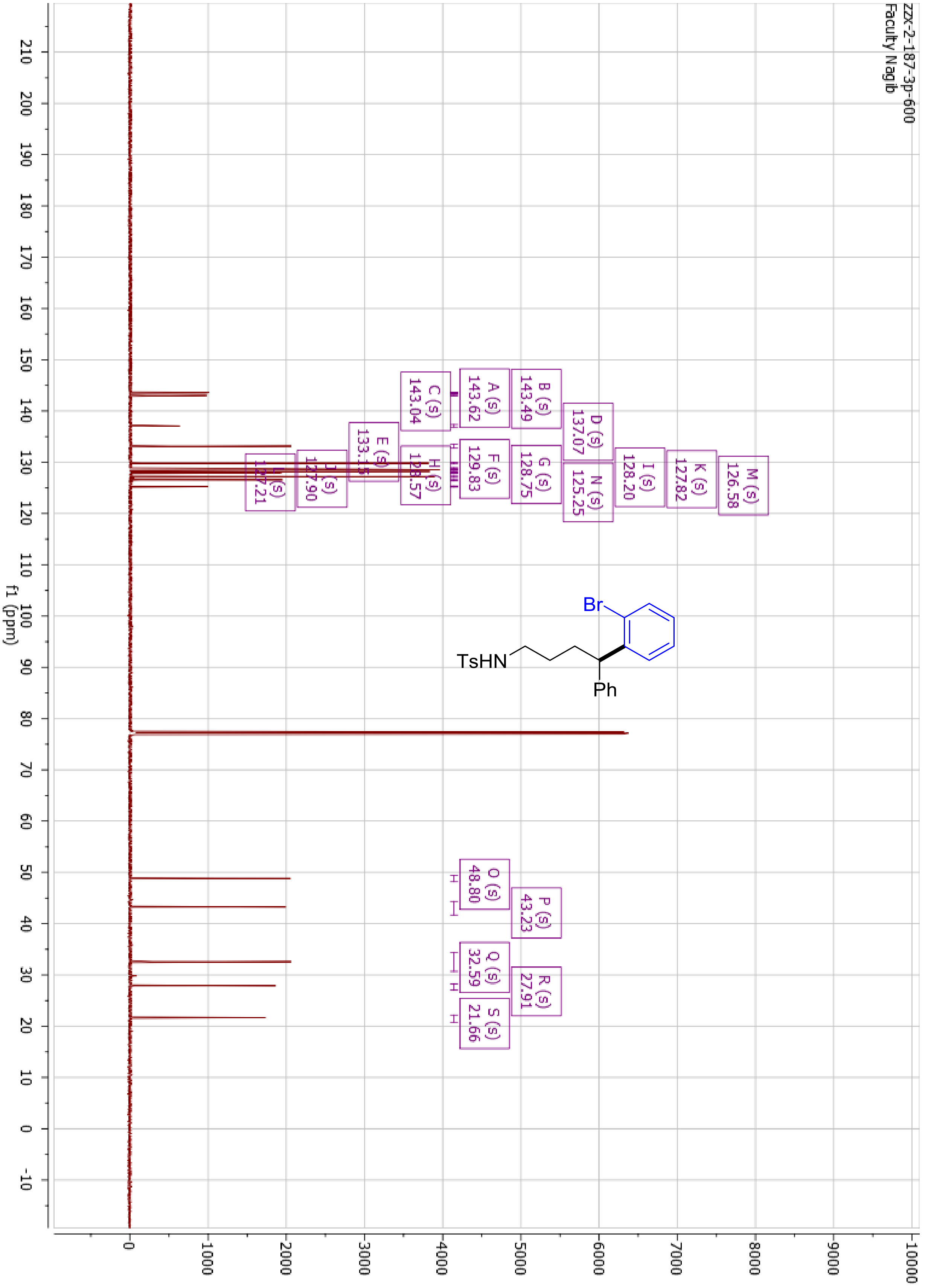

ZZX-2-187-3p-600
Faculty Nagib
A (s) 143.62
B (s) 143.49
C (s) 143.04
D (s) 137.07
F (s) 129.83
G (s) 128.75
H (s) 128.57
I (s) 128.20
K (s) 127.82
M (s) 126.58
N (s) 125.25
O (s) 48.80
P (s) 43.23
Q (s) 32.59
R (s) 27.91
S (s) 21.66
Br
TsHN
Ph
f1 (ppm)

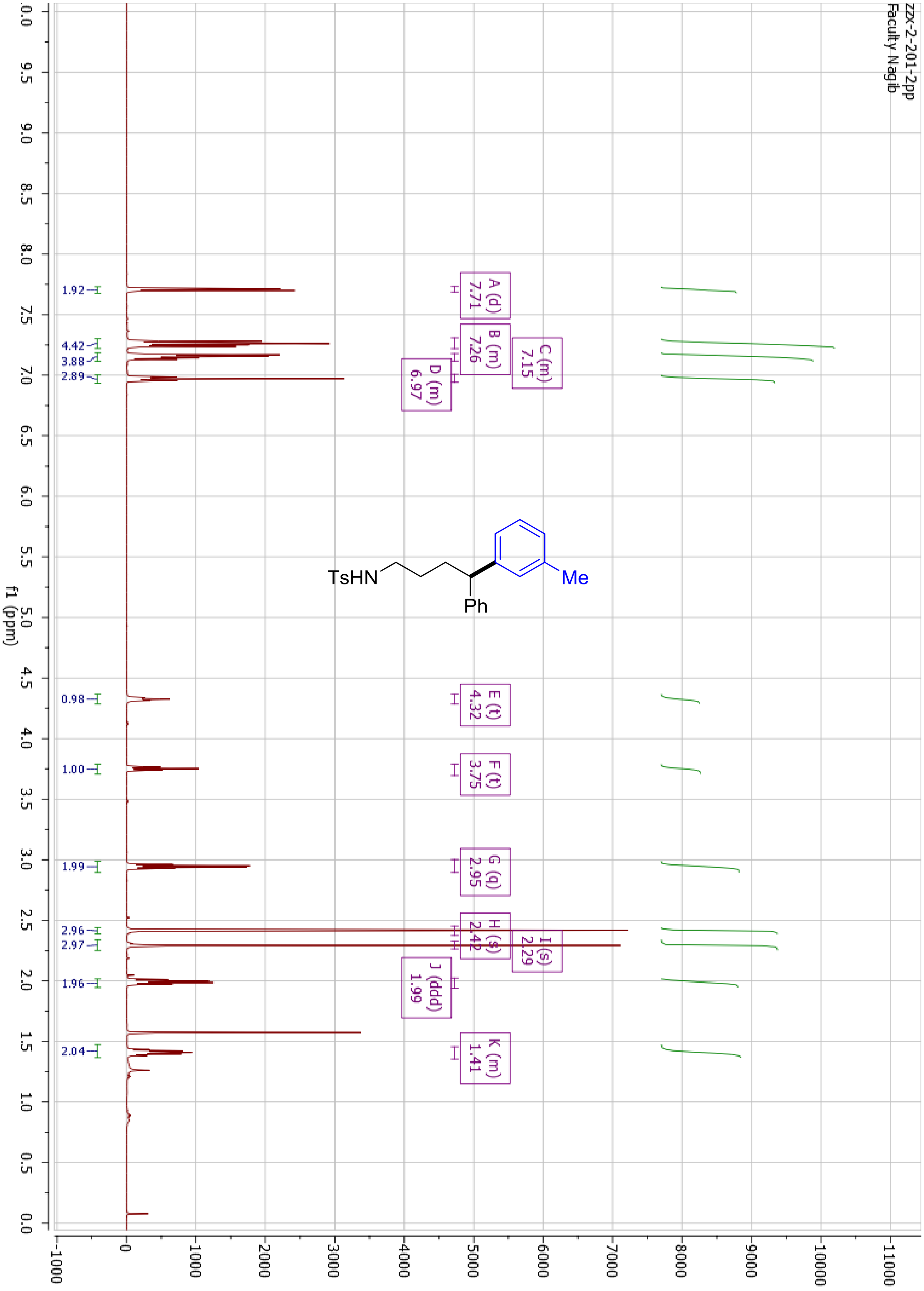
zzx-2-201-2pp
Faculty Nagib
TsHN
Me
Ph
A (d) 7.71
B (m) 7.26
C (m) 7.15
D (m) 6.97
E (t) 4.32
F (t) 3.75
G (q) 2.95
H (s) 2.42
I (s) 2.29
J (ddd) 1.99
K (m) 1.41
1.92
4.42
3.88
2.89
0.98
1.00
1.99
2.96
2.97
1.96
2.04
f1 (ppm)

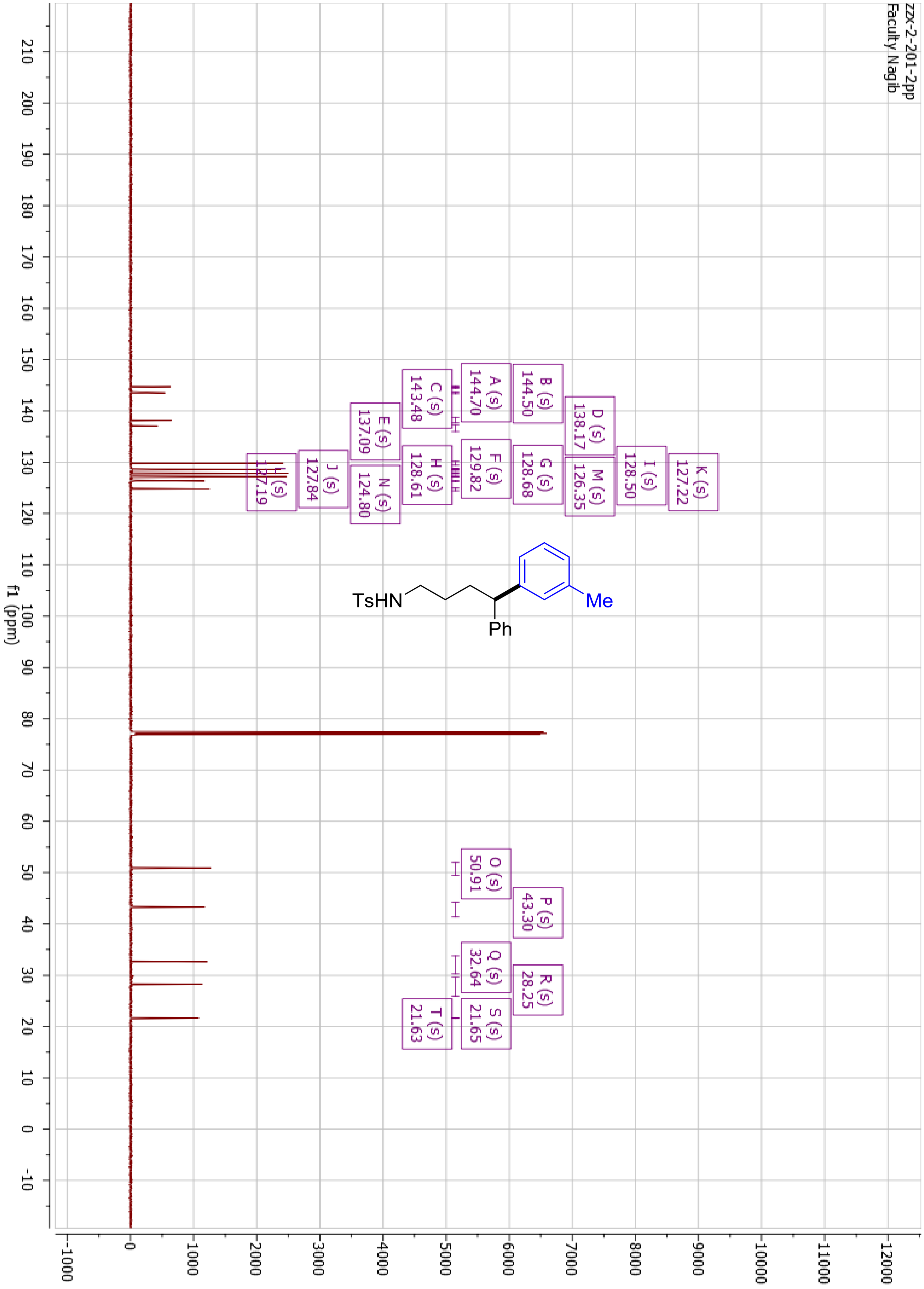
zzx-2-201-2pp
Faculty Nagib
A (s) 144.70
B (s) 144.50
C (s) 143.48
D (s) 138.17
E (s) 137.09
F (s) 129.82
G (s) 128.68
H (s) 128.61
I (s) 128.50
J (s) 127.84
K (s) 127.22
L (s) 127.19
M (s) 126.35
N (s) 124.80
O (s) 50.91
P (s) 43.30
Q (s) 32.64
R (s) 28.25
S (s) 21.65
T (s) 21.63
TsHN
Ph
Me
f1 (ppm)

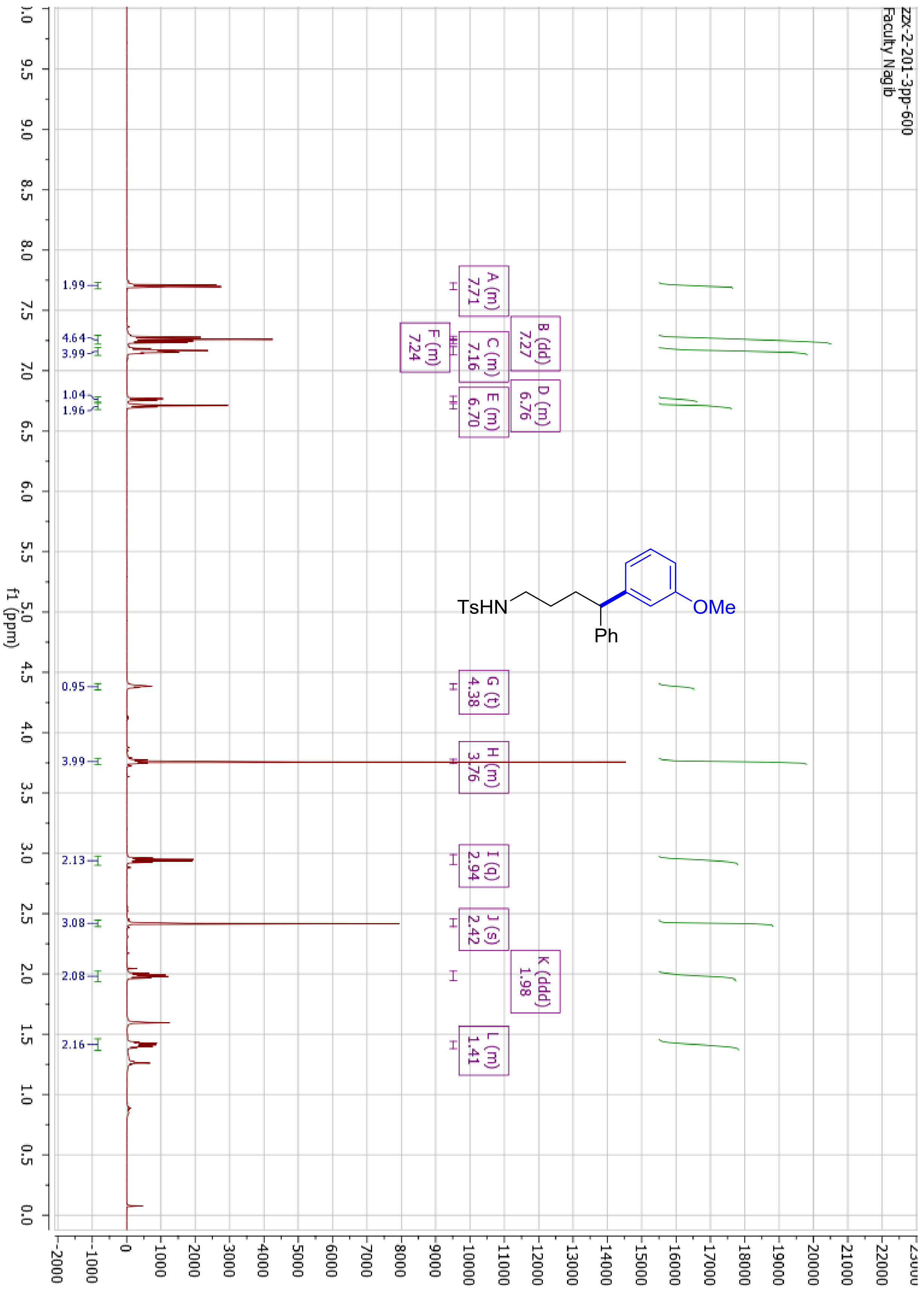
ZZX-2-201-3pp-600
Faculty Nagib
TsHN
Ph
OMe
A (m) 7.71
B (dd) 7.27
C (m) 7.16
F (m) 7.24
D (m) 6.76
E (m) 6.70
G (t) 4.38
H (m) 3.76
I (q) 2.94
J (s) 2.42
K (ddd) 1.98
L (m) 1.41
1.99
4.64
3.99
1.04
1.96
0.95
3.99
2.13
3.08
2.08
2.16
f1 (ppm)

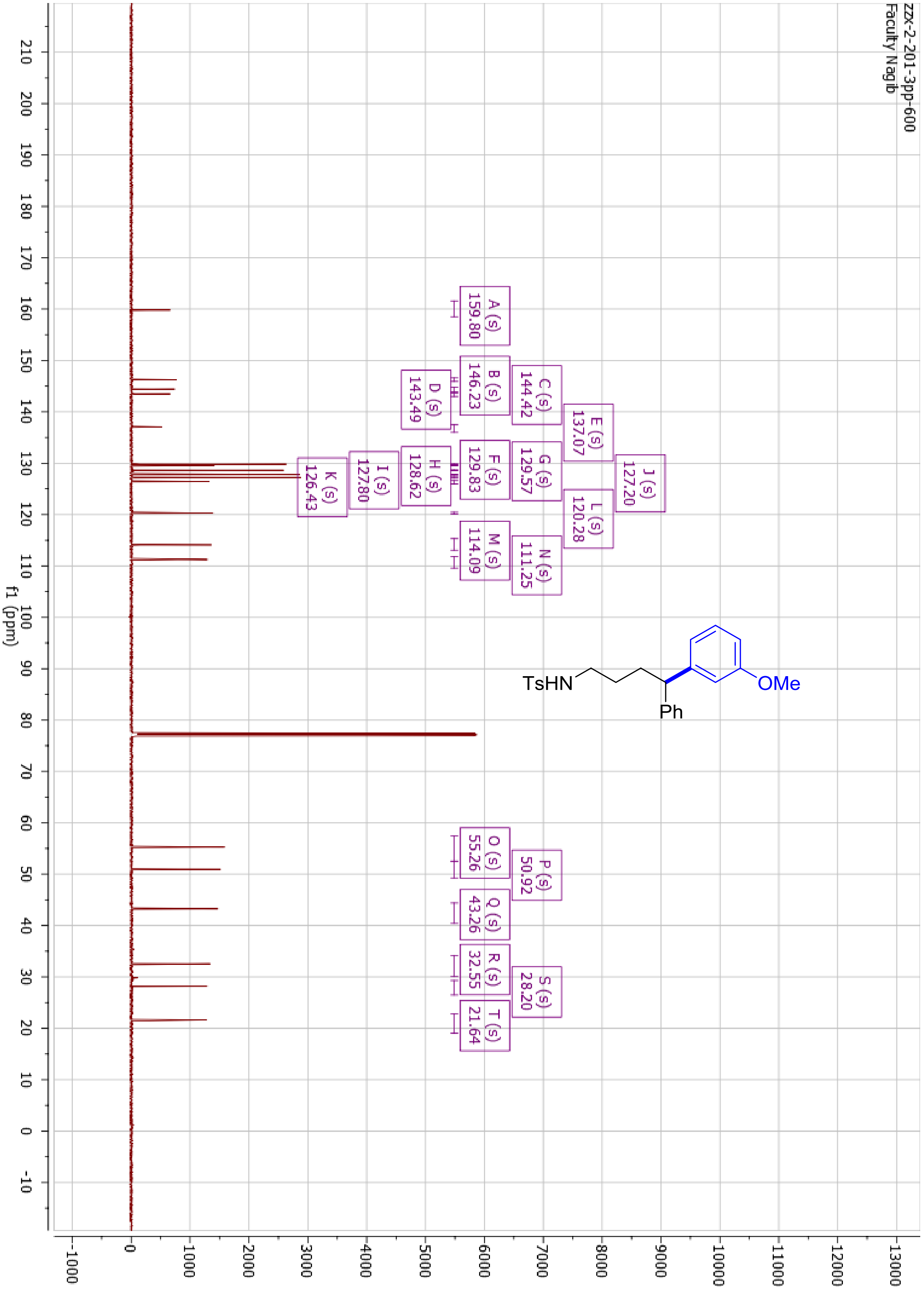
zzx-2-201-3pp-600
Faculty Nagib
A (s) 159.80
B (s) 146.23
C (s) 144.42
D (s) 143.49
E (s) 137.07
F (s) 129.83
G (s) 129.57
H (s) 128.62
I (s) 127.80
J (s) 127.20
K (s) 126.43
L (s) 120.28
M (s) 114.09
N (s) 111.25
O (s) 55.26
P (s) 50.92
Q (s) 43.26
R (s) 32.55
S (s) 28.20
T (s) 21.64
TsHN
Ph
OMe
f1 (ppm)

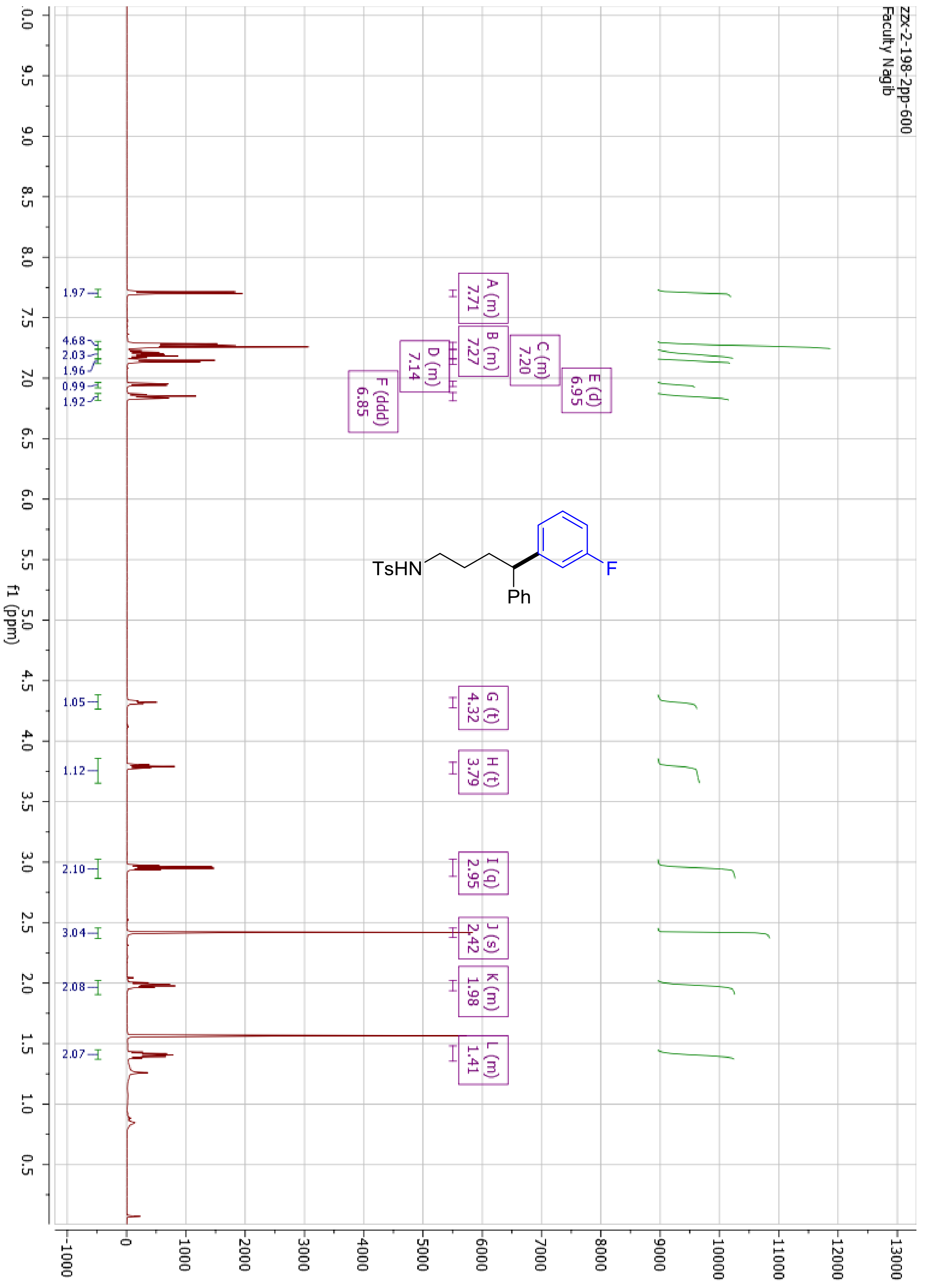
zzx-2-198-2pp-600
Faculty Nagib
TsHN
Ph
F
A (m) 7.71
B (m) 7.27
C (m) 7.20
D (m) 7.14
E (d) 6.95
F (ddd) 6.85
G (t) 4.32
H (t) 3.79
I (q) 2.95
J (s) 2.42
K (m) 1.98
L (m) 1.41
1.97
4.68
2.03
1.96
0.99
1.92
1.05
1.12
2.10
3.04
2.08
2.07
f1 (ppm)

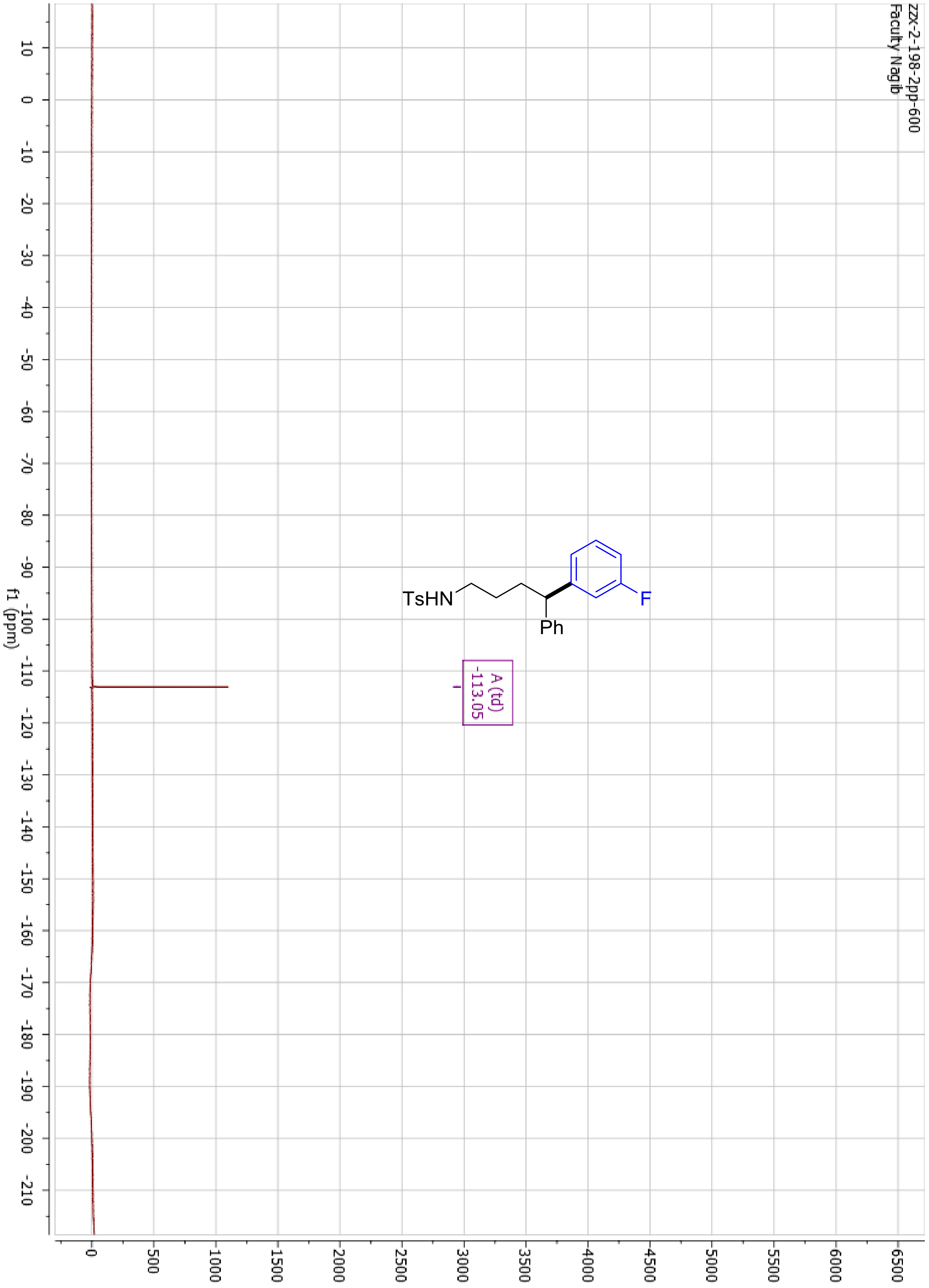
zzx-2-198-2pp-600
Faculty Nagib
TsHN
Ph
F
A (td)
-113.05
f1 (ppm)

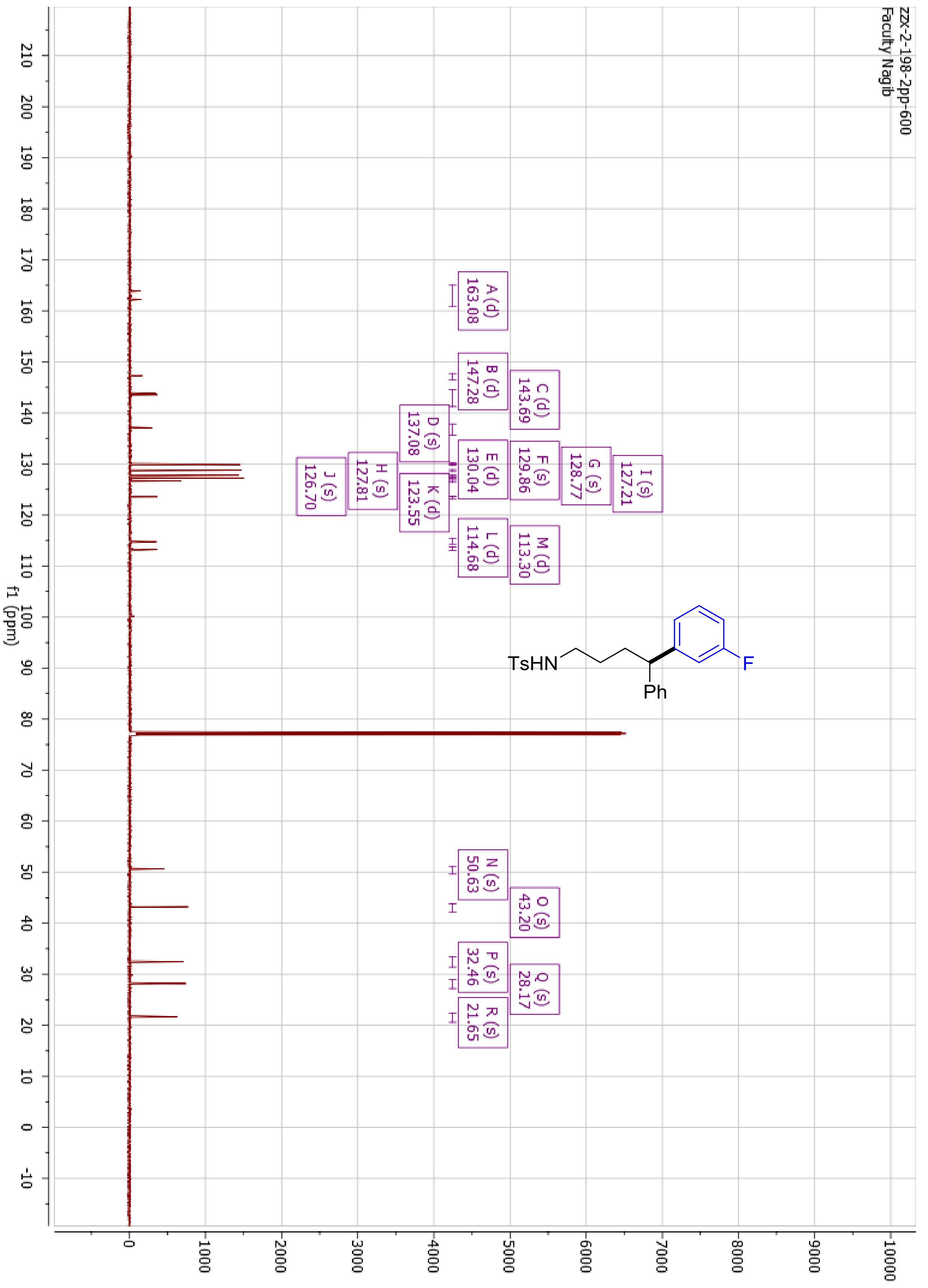
ZZX-2-198-2pp-600
Faculty Nagib
A (d) 163.08
B (d) 147.28
C (d) 143.69
D (s) 137.08
E (d) 130.04
F (s) 129.86
G (s) 128.77
H (s) 127.81
I (s) 127.21
J (s) 126.70
K (d) 123.55
L (d) 114.68
M (d) 113.30
N (s) 50.63
O (s) 43.20
P (s) 32.46
Q (s) 28.17
R (s) 21.65
TsHN
Ph
F
f1 (ppm)

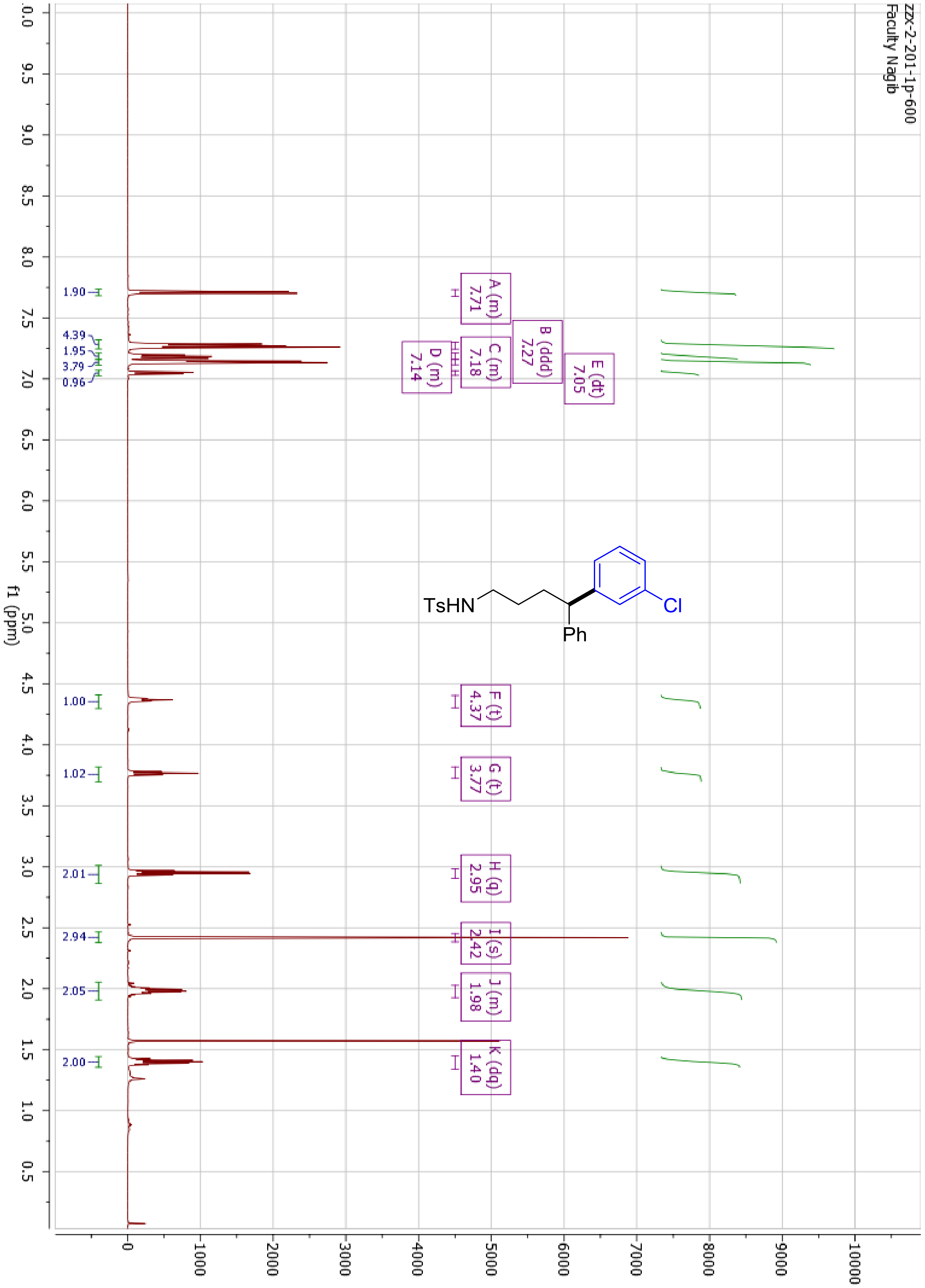
zzx-2-201-1p-600
Faculty Nagib
A (m)
7.71
B (ddd)
7.27
C (m)
7.18
D (m)
7.14
E (dt)
7.05
F (t)
4.37
G (t)
3.77
H (q)
2.95
I (s)
2.42
J (m)
1.98
K (dq)
1.40
TsHN
Ph
Cl
f1 (ppm)

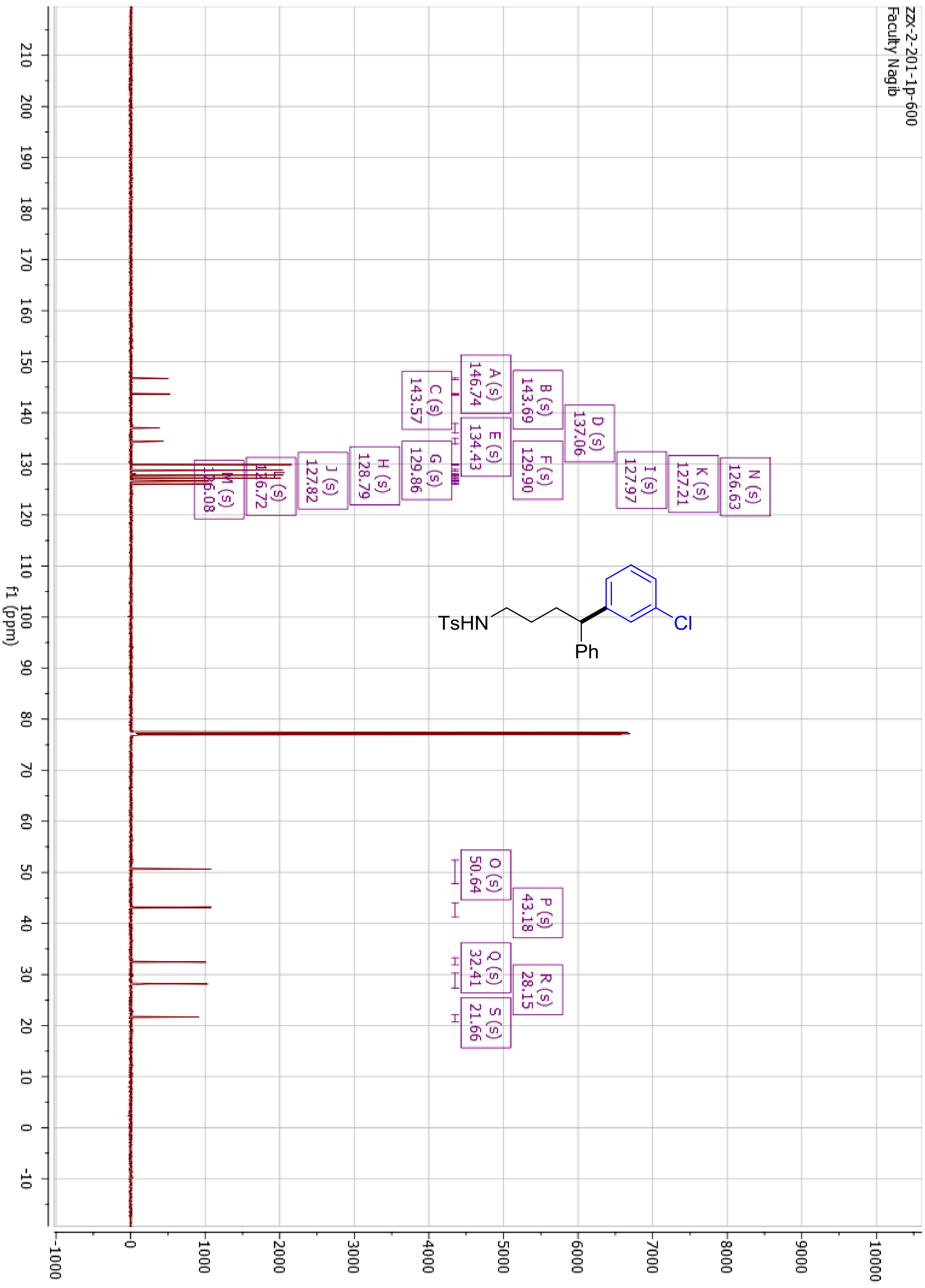
zzx-2-201-1p-600
Faculty Nagib
A (s) 146.74
B (s) 143.69
C (s) 143.57
D (s) 137.06
E (s) 134.43
F (s) 129.90
G (s) 129.86
H (s) 128.79
I (s) 127.97
J (s) 127.82
K (s) 127.21
L (s) 126.72
M (s) 126.08
N (s) 126.63
O (s) 50.64
P (s) 43.18
Q (s) 32.41
R (s) 28.15
S (s) 21.66
TsHN
Ph
Cl
f1 (ppm)

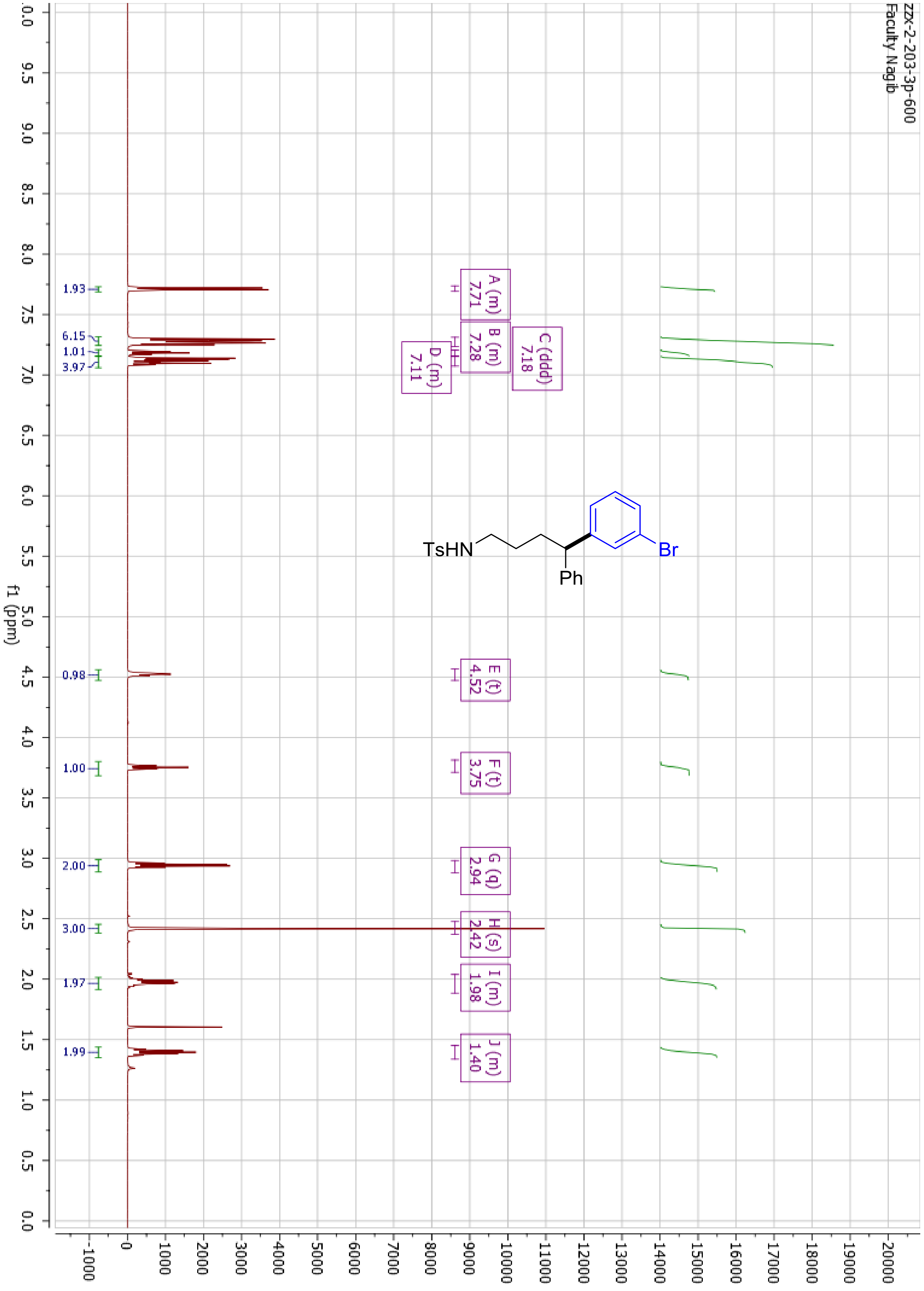
zzx-2-203-3p-600
Faculty Nagib
TsHN
Br
Ph
A (m) 7.71
B (m) 7.28
C (ddd) 7.18
D (m) 7.11
E (t) 4.52
F (t) 3.75
G (q) 2.94
H (s) 2.42
I (m) 1.98
J (m) 1.40
1.93
6.15
1.01
3.97
0.98
1.00
2.00
3.00
1.97
1.99
f1 (ppm)

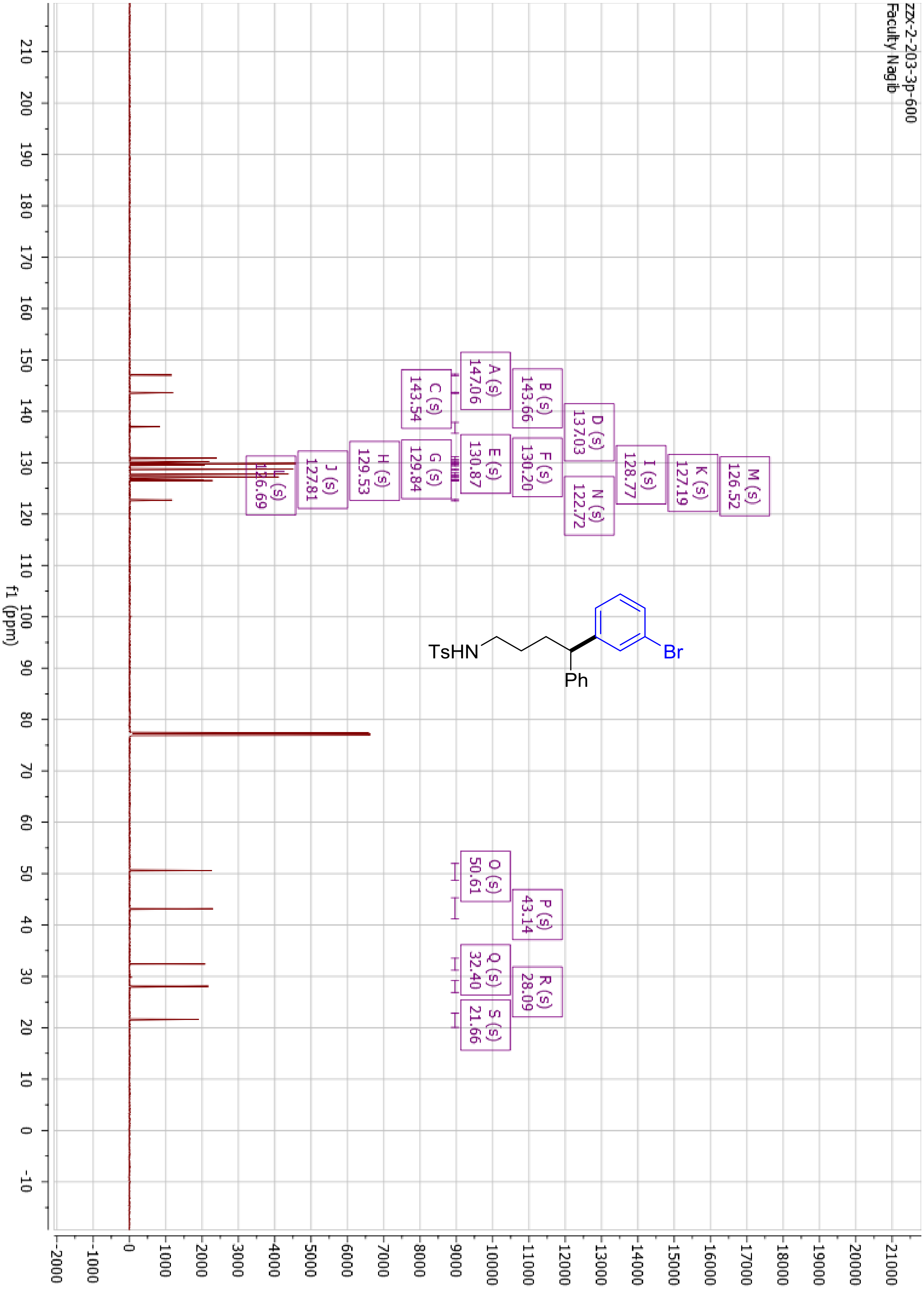

ZZX-2-203-3p-600
Faculty Nagib
A (s) 147.06
B (s) 143.66
C (s) 143.54
D (s) 137.03
E (s) 130.87
F (s) 130.20
G (s) 129.84
H (s) 129.53
I (s) 128.77
J (s) 127.81
K (s) 127.19
L (s) 126.69
M (s) 126.52
N (s) 122.72
O (s) 50.61
P (s) 43.14
Q (s) 32.40
R (s) 28.09
S (s) 21.66
TsHN
Ph
Br
f1 (ppm)

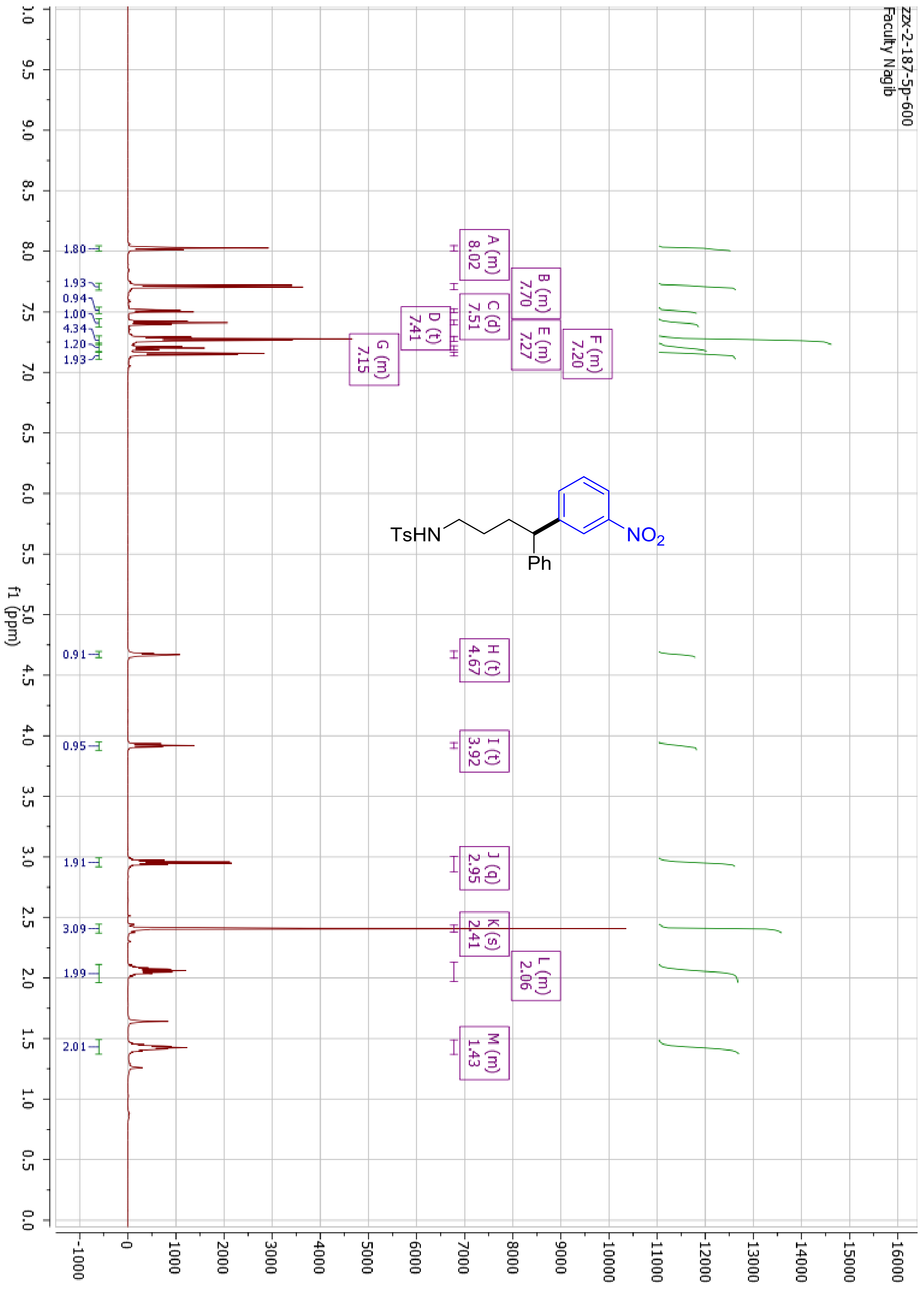
zzx-2-187-5p-600
Faculty Nagib
TsHN
Ph
NO2
f1 (ppm)
A (m) 8.02
B (m) 7.70
C (d) 7.51
D (t) 7.41
E (m) 7.27
F (m) 7.20
G (m) 7.15
H (t) 4.67
I (t) 3.92
J (q) 2.95
K (s) 2.41
L (m) 2.06
M (m) 1.43

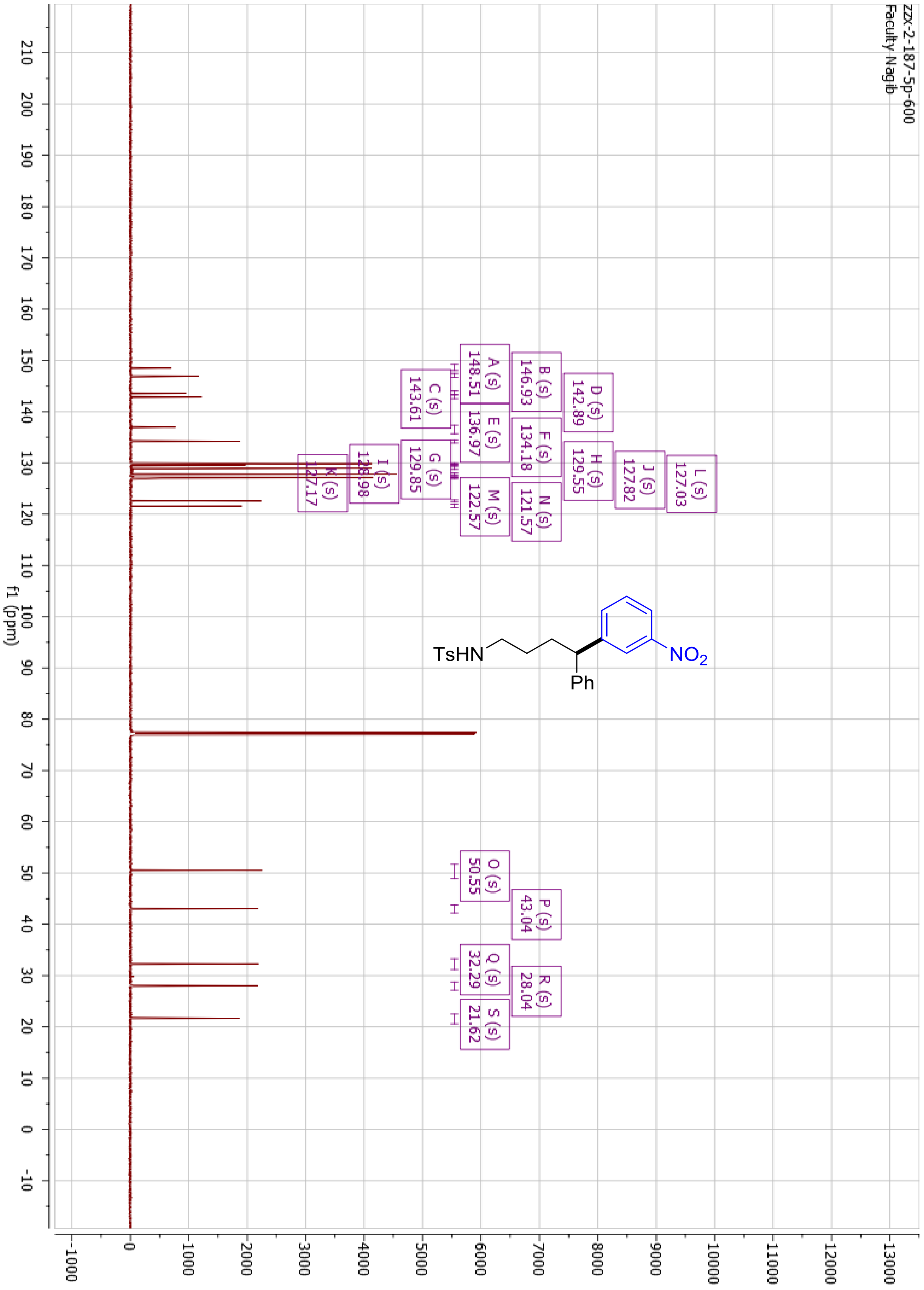

ZZX-2-187-5p-600
Faculty Nagib
TsHN
Ph
NO2
A (s) 148.51
B (s) 146.93
C (s) 143.61
D (s) 142.89
E (s) 136.97
F (s) 134.18
G (s) 129.85
H (s) 129.55
J (s) 127.82
L (s) 127.03
M (s) 122.57
N (s) 121.57
O (s) 50.55
P (s) 43.04
Q (s) 32.29
R (s) 28.04
S (s) 21.62
f1 (ppm)

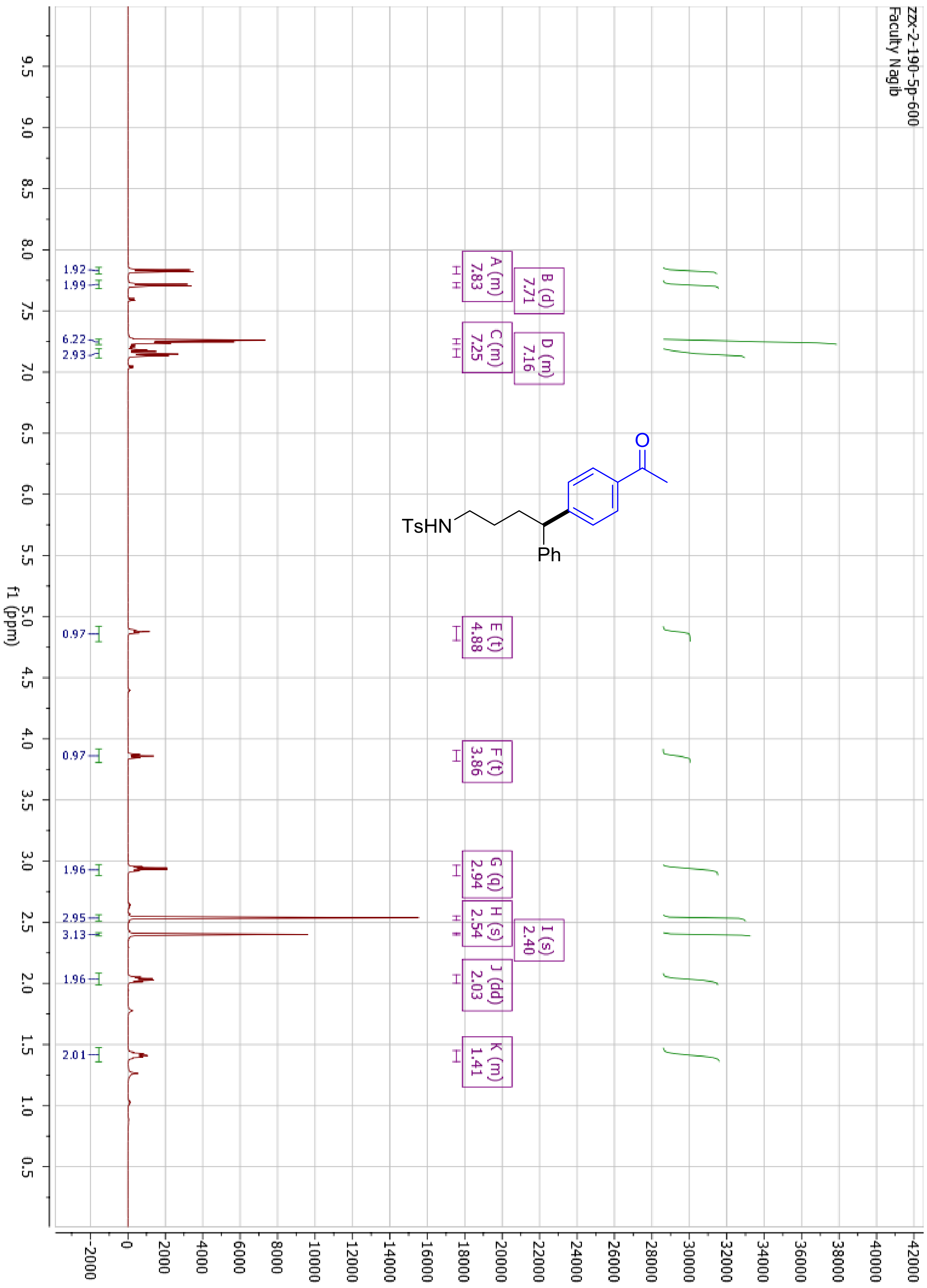
zzx-2-190-5p-600
Faculty Nagib
TsHN
Ph
O
A (m) 7.83
B (d) 7.71
C (m) 7.25
D (m) 7.16
E (t) 4.88
F (t) 3.86
G (q) 2.94
H (s) 2.54
I (s) 2.40
J (dd) 2.03
K (m) 1.41
1.92
1.99
6.22
2.93
0.97
0.97
1.96
2.95
3.13
1.96
2.01
f1 (ppm)

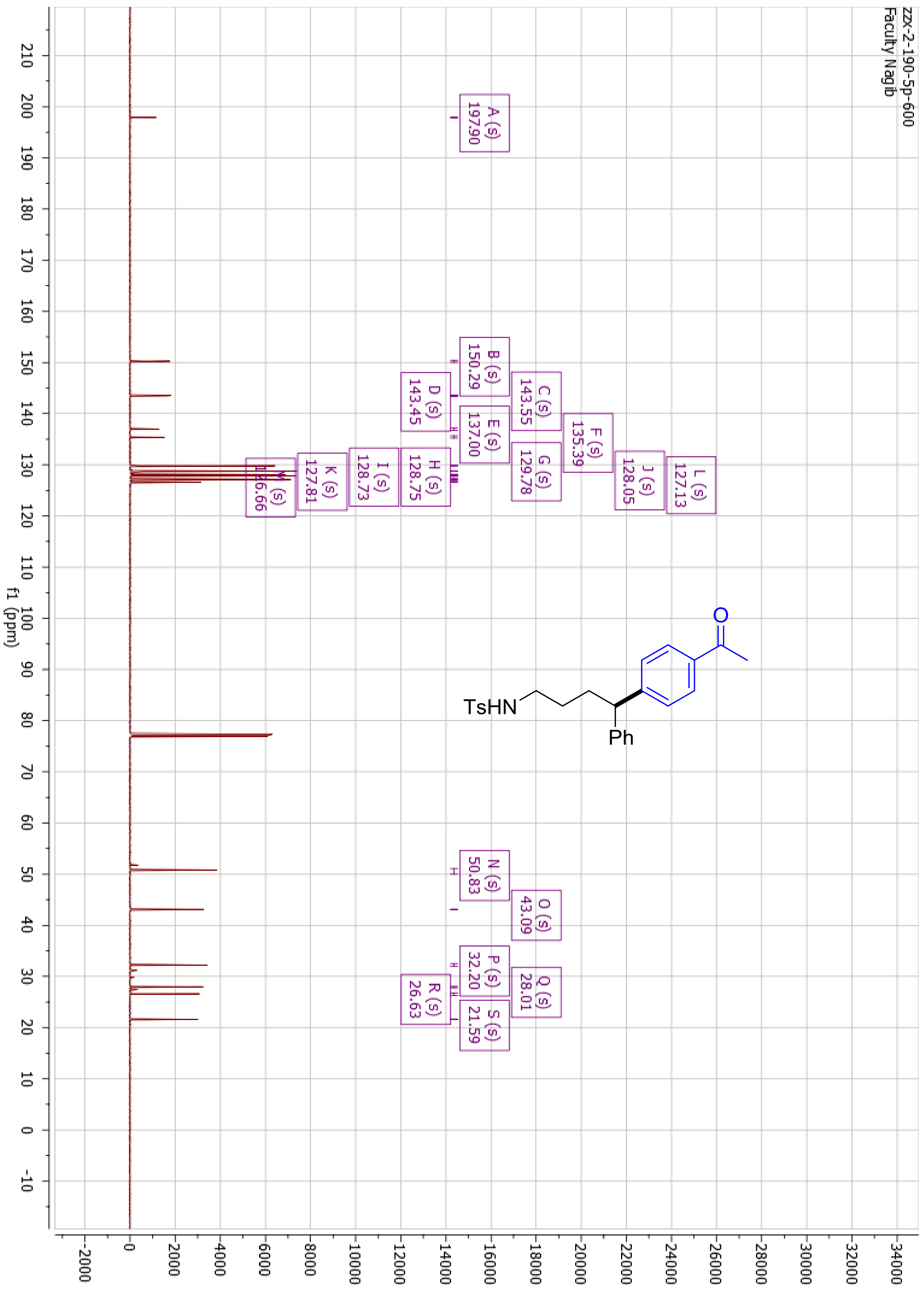
zzx-2-190-5p-600
Faculty Nagib
A (s) 197.90
B (s) 150.29
C (s) 143.55
D (s) 143.45
E (s) 137.00
F (s) 135.39
G (s) 129.78
H (s) 128.75
I (s) 128.73
J (s) 128.05
K (s) 127.81
L (s) 127.13
M (s) 126.66
N (s) 50.83
O (s) 43.09
P (s) 32.20
Q (s) 28.01
R (s) 26.63
S (s) 21.59
TsHN
Ph
O
f1 (ppm)

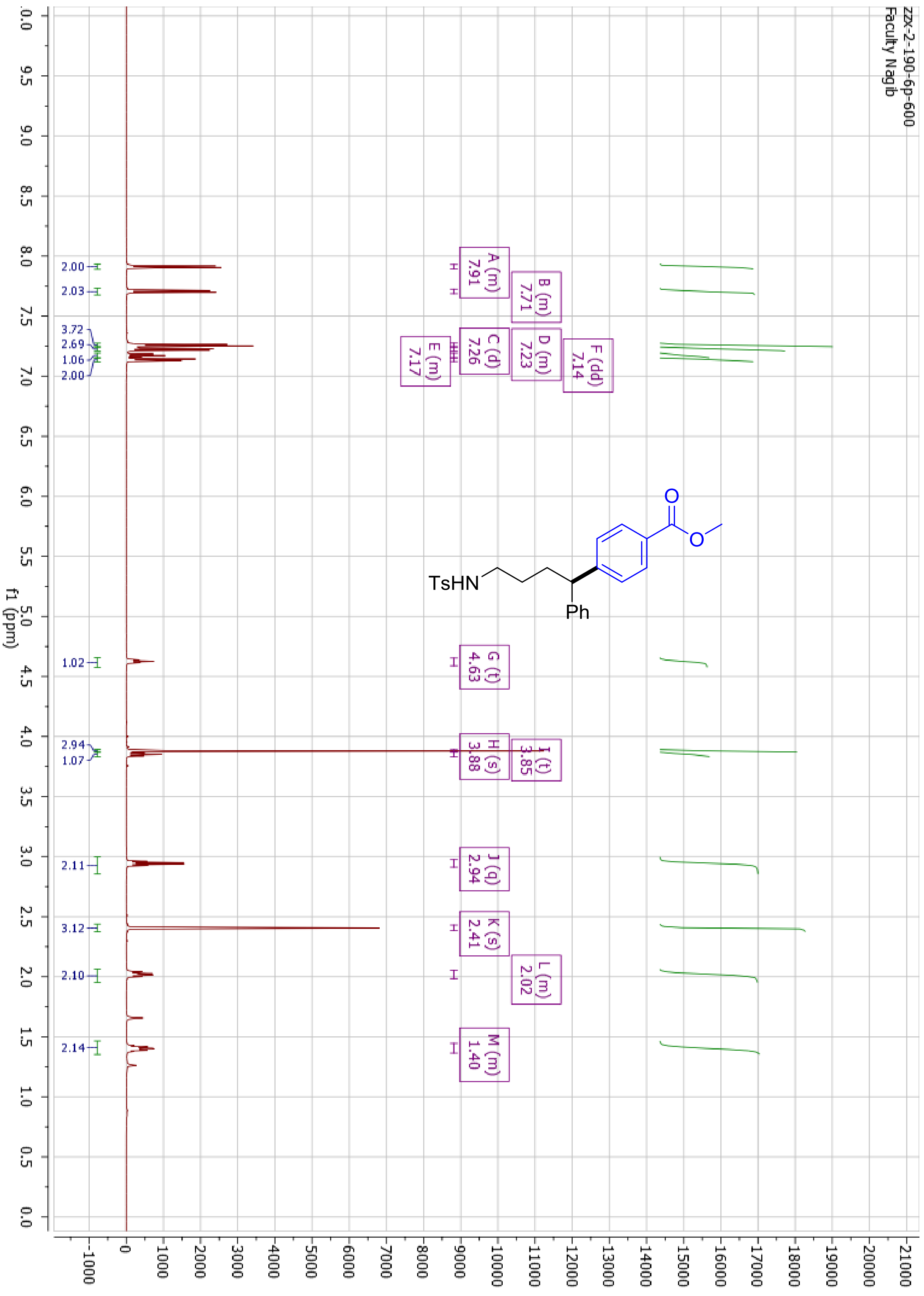
zzx-2-190-6p-600
Faculty Nagib
TsHN
Ph
O
O
A (m) 7.91
B (m) 7.71
C (d) 7.26
D (m) 7.23
E (m) 7.17
F (dd) 7.14
G (t) 4.63
H (s) 3.88
I (t) 3.85
J (q) 2.94
K (s) 2.41
L (m) 2.02
M (m) 1.40
f1 (ppm)

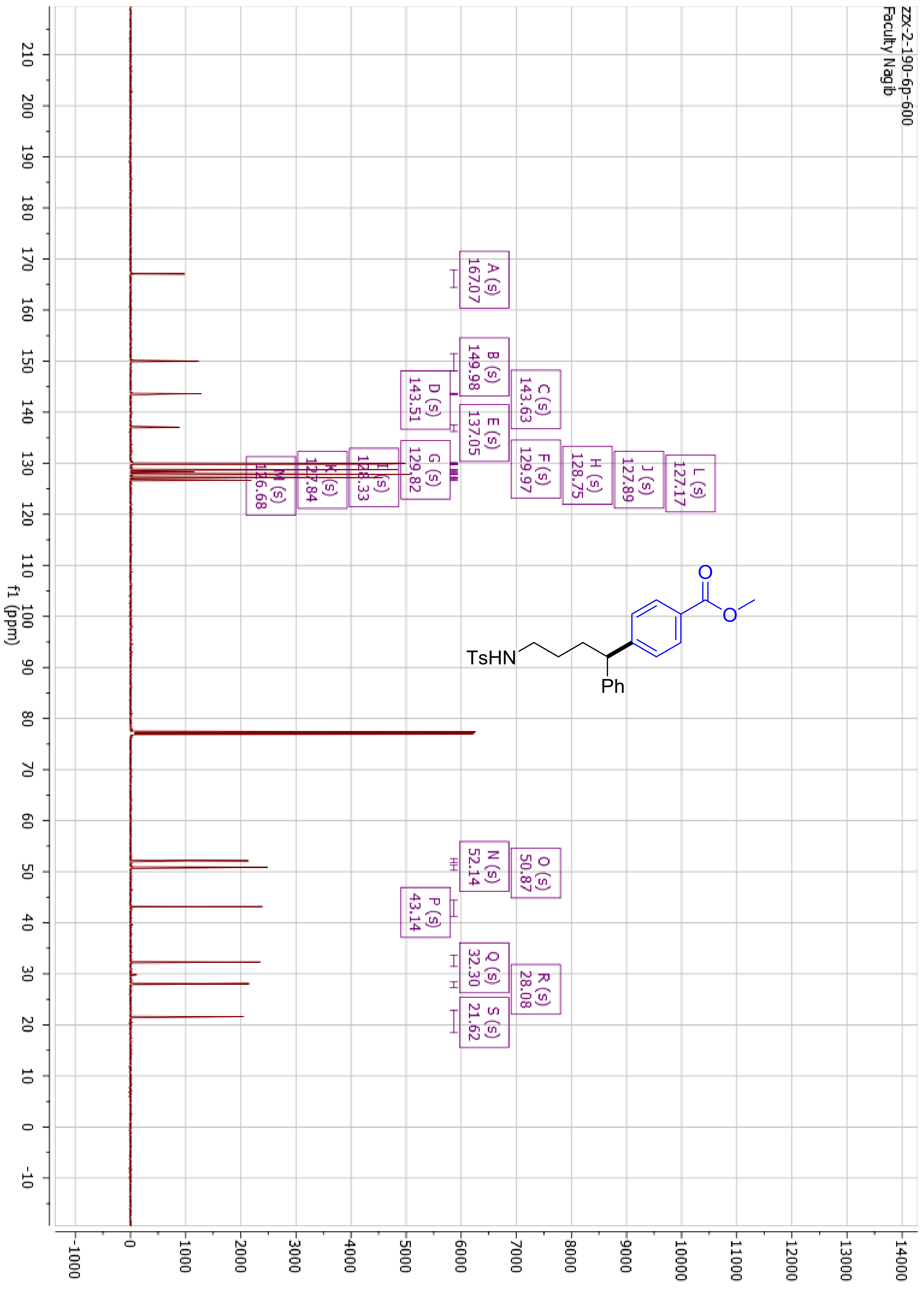

ZZX-2-190-6p-600
Faculty Nagib
A (s) 167.07
B (s) 149.98
C (s) 143.63
D (s) 143.51
E (s) 137.05
F (s) 129.97
G (s) 129.82
H (s) 128.75
I (s) 128.33
J (s) 127.89
K (s) 127.84
L (s) 127.17
M (s) 126.68
N (s) 52.14
O (s) 50.87
P (s) 43.14
Q (s) 32.30
R (s) 28.08
S (s) 21.62
TsHN
Ph
O
O
f1 (ppm)

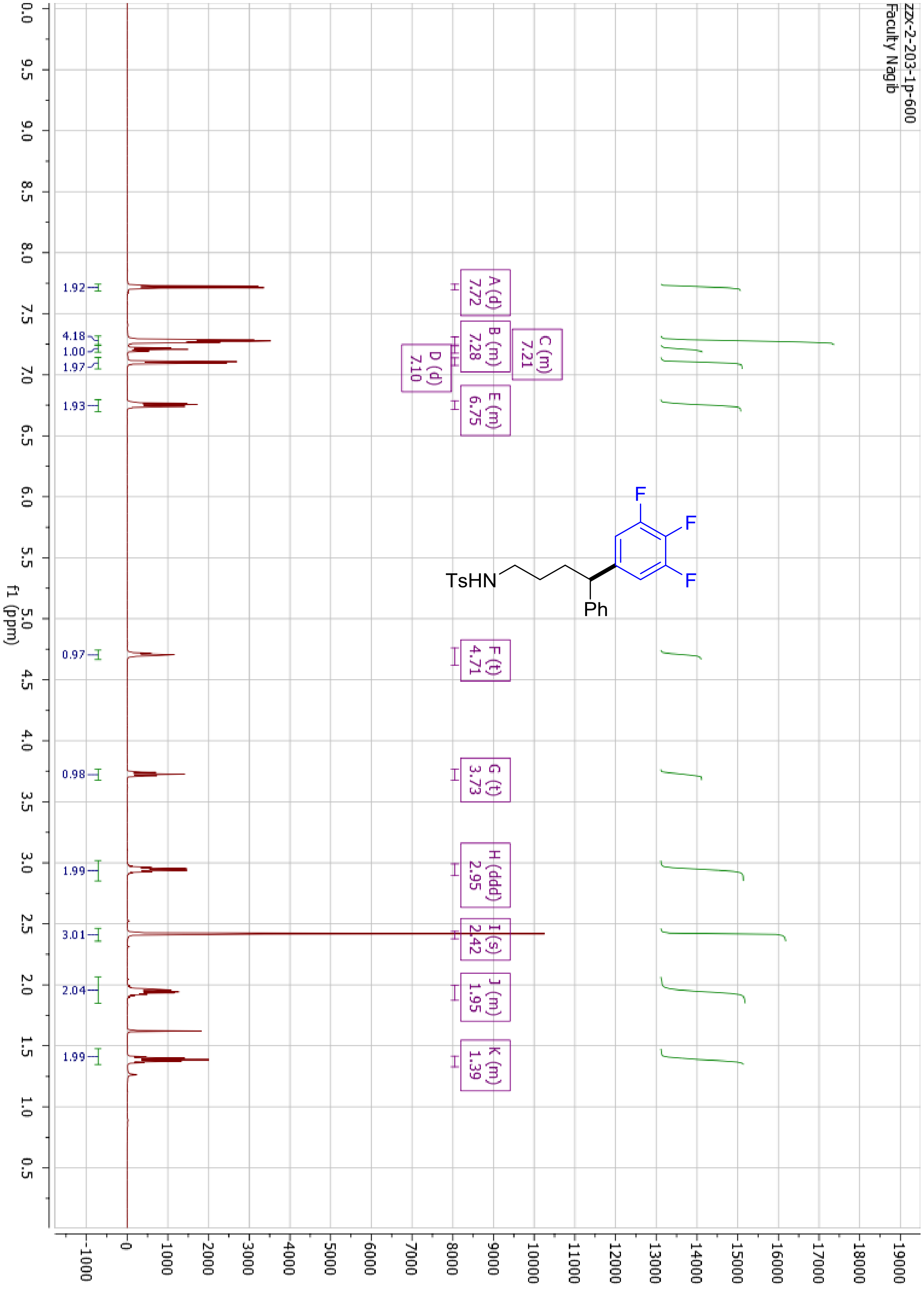
zzx-2-203-1p-600
Faculty Nagib
A (d) 7.72
B (m) 7.28
C (m) 7.21
D (d) 7.10
E (m) 6.75
F (t) 4.71
G (t) 3.73
H (ddd) 2.95
I (s) 2.42
J (m) 1.95
K (m) 1.39
1.92
4.18
1.00
1.97
1.93
0.97
0.98
1.99
3.01
2.04
1.99
TsHN
Ph
F
F
F
f1 (ppm)

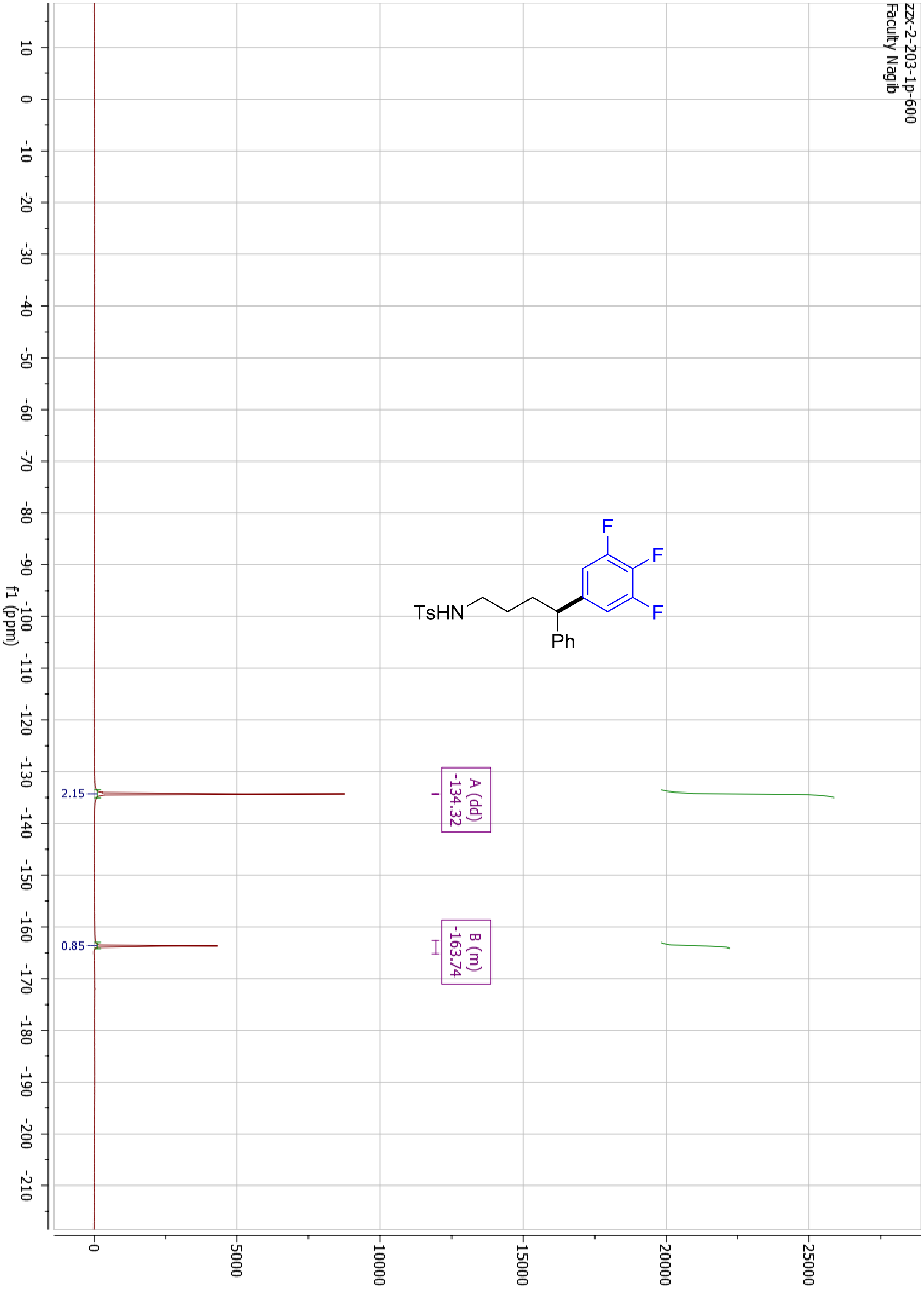
zzx-2-203-1p-600
Faculty Nagib
TsHN
Ph
F
F
F
A (dd)
-134.32
B (m)
-163.74
2.15
0.85
f1 (ppm)

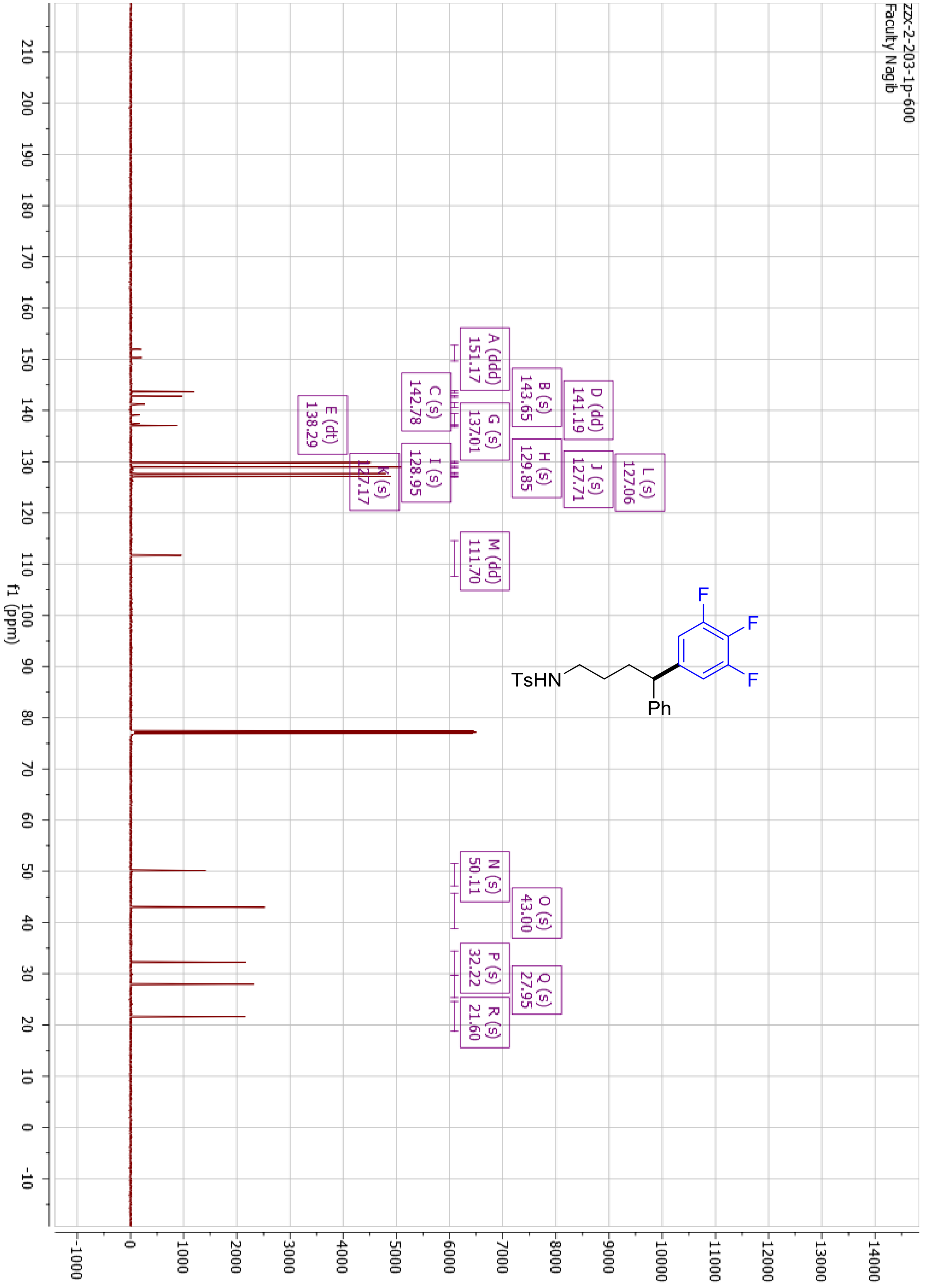
zzx-2-203-1p-600
Facuty Nagib
A (ddd) 151.17
B (s) 143.65
C (s) 142.78
D (dd) 141.19
E (dt) 138.29
G (s) 137.01
H (s) 129.85
I (s) 128.95
J (s) 127.71
K (s) 127.17
L (s) 127.06
M (dd) 111.70
N (s) 50.11
O (s) 43.00
P (s) 32.22
Q (s) 27.95
R (s) 21.60
TsHN
Ph
F
F
F
f1 (ppm)

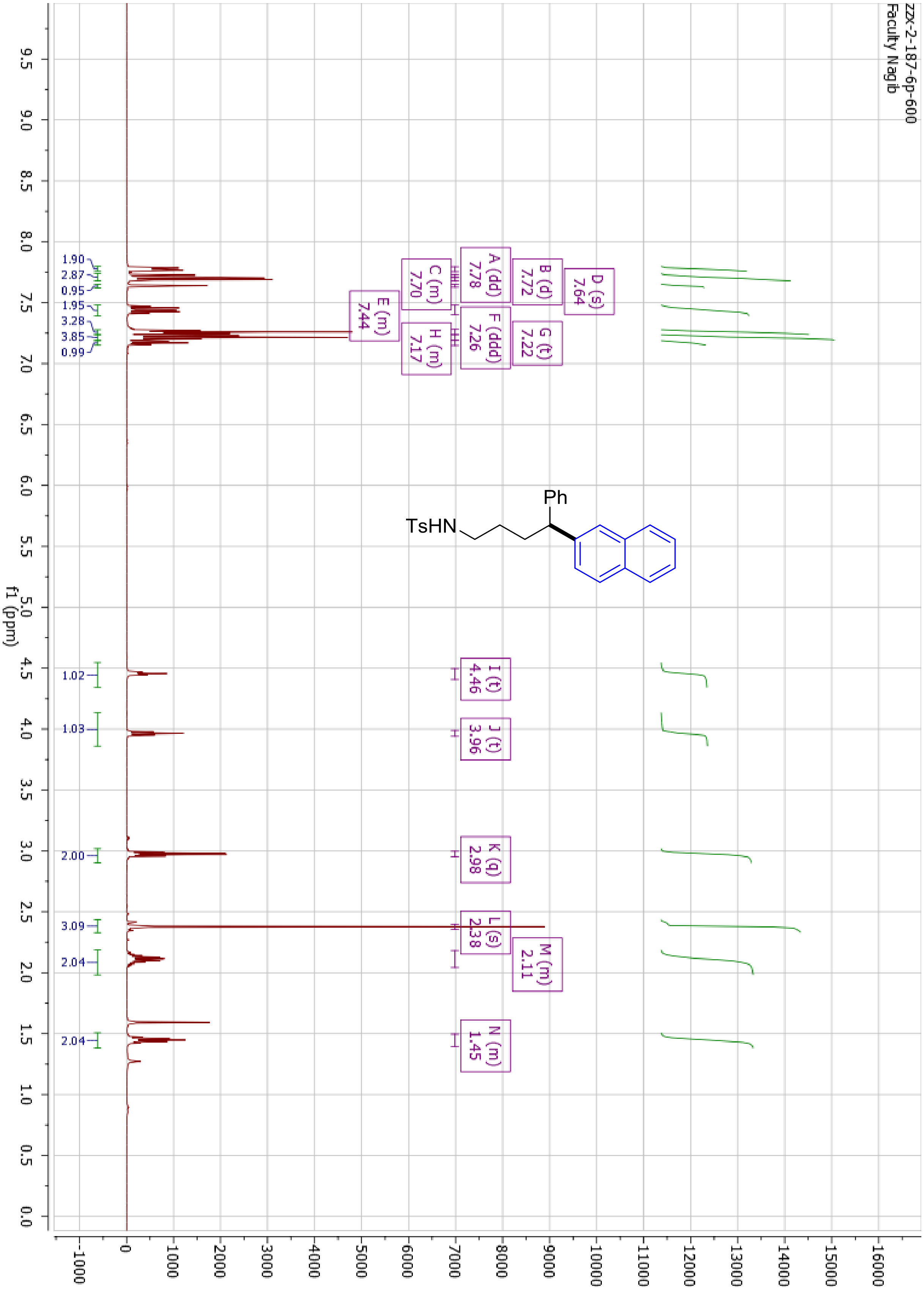
zzx-2-187-6p-600
Faculty Nagib
TsHN
Ph
A (dd) 7.78
B (d) 7.72
C (m) 7.70
D (s) 7.64
E (m) 7.44
F (ddd) 7.26
G (t) 7.22
H (m) 7.17
I (t) 4.46
J (t) 3.96
K (q) 2.98
L (s) 2.38
M (m) 2.11
N (m) 1.45
1.90
2.87
0.95
1.95
3.28
3.85
0.99
1.02
1.03
2.00
3.09
2.04
2.04
f1 (ppm)

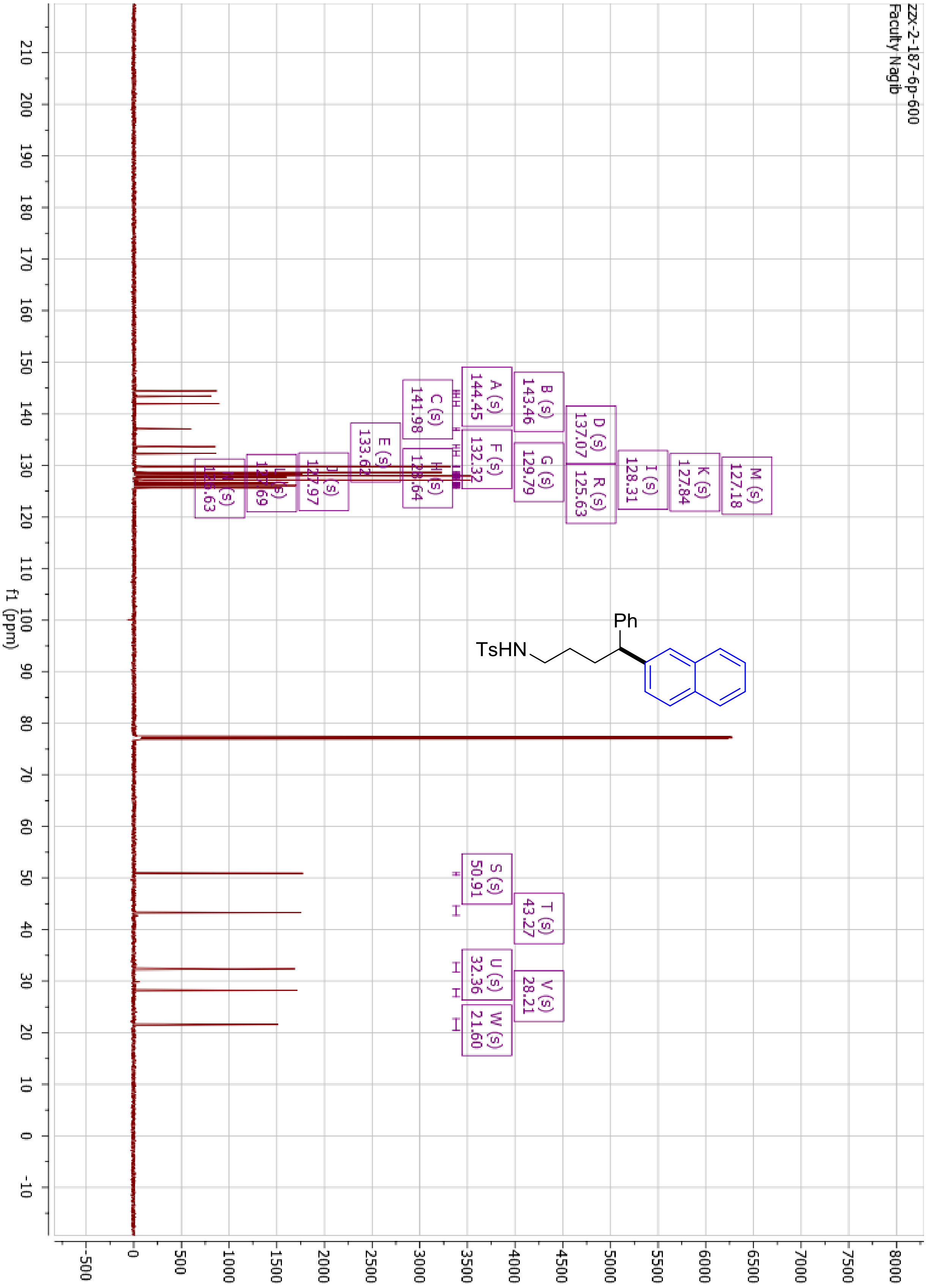
ZZX-2-187-6p-600
Faculty Nagib
A (s) 144.45
B (s) 143.46
C (s) 141.98
D (s) 137.07
E (s) 133.62
F (s) 132.32
G (s) 129.79
H (s) 128.64
I (s) 128.31
J (s) 127.97
K (s) 127.84
M (s) 127.18
R (s) 125.63
S (s) 50.91
T (s) 43.27
U (s) 32.36
V (s) 28.21
W (s) 21.60
TsHN
Ph
f1 (ppm)

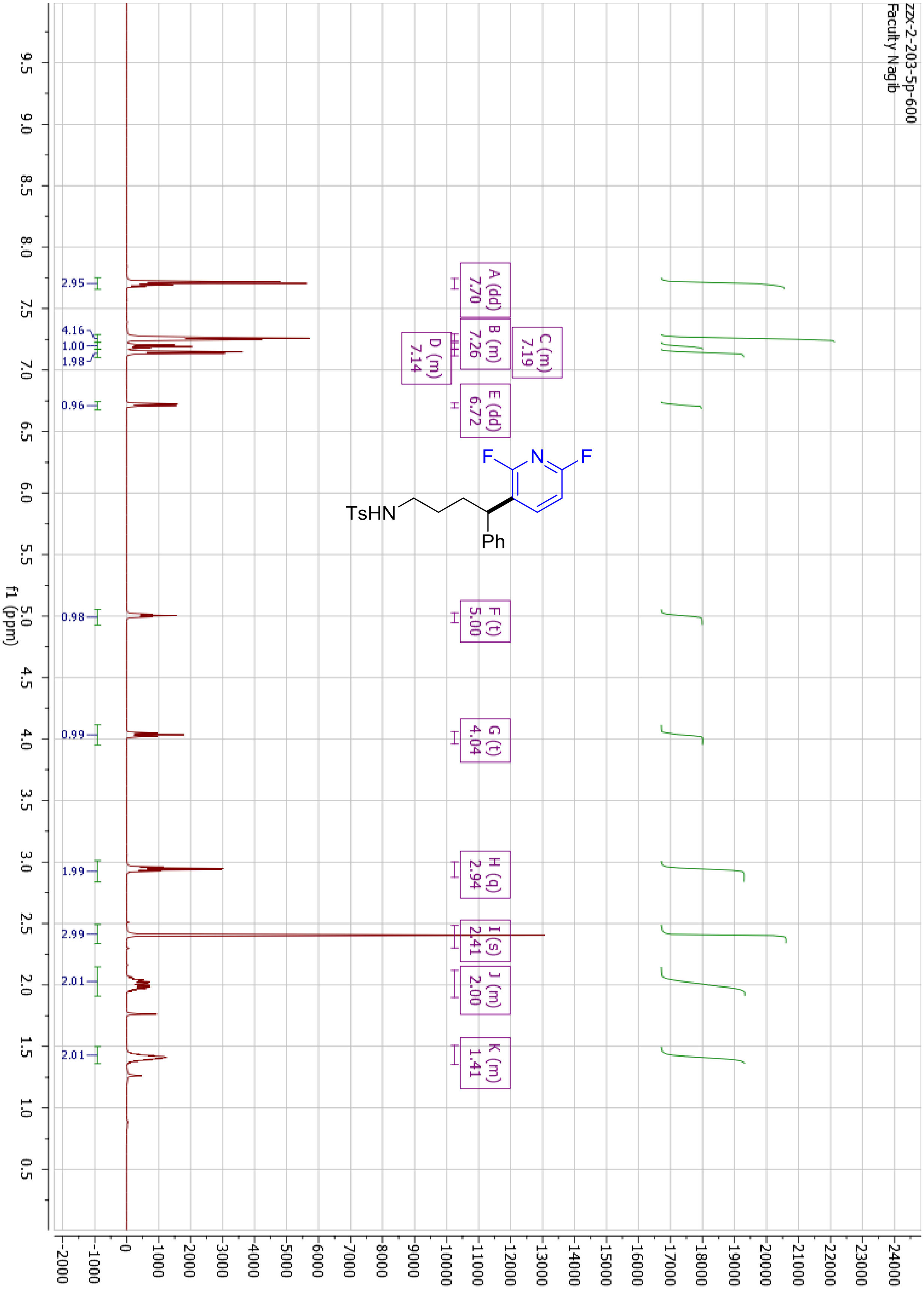
zzx-2-203-5p-600
Faculty Nagib
f1 (ppm)
TsHN
Ph
F
N
F
A (dd) 7.70
B (m) 7.26
C (m) 7.19
D (m) 7.14
E (dd) 6.72
F (t) 5.00
G (t) 4.04
H (q) 2.94
I (s) 2.41
J (m) 2.00
K (m) 1.41

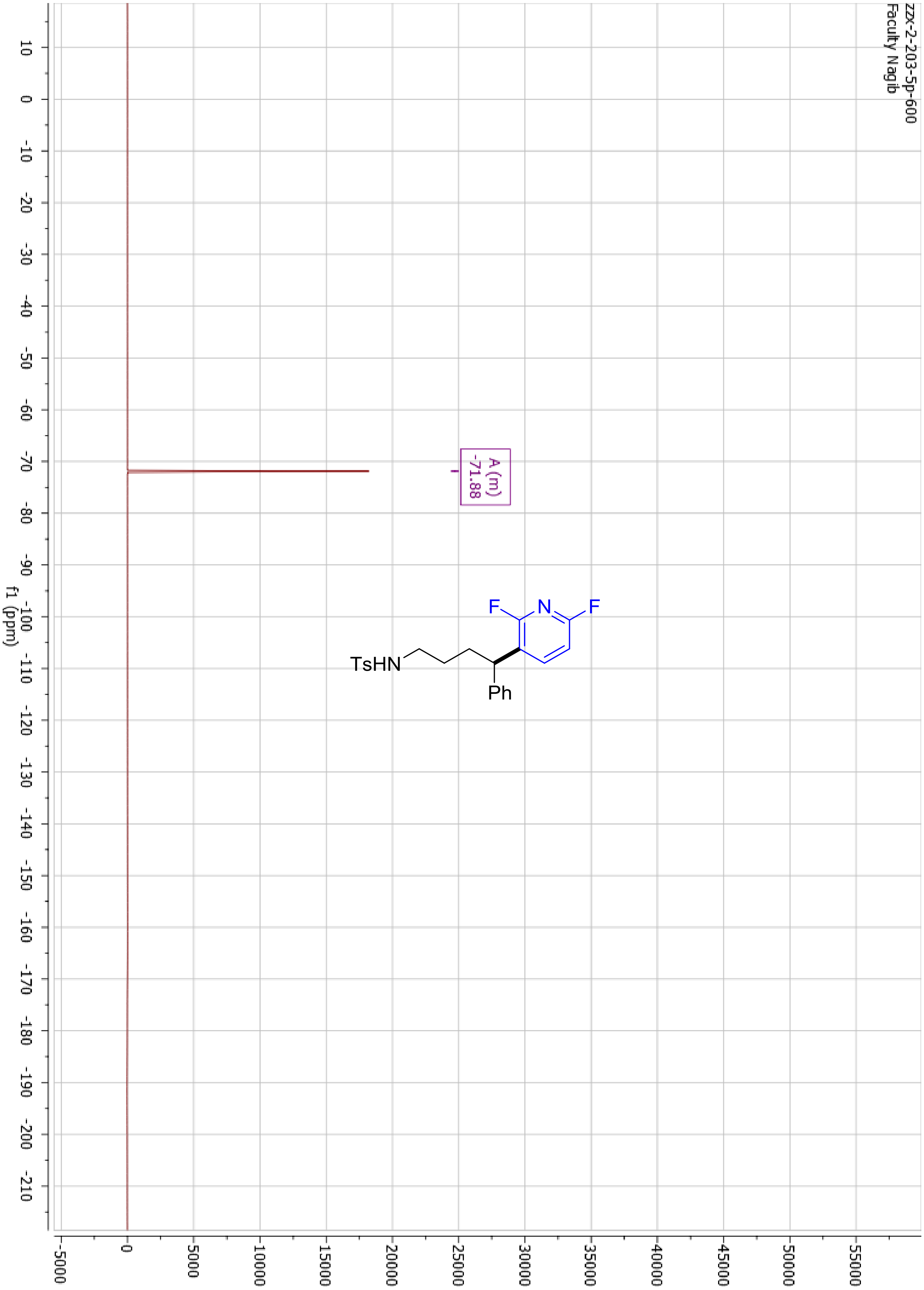
zzx-2-203-5p-600
Faculty Nagib
A (m)
-71.88
f1 (ppm)
F
N
F
TsHN
Ph

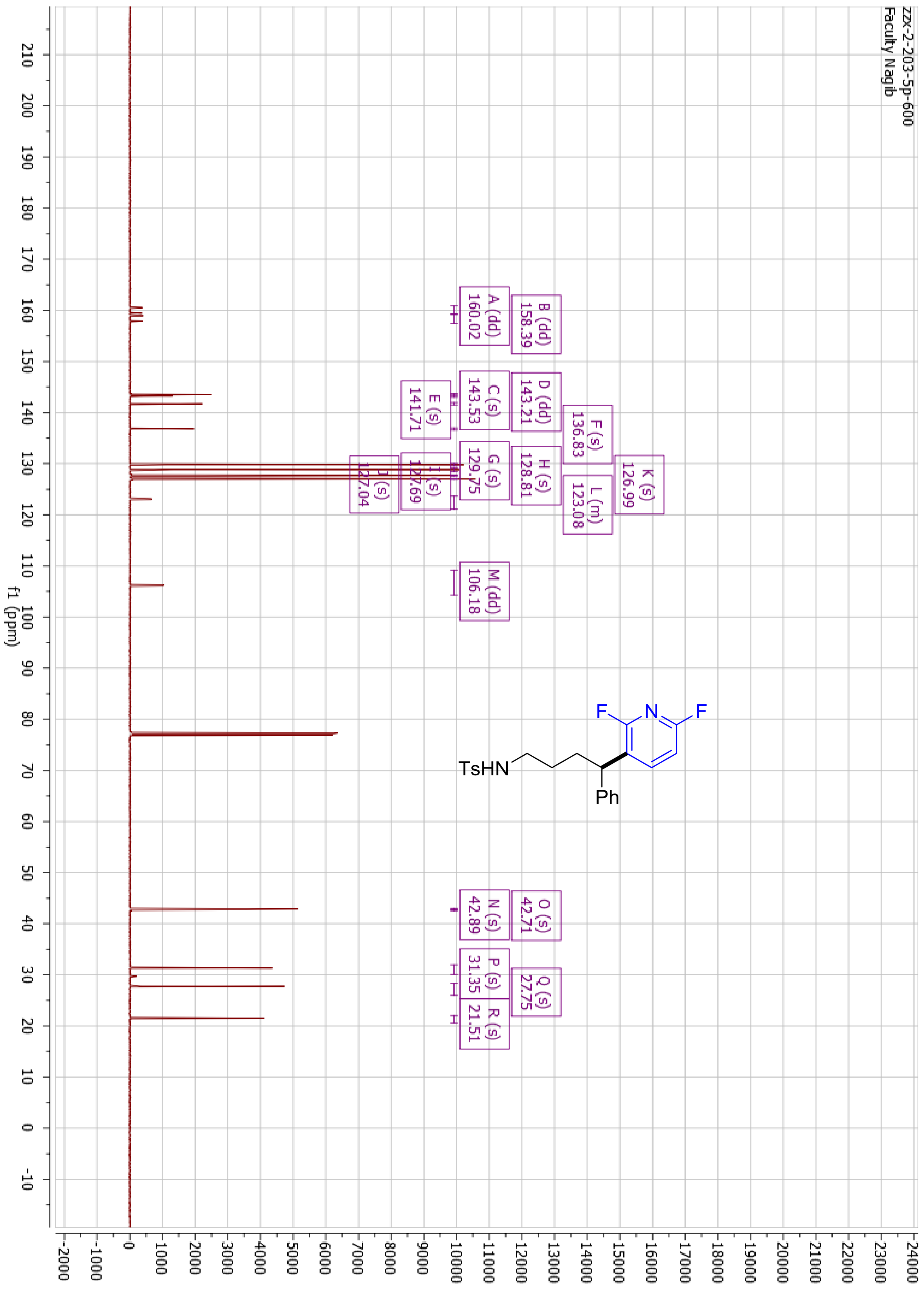

zzx-2-203-5p-600
Faculty Nagib
A (dd) 160.02
B (dd) 158.39
C (s) 143.53
D (dd) 143.21
E (s) 141.71
F (s) 136.83
G (s) 129.75
H (s) 128.81
I (s) 127.69
J (s) 127.04
K (s) 126.99
L (m) 123.08
M (dd) 106.18
N (s) 42.89
O (s) 42.71
P (s) 31.35
Q (s) 27.75
R (s) 21.51
TsHN
Ph
F
N
F
f1 (ppm)

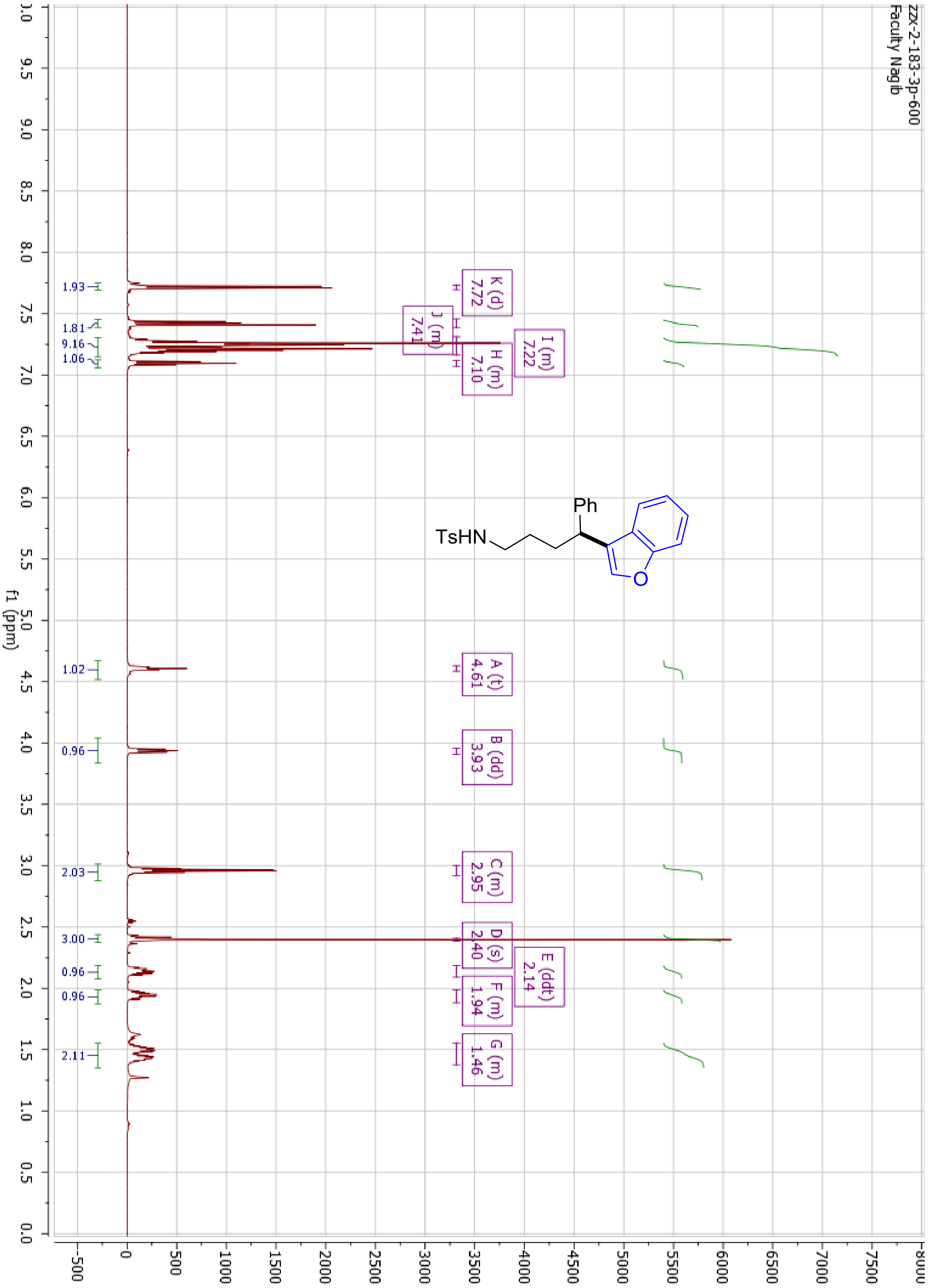
zzx-2-183-3p-600
Faculty Nagib
TsHN
Ph
O
K (d) 7.72
J (m) 7.41
I (m) 7.22
H (m) 7.10
A (t) 4.61
B (dd) 3.93
C (m) 2.95
D (s) 2.40
E (ddt) 2.14
F (m) 1.94
G (m) 1.46
f1 (ppm)

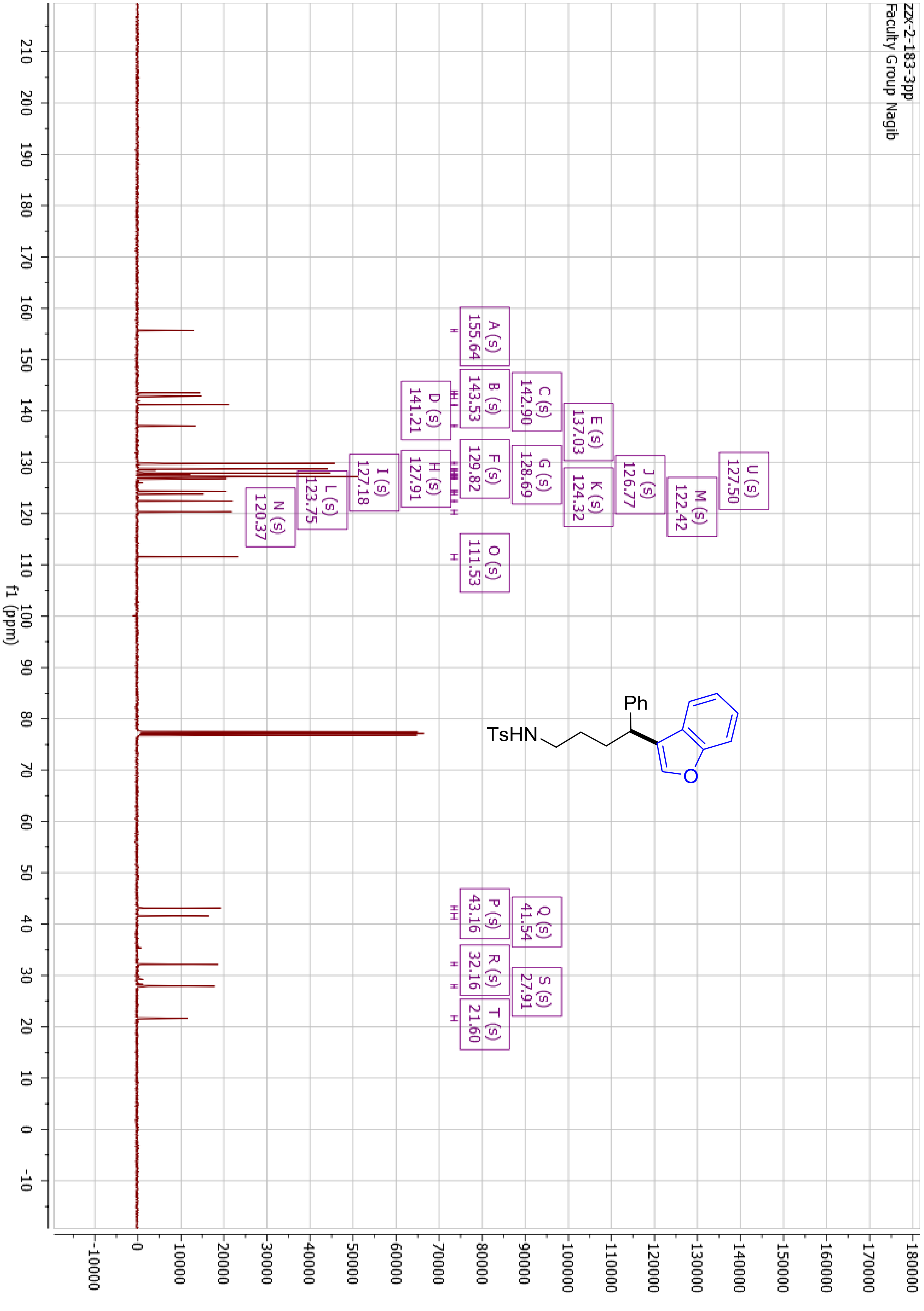
zzx-2-183-3pp
Faculty Group Nagib
A (s) 155.64
B (s) 143.53
C (s) 142.90
D (s) 141.21
E (s) 137.03
F (s) 129.82
G (s) 128.69
H (s) 127.91
I (s) 127.18
J (s) 126.77
K (s) 124.32
L (s) 123.75
M (s) 122.42
N (s) 120.37
O (s) 111.53
P (s) 43.16
Q (s) 41.54
R (s) 32.16
S (s) 27.91
T (s) 21.60
U (s) 127.50
TsHN
Ph
O
f1 (ppm)

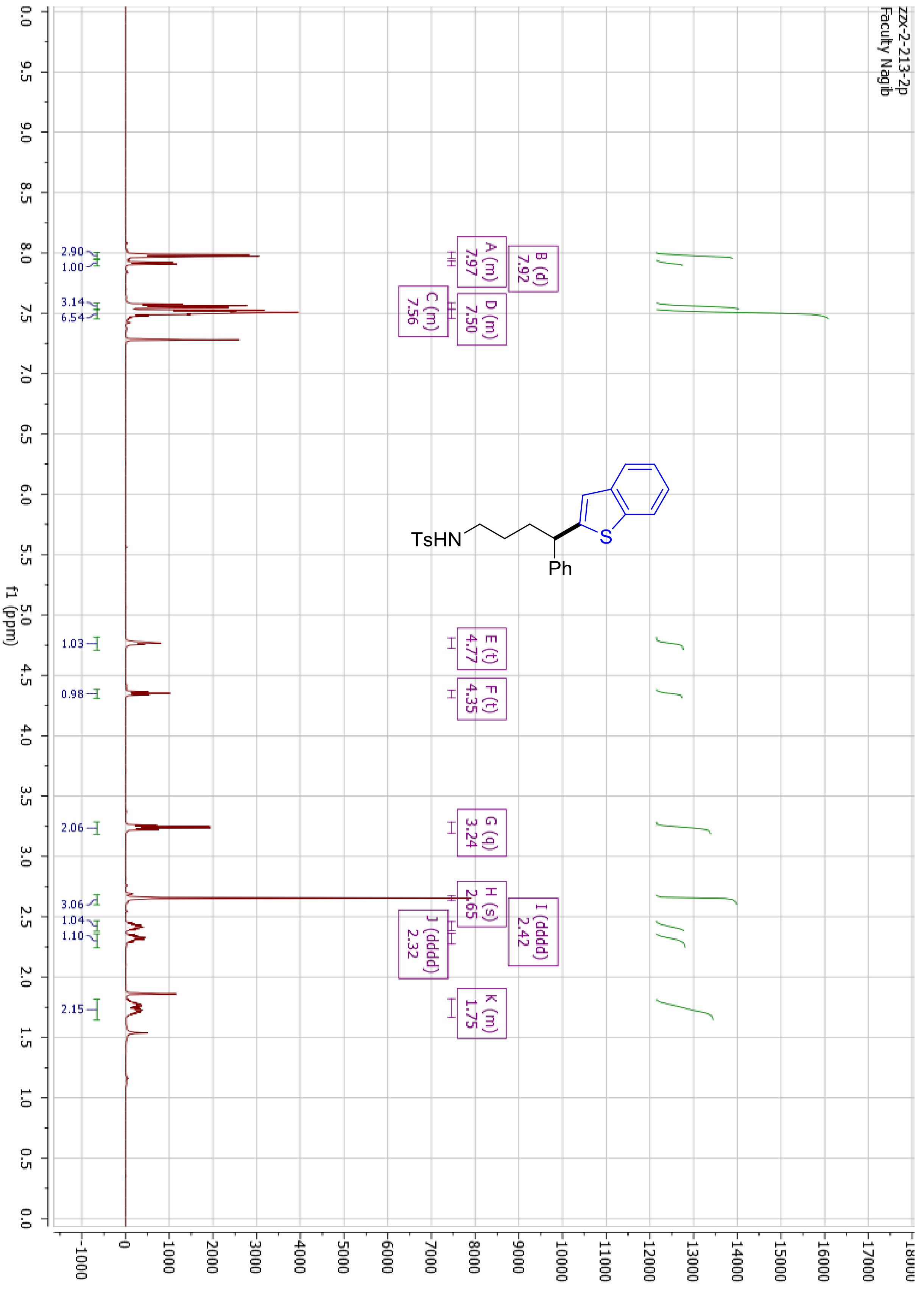
zzx-2-213-2p
Faculty Nagib
TsHN
S
Ph
A (m) 7.97
B (d) 7.92
C (m) 7.56
D (m) 7.50
E (t) 4.77
F (t) 4.35
G (q) 3.24
H (s) 2.65
I (dddd) 2.42
J (dddd) 2.32
K (m) 1.75
2.90
1.00
3.14
6.54
1.03
0.98
2.06
3.06
1.04
1.10
2.15
f1 (ppm)

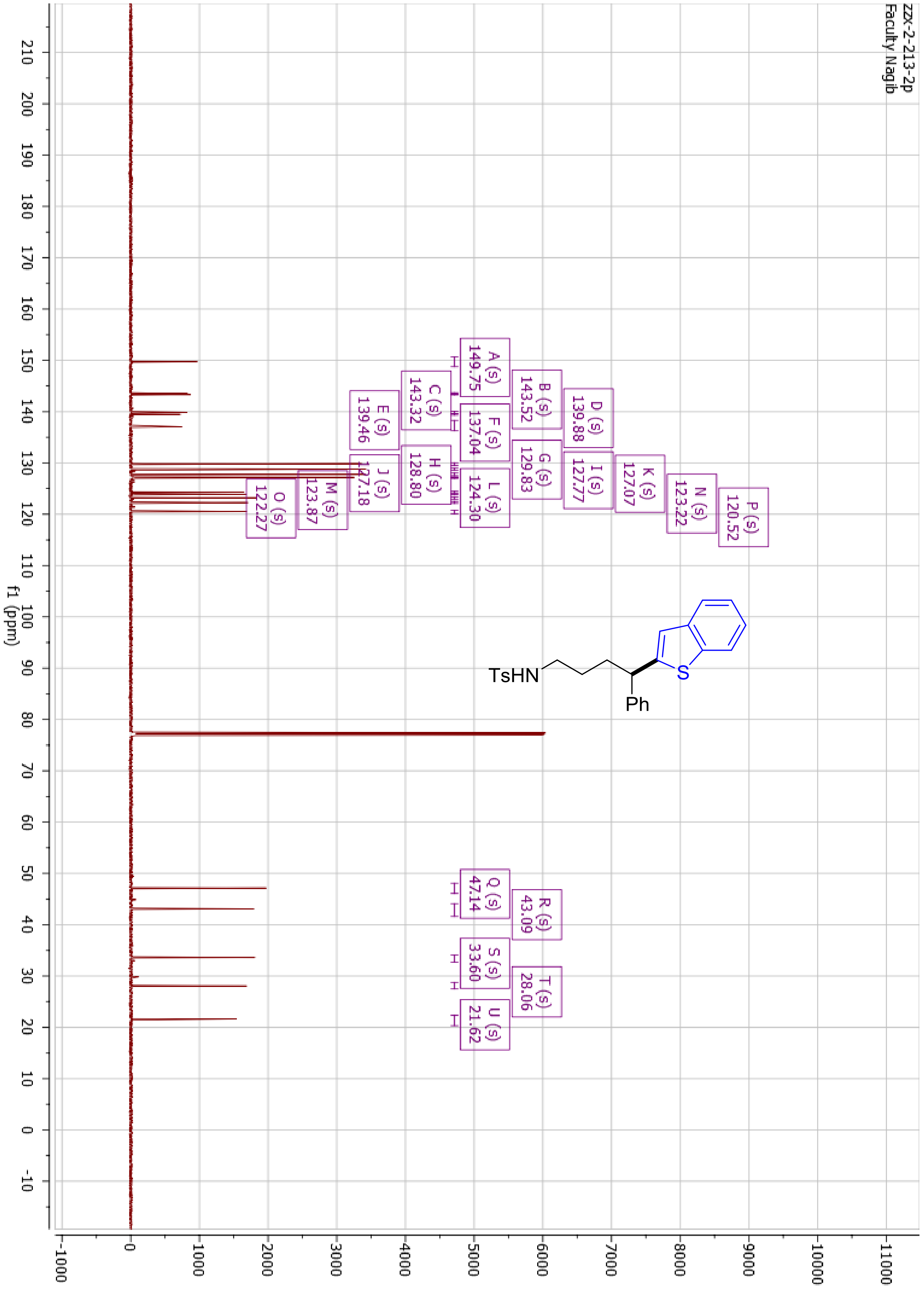

zzx-2-213-2p
Faculty Nagib
A (s) 149.75
B (s) 143.52
C (s) 143.32
D (s) 139.88
E (s) 139.46
F (s) 137.04
G (s) 129.83
H (s) 128.80
I (s) 127.77
J (s) 127.18
K (s) 127.07
L (s) 124.30
M (s) 123.87
N (s) 123.22
O (s) 122.27
P (s) 120.52
Q (s) 47.14
R (s) 43.09
S (s) 33.60
T (s) 28.06
U (s) 21.62
TsHN
Ph
S
f1 (ppm)

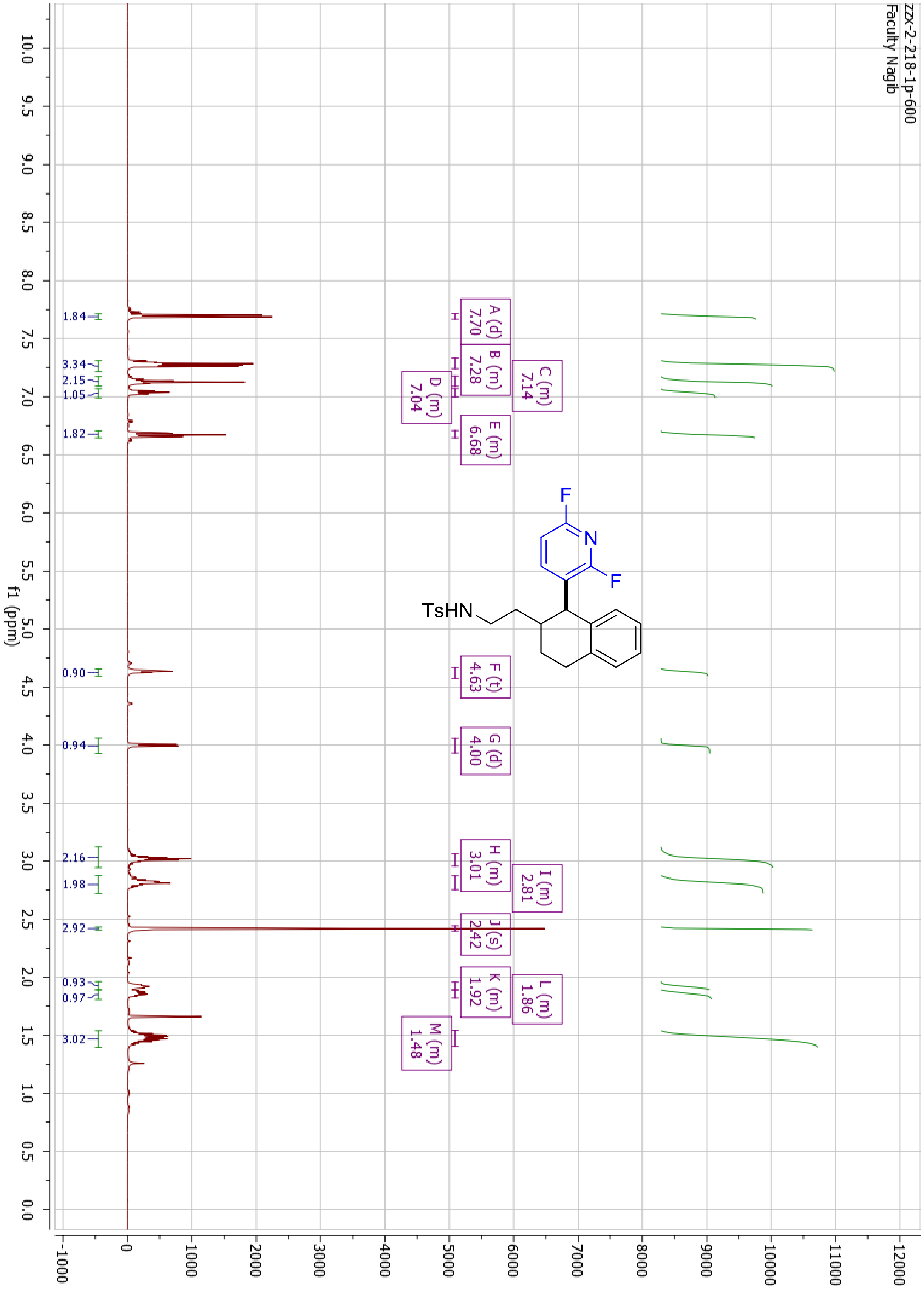
ZZX-2-218-1p-600
Faculty Nagib
TsHN
N
F
F
A (d) 7.70
B (m) 7.28
C (m) 7.14
D (m) 7.04
E (m) 6.68
F (t) 4.63
G (d) 4.00
H (m) 3.01
I (m) 2.81
J (s) 2.42
K (m) 1.92
L (m) 1.86
M (m) 1.48
1.84
3.34
2.15
1.05
1.82
0.90
0.94
2.16
1.98
2.92
0.93
0.97
3.02
f1 (ppm)

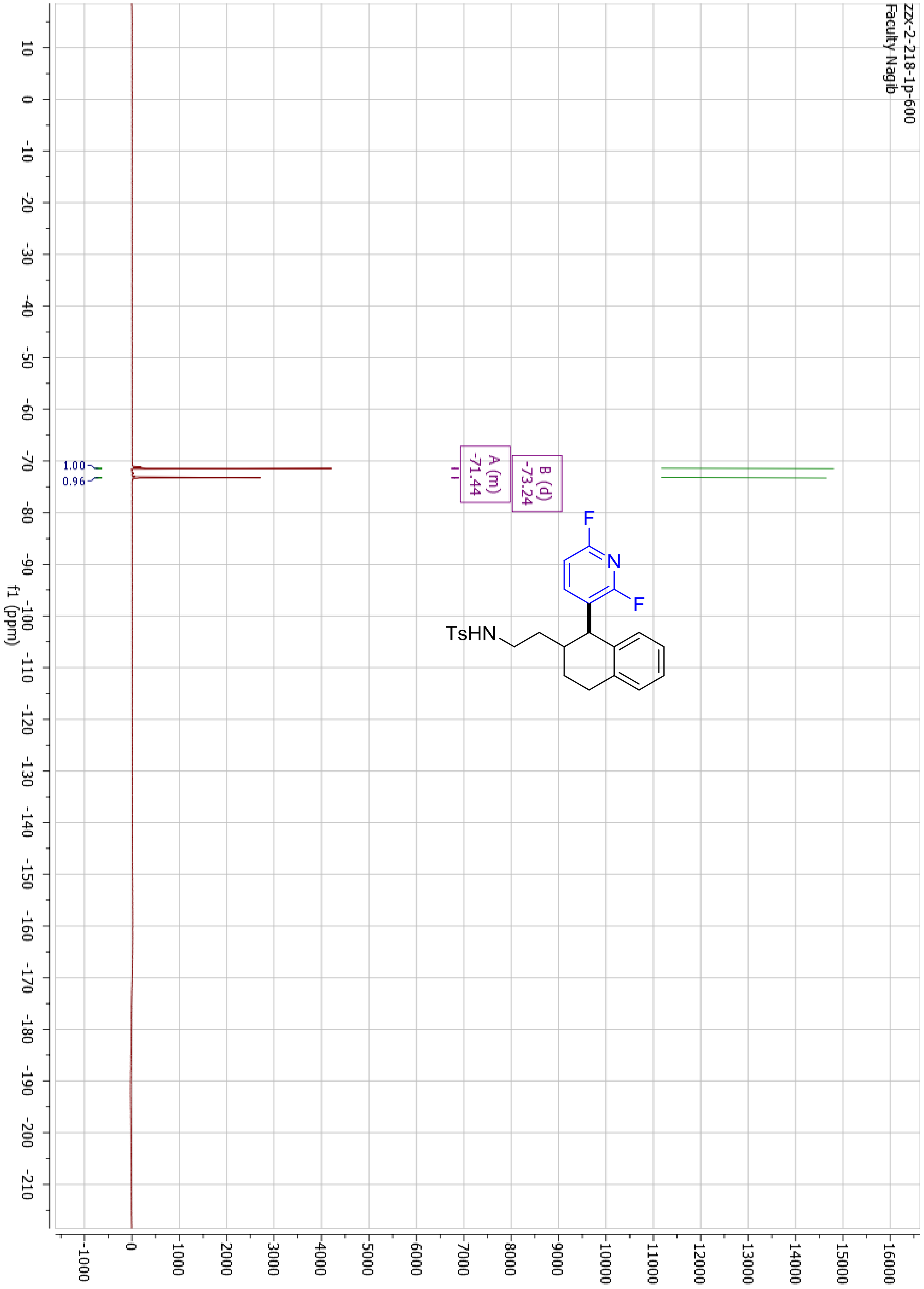

ZZX-2-218-1p-600
Faculty Nagib
A (m)
-71.44
B (d)
-73.24
1.00
0.96
f1 (ppm)
TsHN
F
N
F

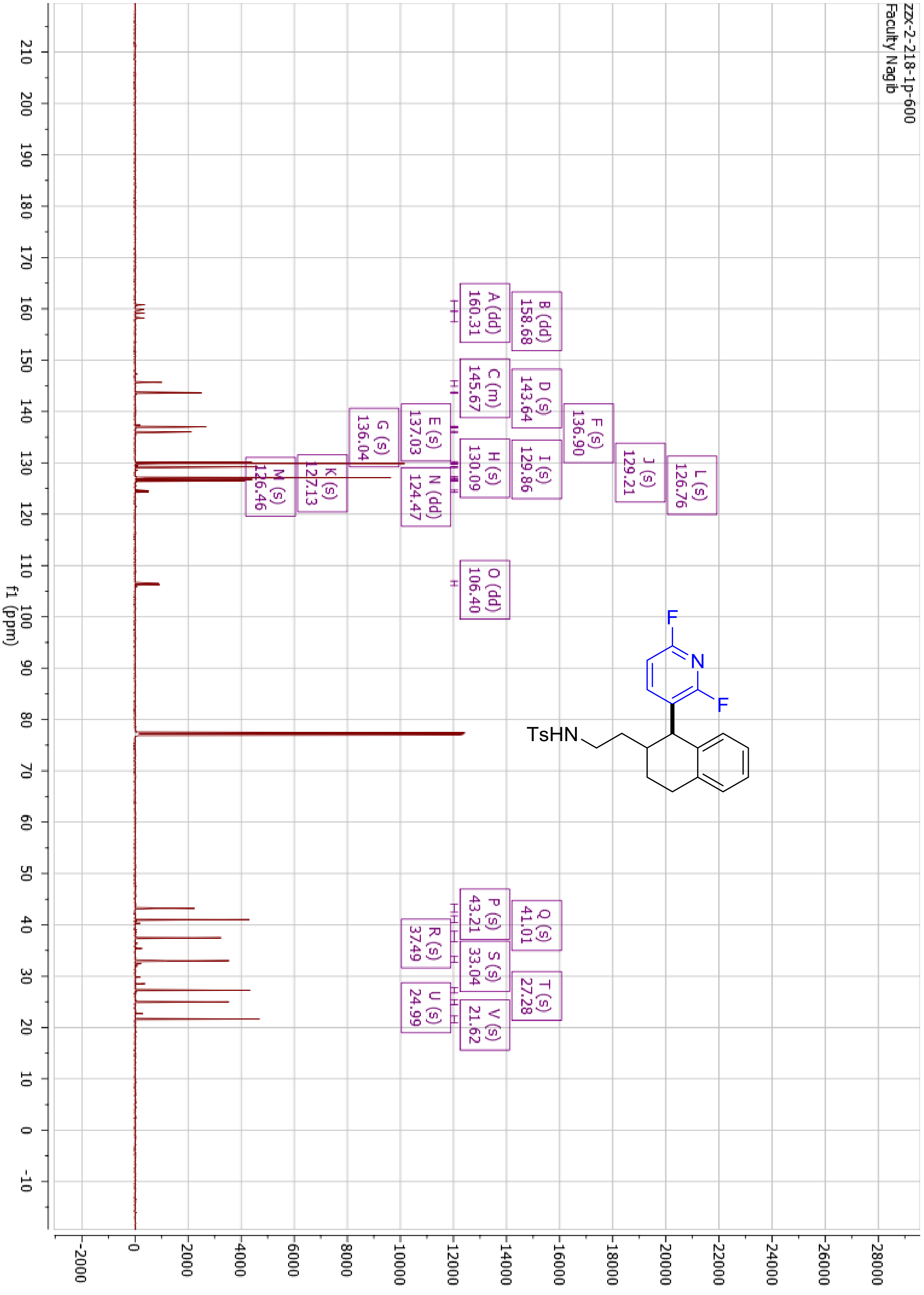
zzx-2-218-1p-600
Faculty Nagib
A (dd) 160.31
B (dd) 158.68
C (m) 145.67
D (s) 143.64
E (s) 137.03
F (s) 136.90
G (s) 136.04
H (s) 130.09
I (s) 129.86
J (s) 129.21
K (s) 127.13
L (s) 126.76
M (s) 126.46
N (dd) 124.47
O (dd) 106.40
P (s) 43.21
Q (s) 41.01
R (s) 37.49
S (s) 33.04
T (s) 27.28
U (s) 24.99
V (s) 21.62
TsHN
N
F
F
f1 (ppm)

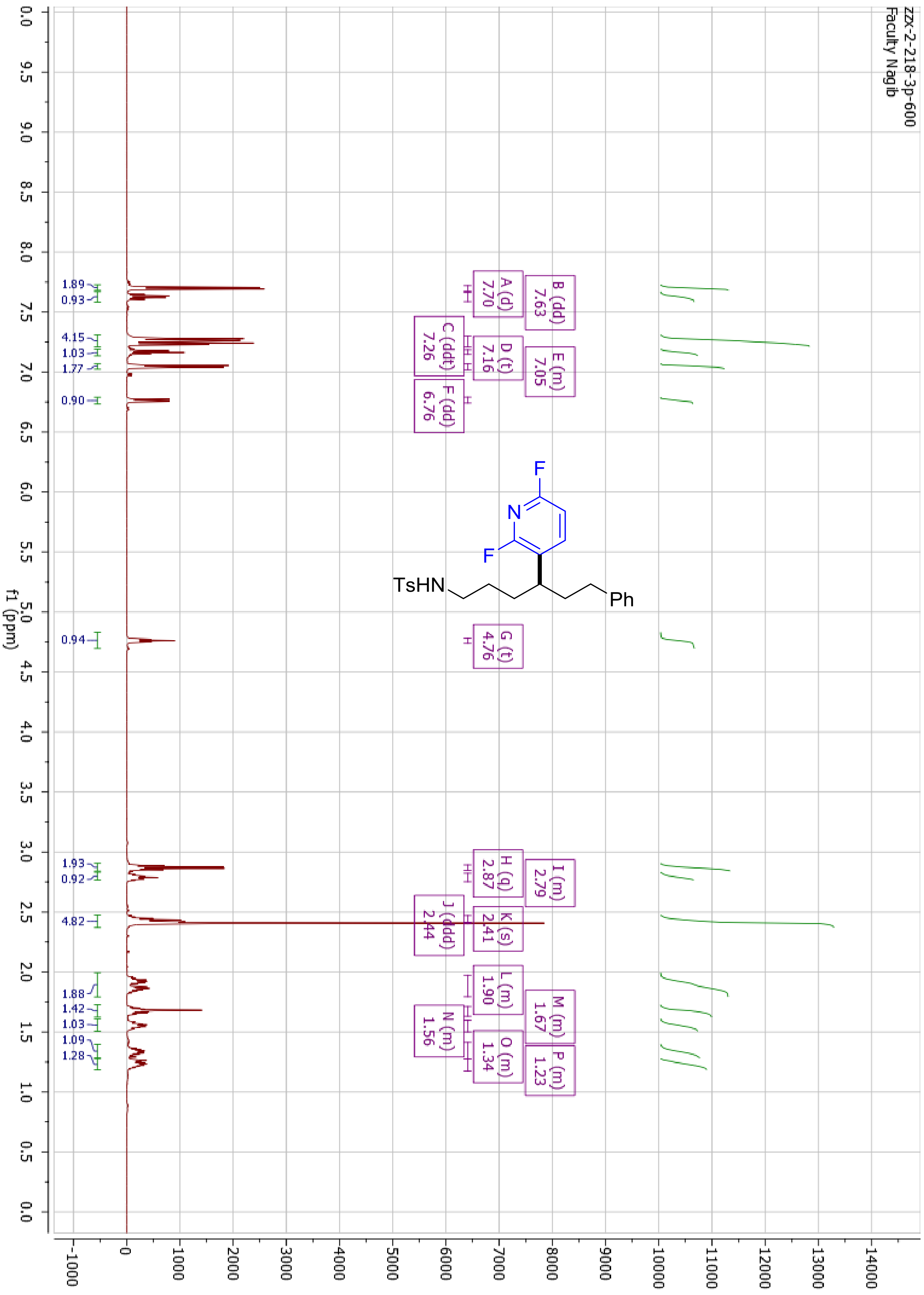
zzx-2-218-3p-600
Faculty Nagib
A (d) 7.70
B (dd) 7.63
C (ddt) 7.26
D (t) 7.16
E (m) 7.05
F (dd) 6.76
G (t) 4.76
H (q) 2.87
I (m) 2.79
J (ddd) 2.44
K (s) 2.41
L (m) 1.90
M (m) 1.67
N (m) 1.56
O (m) 1.34
P (m) 1.23
TsHN
F
N
F
Ph
f1 (ppm)

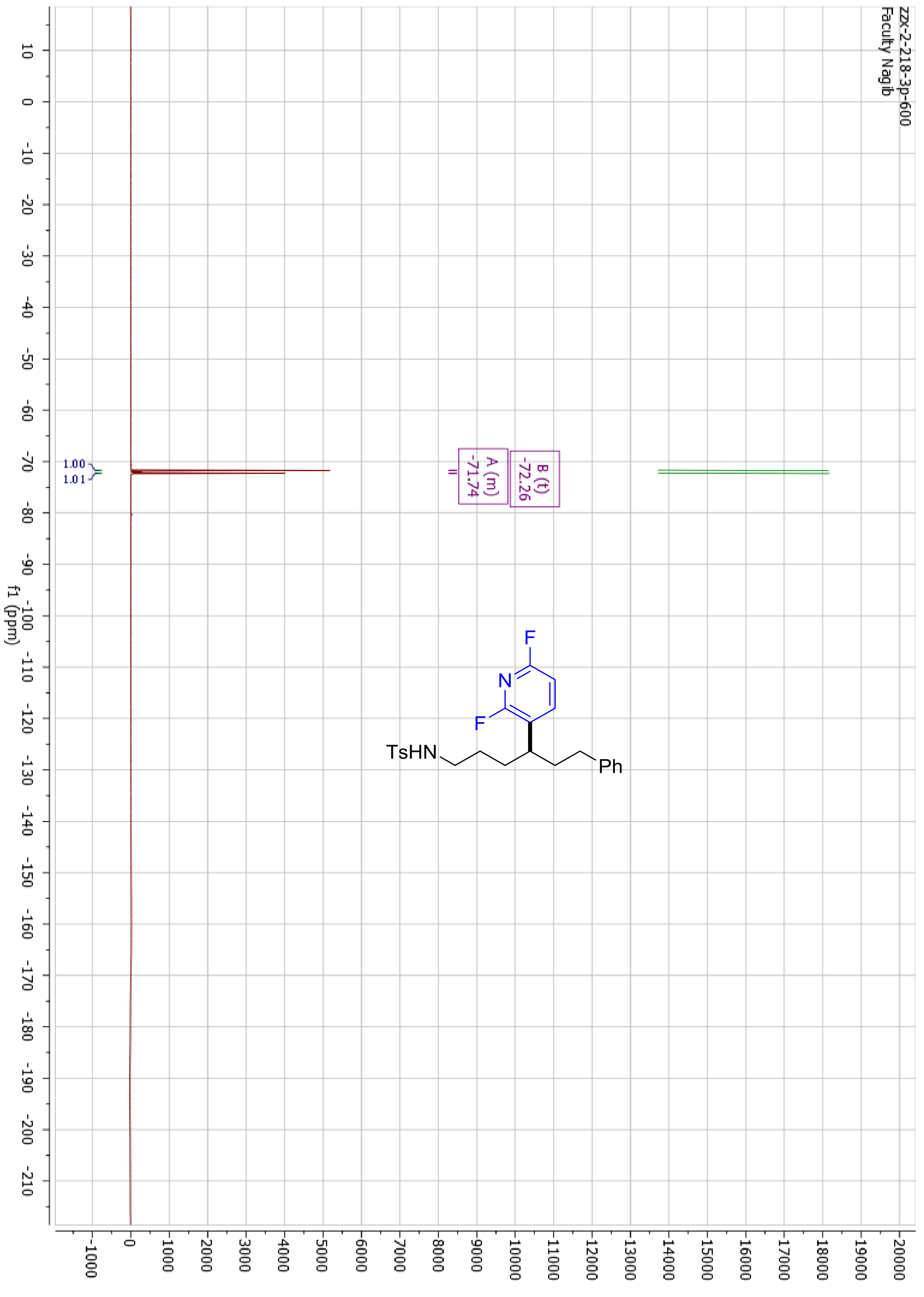

zzx-2-218-3p-600
Faculty Nagib
A (m)
-71.74
B (t)
-72.26
1.00
1.01
TsHN
Ph
F
N
F
f1 (ppm)

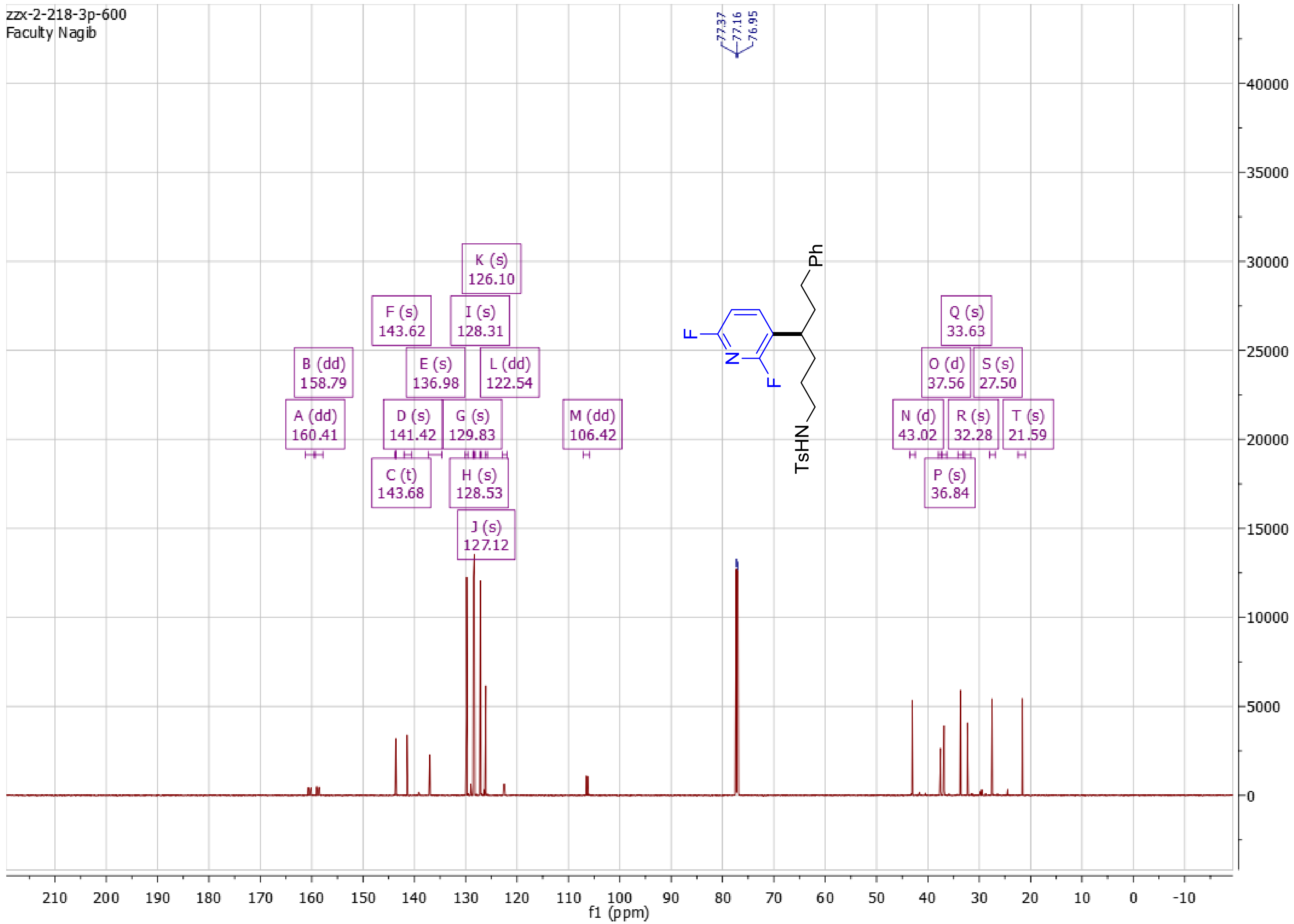

zzx-2-218-3p-600
Faculty Nagib
77.37
77.16
76.95
A (dd) 160.41
B (dd) 158.79
C (t) 143.68
D (s) 141.42
E (s) 136.98
F (s) 143.62
G (s) 129.83
H (s) 128.53
I (s) 128.31
J (s) 127.12
K (s) 126.10
L (dd) 122.54
M (dd) 106.42
N (d) 43.02
O (d) 37.56
P (s) 36.84
Q (s) 33.63
R (s) 32.28
S (s) 27.50
T (s) 21.59
TsHN
Ph
N
F
F
f1 (ppm)

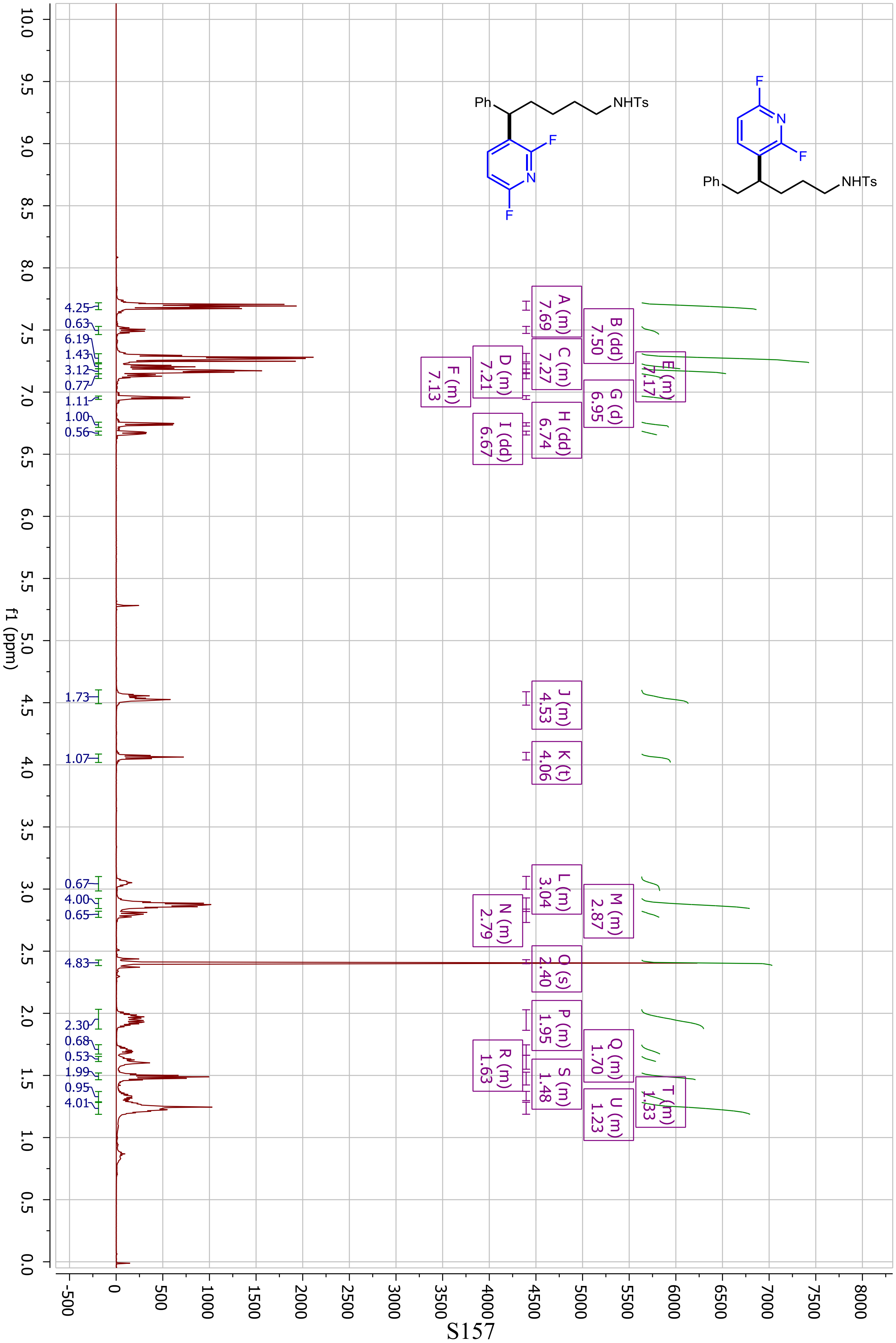

Ph
NHTs
F
N
f1 (ppm)
A (m) 7.69
B (dd) 7.50
C (m) 7.27
D (m) 7.21
E (m) 7.17
F (m) 7.13
G (d) 6.95
H (dd) 6.74
I (dd) 6.67
J (m) 4.53
K (t) 4.06
L (m) 3.04
M (m) 2.87
N (m) 2.79
O (s) 2.40
P (m) 1.95
Q (m) 1.70
R (m) 1.63
S (m) 1.48
T (m) 1.33
U (m) 1.23

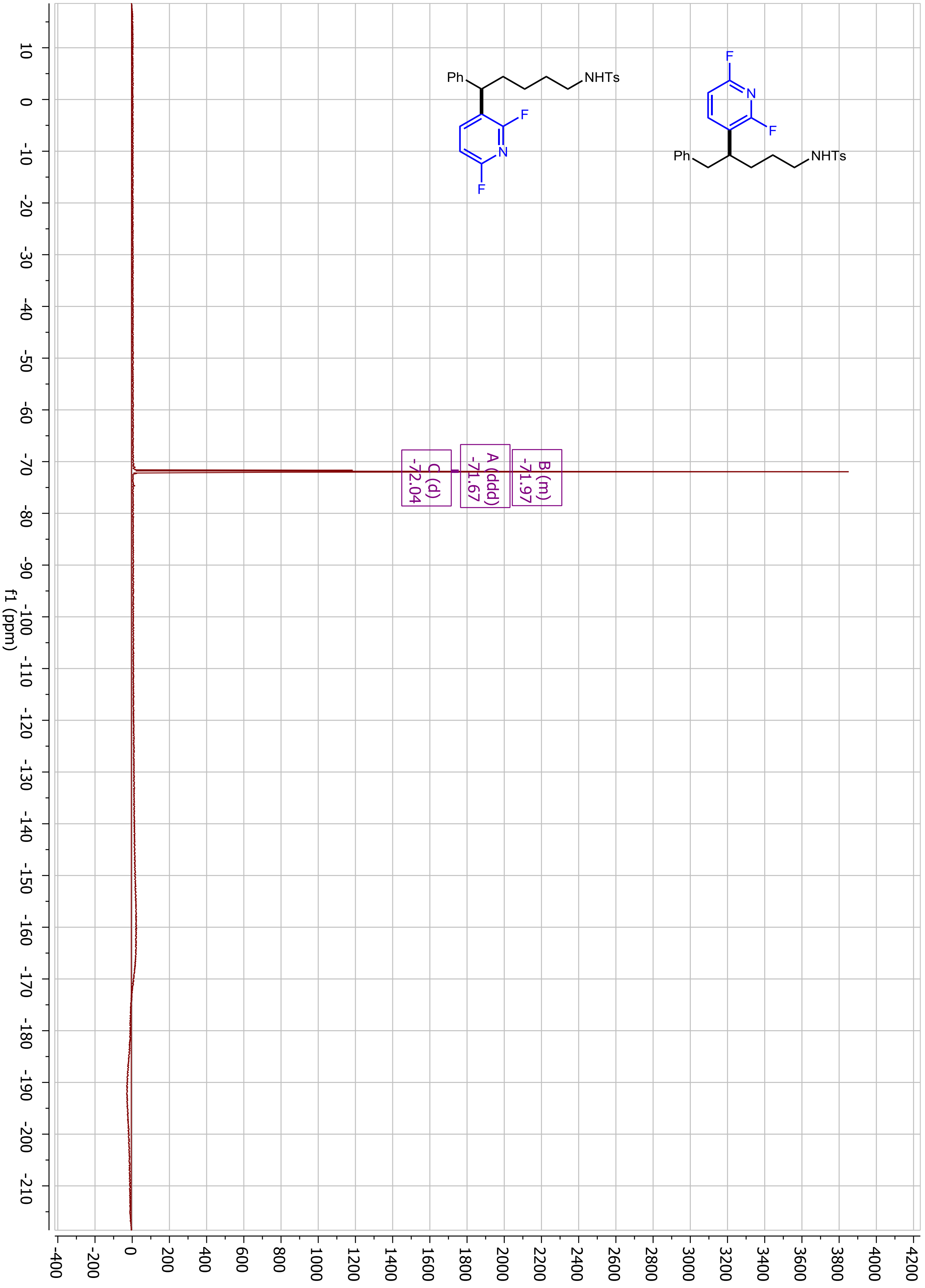
Ph
NHTs
F
N
F
Ph
NHTs
F
N
F
A (ddd)
-71.67
B (m)
-71.97
C (d)
-72.04
f1 (ppm)

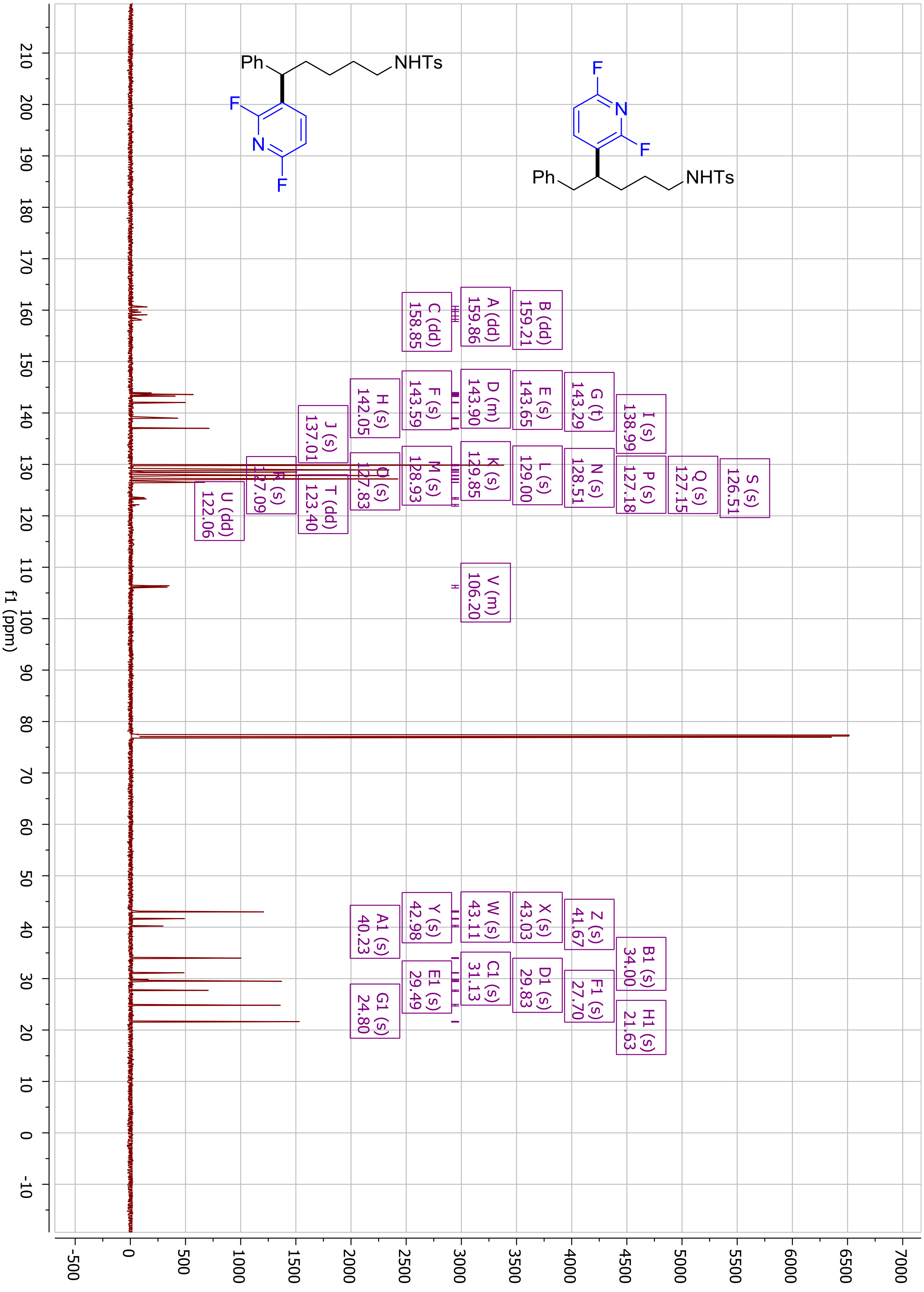
Ph
NHTs
F
N
F
Ph
NHTs
F
N
F
A (dd) 159.86
B (dd) 159.21
C (dd) 158.85
D (m) 143.90
E (s) 143.65
F (s) 143.59
G (t) 143.29
H (s) 142.05
I (s) 138.99
J (s) 137.01
K (s) 129.85
L (s) 129.00
M (s) 128.93
N (s) 128.51
O (s) 127.83
P (s) 127.18
Q (s) 127.15
R (s) 127.09
S (s) 126.51
T (dd) 123.40
U (dd) 122.06
V (m) 106.20
W (s) 43.11
X (s) 43.03
Y (s) 42.98
Z (s) 41.67
A1 (s) 40.23
B1 (s) 34.00
C1 (s) 31.13
D1 (s) 29.83
E1 (s) 29.49
F1 (s) 27.70
G1 (s) 24.80
H1 (s) 21.63
f1 (ppm)

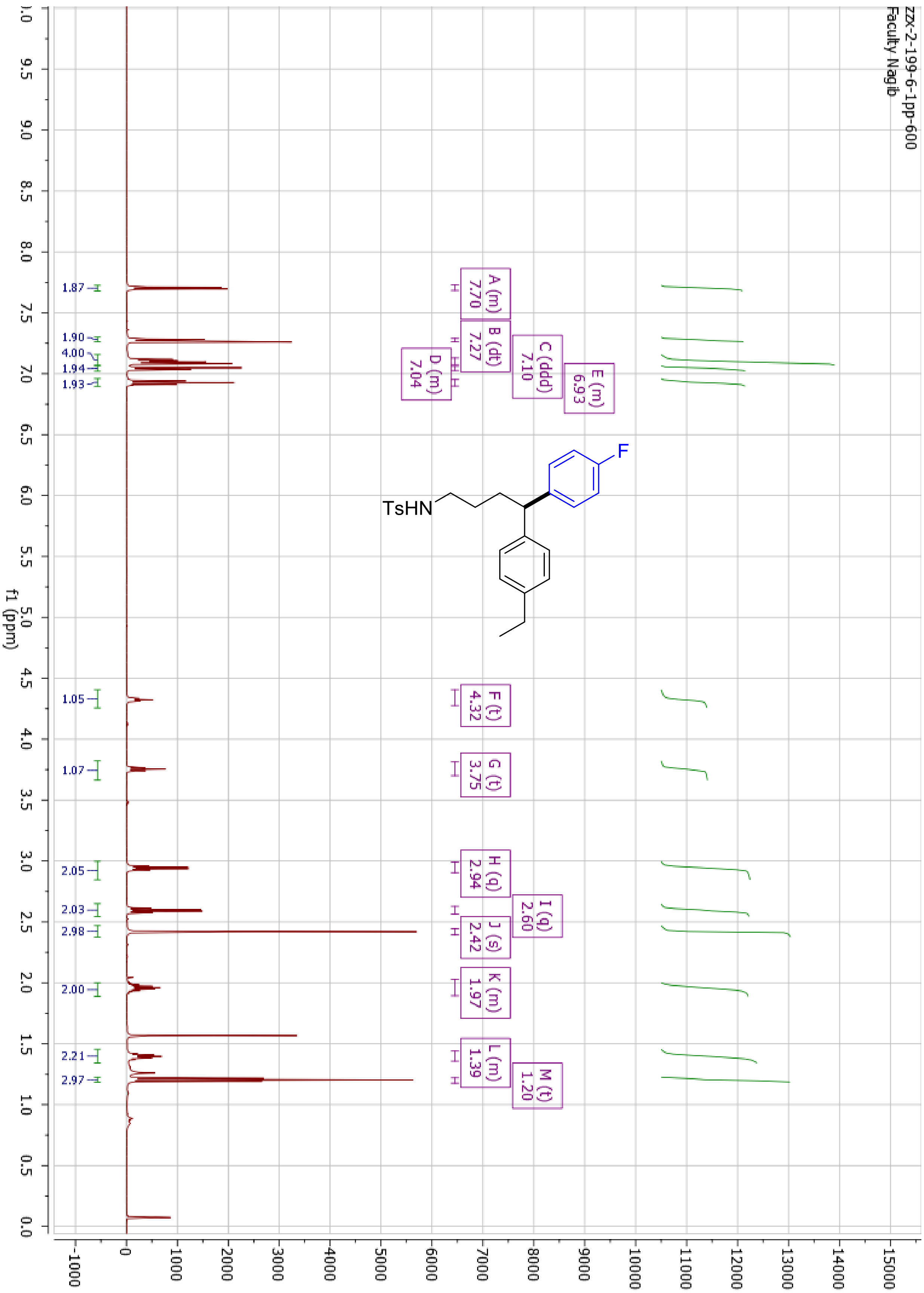
ZZX-2-199-6-1pp-600
Faculty Nagib
A (m) 7.70
B (dt) 7.27
C (ddd) 7.10
D (m) 7.04
E (m) 6.93
F (t) 4.32
G (t) 3.75
H (q) 2.94
I (q) 2.60
J (s) 2.42
K (m) 1.97
L (m) 1.39
M (t) 1.20
1.87
1.90
4.00
1.94
1.93
1.05
1.07
2.05
2.03
2.98
2.00
2.21
2.97
TsHN
F
f1 (ppm)

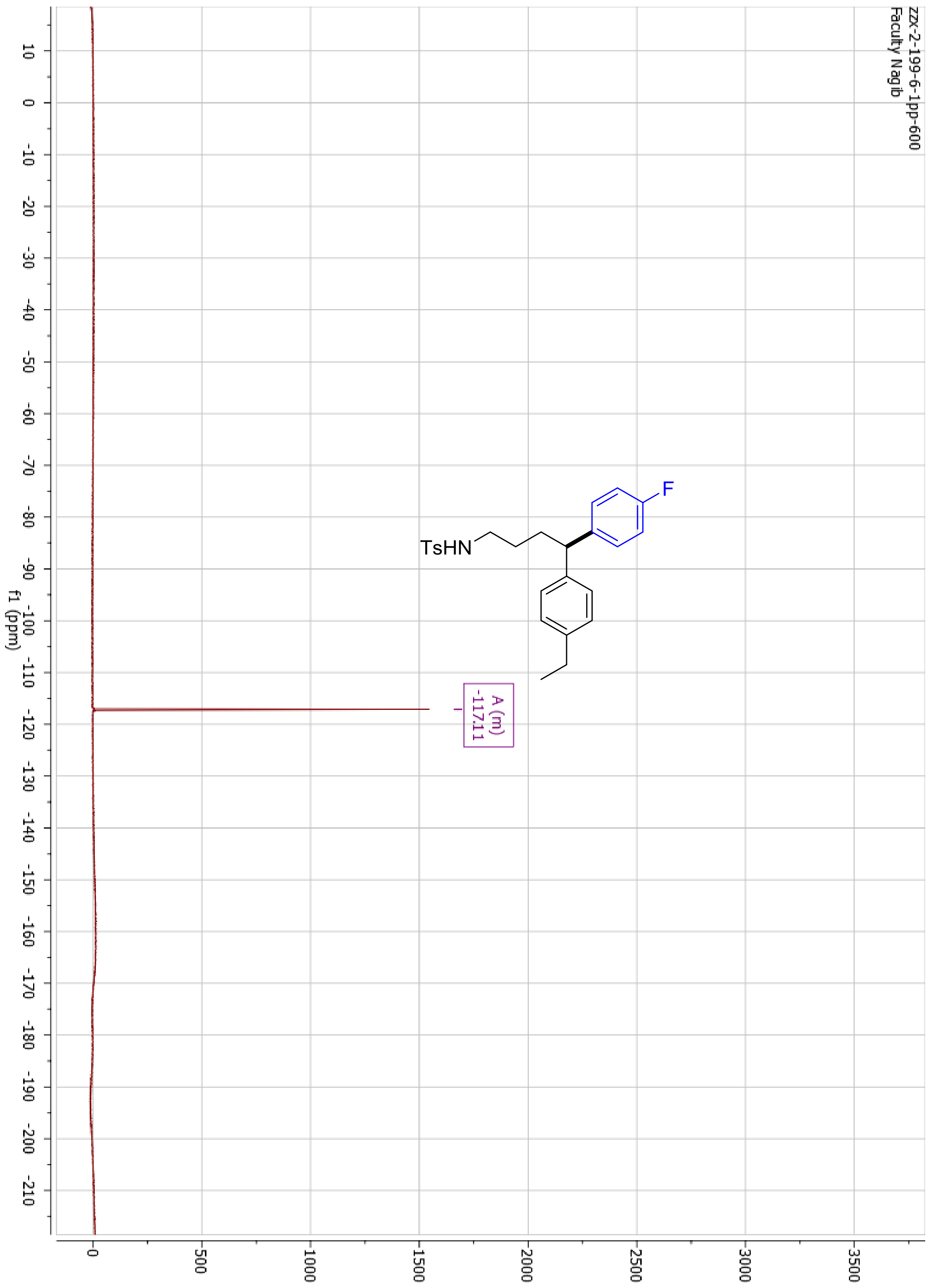
zzx-2-199-6-1pp-600
Faculty Nagib
TsHN
F
A (m)
-117.11
f1 (ppm)

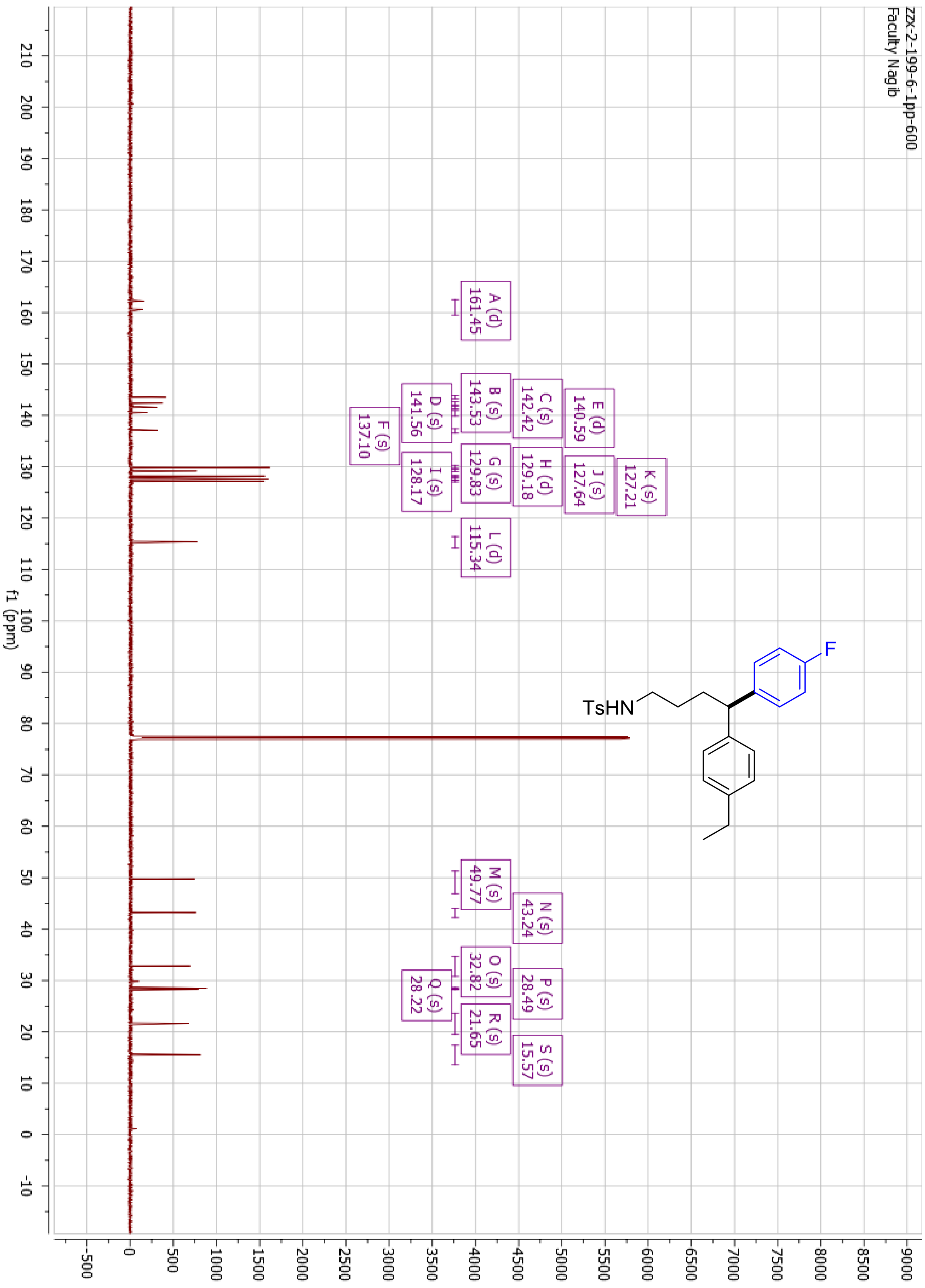

zzx-2-199-6-1pp-600
Faculty Nagib
A (d) 161.45
B (s) 143.53
C (s) 142.42
D (s) 141.56
E (d) 140.59
F (s) 137.10
G (s) 129.83
H (d) 129.18
I (s) 128.17
J (s) 127.64
K (s) 127.21
L (d) 115.34
M (s) 49.77
N (s) 43.24
O (s) 32.82
P (s) 28.49
Q (s) 28.22
R (s) 21.65
S (s) 15.57
TsHN
F
f1 (ppm)

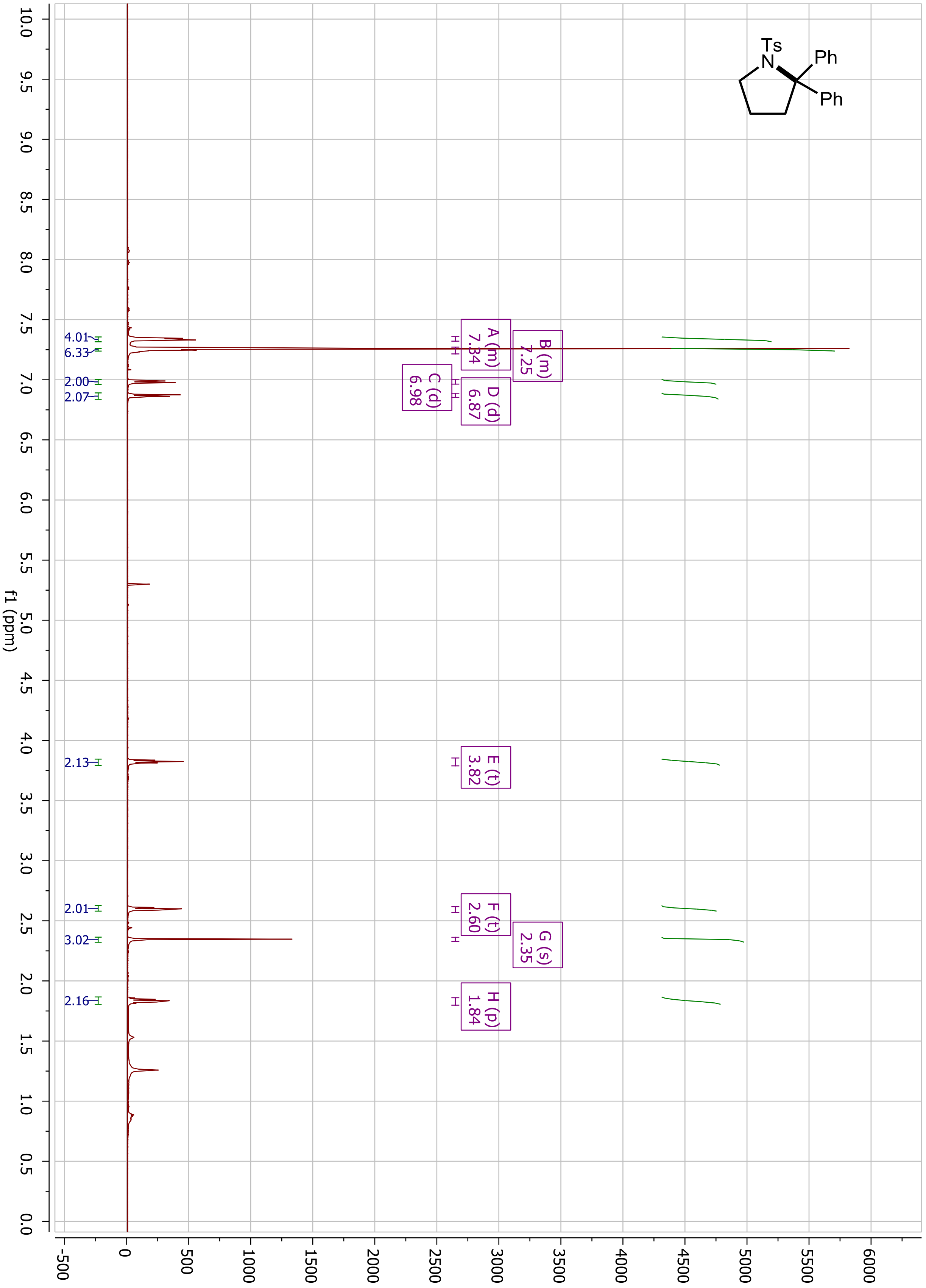
Ts
N
Ph
Ph
f1 (ppm)
A (m) 7.34
B (m) 7.25
C (d) 6.98
D (d) 6.87
E (t) 3.82
F (t) 2.60
G (s) 2.35
H (p) 1.84
4.01
6.33
2.00
2.07
2.13
2.01
3.02
2.16

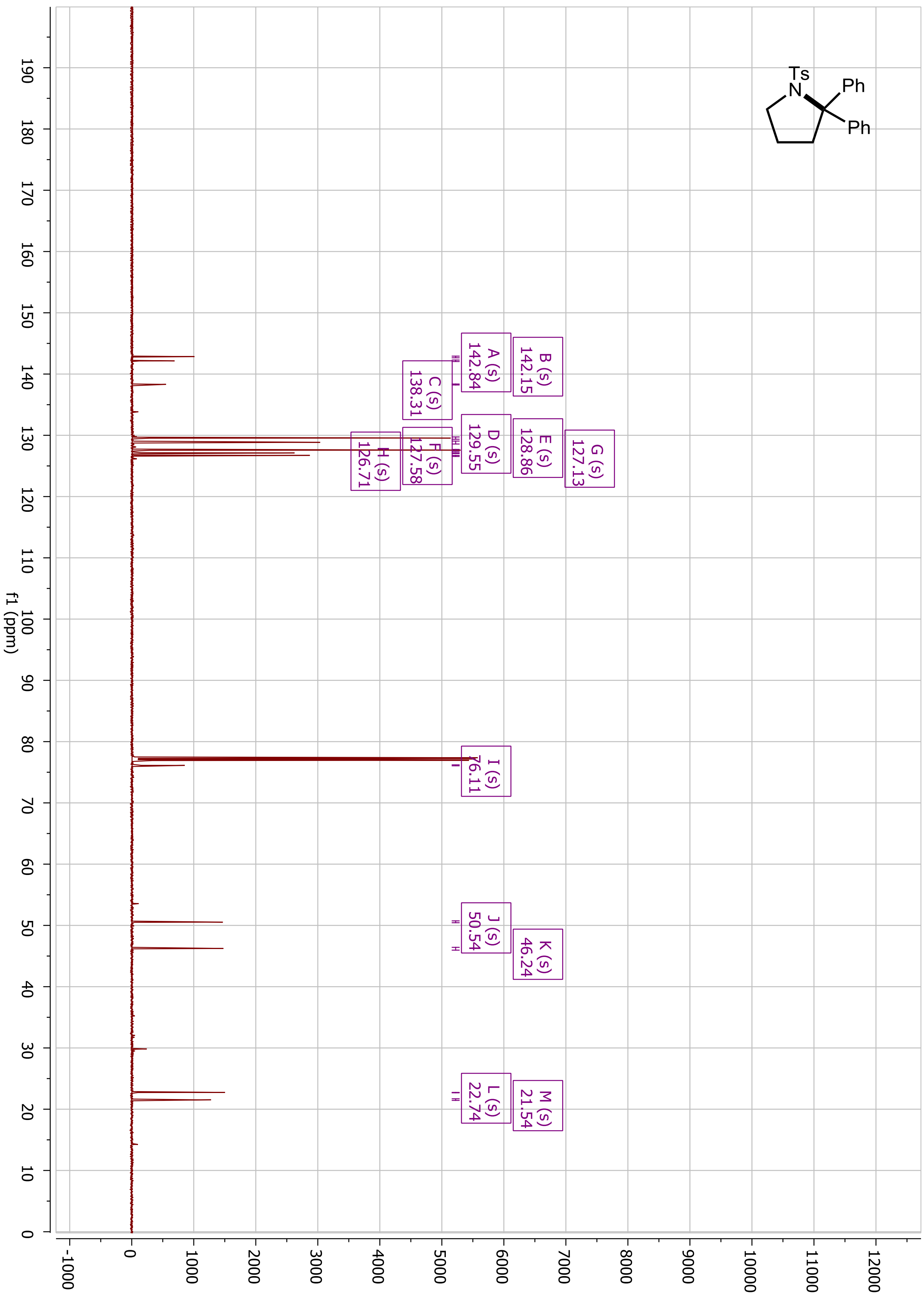
Ts
N
Ph
Ph
A (s) 142.84
B (s) 142.15
C (s) 138.31
D (s) 129.55
E (s) 128.86
F (s) 127.58
G (s) 127.13
H (s) 126.71
I (s) 76.11
J (s) 50.54
K (s) 46.24
L (s) 22.74
M (s) 21.54
f1 (ppm)

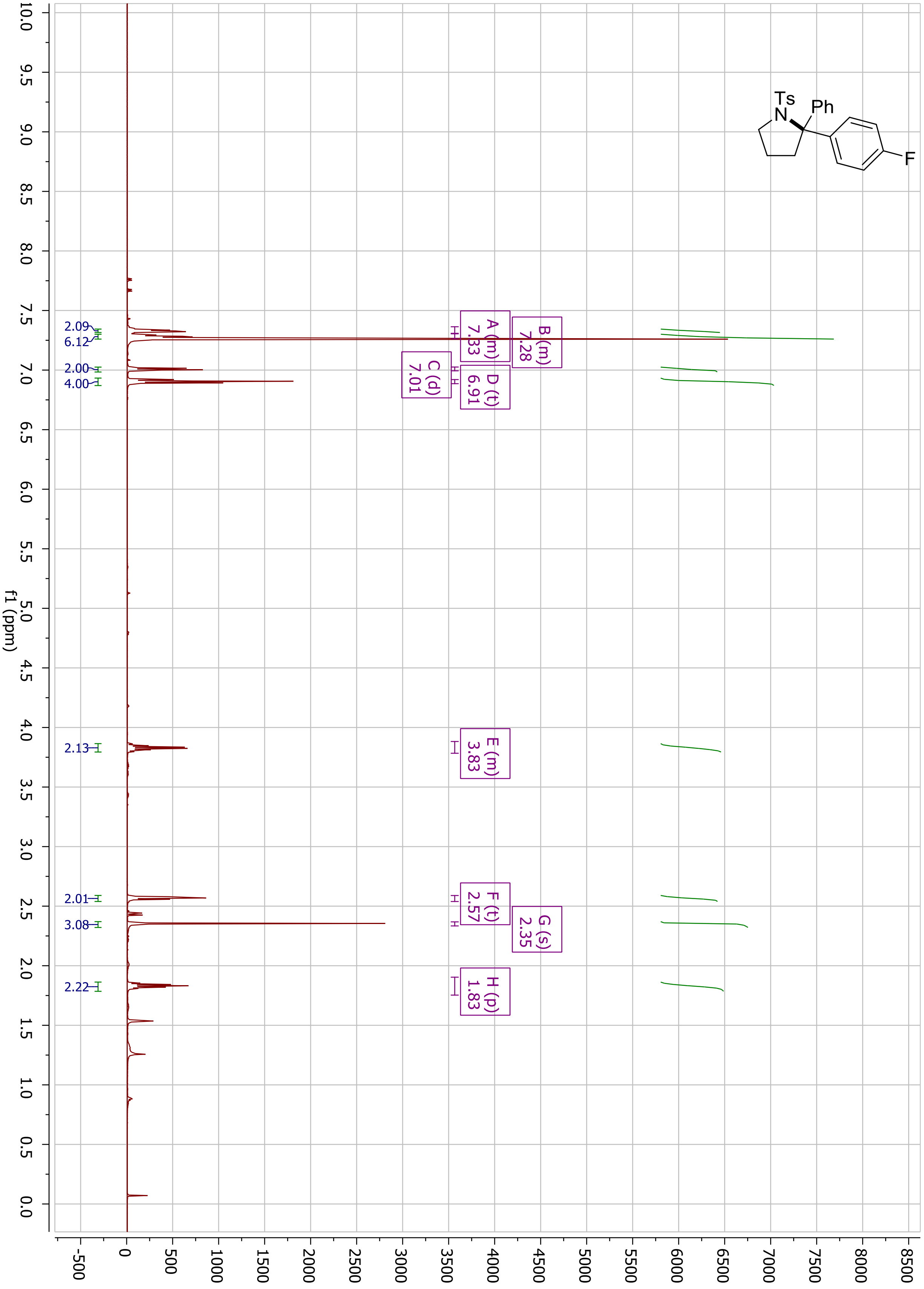
Ts
N
Ph
F
A (m) 7.33
B (m) 7.28
C (d) 7.01
D (t) 6.91
E (m) 3.83
F (t) 2.57
G (s) 2.35
H (p) 1.83
2.09
6.12
2.00
4.00
2.13
2.01
3.08
2.22
f1 (ppm)

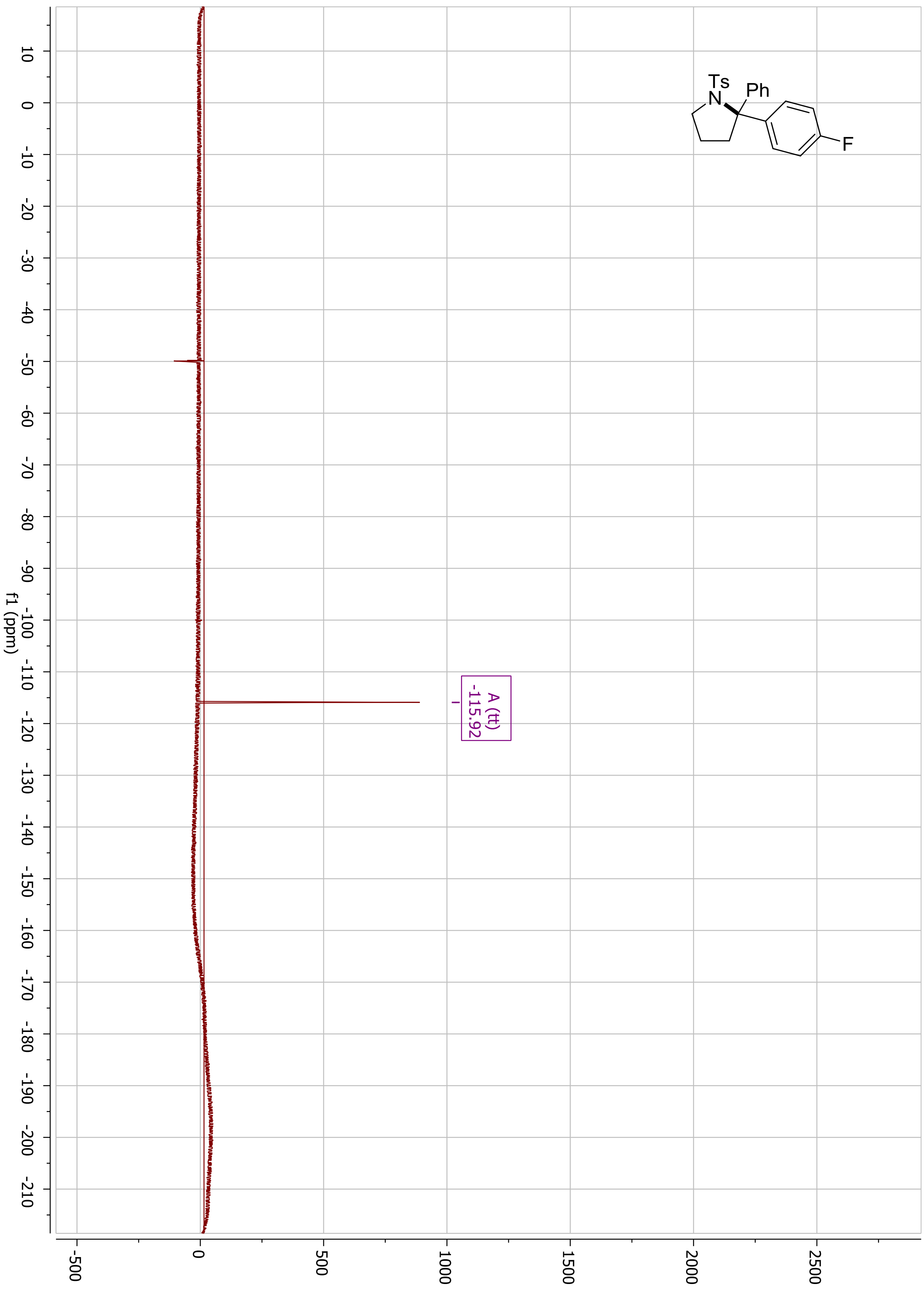
Ts
N
Ph
F
A (tt)
-115.92
f1 (ppm)

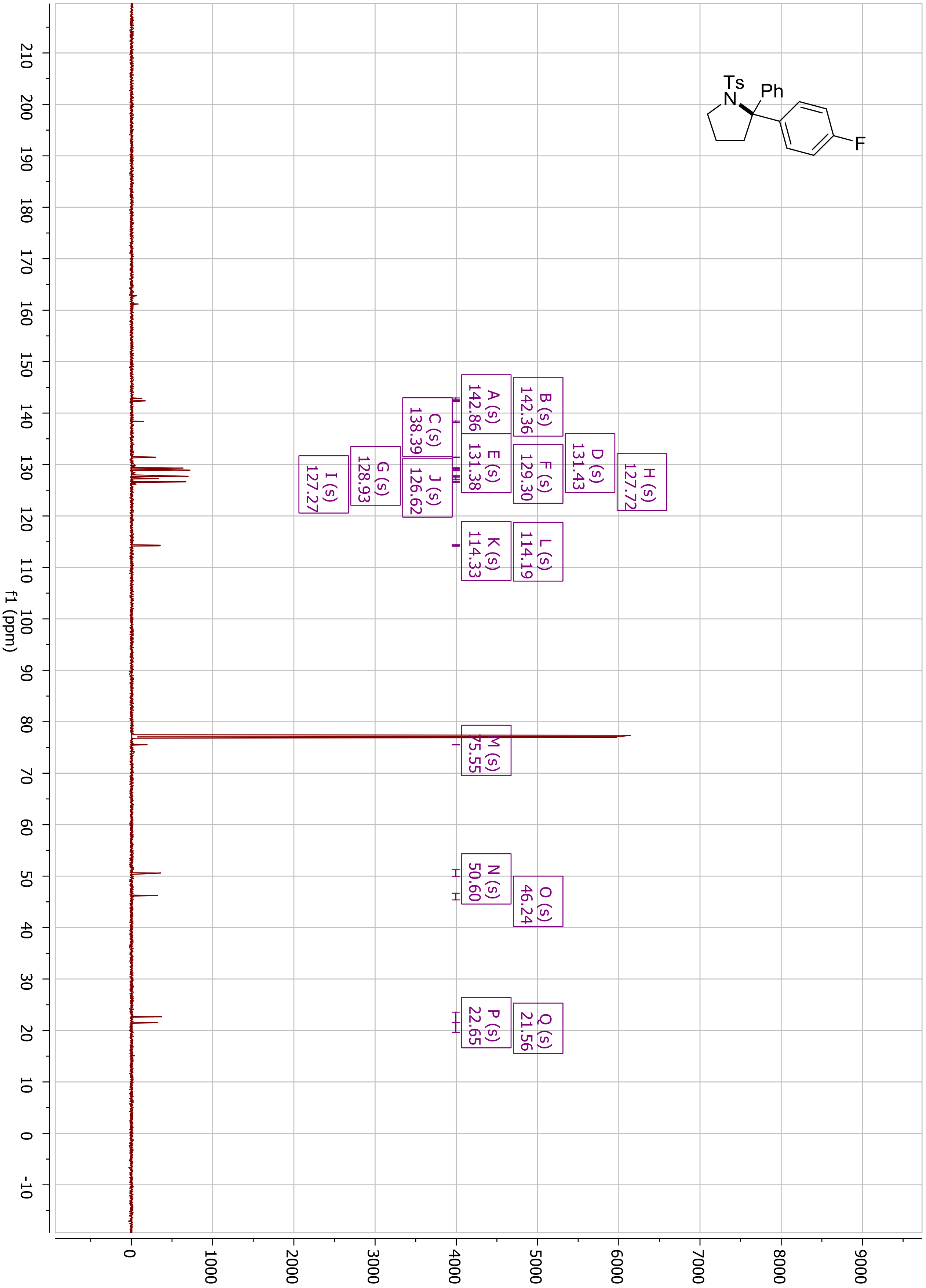

Ts
N
Ph
F
A (s) 142.86
B (s) 142.36
C (s) 138.39
D (s) 131.43
E (s) 131.38
F (s) 129.30
G (s) 128.93
H (s) 127.72
I (s) 127.27
J (s) 126.62
K (s) 114.33
L (s) 114.19
M (s) 75.55
N (s) 50.60
O (s) 46.24
P (s) 22.65
Q (s) 21.56
f1 (ppm)

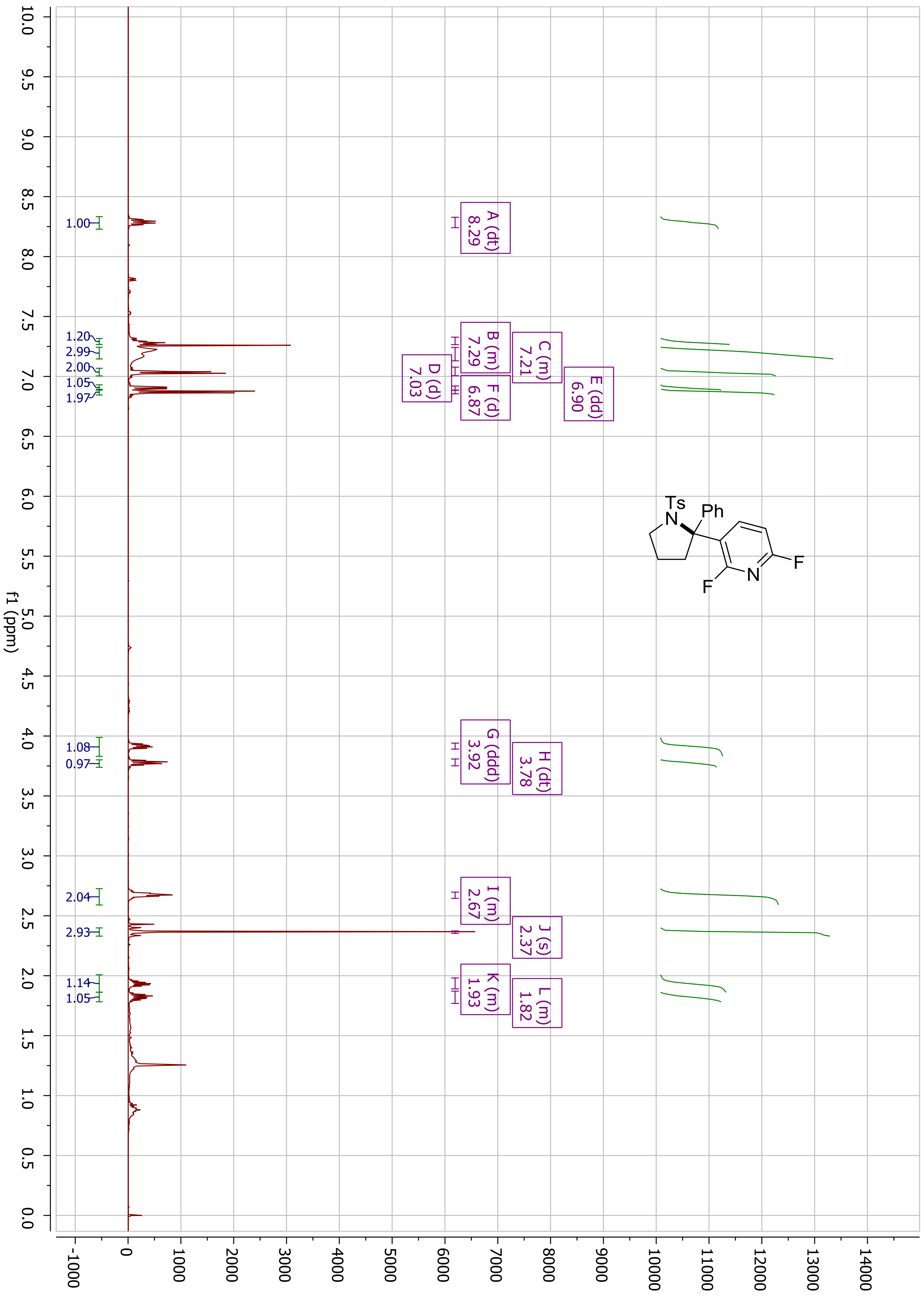
A (dt) 8.29
B (m) 7.29
C (m) 7.21
D (d) 7.03
E (dd) 6.90
F (d) 6.87
G (ddd) 3.92
H (dt) 3.78
I (m) 2.67
J (s) 2.37
K (m) 1.93
L (m) 1.82
1.00
1.20
2.99
2.00
1.05
1.97
1.08
0.97
2.04
2.93
1.14
1.05
f1 (ppm)
Ts
N
Ph
N
F
F

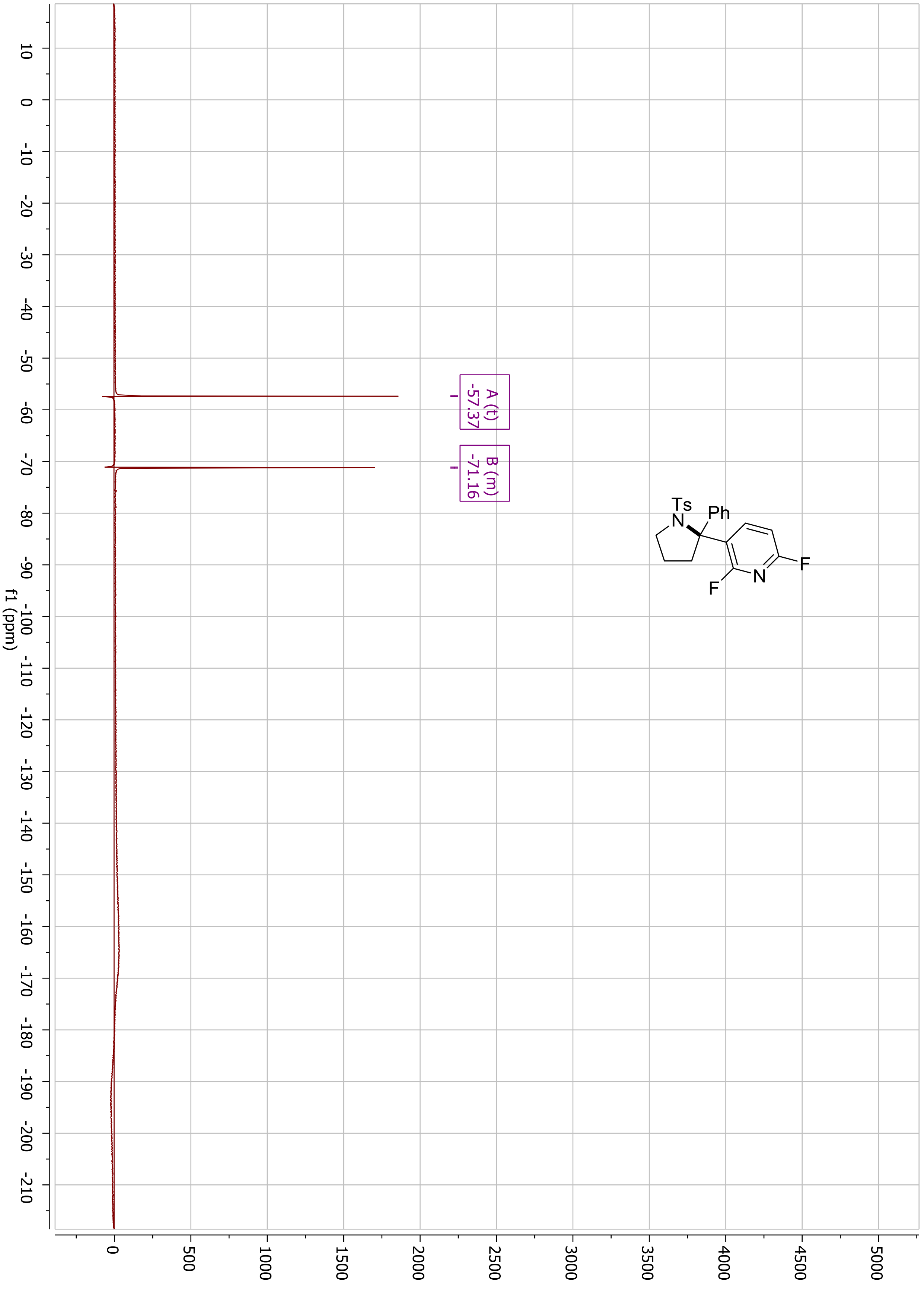
A (t)
-57.37
B (m)
-71.16
f1 (ppm)
Ts
N
Ph
F
N
F

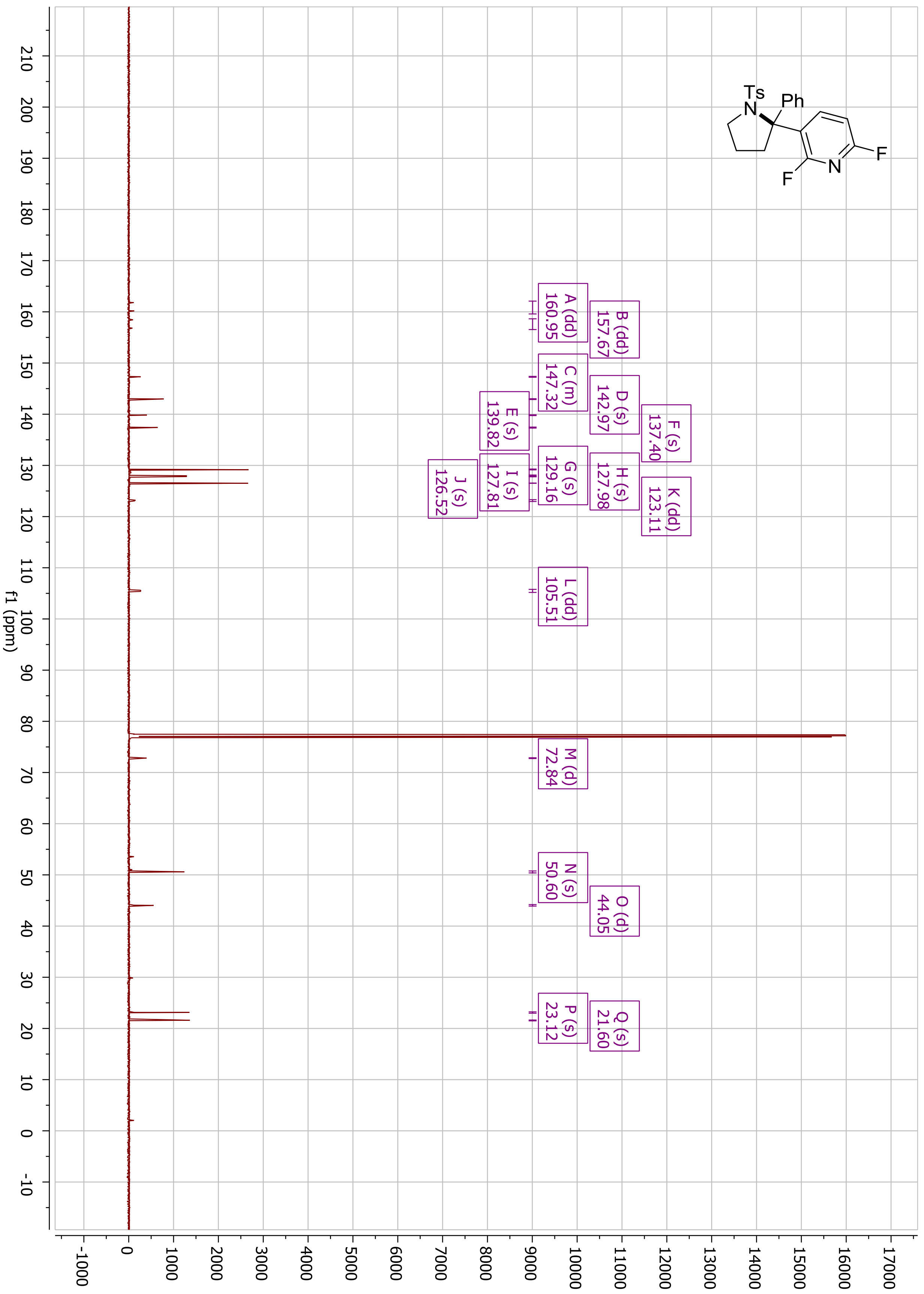

Ts
N
Ph
F
N
F
A (dd) 160.95
B (dd) 157.67
C (m) 147.32
D (s) 142.97
E (s) 139.82
F (s) 137.40
G (s) 129.16
H (s) 127.98
I (s) 127.81
J (s) 126.52
K (dd) 123.11
L (dd) 105.51
M (d) 72.84
N (s) 50.60
O (d) 44.05
P (s) 23.12
Q (s) 21.60
f1 (ppm)
